# Chromosome-Wide Impacts on the Expression of Incompatibilities in Hybrids of *Tigriopus californicus*

**DOI:** 10.1534/g3.116.028050

**Published:** 2016-04-11

**Authors:** Christopher S. Willett, Thiago G. Lima, Inna Kovaleva, Lydia Hatfield

**Affiliations:** Department of Biology, University of North Carolina at Chapel Hill, North Carolina 27599-3280

**Keywords:** postzygotic reproductive isolation, Dobzhansky-Muller incompatibilities, copepod, Mendelian ratio deviations

## Abstract

Chromosome rearrangements such as inversions have been recognized previously as contributing to reproductive isolation by maintaining alleles together that jointly contribute to deleterious genetic interactions and postzygotic reproductive isolation. In this study, an impact of potential incompatibilities merely residing on the same chromosome was found in crosses of populations of the copepod *Tigriopus californicus*. When genetically divergent populations of this copepod are crossed, hybrids show reduced fitness, and deviations from expected genotypic ratios can be used to determine regions of the genome involved in deleterious interactions. In this study, a set of markers was genotyped for a cross of two populations of *T. californicus*, and these markers show widespread deviations from Mendelian expectations, with entire chromosomes showing marked skew. Despite the importance of mtDNA/nuclear interactions in incompatibilities in this system in previous studies, in these crosses the expected patterns stemming from these interactions are not widely apparent. Females lack recombination in this species, and a striking difference is observed between male and female backcrosses. This suggests that the maintenance of multiple loci on individual chromosomes can enable some incompatibilities, perhaps playing a similar role in the initial rounds of hybridization to chromosomal rearrangements in preserving sets of alleles together that contribute to incompatibilities. Finally, it was observed that candidate pairs of incompatibility regions are not consistently interacting across replicates or subsets of these crosses, despite the repeatability of the deviations at many of the single loci themselves, suggesting that more complicated models of Dobzhansky-Muller incompatibilities may need to be considered.

The nature and structure of chromosomes can play an important role in the genetic patterns that underlie postzygotic reproductive isolation. One broadly observed example of this is the impact of sex chromosomes in hybrids, which leads to two important patterns: Haldane’s rule and the large X-effect. The large X-effect is the pattern of a disproportionately high number of Dobzhansky-Muller (DM) incompatibilities occurring on the X (or Z) chromosome relative to autosomes (Masly and Presgraves 2007; Good *et al.* 2008). Haldane’s rule is the observation that if one sex is inviable or sterile, it is most often the heterogametic sex, and relies on the recessive nature of most incompatibility interactions (Haldane 1922; Turelli and Orr 2000). These DM incompatibilities are deleterious interactions between alleles in the hybrid genome, and are thought to cause the lowered hybrid viability or fertility observed in crosses of genetically divergent taxa (Coyne and Orr 2004; Masly and Pregraves 2007). Taxa that lack sex chromosomes or have lower levels of sex chromosome differentiation (lower heteromorphism) show slower rates of build-up of postzygotic reproductive isolation relative to taxa that have highly differentiated sex chromosomes (Lima 2014). It is clear then that differentiated sex chromosomes have an impact on postzygotic reproductive isolation. However, it is less clear in taxa that lack sex chromosomes whether, when multiple DM incompatibility loci occur on the same chromosome, this distribution will influence the nature or accumulation of DM incompatibilities.

One way that chromosomal differences could contribute to postzygotic reproductive isolation is by chromosomal rearrangements, and these rearrangements could act in either a direct or indirect way. Chromosomal inversions can contribute directly to hybrid sterility via the disruption of meiosis, which is then associated with lowered hybrid fertility; however, it is unclear how often this occurs, particularly among animal species (Delneri *et al.* 2003; Faria and Navarro 2010). Indirectly, chromosomal rearrangements can limit recombination between divergent chromosomal regions in hybridizing taxa, holding together sets of alleles that can contribute to reproductive isolation (Noor *et al.* 2001; Navarro and Barton 2003; Strasburg *et al.* 2009).

In second-generation hybrid offspring (F2), the lack or lower levels of recombination in one sex could also influence the impacts of incompatibilities in these taxa. A wide range of taxa show either large differences in recombination rates between the sexes, or even the complete absence of recombination in one sex (Morgan 1914; Lenormand and Duthiel 2005). For heterogametic taxa, when recombination is absent in one sex it is almost always the heterogametic sex (Huxley 1928; Lenormand and Duthiel 2005), and this has been suggested as a mechanism to ensure continued opportunities for recombination across the entire X (or Z) chromosome in the homogametic sex (Lenormand and Duthiel 2005). In *Tigriopus californicus*, a species that lacks sex chromosomes, females lack recombination (Ar-rushdi 1962). Edmands (2008) found that recombinant backcrosses (hybrid male backcrosses) result in higher hybrid fitness in comparison to nonrecombinant backcrosses (hybrid female backcrosses). However, it is unclear if this difference could impact the expression of potential DM incompatibilities in this system.

*Tigriopus californicus* is a copepod that inhabits splash pools in the rocky intertidal pools along the Pacific coast of North America, and has been used extensively as a system to study the genetics of postzygotic reproductive isolation (Burton *et al.* 2006; Willett 2011a; Foley *et al.* 2013). Populations of this species can have high levels of genetic divergence, particularly for mtDNA (Burton 1998; Edmands 2001; Willett and Ladner 2009; Willett 2012). These genetically divergent populations, when crossed in the laboratory, show some level of hybrid inviability, particularly for F2 hybrids (Burton 1987, 1990; Edmands 1999). Interactions between the mtDNA genome and nuclear genomes appear to play an important role in DM incompatibilities in this system (Ellison and Burton 2008a; Burton and Barreto 2012), but there is also evidence for autosome–autosome interactions (Willett 2006; Foley *et al.* 2013).

In F2 hybrids of *T. californicus*, significant deviations from expected Mendelian inheritance are seen at many loci for adult hybrids, but not generally for the first larval stage (nauplii), indicating potential regions of the genome involved in DM incompatibilities (Willett and Burton 2001; Willett 2006; Harrison and Edmands 2006; Pritchard *et al.* 2011; Foley *et al.* 2013). This paper will focus on one particular cross in which previous studies have uncovered a marker, malic enzyme (*ME2*), with a dramatically high level of distortion in F2 hybrids and at least one other marker (*GOT2*) that showed a significant interaction with this *ME2* genomic region (Burton 1987; Willett and Berkowitz 2007; Willett 2011b). Previous studies of this cross have not examined this *ME2* marker in conjunction with other markers on the same chromosome, or markers on other chromosomes.

Here, we conducted a series of crosses with targeted genotyping of F2 hybrids from a cross of two populations of *T. californicus* to examine the wider chromosomal context of potential DM incompatibilities. First, a set of markers spanning the chromosome with the *ME2* marker and each of the other 11 chromosomes was examined to determine the extent to which the distortion detected at *ME2* extends into the rest of its chromosome, and to determine other potentially interacting genomic regions. Next, we looked at a full set of backcrosses of these two populations to determine the impact of sex-limited intrachromosomal recombination in the expression of this potential incompatibility associated with *ME2*, to explore potential interacting chromosomal regions. The results of this study provide a more complete view of the wider genomic impacts of hybridization, and the potential impacts of differing patterns of sex-limited intrachromosomal recombination on DM incompatibilities.

## Materials and Methods

### Copepod culturing and crossing

*T. californicus* copepods used for crosses were collected from intertidal rock pools at two southern California locations, San Diego (SD, 32.7457°N, 117.2550°W, San Diego County, CA) and Abalone Cove (AB, 33.7377°N, 118.3753°W, Los Angeles County, CA). The copepods were maintained in the laboratory in mass culture in artificial seawater (Instant Ocean, Aquarium Systems Inc.) in 400-ml beakers at 20° with a 12:12 light:dark photoperiod (L:D). Copepods were maintained at a saltwater concentration of 35 parts per 1000 (similar to normal coastal ocean salinities), and fed with commercial flake fish food. Copepods also consumed natural algal growth and detritus in these cultures.

A series of different crosses were set up between copepods from the AB and SD populations. The general practice used for these crosses was to first collect virgin females by separating clasped pairs to obtain putatively unmated females. These females were individually monitored for approximately a week after maturity to verify that they produced no offspring and were indeed virgin females. Females were then mated to males from the other population. After first generation hybrid nauplii were observed in a cross, males were removed. Females were removed when offspring reached the copepodid stage. With the exception of one cross (discussed below), the crosses were done at 20° with a 12:12 L:D cycle.

### F1 x F1 cross and iPlex gold SNP assays (DA1 and AD1)

**Figure 1:**
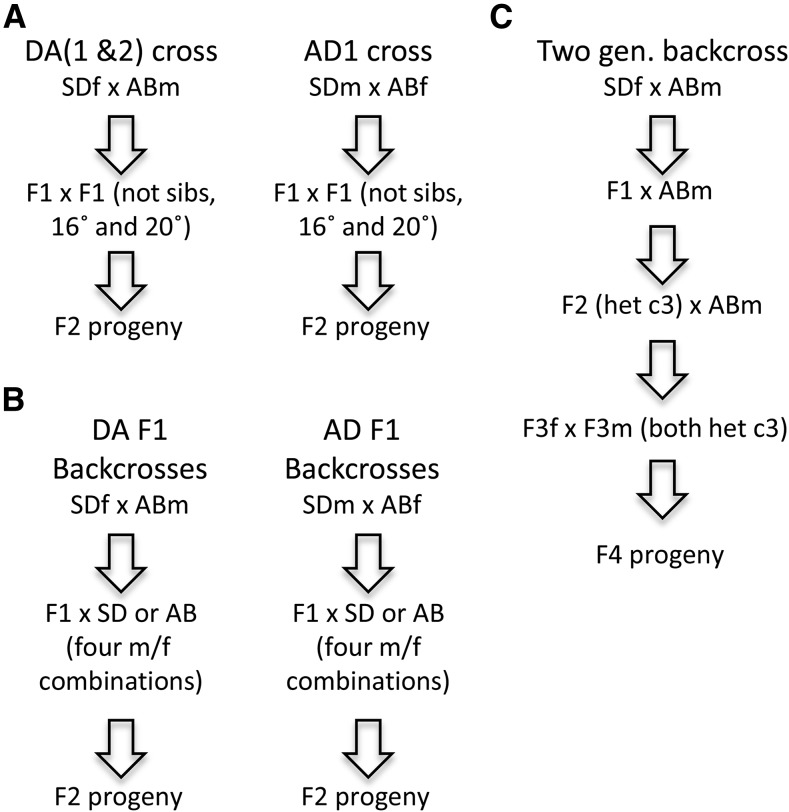
Crosses used for populations of *T. californicus*. (A) DA1, AD1, and DA2 crosses of the SD and AB populations with 16° and 20° environments used for the DA1 and AD1 crosses. (B) All eight possible backcrosses for the two reciprocal DA and AD crosses were performed. (C) Two generations of backcrosses to AB males were done, starting with DA F1 females, and genotyping of individuals was used to select for individuals that were heterozygous for the third chromosome (het c3 above). Note that there is no recombination in females in this species, so the entire chromosome should be transmitted intact.

One set of crosses was set up to generate F2 adult and nauplii hybrids to genotype for a set of SNPs covering all 12 chromosomes (Figure 1A). To generate F1 hybrids for these crosses, two Petri dishes, each with 20 females and 20 males, were set up for the two reciprocal crosses between copepods from the AB and SD populations. AB females crossed with SD males will be called AD, while SD females crossed with AB males will be DA. F1 hybrids were mixed across replicate dishes of the same cross to help minimize any chance of inbreeding. Collection dishes for F2 progeny were then set up in new Petri dishes starting with 20–25 mated F1 females. Replicated sets of these F2 collection Petri dishes were then placed at each of two temperatures (16° and 20°), and the female copepods left to produce progeny. Previous studies had shown some impacts of temperatures on the expression of incompatibilities and interactions (Willett and Burton 2003; Willett 2008), and we used these two temperatures to help determine the stability of patterns of interactions and marker distortion in the current crosses. F2 hybrid, first-stage nauplii were collected from the SDf × ABm cross at 20°. Adult male and female F2 hybrids were collected from each of the four crosses (two reciprocal crosses each with two temperatures) in the numbers recorded in Table 1. Note that males and females were collected as they matured and reflect the biased sex ratios produced for these crosses. Copepods were collected and placed into 20-μl of lysis buffer in 96-well plates for genotyping (Willett 2011b).

**Table 1:** AB × SD *T. californicus* hybrid cross sample sizes

Cross	Pool	Genotyped				Total Collected*^a^*	
		Female	Male	Total Adult	Nauplii	Female	Male
SDf × ABm (DA1)	16°	214	25	235		302	25
	20°	332	53	388	96	460	124
SDm × ABf (AD1)	16°	142	134	276		237	229
	20°	184	114	298		186	117
SDf × ABm (DA2)	1 d				165		
	2 d				139		
	Adult	137	87	224			
F1f (DA) × SDm		16*^b^*	16	54			
F1m (DA) × SDf		17	7	72			
F1f (AD) × SDm		34	53	206			
F1m(AD) × SDf				43			
F1f (DA) × ABm		53	36	43			
F1m(DA) × ABf				48			
F1f (AD) × ABm				119			
F1m (AD) × ABf				45			

a For the DA1 and AD1 crosses, a subset of the total progeny collected were genotyped. Total collected for each of these four cross/temperature combinations are given here to provide an estimate of the sex ratio of the progeny in each cross.

b For backcrosses, progeny were collected across several different independent crosses and sex was not recorded for several of these crosses.

A set of 32 SNP markers was selected that both spanned the 12 chromosomes of *T. californicus* and also included a number of other genes of interest. A subset of these markers were developed previously by the Edmands laboratory (Foley *et al.* 2011), and we also developed novel markers in conjunction with Jeffrey Conroy at the Roswell Park Cancer Institute (all markers are described in Supplemental Material, Table S1). We selected the novel markers in an attempt to expand upon potential DM incompatibility interactions detected in previous studies (Willett 2006, 2011b) that were either associated with complex III of the electron transport system (markers 2a, 3b, 4a, 4b, 6a, 6b, 8a, and 9), or potentially involved with the substrate malate and the aspartate/malate shuttle (markers 2b, 3d, 5a, 8b, 8c, 8d, 10a, and 12). Genotyping was performed in two pools using the iPlex Gold Assay on the MassARRAY compact (Sequenom) mass spectrometer at the Roswell Park Cancer Institute. DNA for genotyping was from a 10-μl subsample of DNA-prep/lysis buffer that was dried prior to shipping to Roswell Park for genotyping. Markers were tested against a panel of 48 control samples consisting of 16 AB, 16, SD, and 16 F1 hybrid individuals. Seven markers either failed, or were unreliable, and were excluded from subsequent analyses (four of these were used only for mapping purposes to help determine linkage patterns) leading to a set of 25 SNP markers that were retained for the full analyses.

Calculations of single locus deviations from expected 1:2:1 Mendelian ratios, and two- and three-locus deviations from independence were conducted as described in Willett (2006). For two-locus interactions the deviations of each single locus was accounted for prior to determining nonindependence between each pair of loci. Similarly, for three-locus interactions the two-locus deviations were taken into account first prior to determining nonindependence for each possible set of three loci. R scripts were written to compute these measures across all loci and are available upon request from the authors [R version 3.0.0; R Core Development Team (2015)]. The SNP markers were mapped relative to one another using the program MapDisto (Lorieux 2012) for DA F2 hybrid adults. Given the high levels of segregation distortion for many markers in these F2 adult hybrids, the distance values for this map reported in Table S1 should be regarded with caution (the ordering is, however, consistent with previous results as discussed later).

### First and second day nauplii crosses (DA2)

Crosses between females from the SD and males from the AB populations of *T. californicus* were set up to examine the pattern of segregation distortion across developmental stages (DA2 crosses; Figure 1A). For these crosses, a single pair of copepods (an SD virgin female and an AB male) was placed into each well of a 24-well culture plate. F1 progeny were separated to their own individual wells at the copepodid stage, and allowed to develop to the adult stage. Individual lineages of F1s were tracked, and F1 by F1 crosses were set up to avoid sib-mating. F2 individuals were collected from these F1 by F1 crosses as first and second day nauplii, and lysis preps were performed on these offspring (Table 1). F2 adults were also collected from an additional cross of SD females and AB males. Individuals were genotyped using PCR-based assays for the *3QCR8p* marker (closely associated with the 3b marker; note we will indicate the most proximate iPlex markers associated with these other markers for PCR-based assays in parentheses), a *3FBLRR* PCR-based marker (3d), and an *11sep_tub* PCR-based marker (11); primers and more details are given in Table S2.

### Backcrosses to test impact of chromosome 3

To explore the impacts of females lacking recombination on any potential DM inviability loci on chromosome 3, a set of backcrosses were conducted using F1 hybrids from the AB and SD population crosses (Figure 1B). These crosses were done in the same manner as the DA2 cross using 24-well culture plates. All eight possible combinations of backcrosses were performed using F1 males and females from the two parental reciprocal crosses (Table 1 gives all combinations and numbers of progeny collected for each). An initial set of up to 48 progeny was genotyped for each of these eight possible backcrosses for the markers *ME2* (3d) and *GOT2* (8d). Further crosses were done to extend and confirm the results from the initial set of backcross progeny for four of these backcrosses. These additional backcross progeny were initially genotyped for the *ME2* locus and/or the *3FBLRR* locus (both closely associated with marker 3d). A set of progeny from the AD F1f x SDm cross was further genotyped for a single marker on each of the 12 chromosomes (Table S2). This cross is a female backcross, so no recombination should occur between the two different populations’ chromosomes, and a single marker will be sufficient to characterize the genotype for each chromosome in each hybrid individual.

To further explore the impact of chromosome 3 and any potentially interacting chromosomes, a multigenerational backcross was done, and followed by a controlled genotype intercross with backcross generation two progeny (Figure 1C). For these crosses DA F1f × ABm backcross hybrid progeny were backcrossed for two generations to AB males. The hybrid female parent was scored in each cross, and only the crosses where the female was heterozygous for chromosome 3 were retained [as scored by genotyping *ME2* and/or *3FBLRR* markers (3d)]. Progeny of this second backcross generation were then intercrossed, and crosses were retained that were heterozygous for chromosome 3 in both parents. Four heterozygote/heterozygote lineages were obtained from these crosses and these produced 64, 51, 14, and six adult progeny. The parents and a set of progeny of these crosses were scored for a single marker on each of the 12 chromosomes of *T. californicus*.

### Data availability

The authors state that all data necessary for confirming the conclusions presented in the article are represented fully within the article. In addition to the summary results given in the Supplemental Material, genotyping data and R source code has been archived in the Dryad data repository at http://dx.doi.org/10.5061/dryad.gh2pb.

## Results

### iPlex SNP genotyping

To look for the potential impacts of DM incompatibilities and find potential interactions we scored F2 hybrid progeny from four different crosses between the AB and SD populations of *T. californicus* for a set of 25 markers spanning the 12 chromosomes of this species (consisting of two reciprocal crosses at two temperatures; Table 1 and Table S3). These 25 markers were pared down from an original set of 32 markers, seven of which were deemed unreliable based on control scoring or aberrant genotyping results. Genetic maps constructed from these markers were largely consistent with each other for the DA F2 adult, F2 nauplii, and AD F2 adult as well as the relative positions of the markers that were included in the map of Foley *et al.* (2011) that utilized the SD and a central California population, Santa Cruz, CA (SC; results for DA1 adult F2 in Table S1; others not shown). These results suggest that there may not be extensive large-scale rearrangements between the regions of the genomes surveyed by these markers in these genetically divergent populations.

**Figure 2:**
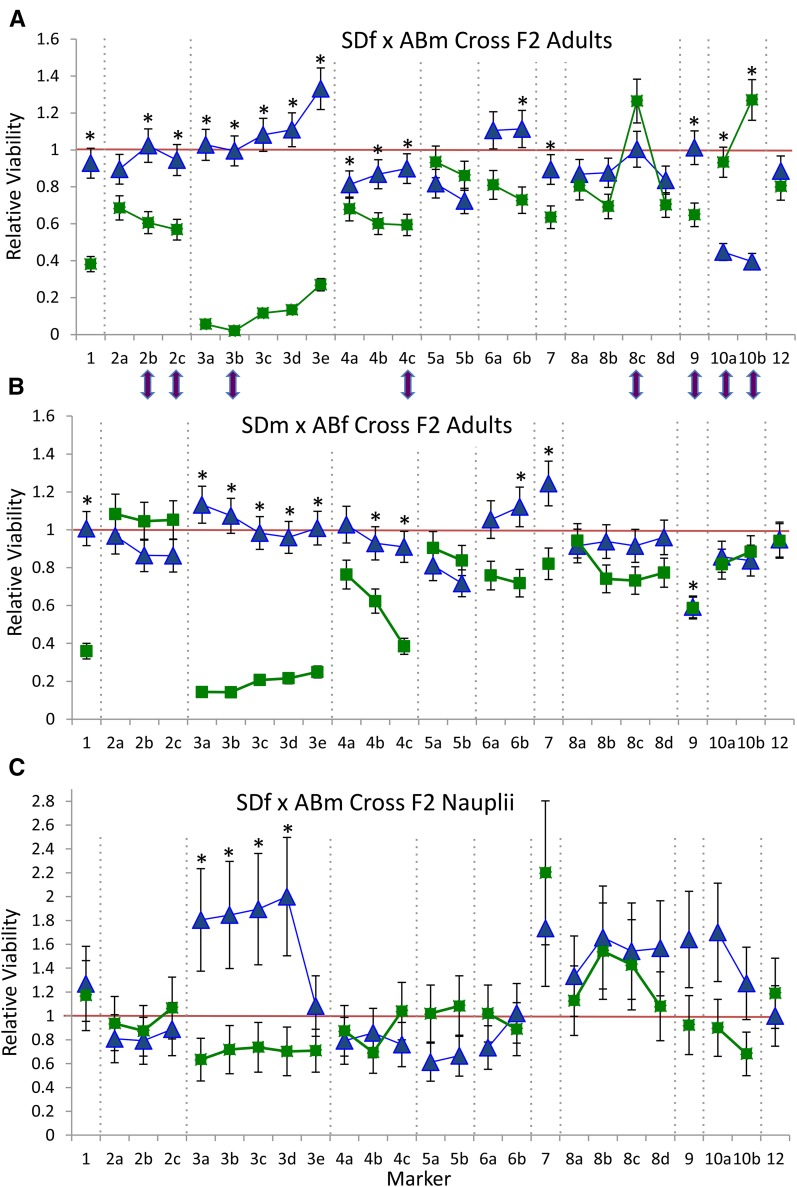
Relative viabilities of genotypes across iPlex markers in AB × SD F2 hybrids of *T. californicus*. Panel (A) shows the results for the F2 adults from the DA1 cross, (B) for the F2 adults from the AD1 cross, while (C) shows the results for F2 nauplii from the DA1 cross. Blue triangles give the relative viabilities of the AB/AB homozygous genotypic class in comparison to the expected 1:2 homozygote: heterozygote ratio, while green squares give the relative viabilities for the SD/SD homozygous genotypic class. The red line indicates the expected relative viability of one for each homozygote genotypic class. Relative viabilities and their SDs are calculated using Haldane’s (1956) formulation. An asterisk indicates a marker where genotypes differ significantly from the expected 1:2:1 ratio (*P* < 0.002 corrected *P*-value after applying a Bonferroni correction for 25 tests with α = 0.05 and 2 d.f.). Purple arrows show the markers for which there is a significant difference between the reciprocal crosses for genotypic ratios. This was tested in a 2 by 3 contingency table analysis with a critical *P*-value again of 0.002.

The genotypes of the F2 progeny at many of these 25 loci showed large deviations from expected Mendelian ratios, as well as some differences across reciprocal crosses. The results for the DA1 and AD1 crosses combined across the two different thermal environments (16° and 20°) show that 28 of 50 markers/cross combinations were significantly different from a 1:2:1 ratio for the F2 adults (Figure 2). The chromosome 3 markers show the most pronounced deviations, with very low viabilities for the SD/SD homozygous genotypes for all markers on this chromosome. Eight of 25 markers showed significant differences between the DA1 and AD1 reciprocal crosses, but many of these are also associated with sex (see next paragraph). Results across the two temperature treatments were largely similar, with no significant differences between 16° and 20° treatments for the DA1 cross, and only three markers showing significant differences for the AD1 cross between these same two treatments (Figure S2). These three markers showing deviations across temperatures were different in magnitude but not direction of skew for relative viability of each homozygous class. Although the sample size was more limited in comparison to the adult hybrids, the DA1 F2 nauplii did show significant deviations from Mendelian ratios for four chromosome 3 markers (Figure 2C).

**Figure 3:**
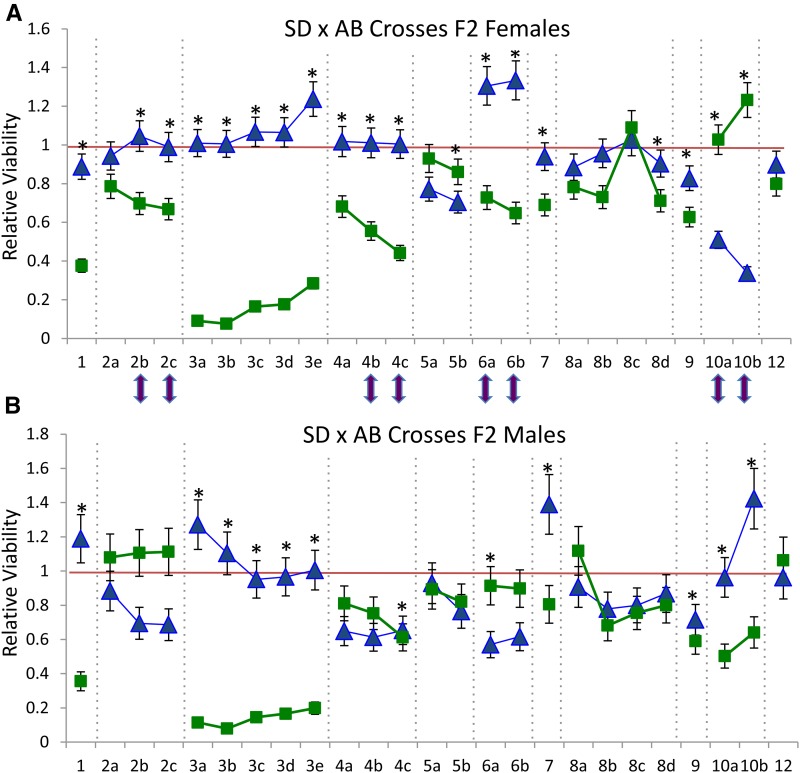
Sex impacts on relative viabilities of genotypes across iPlex markers in AB × SD F2 hybrids of *T. californicus*. Panel (A) gives the relative viabilities for all F2 females, while (B) gives the relative viabilities of all F2 males. Blue triangles give the relative viabilities of the AB/AB homozygous genotypic class, while green squares give the relative viabilities for the SD/SD homozygous genotypic class. The red line indicates the expected relative viability of one for each homozygote genotypic class. An asterisk indicates a marker where genotypes differ significantly from the expected 1:2:1 ratio (*P* < 0.002 corrected *P*-value after applying a Bonferroni correction for 25 tests with α = 0.05 and 2 d.f.). Purple arrows show the markers for which there is a significant difference between the two sexes for genotypic ratios. This was tested in a 2 by 3 contingency table analysis with a critical *P*-value again of 0.002.

The sex of the F2 hybrid offspring had a large impact on the nature of deviations observed across the 25 iPlex loci (Figure 3). Eight of 25 markers showed a significant impact of sex when reciprocal crosses were combined (five of these were markers with significant differences between reciprocal crosses). Figure S2 separates out the sex and reciprocal cross factors, but does not uncover any novel chromosomes displaying significant sex effects beyond the chromosomes highlighted by the combined analysis, chromosomes 2, 4, 6, and 10. For the DA1 and AD1 crosses, we sampled copepods as they matured, and did not equalize the numbers of males and females; we found substantially fewer F2 males in the DA1 cross due to the biased sex ratio of progeny that occurred in this cross (Table 1). Only two markers showed significant differences between reciprocal crosses when considered within a sex after a Bonferroni correction (in females, only markers 3b χ^2^ = 17.1, *P* = 0.0019, and 4c χ^2^ = 21.4, *P* < 0.001). Neither of these markers shows a reversal in viabilities consistent with the different mtDNA background in each reciprocal cross (mtDNA is contributed by the female parent; see Figure S2).

There were relatively limited numbers of significant two-way and three-way epistatic interactions detected in this dataset (Table 2). The two-locus interactions were calculated after having first removed the effects of the deviations observed for each single locus involved. In a similar way, three-locus interactions removed the effects of the two-locus interactions. With a Bonferroni correction for multiple tests, only a subset of interactions between markers on the chromosome pairs 2/3 and 3/4 showed significant interactions, and these were manifested only in limited subsets of the crosses. Five other pairs of chromosomes showed relatively strong deviations from independence, but these did not reach the Bonferroni correction level (Table 2), and numerous other pairs of loci were significant with lower levels of stringency (Table S4). For three-locus interactions no comparisons were significant with a Bonferroni correction, and only a fairly small number were significant at the *P* < 0.001 level (Table 2; full results in Table S5).

**Table 2:** Two- and three-locus interactions for iPlex markers for F2 hybrids in crosses of the AB and SD populations of *T. californicus*

		2-Way Loci			3-Way Loci			
Cross	Subset	L1*^a^*	L2	χ^2^ Value	L1	L2	L3	χ^2^ Value*^b^*
All	Females	None			4a	7	10a	36.1
	Males	2 (b,c)	3b	28.6-26.1^3^	2 (b,c)	3 (a,b)	4c	35.5-34.1
		2 (b,c)	3a	21.4-18.9				
		1	2b	18.6				
DA1	All adults	4 (a,b)	8a	20.9-18.6	None			
	16°	None			None			
	20°	1	3b	19.7	2 (b,c)	3a	5a	34.7-34.3
					3d	4b	8a	33.1
					4b	5a	6a	36.4
	Females	3e	4c	18.6	None			
		4b	8a	20.1				
	Males	None			None			
	Nauplii	None			5b	6a	8a	33.6
AD1	All adults	3 (c,d,e)	4 (b,c)	35.6-22.7*^c^*	3e	4c	8 (a,b,c)	36.0-33.8
	16°	6b	8a					
	20°	None			4c	9	10a	33.9
					6 (a,b)	7	10a	36.3-34.1
	Females	3e	4 (b,c)	30.9-23.4^3^	None			
		3d	4c	19.3				
	Males	2c	3b	20.8	1	3c	4c	33.4
		1	10a	19.2				

a L1, L2, and L3 refer to the interacting loci with marker designation given by the letters next to each chromosome number.

b No comparisons exceed the Bonferroni-corrected *P*-value of 0.00003 that with 12 d.f. corresponds to a χ^2^ value of 42.4. Comparisons shown are those with *P* < 0.001 (χ^2^ greater than 32.9). Full results can be found in Table S5.

c These comparisons exceed the Bonferroni-corrected *P*-value of 0.00018 based on 276 tests. With 4 d.f., the critical χ^2^ value is 22.3. All other comparisons correspond to *P* < 0.001 (χ^2^ greater than 18.5). Full results can be found in Table S4.

### Genotypes from first and second day hybrid nauplii

**Figure 4:**
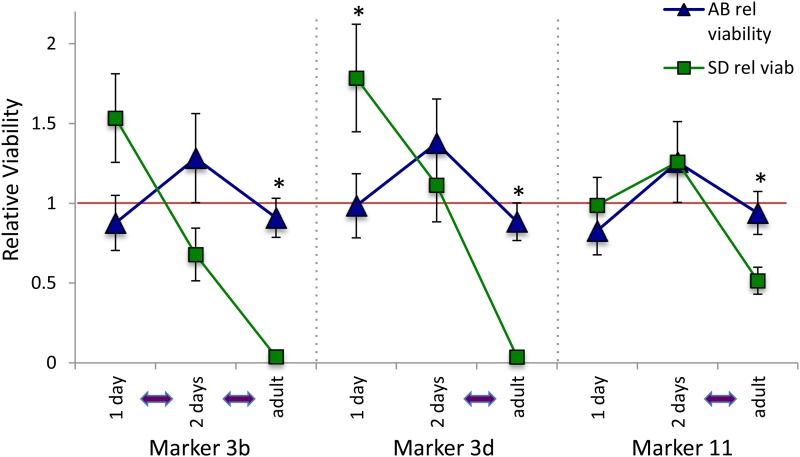
First and second day F2 hybrid nauplii relative viabilities. F2 hybrid nauplii and adults were collected from the SDf × ABm cross (DA2) of *T. californicus* populations, and genotyped for the PCR-based markers 3b, 3d, and 11. The red line again indicates the expected relative viabilities of one. An asterisk indicates a marker where genotypes differ significantly from the expected 1:2:1 ratio (*P* < 0.01 corrected *P*-value after applying a Bonferroni correction for five tests with α = 0.05 and 2 d.f.). Purple arrows indicate time periods over which there was a significant difference in genotypic ratios. Note that the 1–2 d period for marker 3d was approaching significance as well (*P* = 0.036). Tabular results are in Table S6.

We looked at the time course for the expression of incompatibilities by examining several markers in the earliest free-living life stage, F2 nauplii. Targeted genotyping of three different SNP markers showed that markers on chromosome 3 change quickly in their genotypic ratios over the course of development (Figure 4). The F2 hybrids for this targeted genotyping were from another replicate SDf × ABm cross (DA2). The three markers included two on chromosome 3 (3b and 3d) and a third on chromosome 11, and were scored using a PCR-based genotyping method (these did not score the exact same SNP as the nearby iPlex markers). Both markers on chromosome 3 were shifted in their genotypic ratios from the first to the second day (with one of the two markers showing a significant shift), and then another significant shift in genotypes from d 2 to adults. For the chromosome 11 marker, there was little change for the first and second day nauplii, but the genotypic ratios shifted significantly for adults. Another marker on chromosome 8 (c8_3336) showed no shifts in genotypic ratios across these three developmental points (included in Table S6).

### Backcross genotyping

**Figure 5:**
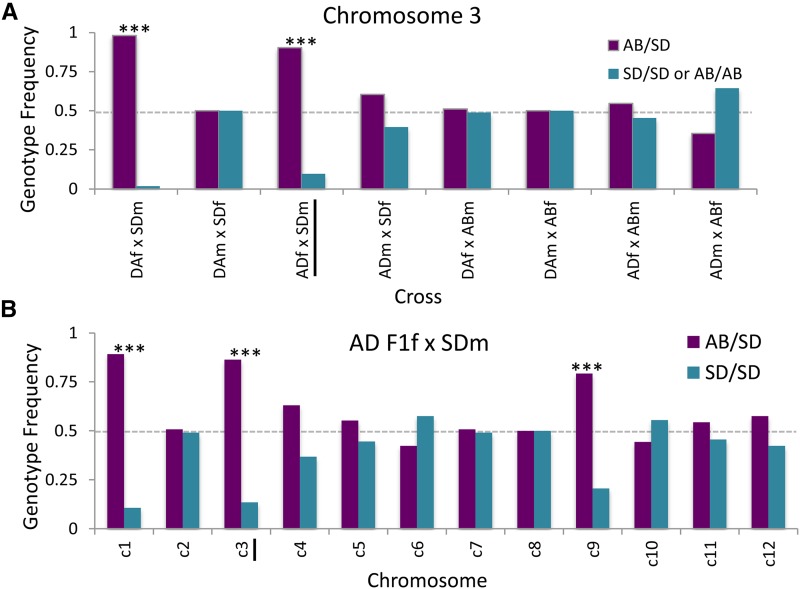
Backcrosses reveal the importance of intrachromosomal recombination for hybrids of *T. californicus*. (A) Progeny from the complete set of eight backcrosses genotyped for PCR-based marker 3d, with the genotypic frequency of the heterozygote and homozygote genotypic classes depicted. Only the two crosses using F1 hybrid females (with no recombination) show deviations from the expected 1:1 pattern of inheritance (*** denote a significant deviation with a Bonferroni correction). (B) A set of progeny from the AD F1f × SDm cross were scored for PCR-based markers on all 12 chromosomes, and the genotypic frequencies of heterozygotes and homozygotes at these 12 loci are shown. Two additional markers (c1 and c9) show significant deviations from the expected 1:1 ratios in these hybrid.

We took advantage of the lack of crossing over in females to determine whether markers that reside on the same chromosome could impact the expression of hybrid incompatibilities differently in male *vs.* female backcrosses. The patterns of segregation for two chromosomal markers were examined across the eight possible backcrosses of the AB and SD populations of *T. californicus*. The first marker, 3d, was highly skewed in two nonrecombinant backcrosses when F1 hybrid females were backcrossed to the SD population (Figure 5A). The second marker, on chromosome 8 (*GOT2*, 8d), showed no significant differences from expected patterns of inheritance across these eight crosses (Table S7). We then focused on a subset of individuals for the nonrecombinant backcross AD F1f × SDm, and genotyped a single marker per chromosome to determine if any associations between chromosomes could be found (particularly for the individuals that were SD/SD at marker 3d). While two additional chromosomes (1 and 9) were skewed in these backcross hybrids (Figure 5B), no significant associations were found between chromosomes (Table S8).

A final set of backcrosses was done to help determine whether other chromosomes had to cosegregate with chromosome 3 to maintain viability. These backcrosses were done for two generations for the nonrecombinant backcross, DA F1f × ABm cross (Figure 1c). In these crosses, individual hybrids that maintained the third chromosome in the heterozygous state were backcrossed to the AB population for two generations (the effect is to retain one allele of the SD 3^rd^ chromosome per line in an increasingly AB genomic background). The backcrossed progeny of these lineages were intercrossed and crosses that had parents that were both heterozygous for chromosome 3 were identified. The genotypic ratios at chromosome 3 and the other 11 chromosomes were examined in the parents and a set of progeny of these lineages. These crosses proved to be difficult to produce in appreciable numbers, and only four different chromosome 3 heterozygous by heterozygous lines were obtained. These lines showed evidence for significant skew at the 3d marker as well as skew at a few other chromosomes (Table 3). For the two lineages with relatively large progeny numbers (> 50), line 2 showed elevated AB/AB 3d frequencies, while line 1 showed both elevated AB/AB frequencies and lowered SD/SD 3d frequencies. For lines 1, 2, and 4, which produced at least some progeny that were SD/SD homozygous for marker 3d, it is interesting to look at the genotypes of the parents at the other chromosomes (Table 3 and Table S9). For the chromosomes 4, 5, and 12, SD alleles of each of these chromosomes could have been contributed by at least one parent in every line (at least one parent is heterozygous at these chromosomes in each line). If the region of the 3rd chromosome with the 3d marker is contributing to a DM incompatibility that is lethal, these three chromosomes could be candidates for harboring an interacting partner, as they could contribute an SD allele to rescue the incompatibility. Obtaining more lineages would be desirable to further test the robustness of these findings.

**Table 3:** Parental genotypes in second generation backcross hybrids between the AB and SD populations of *T. californicus* (DA F1f x ABm)

Chromosome	Marker		Line 1*^a^*	Line 2	Line 3	Line 4
1	c1_1718		AA	AA	HA	
2	c2_5		AA	AA	HH	AH
3	3d		HH*^b^*	HH*^b^*	HH*^d^*	HH
		AB rel. viab.	2.17	2.26	1.75	2.0
		SD rel. viab.	0.35	1.39	0	0.67
4	4a		HH*^b^*	HH*^c^*	AA	HA
5	P5CS		AH*^c^*	HA*^c^*	HA	HA
6	6a		HA	AH	HA	AA
7	c7_2276		AA	AA	HA	AH
8	8d		AH	AH	HH	AA
9	c9_2203		AH*^b^*	AA	AH	AA
10	c10_1464		AH	AA	AH	AH
11	11		AA	AA	AA	HH
12	12		HA*^c^*	AH*^b^*	HA	HA
		Total progeny	51	73	14	6

a The genotypes of the two parents for scored chromosome: AH = AB/AB female and AB/SD male parent; HA = AB/SD female and AB/AB male parent; HH = both parents AB/SD; AA = both parents AB/AB.

b The progeny in these lines showed a significant deviation from expected Mendelian ratios when correcting for multiple tests with a Bonferroni correction (19 tests, adjusted *P* = 0.0026), full results in Table S9.

c The progeny of these lineages showed no evidence for deviations from expected Mendelian patterns of inheritance (*P* > 0.05 with more than 20 genotyped progeny).

d No SD/SD homozygotes were found for this lineage at marker 3d, and *P*-value was 0.0092 for departure from 1:2:1 ratio.

## Discussion

### Chromosome-wide deviations for chromosome 3

One striking pattern to emerge from the analyses of the genetic markers in the DA1 and AD1 crosses of the AB and SD populations of *T. californicus* is the strong and potentially chromosome-wide deviations observed for chromosome 3 (Figure 2). Previous studies had consistently found that the SD/SD genotype at the *ME2* locus was nearly completely inviable in F2 adult hybrids from these two populations for both directions of the cross (Willett and Berkowitz 2007; Willett 2011b), and this study shows that this pattern extends to the entire surveyed portion of the 3rd chromosome. Results from complete genome sequencing of pools of F2 hybrids also confirm a strong AB allele bias across the entire 3rd chromosome for this cross (T. G. Lima and C. S. Willett, unpublished data). Based on the genetic map of Foley *et al.* (2011) for a different pair of populations (SD and SC), the markers used in this study extend close to both ends of the chromosome 3 linkage group. The linkage map distances for chromosome 3 markers are comparable to those obtained by Foley *et al.* (2011); also, chromosome 3 showed little evidence of deviations from expected Mendelian inheritance for this SD × SC cross (Foley *et al.* 2013). The comparison of these results suggests that recombination is similar for chromosome 3 for these two different F1 × F1 crosses (with recombination occurring presumably in the male F1 parent) that are nonetheless showing dramatically different patterns of genotypic viability for this chromosome.

Given the importance of sex chromosomes for the expression of DM incompatibilities in other systems (Coyne and Orr 2004; Masly and Presgraves 2007; Good *et al.* 2008; Lima 2014), it is interesting to ask whether the chromosomal regions that show strong deviations in these crosses are connected with sex determination in this *T. californicus* species. Clearly, a number of chromosomes show strongly biased genotypic ratios between males and females—in particular chromosomes 2, 4, 6, and 10 (Figure 3). Interestingly, these same chromosomes were shown to harbor sex determination QTL in a recent study by Alexander *et al.* (2015) that used a cross between SD and a population from British Columbia, Canada. Given that the SD population was used in both of these crosses (and chromosome 10 previously has been suggested to harbor a sex determination locus in a cross of SC and SD; Foley *et al.* 2013), our results lend further support to at least the SD population harboring sex ratio altering alleles on these chromosomes. However, these results do not suggest that the large impacts of markers on chromosomes 1 and 3 have any connection to sex, nor that chromosome 3 is likely to be a proto-sex chromosome.

In contrast to the results for F2 adults for chromosome 3, F2 nauplii show inconsistent deviations from Mendelian expectations in hybrids for markers on this chromosome. For the DA1 cross, four of five markers show an excess of AB/AB homozygotes (Figure 2), while in the DA2 cross, nauplii tend to show an excess of SD/SD homozygotes at *ME2* (marker 3d; Figure 4). Interestingly, the genotypic frequencies for a marker on chromosome 3 shift rapidly in the first 2 d after naupliar eclosion from the egg sac, suggesting the deleterious impacts of the 3rd chromosome in a hybrid genetic background might be particularly strong during this early free-living phase of the life cycle. Nauplii have undergone significant development in the egg sac before emerging as free-swimming organisms, and it is possible that maternal effects could lead to differences across clutches at hatching (perhaps mediated by different amounts of resources provided by different females). Previous studies had found either no or inconsistent deviations for first-day nauplii for the *ME2* marker in crosses of these and other related populations (Willett and Berkowitz 2007; Willett 2011b), while markers on other chromosomes have shown little evidence for deviations for markers across the genome in first-stage nauplii in this and other crosses of this copepod species (Pritchard *et al.* 2011; Foley *et al.* 2013; T. G. Lima and C. S. Willett, unpublished data).

The results from the progeny of first-generation backcrosses highlight the importance of females lacking recombination in *T. californicus* for the expression of potential DM incompatibilities on chromosome 3 (Figure 5A). It is only for crosses in which there is a hybrid female parent crossed to an SD male that there is a significant deviation from the expected 1:1 ratio for the progeny. For F1 hybrid male parents crossed to SD females, there is no evidence for a deviation from expected ratios. This observation implies that an intact chromosome 3 is important for the expression of this incompatibility in this context. In hybrid male parents there will be recombination between the AB and SD alleles for this chromosome, while in females the chromosome will be passed on to the offspring intact. These results could suggest that two or more factors on this chromosome (and potentially chromosome 1 as well) contribute jointly to hybrid breakdown in these backcross hybrids, and that the lack of intrachromosomal recombination in one sex can be an important factor for the expression of DM incompatibilities.

The importance of an intact chromosome for expressing a subset of DM incompatibilities parallels the impact that chromosomal rearrangements can have in maintaining a set of coadapted alleles that together can produce incompatibilities in hybrids (Noor *et al.* 2001; Navarro and Barton 2003; Strasburg *et al.* 2009). However, there is no evidence in these crosses of *T. californicus* that the chromosome-wide skew patterns are associated with chromosomal rearrangements or reductions in recombination in hybrid males. The genetic maps in crosses of three different highly divergent populations show the same relative ordering of markers, and no regions of highly clumped markers that could indicate rearrangements (Foley *et al.* 2011; Table S1). The contrast between the male backcrosses and female backcrosses (Figure 5A) also indicate that it is the lack of recombination in females, and not chromosomal rearrangements, that are responsible. A study by Edmands (2008) examined the fitness consequences of nonrecombinant backcrosses in *T. californicus* using a population from Laguna Beach, CA (genetically most similar to SD; Peterson *et al.* 2013), and the Royal Palms, CA population (very closely related to AB). She found that recombinant crosses had faster development rates than nonrecombinant crosses—a finding that is consistent with the pattern of incompatibilities that we observed in this study.

It is interesting to ask why there would be such strong inviability of SD/SD homozygotes at chromosome 3 in hybrids of F1 × F1 (*e.g.*, DA1 and AD1 crosses) and departures from the expected 1:1 ratio for only the nonrecombinant backcrosses of the two backcross types. One potential cause of this pattern is that in these backcrosses only homozygous (chromosome 3)/heterozygous (partner) DM incompatibilities would be expressed. It is possible that other homozygous/homozygous DM incompatibilities stem from chromosome 3 that would be expressed in F1 × F1 crosses. Previous work has suggested that the DM incompatibilities from the *ME2* region of the genome in this cross are likely to be complex, and involve more than one partner (Willett 2011a,b). Another possibility is that the level of the incompatibility expressed by individual DM incompatibility loci on chromosome 3 could differ in the different types of hybrids. If the degree of hybridity makes a difference in the expression of incompatibilities (Maheshwari and Barbash 2011), it could be that single interactions that are lethal in a genome with a higher level of hybridity (*i.e.*, the F1 × F1 hybrids) may not be lethal in the backcrosses, and additional DM incompatibilities would be needed to cause lethality (where 75% of the genome is from one parental population). If these additional loci reside on the same chromosome, limiting recombination would increase the percentage of times they segregate together in hybrids.

### Interactions between markers

There is little evidence for strong, consistent interactions between markers in this dataset that could indicate regions of the genome that are interacting to cause DM incompatibilities across all conditions and crosses (Table 2). The two-way interactions that were significant with a Bonferroni correction (between chromosomes 2/3 and 3/4) were not observed for all markers on these chromosomes, nor were they found in many subsets of these crosses. The second-generation backcross results do lend some further support to the possibility of an interaction between chromosomes 3 and 4, but more lines are needed to strengthen this conclusion (Table 3). Previous studies using hybrids from this pair of populations had shown significant interactions between *ME2* (3d) and *GOT2* (8d) in one dataset, and in another, between *CYC* (6a), *CYC1* (4a), *RISP* (8a), which are associated with the mitochondrial ETS complex III (Willett 2006, 2011b). Of these interactions, only the *CYC1* (4a) and *RISP* (8a) markers show a strong interaction in the present dataset, but only for the combined DA crosses. Deviations at single loci for crosses of *T. californicus* have sometimes shown a large degree of variation across repeated crosses (Willett 2008)—a pattern that contrasts with the consistently strong deviations associated with *ME2* in this AB × SD cross (Burton 1987; Willett and Berkowitz 2007; Willett 2011b). In this study, the repeated crosses across the two temperature regimes did not have a large impact on the size or direction of single-locus deviations (Figure S1), but they did appear to impact which sets of two- and three-way interactions between loci were significant (Table 2). Variation from cross to cross could result from uncontrolled environmental factors such as the algal/microbial community that develops in each culture and the density of the copepods (both of which can vary from culture to culture despite similar starting conditions).

Previous work looking at crosses involving populations of *T. californicus* has found both a number of nuclear/nuclear interactions as well as nuclear/mitochondrial interactions (Foley *et al.* 2013). Foley *et al.* 2013 used crosses between the SD population and the SC population from central California, and found significant two-way interactions between chromosomes 4 and 7 across both reciprocal crosses, as well as several significant differences between the reciprocal crosses that could be due to mitochondrial interactions. In most cases, markers from across each of these chromosomes showed similar patterns of interactions. They found 15 different chromosome three-way interactions that were significant with a Bonferroni correction, suggesting an increased number of more complex interactions were occurring in comparison to two-way interactions. These results suggest that the stability and strength of epistatic interactions could differ markedly across different crosses of populations of this copepod.

Although a number of studies have suggested that mtDNA/nuclear incompatibilities could play an important role in incompatibilities in hybrids in the *T. californicus* system (Rawson and Burton 2002; Ellison and Burton 2006; Barreto and Burton 2013; Barreto *et al.* 2014), the results of the current study do not provide any strong additional support for this idea in this cross. There are a number of markers that are significantly different in their patterns of deviation between the two reciprocal crosses (Figure 2); however, most of these differences appear to be driven by differences in patterns of deviations between the sexes in conjunction with the different sex ratios that were produced in these two reciprocal crosses (Figure 3 and Figure S2). Markers from two of the chromosomes that have the largest deviations in this dataset, chromosomes 1 and 3, show greatly reduced viability of SD/SD homozygotes on both mtDNA backgrounds, suggesting that a simple pattern of breakdown in mtDNA/nuclear coadaptation cannot explain the deviations seen for these chromosomes. Recent results do indicate that in *T. californicus* hybrids, multiple higher-level systems associated with mtDNA are disrupted, perhaps leading to an altered metabolic syndrome in hybrids rather than simple protein–protein interactions underlying incompatibilities (Barreto *et al.* 2014). These disrupted systems include mtDNA transcription, altered gene expression of genes involved in oxidative phosphorylation, and antioxidant response (Ellison and Burton 2008b; Barreto and Burton 2013; Barreto *et al.* 2014). One possibility (a bit speculative) is that particular genotypic combinations may not function properly in this altered metabolic environment in F2 hybrids, combinations that would be fine in either parental metabolic environment (and the severity of this could potentially differ depending upon the degree of hybridity as discussed earlier).

### Conclusions

This study has shown that the linkage of potential DM incompatibilities in a chromosome could have impacts on their expression in hybrids of the copepod *T. californicus*. Strong deviations were found for the entire 3rd chromosome, and, in backcross hybrids, these deviations were observed only when individuals inherited nonrecombinant chromosomes from both parents. As illustrated by difference between the backcrosses with males (with recombination) and females (no recombination), it is possible that a contribution to hybrid inviability is made by the chromosome holding together a set of loci that can jointly cause incompatibilities in a manner analogous to the role played by inversions in crosses of other species (Noor *et al.* 2001; Navarro and Barton 2003; Strasburg *et al.* 2009). However, it is unlikely that the sex-limited lack of recombination completely determines the patterns of viability at chromosome 3 in F1 × F1 crosses. Given the magnitude of the deviations in F2 hybrids for markers across chromosome 3, it is most likely that there are other factors that do not depend on having a complete 3rd chromosome, and these factors could interact with novel homozygous genotypes in these crosses (that cannot be produced in a backcross).

The regions of the genome that are interacting to cause these DM incompatibilities in this cross of *T. californicus* are less clear. In this study, a number of different regions of the genome show some evidence for interactions, but these are not consistently displayed across different subsets of the data. Additionally, strong two-way interactions that were observed in previous studies in this same cross were no longer detected in this dataset. This apparently sporadic expression of interactions stands in contrast to the consistently strong deviations for the single-locus effects stemming from the region of chromosome 3 marked by the *ME2* (3d) locus across a number of different replicates of this cross for these and closely related populations (Burton 1987; Willett and Berkowitz 2007; Willett 2011b; this study). Inconsistency could result from differences in density or culture conditions, as discussed previously. Alternatively, these results suggest that it is worth considering other DM incompatibilities model variants, including interactions with widespread genetic elements, novel responses that occur due to the unique nature of the hybrid genome overall (Maheshwari and Barbash 2011; Satyaki *et al.* 2014), or potentially a unique metabolic syndrome in hybrid individuals (Barreto *et al.* 2014).

## 

## Supplementary Material

Supplemental Material
