# Supplementary material for: Chromosome-Wide Impacts on the Expression of Incompatibilities in Hybrids of Tigriopus californicus
Source: G3 (Bethesda). 2016 Apr 11;6(6):1739–49. doi: 10.1534/g3.116.028050 (PMC4889669; doi:10.1534/g3.116.028050)
Supplement: Supplemental Material [file supp_6_6_1739__index.html]

Chromosome-Wide Impacts on the Expression of Incompatibilities in Hybrids of Tigriopus californicus — Supplemental Material 

# Chromosome-Wide Impacts on the Expression of Incompatibilities in Hybrids of *Tigriopus californicus*

## Supplemental Material for Willett *et al.*, 2016

**Files in this Data Supplement:**

- Figure S1 - Impact of temperature difference on relative viabilities in AD × SD F2 hybrids of *T. californicus*. (.pdf, 135 KB)
- Table S8 - Two locus genotypes and statistical associations for the AD F1f × SDm backcross. (.pdf, 482 KB)
- Table S9 - Second generation backcross intercross progeny (DA F1f × ABm cross). (.pdf, 484 KB)
- Figure S2 - Impact of sex on relative viabilities in AD × SD F2 hybrids of *T. californicus*. (.pdf, 132 KB)
- Table S1 - iPlex markers used for SNP genotyping of AB × SD F2 hybrids of *T. californicus*. (.pdf, 51 KB)
- Table S2 - PCR--based SNP scoring markers used to score F2 and backcross hybrids of *T. californicus*. (.pdf, 41 KB)
- Table S3 - Genotypic counts for iPlex loci scoring. (.pdf, 86 KB)
- Table S4 - Two-way interactions between iPlex markers. (.pdf, 517 KB)
- Table S5 - Three-way interactions between iPlex markers. (.pdf, 851 KB)
- Table S6 - First and second day nauplii genotypes and statistical tests. (.pdf, 477 KB)
- Table S7 - Observed genotypes at two markers in each of eight possible backcrosses. (.pdf, 478 KB)
