# Supplementary material for: Chromosome-Wide Impacts on the Expression of Incompatibilities in Hybrids of Tigriopus californicus
Source: G3 (Bethesda). 2016 Apr 11;6(6):1739–49. doi: 10.1534/g3.116.028050 (PMC4889669; doi:10.1534/g3.116.028050)
Supplement: Supplemental Material [file supp_g3.116.028050_FigureS1.pdf]

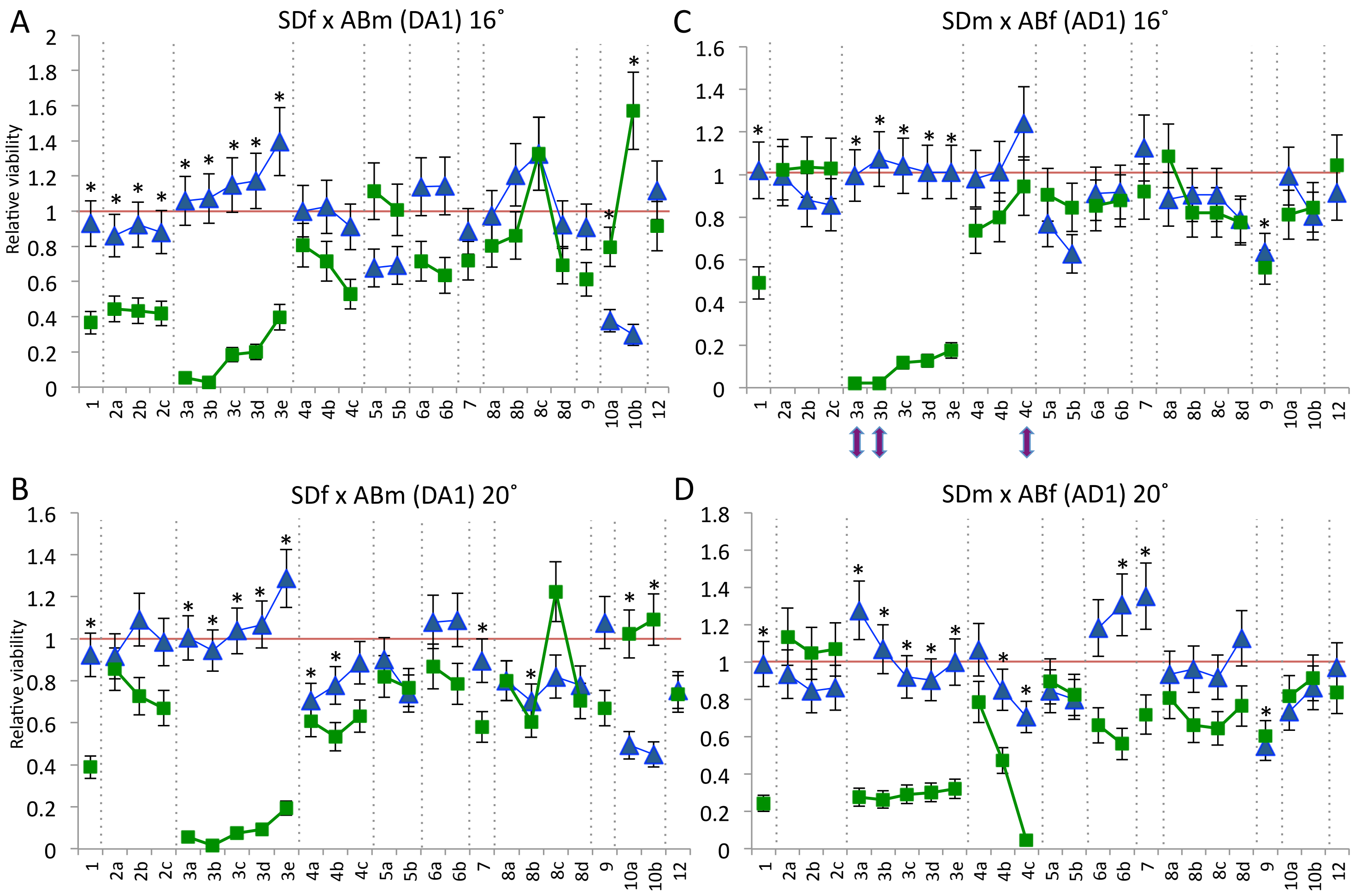

Supplemental Figure 1. Impact of temperature difference on relative viabilities in AD x SD F2 hybrids of *T. californicus*. (A) shows the results for the F2 adults from the 16˚ DA1 cross, (B) for the F2 adults from the 20˚ DA1 cross, (C) for the F2 adults from the 16˚ AD1 cross, and finally (D) for the F2 adults from

the 20˚ AD1 cross. Blue triangles give the relative viabilities of the SD/SD homozygous genotypic class, while green squares give the relative viabilities for the AB/AB homozygous genotypic class. The red line indicates the expected relative viability of one for each homozygote genotypic class. An asterisk indicates a marker where genotypes differ significantly from the expected 1:2:1 ratio ($P<0.002$ corrected P-value after applying a Bonferroni correction for 25 tests with $\alpha=0.05$ and 2 d.f.). Purple arrows show the crosses for which there is a significant difference between the temperature environments within a reciprocal cross for genotypic ratios. This was tested in a 2 by 3 contingency table analysis with a critical P-value again of 0.002.
