# Supplementary material for: Chromosome-Wide Impacts on the Expression of Incompatibilities in Hybrids of Tigriopus californicus
Source: G3 (Bethesda). 2016 Apr 11;6(6):1739–49. doi: 10.1534/g3.116.028050 (PMC4889669; doi:10.1534/g3.116.028050)
Supplement: Supplemental Material [file supp_g3.116.028050_TableS8.pdf]

Supplemental Table 8. Two locus genotypes and statistical associations for the AD F1f x SDm backcross. With a Bonferroni correction for 66 tests with α=0.05 and 2 d.f. the cutoff chi-square for significance is 14.4

first_locus	second_locus	H H	H S	S H	S S	rowSums(full	tchisq
c6_CYC	X11s	18	6	13	20	57	7.10089104
c6_CYC	c9_2203	15	9	31	3	58	7.05067136
c8_3336	c12_ME1	20	8	12	16	56	4.66666667
c3_composit	c12_ME1	27	24	7	1	59	3.38229239
c7_2276	X11s	13	16	18	8	55	3.31935769
c1_1718	c9_2203	40	9	3	3	55	3.13567869
c4_CYC1	c5_P5CS	17	10	7	11	45	2.51488095
c2_5	c3_composit	27	2	22	6	57	2.49342767
c8_3336	c10_1464	8	17	14	12	51	2.47977815
c7_2276	c12_ME1	14	15	19	9	57	2.24072156
c4_CYC1	c9_2203	26	9	19	2	56	2.1796633
c10_1464	X11s	16	8	13	15	52	2.14578425
c4_CYC1	c6_CYC	17	19	6	15	57	1.91683413
c7_2276	c8_3336	16	12	10	16	54	1.88455501
c1_1718	c6_CYC	19	31	4	2	56	1.81895477
c9_2203	c12_ME1	29	17	5	7	58	1.79292768
c4_CYC1	c12_ME1	18	18	14	7	57	1.49625
c1_1718	c10_1464	19	26	4	2	51	1.27763975
c6_CYC	c8_3336	14	10	14	18	56	1.16666667
c4_CYC1	c8_3336	15	20	11	8	54	1.11542593
c5_P5CS	c9_2203	22	3	16	5	46	1.1079198
c5_P5CS	c8_3336	11	15	11	8	45	1.0673737
c9_2203	X11s	22	22	8	4	56	1.05299145
c2_5	c4_CYC1	16	12	19	8	55	1.03930461
c1_1718	c3_composit	43	7	6	0	56	0.96
c8_3336	c9_2203	21	7	23	4	55	0.8912037
c3_composit	c10_1464	22	25	2	5	54	0.82066869
c5_P5CS	c12_ME1	14	12	14	7	47	0.7928475
c1_1718	c2_5	25	23	2	4	54	0.75
c2_5	c8_3336	15	11	13	15	54	0.68510748
c1_1718	c8_3336	23	24	4	2	53	0.66931361
c8_3336	X11s	13	13	17	11	54	0.62678571
c1_1718	c7_2276	24	24	4	2	54	0.59340659
c3_composit	c4_CYC1	30	19	6	2	57	0.56086006
c5_P5CS	c7_2276	13	12	13	8	46	0.45561905
c3_composit	X11s	28	22	3	4	57	0.42755051
c1_1718	X11s	26	23	4	2	55	0.39909297
c3_composit	c9_2203	39	11	7	1	58	0.37931159
c4_CYC1	c10_1464	14	20	9	9	52	0.37146133
c3_composit	c5_P5CS	22	19	4	2	47	0.35831919
c4_CYC1	X11s	19	15	10	11	55	0.35562899
c9_2203	c10_1464	19	23	4	7	53	0.27948115
c1_1718	c12_ME1	28	22	4	2	56	0.24888889
c10_1464	c12_ME1	13	11	18	12	54	0.185554
c2_5	c6_CYC	12	17	13	15	57	0.14750154
c2_5	c12_ME1	18	11	16	12	57	0.14362567
c5_P5CS	c6_CYC	10	16	7	14	47	0.1323278
c5_P5CS	X11s	13	12	12	9	46	0.12166531
c7_2276	c9_2203	22	6	23	5	56	0.11313131
c3_composit	c6_CYC	22	29	3	5	59	0.08999712
c7_2276	c10_1464	11	14	13	14	52	0.08987654
c5_P5CS	c10_1464	10	12	10	10	42	0.08677686
c1_1718	c5_P5CS	24	19	2	2	47	0.05004685
c6_CYC	c12_ME1	14	11	20	14	59	0.04703668
X11s	c12_ME1	17	14	15	11	57	0.04676334
c4_CYC1	c7_2276	17	17	10	11	55	0.02944696
c2_5	c10_1464	12	15	11	15	53	0.0246191
c2_5	X11s	15	13	15	12	55	0.0218254
c1_1718	c4_CYC1	31	18	3	2	54	0.0207443
c2_5	c9_2203	23	6	21	6	56	0.0195054
c2_5	c7_2276	14	14	14	13	55	0.01886145
c2_5	c5_P5CS	13	10	12	10	45	0.01778656
c6_CYC	c10_1464	10	13	14	17	54	0.01514727
c6_CYC	c7_2276	12	12	17	16	57	0.0127631
c3_composit	c7_2276	25	24	4	4	57	0.00286519
c3_composit	c8_3336	24	24	4	4	56	0
