# Supplementary material for: Chromosome-Wide Impacts on the Expression of Incompatibilities in Hybrids of Tigriopus californicus
Source: G3 (Bethesda). 2016 Apr 11;6(6):1739–49. doi: 10.1534/g3.116.028050 (PMC4889669; doi:10.1534/g3.116.028050)
Supplement: Supplemental Material [file supp_g3.116.028050_TableS9.pdf]

Supplemental Table 9. Second generation backcross intercross progeny (DA F1f x ABm cross). With the exception of the target chromosome 3, for other chromosomes relative viabilities, genotypic frequencies, and chi-square tests are only done for those lines that produced more than 20 genotyped progeny. Parents genotype is given with the female parent first.

Chromosome 1 (c1_1718)

	Line 1	Line 2	Line 3	Line 4
parents genotype	AA	AA	HA	
# AB/AB	51	73	6	0
# Het.	0	0	7	0

Chromosome 2 (c2_5)

	Line 1	Line 2	Line 3	Line 4
parents genotype	AA	AA	HH	AH
AB/AB	51	71	0	5
SD/SD	0	0	2	0
Het.	0	0	12	1

Chromosome 3 (3d, ME2)

	Line 1	Line 2	Line 3	Line 4
parents genotype	HH	HH	HH	HH
AB/AB	25	26	7	3
SD/SD	4	16	0	1
Het.	22	22	7	2
AB rel viability	2.17	2.26	1.75	2.00
SD rel viab	0.35	1.39	0.00	0.67
Chi-Square 1:2:1	18.25	9.38	7.00	2.00

Chromosome 4 (4a, CYC1)

	Line 1	Line 2	Line 3	Line 4
parents	HH	HH	AA	HA
AB/AB	36	16	14	5
SD/SD	3	17	0	0
Het.	11	35	0	1
1:2:1 chisq	59.24	0.09		
AB rel viability	6.00	0.89		
SD rel viab	0.50	0.94		

Chromosome 5 (P5CS)

	Line 1	Line 2	Line 3	Line 4
parents genotype	AH	HA	HA	HA
# AB/AB	19	40	9	2
# Het.	28	28	4	2
AB/SD freq	0.60	0.41		
SD/SD freq	0.40	0.59		
Chi-Square 1:1	1.72	2.12		

Chromosome 6 (6a, CYC)

	Line 1	Line 2	Line 3	Line 4
parents genotype	HA	AH	HA	AA
# AB/AB	8	9	7	6
# Het.	10	8	7	0

Chromosome 7 (c7_2776)

	Line 1	Line 2	Line 3	Line 4
parents genotype	AA	AA	HA	AH
# AB/AB	51	18	7	3
# Het.	0	0	7	1

Chromosome 8 (8d, GOT2)

	Line 1	Line 2	Line 3	Line 4
parents genotype	AH	AH	HH	AA
AB/AB	18	38	3	6
SD/SD	0	0	2	0
Het.	8	14	8	0
AB/SD freq		0.73		
SD/SD freq		0.27		
Chi-Square 1:1		11.08		

Chromosome 9 (c9_2203)

	Line 1	Line 2	Line 3	Line 4
parents genotype	AH	AA	AH	AA
# AB/AB	5	60	5	3
# Het.	19	0	9	3

Chromosome 10 (c10_1464)

	Line 1	Line 2	Line 3	Line 4
parents genotype	AH	AA	AH	AH
# AB/AB	12	60	4	4
# Het.	7	0	6	2

Chromosome 11 (11, 11sep_tub)

	Line 1	Line 2	Line 3	Line 4
parents genotype	AA	AA	AA	HH
AB/AB	51	18	14	1
SD/SD	0	0	0	4
Het.	0	0	0	1

Chromosome 12 (12, ME1)

	Line 1	Line 2	Line 3	Line 4
parents genotype	HA	AH	HA	HA
# AB/AB	13	8	1	4
# Het.	16	46	13	1
AB/SD freq	0.55	0.85		

SD/SD freq	0.45	0.15
Chi-Square 1:1	0.31	26.74
