# Supplementary material for: Chromosome-Wide Impacts on the Expression of Incompatibilities in Hybrids of Tigriopus californicus
Source: G3 (Bethesda). 2016 Apr 11;6(6):1739–49. doi: 10.1534/g3.116.028050 (PMC4889669; doi:10.1534/g3.116.028050)
Supplement: Supplemental Material [file supp_g3.116.028050_FigureS2.pdf]

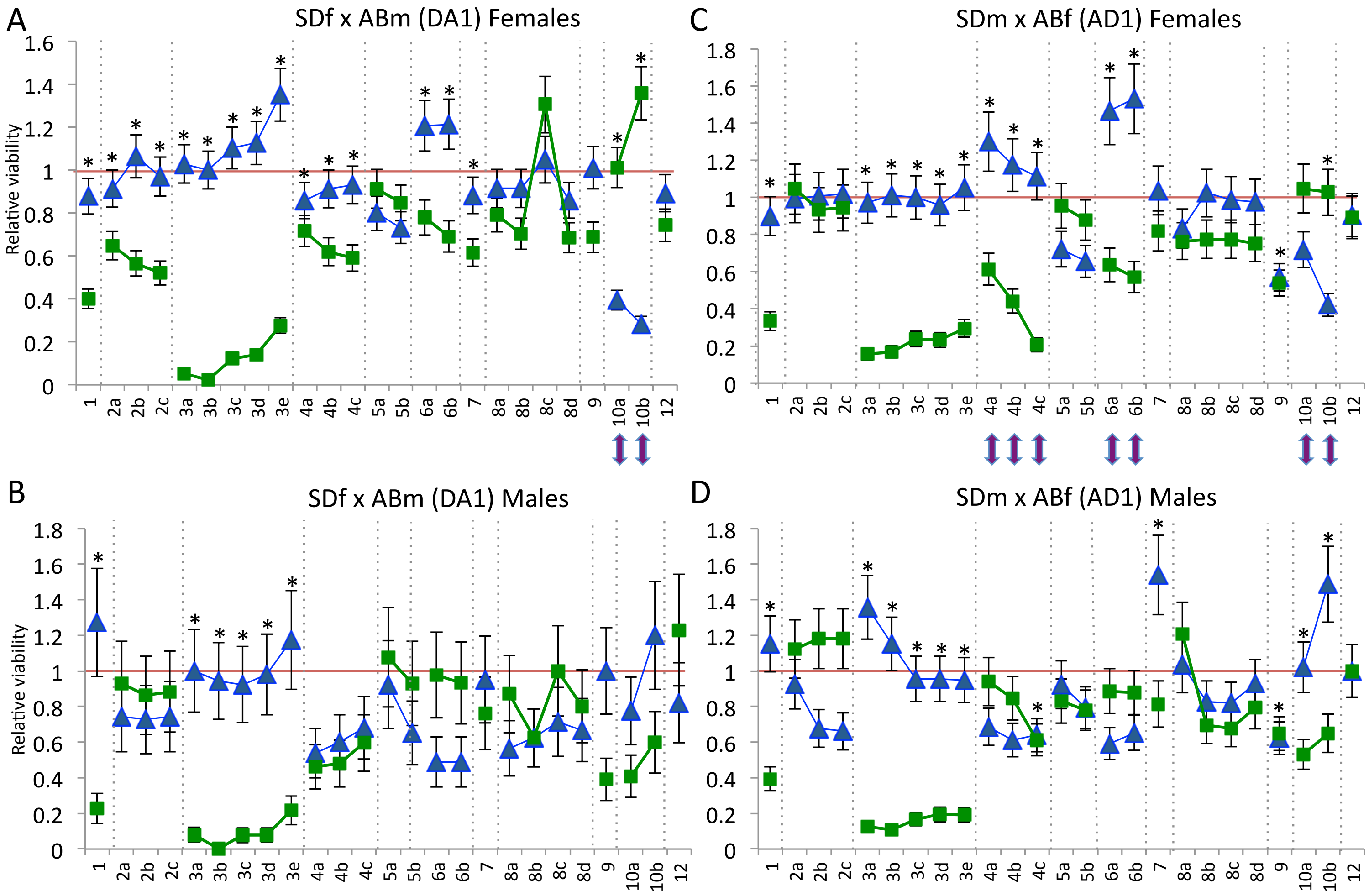

Supplemental Figure 2. Impact of sex on relative viabilities in AD x SD F2 hybrids of *T. californicus*. (A) shows the results for the F2 females from the DA1 cross, (B) for the F2 males from the DA1 cross, (C) for the F2 females from the AD1 cross, and finally (D) for the F2 males from the AD1 cross. Blue triangles give the relative viabilities of the SD/SD homozygous genotypic class, while green squares give the relative viabilities for the AB/AB homozygous

genotypic class. The red line indicates the expected relative viability of one for each homozygote genotypic class. An asterisk indicates a marker where genotypes differ significantly from the expected 1:2:1 ratio ($P<0.002$ corrected P-value after applying a Bonferroni correction for 25 tests with $\alpha=0.05$ and 2 d.f.). Purple arrows show the crosses for which there is a significant difference between the sexes within a reciprocal cross for genotypic ratios. This was tested in a 2 by 3 contingency table analysis with a critical P-value again of 0.002
