# Supplementary material for: Chromosome-Wide Impacts on the Expression of Incompatibilities in Hybrids of Tigriopus californicus
Source: G3 (Bethesda). 2016 Apr 11;6(6):1739–49. doi: 10.1534/g3.116.028050 (PMC4889669; doi:10.1534/g3.116.028050)
Supplement: Supplemental Material [file supp_g3.116.028050_TableS1.pdf]

Supplemental Table 1. iPlex markers used for SNP genotyping of AB x SD F2 hybrids of *T. californicus.* AP3 scaffold indicates the scaffold from the current assembly of the genome that contains the marker (genome scaffolds can be found at https://i5k.nal.usda.gov/Tigriopus_californicus)

Marker	Marker Number	SNP pool	Source	Chrom	Foley et al. m	DA adult ma	AP3 scaffold	Primer1	Primer2	Extension Sequence	SD	AB	Comments on reliability
TcGDHad	1	1	This paper	1	18.862		40	ACGTTGGATGGTGCGATGATGCGAATGCTG	ACGTTGGATGCTTCCCTCTTCAACGAGATG	CCTCTTCAACGAGATGATGGCTC	C	A	reliable
TcQCR9p	2a	2	This paper	2		0	274	ACGTTGGATGACGTCCACCTTCTTCTTGTC	ACGTTGGATGATAGATTTGATCGGCCACCC	TCAAAGGTCCGCTCGAA	C	G	reliable
TcmtMDH	2b	2	This paper	2		12.8	540	ACGTTGGATGTTGCTGCTCAAGCTCAATCC	ACGTTGGATGTCGCAATGGGACAGATCGG	GTGCACGATGTCGTA	G	A	reliable
P060_S200	2c	1	Foley et al.	2	15.113	13.6	3	GAGGACCCATTTTTCCACAC	TCTTTTACAAGCACTCCTC	GAAGTCATCACACACG	G	A	reliable
Tc30317_p392_rAyG		1	Foley et al.	3	3.487	0	64	ACGTTGGATGCACACTTCACTGATGTTCCC	ACGTTGGATGGAAAGGTGTTCAACGACTTC	CCGAGATTAGAGAACTCA	A	G	some issues with controls; appears unreliable; kept only
P102_S185	3a	2	Foley et al.	3	13.769	14.7	1075	GCGTCAACGATGTTGAGAAC	TGGTGCCCATACATGAGTTC	GAACTTCATGGCCTTGAAGAT	A	T	reliable
P099_S262		1	Foley et al.	3	15.493		202	TAGCAATCTTCGGTCACCAC	CAGCCCATTATGATTGCTCG	GGGTTTCCACGGGATA	T	C	unreliable; replaced by P102_S185
P125_S465		2	Foley et al.	3	20.302	**	42	TTGTTGGACTTGATGCCTCG	GCTCGTATTGCTCGATCTTG	ATGTGGACAAATTGAA	G	A	appears unreliable; one control misscored; kept only for
TcQCR8p		1	This paper	3			9	ACGTTGGATGCATGGTGGCAAAAAAAGAAC	ACGTTGGATGCATCTTGATGGGCTGAAATG	GGCTGAAATGAACACAA	T	A	unreliable; replaced by TcQCR8p_2 genotype
TcQCR8p_2 genotype	3b	2	This paper	3		19.4	9	ACGTTGGATGTTTCTGCCGTGTAGTAGACC	ACGTTGGATGGGTTCGCCAGTCGGCTCAT	CCAGTCGGCTCATGTCTGT	A	C	reliable
Tc22708_p256_rTyC	3c	1	Foley et al.	3	25.971	25.5	15	ACGTTGGATGGCCGCTGTTTGTAGTCATAC	ACGTTGGATGGTTCACTCAACGATGCTCTC	GAGCAACACCATGAAC	T	C	reliable
TcME2ad	3d	1	This paper	3	26.638	25.9	15	ACGTTGGATGCCATGGAAAAACGGGAAGGC	ACGTTGGATGCGATCCTTGACAATCAGACC	AGTCTTTCATCCAGATCTGCTTTCG	T	A	reliable
Tc14140_p806_rAyG	3e	2	Foley et al.	3	29.902	32.1	136	ACGTTGGATGTGAAAGCCTCGAACATCCTC	ACGTTGGATGTTGTGGCTCAAAAGCGCCC	AAGCCTCGAACATCCTCGTCCCA	A	G	reliable
TcCYC1	4a	1	This paper	4		0	62	ACGTTGGATGTGTTCATGACGGAGGACGAG	ACGTTGGATGATTTGCCGGGTCGATCGAAC	ATCGAACATCTCGCC	A	C	reliable
TcQCR10p	4b	1	This paper	4		14.4	411	ACGTTGGATGGGATCCGGATTGATGAGTTC	ACGTTGGATGGAATAAGACGGCCAATCCAG	TACTGGGCAAGCTGAAATCAA	C	A	reliable
P169_S291	4c	1	Foley et al.	4	1.621	20.8	33	GTAGCCCCTCAAGGTTATGC	TCACGCTTGGCAACATGGAG	AAGGGCCAATATGTGGC	T	C	reliable
TcGOT1Srg	5a	1	This paper	5		0	466	ACGTTGGATGTCCTCATACTTGAACCTTG	ACGTTGGATGCCATTTGGTATATTGTAAAC	GCATTTATGAACAATGTTTTCAA	T	A	reliable
Tc34449_p230_rTyC	5b	1	Foley et al.	5	11.047	6	336	ACGTTGGATGTAGCGTTGGAAGCTGCTTTG	ACGTTGGATGCGGAGTATGATGATGATGTC	AAGCTGCTTTGGCTGAAGATAG	T	C	reliable
TcCYCad	6a	1	This paper	6	2.969	0	262	ACGTTGGATGTACCTTTGGACTTGTTGGCG	ACGTTGGATGAACCTCCACGGCATGTACGG	CAGACGGGCAAGGC	T	C	reliable
TcQCR7p	6b	2	This paper	6		0.4	262	ACGTTGGATGACAACCCGTACTGGTTGAAG	ACGTTGGATGACCCTCAAGATGTACTACGG	GGCAACGAAAGCACGGA	A	G	reliable
Tc05997_p430_rTyA		1	Foley et al.	7	13.612		0	ACGTTGGATGAAGAGCATGAAGACGCACTG	ACGTTGGATGTGGTGATGGTATACACCTCG	AGGTACAAAAACAGGGGATCCACTA	T	A	unreliable; replaced by RPOL_864
RPOL_864	7	2	Foley et al.	7	12.25		435				G	T	appears reliable; two controls misscored
TcRISP_p1671_rTyC	8a	2	Foley et al.	8	30.802	0	34	ACGTTGGATGTGCACACACCGATCACAATC	ACGTTGGATGTCGTTGCGCGATCCTCAAAC	TCACAATCAAGAACTTGGGGTCCTT	T	C	mostly reliable; one control misscored
TcGOT1p1	8b	1	This paper	8		22.8	125	ACGTTGGATGGGATCGGAGAAGTAGAACAC	ACGTTGGATGTTTGGCGTTCAATCGCTGAG	GTTCCTGTTCCGTCAATT	G	A	reliable
TcGOT1p2	8c	1	This paper	8		32	links to 527	ACGTTGGATGCAGTCCATGATTACCCCAAG	ACGTTGGATGATGGAGCCGAATTTCTAGTC	GCCGAATTTCTAGTCCAACAA	T	C	reliable
TcGOT2_p391_rTyA	8d	1	Foley et al.	8	9.943	37.8	554	ACGTTGGATGACGTTCCGTCCACTTGAAAC	ACGTTGGATGATACGCTCCAATTGGAGGTG	AAATGCTAAGTTAGCGGA	T	A	reliable
Tc28594_p741_rTyG		2	Foley et al.	9	19.359	0	228	ACGTTGGATGGAGGCAACTTGTCCCAAATG	ACGTTGGATGTAAAACGTGGTCATGGGCTC	CAACTTGTCCCAAATGTGATCAT	T	G	appears somewhat unreliable; two control misscored; ke
TcQCR6p	9	1	This paper	9		27.9	217	ACGTTGGATGCTTCAAAACAGGTCTCCGTG	ACGTTGGATGTGCACCAAGTACAAGGACCG	GCAATCAGCGGGTCAAGTC	C	T	reliable
TccytMDH	10a	1	This paper	10		0	not in AP3	ACGTTGGATGTTTGCACCATGGGCAAGAAC	ACGTTGGATGAAACCCGCCCCTGAAGTCAT	CCCGCCCCTGAAGTCATCGGGGACGA	T	G	reliable
Tc06422_p285_rTyG	10b	1	Foley et al.	10	25.314	40.4	76	ACGTTGGATGGCCTCCGATTAGTTGTCTTC	ACGTTGGATGGGTCGAGTACAAGATTGGAG	CGATCTTGGGCGCCAAATAATATC	T	G	reliable
Tc30264_p415_rGyA	11	2	Foley et al.	11	14.902		118	ACGTTGGATGGGACCAACTACCAACTTCTG	ACGTTGGATGTTGGCAAATTGGCAAACCCC	TTCAATCCAAGAAGCTTCGCCA	G	A	appears unreliable; kept only for mapping
TcME1ad	12	2	This paper	12	0		613	ACGTTGGATGATGGCGCCCATCATCTACAC	ACGTTGGATGGGACAAAATAGCTCTTTGC	CTCTTTGCTTTGTGCC	C	A	mostly reliable; one control misscored

* Mapping postions obtained from DA F2 adult mapping; note that chromosomes may not be oriented in the same direction as those for Foley et al (2011) and will have different starting positions.
**Abberant postion in map further suggests genotyping errors (located 52.5 cm from Tc14140_p806_rAyG in this map).
