# Supplementary material for: Chromosome-Wide Impacts on the Expression of Incompatibilities in Hybrids of Tigriopus californicus
Source: G3 (Bethesda). 2016 Apr 11;6(6):1739–49. doi: 10.1534/g3.116.028050 (PMC4889669; doi:10.1534/g3.116.028050)
Supplement: Supplemental Material [file supp_g3.116.028050_TableS2.pdf]

Supplemental Table 2. PCR-based SNP scoring markers used to score F2 and backcross hybrids of *T. californicus.*

Locus	Foley et al./iPlex mar	Chromosome	AP3 Scaffold	primer 1 name	primer 1 seq	primer 2 name	primer 2 seq	primer 3 name	primer 3 seq	primer 4 nan	primer 4 seq	Tm for PCR	notes
c1_1718	NA	1	814	Ch1_1718_cons.f	CTACARTTTGTTCAGTGCACATG	Ch1_1718_cons.r	CCAATATTTCAGGATTATTGCCTCA	NA		NA		53˚	
c2_5	NA	2	5	c2_5_con.r	GCCACCGTGAGTTTTGAGTTC	c2_5_SD.f	ACTCTCAATGTATCAGCCTAATCC	c2_5_AB.f	GAACTACATTTGGAGGTACAGACA	NA		54˚	
3QCR8p	3b	3	9	3QCR8p_con.r	TTTGAACTTACGGGGCACCACG	3QCR8p_SD.f	TGAAATGAACCTAGCTTAGCTGCC	3QCR8p_AB.f	TTCTCATGGGGAGGCCCGAA	NA		52˚	
ME2	3d	3	15	MEprot2in_5.f	GTTCAACCCATTCCAACATGAGTTACA	ME2new.r	CTCAATGTTCGGAAACCTAGCT	ME2ab_3.r	TCTTTTTACCAACAGTCTTCATATCCTC	NA		54˚	developed in
3FBLRR	3d	3	15	3FBLRR_con.f	CCTGGCACGGTTCCCAATCTGTTC	3FBLRR_SDspec.r	AGCGGCCAATGTCTCTTATCATAATAAACC	3FBLRR_ABspec.	CAATGATCTTCCACGAGTGCGGGA	NA		54˚	alternative m
CYC1	4a	4	62	CYC1 ex3.con.f	GACTCGTTTGACCATGCTTCCATTCG	CYC1 ex4 con.r	CGGCATGGGTAGATAGTCGGTCAAT	NA		NA		58˚	developed in
P5CS	TcP5CS_p2737_rAyG	5	157	P5CexD.fwd	TTCGCCGCAAAAGATCCAAGC	TcP5Cex11.fwd	TCACGAGGATCTGCTCAAGAC	P5Cs-SD.rev	GAATCGGCGTACTGATACAAATCC	P5Cs-AB.rev	GCTTCTTTGACGATGTTCATCAACA	56˚	used 1.5x Mg
CYC	6a	6	262	Cyt 14.r	GGAATGTACTTTTTGGGGTTCG	ccSD.f	CCACAAAATGCCCCTCGCTCG	ccAB.F	CCGACAGACGGGCAAGGCCTCT	NA		58˚	developed in
c7_2276	NA	7	491	c7_2276_conr	CCCTGGCTGCTGAGCTGCTAG	c7_2276_SDf	TTAAGGAGATAATCTACGACGCTA	c7_2276_ABf	TAATTTGTTTTGTGCCGGCGATTT	NA		51˚	
GOT2	8d	8	554	GOT2_cons.fwd	GAGGCCTTCAAAAAGGATACTAATCC	GOT2_ab.rev	GAAACAATCTCATTTCCTGAAGATGA	GOT2sd3new.r	CGGAAAGCCTGCCAAACTCG	NA		54˚	variant of pri
c8_3336	NA	8	172	Ch8_3336_cons.r	CTCTGTGCCAATGTGGTCAGTTATT	Ch8_3336_SD.f	CAACTACTTGACGTTTCAAAAAAGGGA	Ch8_3336_AB.f	CGGTGATTGGAACACAAATATGAGC	NA		58˚	
c9_2203	NA	9	217	Ch9_2203_cons.r	AGGTTTCGGTGGAGGATCAA	Ch9_2203_SD.f	TAATCATCTACAAAACGCTCTGAAG	Ch9_2203_AB.f	TTGGCATGTTCTGTTTGATCAC	NA		54˚	
c10_1464	NA	10	24	Ch10_1464_cons.r	GGATGGAAAACATAAGCAGGTG	Ch10_1464_SD.f	CATCGTGCAATTTTTGGCAGGT	Ch10_1464_AB.f	CAGTTTGACATTGGTCACCCTT	NA		54˚	
11sep_tub	11	11	118	11sep_tub_con.f	CTCAGACAGCTCCTTGTGCT	11sep_tub_SD.r	ACAATCTCTGATCAACTAACATCG	11sep_tub_AB.r	CACATGTTGAAATCATCCGGATAA	NA		54˚	
ME1	12	12	613	MEpro1con.f	GGAATGTACTTTTCTCACGGAGATCG	ME1_SD.r	ATATCAAGAACCTGTGCCACAC	ME1_ab.r	CTAGAGCATCCTACGCATTCC	NA		54˚	developed in
