# Supplementary material for: Chromosome-Wide Impacts on the Expression of Incompatibilities in Hybrids of Tigriopus californicus
Source: G3 (Bethesda). 2016 Apr 11;6(6):1739–49. doi: 10.1534/g3.116.028050 (PMC4889669; doi:10.1534/g3.116.028050)
Supplement: Supplemental Material [file supp_g3.116.028050_TableS3.pdf]

Supplemental Table 3. Genotypic counts for iPlex loci scoring. Significance of deviations from Mendelian ratios and calculated relative viabilities are also given. For 1:2:1 deviations signficance threshhold was set at p<0.002 in paper which corresponds to a chi-square value>12.43.

Suspect Marker (included for mapping only)

DA adults

marker name	1	2a	2b	2c	3a	3b	3c	3d	3e	4a	4b	4c	5a	5b	6a	6b	7	8a	8b	8c	8d	9	10a	10b	12				11
Chromosome	1	2	2	2	3	3	3	3	3	4	4	4	5	5	6	6	7	8	8	8	8	9	10	11	12	3	3	9	11
pool	W1	W2	W2	W1	W2	W2	W1	W1	W2	W1	W1	W1	W1	W1	W1	W2	W2	W2	W1	W1	W1	W1	W1	W1	W2	W1	W2	W2	W2
	GDHad_1	QCR9p_2	mtMDH_2	P060_2	P102_3	QCR8p_3	I22708_3	ME2ad_3	I14140_3	CYC1_4	QCR10p_4	P169_4	GOT1Srg_5	I34449_5	CYCad_6	QCR7p_6	RPOL_7	RISP_8	GOT1p1_8	GOT1p2_8	GOT2_8	QCR6p_9	cytMDH_10	I06422_10	ME1ad_12	I30317_3	P125_3	I28594_9	I30264_11
B	168	154	174	163	203	200	205	208	227	141	149	156	132	122	172	176	152	146	149	142	143	167	80	65	148	269	170	254	32
S	69	118	103	98	11	4	22	25	46	118	103	103	151	145	126	115	108	135	118	179	120	107	167	209	134	41	232	68	146
SB	361	343	339	344	394	401	378	374	340	346	342	346	322	336	310	315	339	335	339	282	341	329	357	328	333	288	212	267	442
total	598	615	616	605	608	605	605	607	613	605	594	605	605	603	608	606	599	616	606	603	604	603	604	602	615	598	614	589	620
NA	26	9	8	19	16	19	19	17	11	19	30	19	19	21	16	18	25	8	18	21	20	21	20	22	9	26	10	35	4
%NA	4.17%	1.44%	1.28%	3.04%	2.56%	3.04%	3.04%	2.72%	1.76%	3.04%	4.81%	3.04%	3.04%	3.37%	2.56%	2.88%	4.01%	1.28%	2.88%	3.37%	3.21%	3.37%	3.21%	3.53%	1.44%	4.17%	1.60%	5.61%	0.64%
1:2:1 chisq	58.4916388	12.4113821	22.6071429	25.353719	174.552632	191.142149	148.395041	143.095552	114.210441	14.2595041	20.7609428	21.7966942	3.70743802	9.65008292	7.19736842	13.2310231	16.8831386	5.12662338	11.7260726	7.06301824	11.8245033	16.9568823	45.0960265	73.7342193	4.86666667	174.668896	71.3159609	122.609508	154.335484
AB rel viabilit	0.9281768	0.89534884	1.02352941	0.94492754	1.0278481	0.99502488	1.0817942	1.10933333	1.3313783	0.81268012	0.86880467	0.89913545	0.81733746	0.72403561	1.10610933	1.11392405	0.89411765	0.86904762	0.87647059	1.00353357	0.83625731	1.01212121	0.44692737	0.39513678	0.88622755	1.8615917	1.59624413	1.89552239	0.14446953
std dev rv	0.08076708	0.08044649	0.09038597	0.08396508	0.08414198	0.08117608	0.08967592	0.09209048	0.1129723	0.07404854	0.07861117	0.0803742	0.07710958	0.06854779	0.10089193	0.1006877	0.08082832	0.07944556	0.07952442	0.09747195	0.07632746	0.09089282	0.04627266	0.04423787	0.08096441	0.16543305	0.16782765	0.17465158	0.01998035
SD rel viab	0.38121547	0.68604651	0.60588235	0.56811594	0.0556962	0.0199005	0.11609499	0.13333333	0.26979472	0.68011527	0.60058309	0.59365994	0.93498452	0.86053413	0.81028939	0.7278481	0.63529412	0.80357143	0.69411765	1.26501767	0.70175439	0.64848485	0.93296089	1.27051672	0.80239521	0.28373702	2.17840376	0.50746269	0.65914221
std dev rv	0.04112562	0.0650423	0.05945614	0.05621931	0.0123801	0.00714928	0.01901419	0.02071765	0.03357041	0.06432375	0.05880101	0.05794839	0.08602984	0.07869536	0.07804136	0.07108725	0.0616495	0.07457558	0.06602403	0.11865046	0.06639555	0.06357577	0.08156224	0.11044035	0.07471121	0.03769955	0.22263158	0.05867137	0.05555006

DA adults temp

16˚C

marker name	1	2a	2b	2c	3a	3b	3c	3d	3e	4a	4b	4c	5a	5b	6a	6b	7	8a	8b	8c	8d	9	10a	10b	12				11
	GDHad_1	QCR9p_2	mtMDH_2	P060_2	P102_3	QCR8p_3	I22708_3	ME2ad_3	I14140_3	CYC1_4	QCR10p_4	P169_4	GOT1Srg_5	I34449_5	CYCad_6	QCR7p_6	RPOL_7	RISP_8	GOT1p1_8	GOT1p2_8	GOT2_8	QCR6p_9	cytMDH_10	I06422_10	ME1ad_12	I30317_3	P125_3	I28594_9	I30264_11
B	66	62	66	63	81	82	81	82	88	62	63	62	42	44	70	72	58	62	70	67	60	61	28	18	66	112	71	79	8
S	26	32	31	30	4	2	13	14	25	50	44	36	69	64	44	40	47	51	50	67	45	41	59	95	54	14	80	32	54
SB	141	143	142	142	152	152	140	139	125	123	122	135	123	126	122	125	130	126	115	100	129	133	147	120	117	106	85	122	177
total	233	237	239	235	237	236	234	235	238	235	229	233	234	234	236	237	235	239	235	234	234	235	234	233	237	232	236	233	239
1:2:1 chisq	24.0386266	17.7257384	18.7238494	19.4851064	68.9746835	73.8305085	48.5641026	47.2212766	33.9579832	1.74042553	4.13537118	11.6781116	6.84615385	4.8034188	6	9.35443038	3.6893617	1.71966527	3.5106383	4.94017094	4.38461539	7.49361702	23.5982906	51.1030043	1.25316456	84.5172414	19.1440678	19.4806867	73.041841
AB rel viabilit	0.92957747	0.86111111	0.92307692	0.88111888	1.05882353	1.07189543	1.14893617	1.17142857	1.3968254	1	1.02439024	0.91176471	0.67741936	0.69291339	1.13821138	1.14285714	0.88549618	0.97637795	1.20689655	1.32673267	0.92307692	0.91044776	0.37837838	0.29752066	1.11864407	2.09345794	1.65116279	1.28455285	0.08988764
std dev rv	0.12930635	0.12062157	0.12811027	0.12332655	0.13878833	0.14022844	0.1549442	0.15809106	0.19409648	0.14704292	0.15064027	0.13005969	0.1074802	0.1080811	0.16463095	0.1632135	0.12940173	0.14242954	0.17825066	0.20738625	0.13439549	0.13087654	0.06409329	0.06024671	0.16564503	0.30314338	0.27275269	0.18263296	0.02402403
SD rel viab	0.36619718	0.44444444	0.43356643	0.41958042	0.05228758	0.02614379	0.18439716	0.2	0.3968254	0.80645161	0.71544715	0.52941177	1.11290323	1.00787402	0.71544715	0.63492064	0.71755725	0.80314961	0.86206897	1.32673267	0.69230769	0.6119403	0.7972973	1.57024793	0.91525424	0.26168224	1.86046512	0.5203252	0.60674157
std dev rv	0.06397535	0.07279316	0.07176664	0.07011378	0.01926511	0.01337002	0.0411177	0.04338609	0.07184495	0.12333673	0.11261911	0.08509875	0.16086357	0.14623886	0.11261911	0.10141786	0.10935858	0.12146282	0.13459372	0.20738625	0.10674666	0.09559361	0.11181407	0.2196544	0.14011972	0.0589178	0.30374242	0.08837177	0.08234589

20˚C

marker name	1	2a	2b	2c	3a	3b	3c	3d	3e	4a	4b	4c	5a	5b	6a	6b	7	8a	8b	8c	8d	9	10a	10b	12				11
	GDHad_1	QCR9p_2	mtMDH_2	P060_2	P102_3	QCR8p_3	I22708_3	ME2ad_3	I14140_3	CYC1_4	QCR10p_4	P169_4	GOT1Srg_5	I34449_5	CYCad_6	QCR7p_6	RPOL_7	RISP_8	GOT1p1_8	GOT1p2_8	GOT2_8	QCR6p_9	cytMDH_10	I06422_10	ME1ad_12	I30317_3	P125_3	I28594_9	I30264_11
B	102	92	108	100	122	118	124	126	139	79	86	94	90	78	102	104	94	84	79	75	83	106	52	47	82	157	99	175	24
S	43	86	72	68	7	2	9	11	21	68	59	67	82	81	82	75	61	84	68	112	75	66	108	114	80	27	152	36	92
SB	220	200	197	202	242	249	238	235	215	223	220	211	199	210	188	190	209	209	224	182	212	196	210	208	216	182	127	145	265
total	365	378	377	370	371	369	371	372	375	370	365	372	371	369	372	369	364	377	371	369	370	368	370	369	378	366	378	356	381
1:2:1 chisq	34.4849315	1.47089947	7.64190981	8.65945946	105.71159	118.02981	101.010782	96.9193548	82.328	16.2648649	19.4054795	10.639785	2.30997305	7.09756098	2.19354839	4.88617886	13.9945055	4.45888594	16.6334232	7.48780488	8.22702703	10.2608696	23.7081081	30.3170732	7.73544974	92.3606557	55.5396825	120.780899	82.5433071
AB rel viabilit	0.92307692	0.91542289	1.09090909	0.98522168	1.00411523	0.944	1.0376569	1.06779661	1.28703704	0.70535714	0.77828054	0.88679245	0.9	0.73933649	1.07936508	1.08900524	0.8952381	0.8	0.70222222	0.81967213	0.77934272	1.07614213	0.492891	0.44976077	0.75576037	1.715847	1.546875	2.39726027	0.18045113
std dev rv	0.10296454	0.10724675	0.1250488	0.11338942	0.1052601	0.09859371	0.1090947	0.11246011	0.1379009	0.08241693	0.08968249	0.10173847	0.10602931	0.08812689	0.12685744	0.1271396	0.10301767	0.09405539	0.08194684	0.10276534	0.09145613	0.12393342	0.06475196	0.06088594	0.08841973	0.19321673	0.210708	0.29404339	0.02949707
SD rel viab	0.38914027	0.85572139	0.72727273	0.66995074	0.05761317	0.016	0.07531381	0.09322034	0.19444444	0.60714286	0.53393665	0.63207547	0.82	0.76777251	0.86772487	0.78534031	0.58095238	0.8	0.60444444	1.22404372	0.70422535	0.67005076	1.02369668	1.09090909	0.73732719	0.29508197	2.375	0.49315069	0.69172932
std dev rv	0.05343445	0.10150699	0.08981181	0.08318465	0.01608748	0.00811179	0.01877691	0.02128117	0.03428528	0.0734097	0.06710428	0.07782199	0.09831622	0.09080216	0.10588198	0.09719122	0.0733343	0.09405539	0.07299808	0.14350536	0.08441933	0.08445646	0.11481999	0.12170306	0.08670934	0.04866262	0.31138916	0.07793888	0.07446717

DA F (both temps)

marker name	1	2a	2b	2c	3a	3b	3c	3d	3e	4a	4b	4c	5a	5b	6a	6b	7	8a	8b	8c	8d	9	10a	10b	12				11
	GDHad_1	QCR9p_2	mtMDH_2	P060_2	P102_3	QCR8p_3	I22708_3	ME2ad_3	14140_3	CYC1_4	QCR10p_4	P169_4	GOT1Srg_5	I34449_5	CYCad_6	QCR7p_6	RPOL_7	RISP_8	GOT1p1_8	GOT1p2_8	GOT2_8	QCR6p_9	cytMDH_10	I06422_10	ME1ad_12	I30317_3	P125_3	I28594_9	I30264_11
B	140	138	158	147	178	175	181	183	200	127	134	140	114	108	161	165	132	133	134	127	128	144	61	41	132	233	147	223	21
S	64	98	84	79	9	4	20	23	41	106	91	89	130	125	104	94	92	115	103	158	102	98	157	197	110	36	200	61	129
SB	318	301	296	302	345	349	327	324	295	295	293	300	284	294	266	271	298	290	292	241	297	284	309	289	295	252	189	229	392
total	522	537	538	528	532	528	528	530	536	528	518	529	528	527	531	530	522	538	529	526	527	526	527	527	537	521	536	513	542
1:2:1 chisq	47.0268199	13.8268156	25.7769517	28.4545455	154.296993	165.496212	128.253788	122.875472	99.7723881	8.95075758	16.0656371	19.362949	4	8.15749526	12.2391714	19.2943396	16.6206897	4.48327138	9.35160681	7.33460076	11.0834915	11.3992395	50.6888046	97.2922201	7.03351955	149.533589	57.0559702	108.212476	151.092251
AB rel viabilit	0.87774295	0.91390729	1.06397306	0.97029703	1.02890173	1	1.10365854	1.12615385	1.35135135	0.85810811	0.91156463	0.93023256	0.8	0.73220339	1.20599251	1.21323529	0.88294314	0.91408935	0.91467577	1.04958678	0.8590604	1.01052632	0.39354839	0.28275862	0.89189189	1.84189723	1.54736842	1.93913044	0.10687023
std dev rv	0.08220289	0.08732522	0.09991747	0.09159271	0.08999059	0.08737041	0.09805297	0.10020504	0.12285676	0.08378876	0.08832198	0.08875219	0.08070045	0.07392677	0.11723638	0.11674999	0.08532364	0.08897876	0.08872051	0.10945903	0.08358164	0.09769261	0.04544857	0.03754879	0.08646463	0.17513389	0.17286974	0.19250994	0.01780533
SD rel viab	0.40125392	0.64900662	0.56565657	0.52145215	0.05202312	0.02285714	0.12195122	0.14153846	0.27702703	0.71621622	0.61904762	0.59136213	0.9122807	0.84745763	0.77902622	0.69117647	0.61538462	0.79037801	0.70307167	1.30578512	0.68456376	0.6877193	1.01290323	1.35862069	0.74324324	0.28458498	2.10526316	0.53043478	0.65648855
std dev rv	0.04541453	0.06650595	0.06040615	0.05628634	0.01275328	0.00823074	0.02103305	0.02305489	0.03666836	0.0725307	0.06500639	0.06204703	0.08976679	0.08308574	0.08163145	0.07359308	0.06416777	0.07909238	0.07185123	0.13190712	0.0697763	0.07161008	0.0938456	0.1247065	0.07467857	0.04038058	0.22844245	0.06545334	0.05880079

DA M (both temps)

marker name	1	2a	2b	2c	3a	3b	3c	3d	3e	4a	4b	4c	5a	5b	6a	6b	7	8a	8b	8c	8d	9	10a	10b	12				11
	GDHad_1	QCR9p_2	mtMDH_2	P060_2	P102_3	QCR8p_3	I22708_3	ME2ad_3	14140_3	CYC1_4	QCR10p_4	P169_4	GOT1Srg_5	I34449_5	CYCad_6	QCR7p_6	RPOL_7	RISP_8	GOT1p1_8	GOT1p2_8	GOT2_8	QCR6p_9	cytMDH_10	I06422_10	ME1ad_12	I30317_3	P125_3	I28594_9	I30264_11
B	28	16	16	16	25	25	24	25	27	14	15	16	18	14	11	11	20	13	15	15	15	23	19	24	16	36	23	31	11
S	5	20	19	19	2	0	2	2	5	12	12	14	21	20	22	21	16	20	15	21	18	9	10	12	24	5	32	7	17
SB	43	42	43	42	49	52	51	50	45	51	49	46	38	42	44	44	41	45	47	41	44	45	48	39	38	36	23	38	50
total	76	78	78	77	76	77	77	77	77	77	76	76	77	76	77	76	77	78	77	77	77	77	77	75	78	77	78	76	78
1:2:1 chisq	15.2368421	0.87179487	1.05128205	0.87012987	20.2894737	25.7012987	20.6883117	20.6103896	14.7662338	8.22077922	6.60526316	3.47368421	0.24675325	1.78947368	4.71428571	4.52631579	0.74025974	3.1025641	3.75324675	1.25974026	1.80519481	7.28571429	6.79220779	3.96	1.69230769	25.2857143	15.2051282	15.1578947	7.12820513
AB rel viabilit	1.27272727	0.74418605	0.72727273	0.74418605	1	0.94339623	0.92307692	0.98039216	1.17391304	0.53846154	0.6	0.68085106	0.92307692	0.65116279	0.48888889	0.48888889	0.95238095	0.56521739	0.625	0.71428571	0.66666667	1	0.7755102	1.2	0.82051282	1.94594595	1.91666667	1.58974359	0.43137255
std dev rv	0.3042887	0.19756935	0.19177128	0.19756935	0.23249528	0.21511122	0.21334623	0.22642297	0.27758365	0.14002556	0.15491933	0.17614059	0.24690028	0.17804946	0.14037379	0.14037379	0.24395234	0.15451421	0.16315393	0.19360884	0.17716467	0.24253563	0.1910203	0.30362885	0.22440863	0.48431053	0.59536209	0.39309341	0.12036882
SD rel viab	0.22727273	0.93023256	0.86363636	0.88372093	0.08	0	0.07692308	0.07843137	0.2173913	0.46153846	0.48	0.59574468	1.07692308	0.93023256	0.97777778	0.93333333	0.76190476	0.86956522	0.625	1	0.8	0.39130435	0.40816327	0.6	1.23076923	0.27027027	2.66666667	0.35897436	0.66666667
std dev rv	0.08444893	0.2364338	0.21995346	0.22673012	0.04277437	0	0.0410275	0.0418828	0.08027241	0.12509614	0.1312857	0.15900759	0.28059941	0.2364338	0.24072745	0.23166928	0.20370203	0.2162395	0.16315393	0.25400025	0.20446626	0.11843569	0.11812885	0.17354437	0.31430198	0.10313435	0.80737343	0.12137709	0.16625156

16˚C female

marker name	1	2a	2b	2c	3a	3b	3c	3d	3e	4a	4b	4c	5a	5b	6a	6b	7	8a	8b	8c	8d	9	10a	10b	12				11
	TcGDHad ger	TcQCR9p gen	TcmtMDH ge	P060_S200 g	P102_S185 g	TcQCR8p_2 g	Tc22708_p25	TcME2ad gen	Tc14140_p80	TcCYC1 genot	TcQCR10p ge	P169_S291 g	TcGOT1Srg ge	Tc34449_p23	TcCYCad gen	TcQCR7p gen	RPOL_864 ge	TcRISP_p167	TcGOT1p1 ge	TcGOT1p2 ge	TcGOT2_p39	TcQCR6p gen	TccytMDH ge	Tc06422_p28	TcME1ad genotype	Tc30317_p39	P125_S465 g	Tc28594_p74	Tc30264_p41
B	56	56	61	58	73	73	73	73	79	57	57	55	38	40	66	68	51	57	66	63	56	53	20	10	59	99	63	69	3
S	25	28	26	25	4	2	12	13	22	48	40	31	58	55	39	35	42	44	46	61	40	37	55	89	47	13	71	29	50
SB	127	128	127	127	135	136	124	124	112	105	107	122	113	114	106	109	117	113	98	85	113	120	134	109	106	95	77	111	161
total	208	212	214	210	212	211	209	210	213	210	204	208	209	209	211	212	210	214	210	209	209	210	209	208	212	207	211	209	214
1:2:1 chisq	19.4134615	16.5283019	18.9252336	19.5904762	60.7830189	65.4170616	42.8851675	41.1619048	31.0751174	0.77142857	3.32352941	11.7692308	5.21052632	3.88038278	6.91469194	10.4433962	3.51428571	2.25233645	4.74285714	7.31578947	3.83253589	6.72380952	28.3779904	60.4903846	1.35849057	72.8550725	16.0047393	16.1196172	75.1495327
AB rel viabilit	0.875	0.86821705	0.953125	0.90625	1.07352941	1.06569343	1.168	1.168	1.39823009	1.0754717	1.05555556	0.89430894	0.66666667	0.69565217	1.23364486	1.23636364	0.86440678	1	1.33333333	1.46511628	0.98245614	0.87603306	0.2962963	0.18181818	1.10280374	2.0625	1.61538462	1.23214286	0.03703704
std dev rv	0.12965216	0.1283313	0.13906763	0.1334191	0.14896543	0.14750572	0.16693507	0.16693507	0.20519345	0.16910592	0.16491032	0.1346505	0.11074258	0.11396526	0.18916683	0.18690844	0.13373501	0.153393	0.21038958	0.24526897	0.15115302	0.1334989	0.05686002	0.04619509	0.17191188	0.31577098	0.28093737	0.18467847	0.01558441
SD rel viab	0.390625	0.43410853	0.40625	0.390625	0.05882353	0.02919708	0.192	0.208	0.38938053	0.90566038	0.74074074	0.50406504	1.01754386	0.95652174	0.72897196	0.63636364	0.71186441	0.77192983	0.92929293	1.41860465	0.70175439	0.61157025	0.81481482	1.61818182	0.87850467	0.27083333	1.82051282	0.51785714	0.61728395
std dev rv	0.07049661	0.07565173	0.07246253	0.07049661	0.0217817	0.01497024	0.04478756	0.04706936	0.07489554	0.14661637	0.12356872	0.08633049	0.15563293	0.14719354	0.12259122	0.10878055	0.11454323	0.12424691	0.15498628	0.23840708	0.11525074	0.10058254	0.1191601	0.23669052	0.14234382	0.06363965	0.3128197	0.09231491	0.08747572

16˚C Male

marker name	1	2a	2b	2c	3a	3b	3c	3d	3e	4a	4b	4c	5a	5b	6a	6b	7	8a	8b	8c	8d	9	10a	10b	12				11
	TcGDHad ger	TcQCR9p gen	TcmtMDH ge	P060_S200 g	P102_S185 g	TcQCR8p_2 g	Tc22708_p25	TcME2ad gen	Tc14140_p80	TcCYC1 genot	TcQCR10p ge	P169_S291 g	TcGOT1Srg ge	Tc34449_p23	TcCYCad gen	TcQCR7p gen	RPOL_864 ge	TcRISP_p167	TcGOT1p1 ge	TcGOT1p2 ge	TcGOT2_p39	TcQCR6p gen	TccytMDH ge	Tc06422_p28	TcME1ad genotype	Tc30317_p39	P125_S465 g	Tc28594_p74	Tc30264_p41
B	10	6	5	5	8	9	8	9	9	5	6	7	4	4	4	4	7	5	4	4	4	8	8	8	7	13	8	10	5
S	1	4	5	5	0	0	1	1	3	2	4	5	11	9	5	5	5	7	4	6	5	4	4	6	7	1	9	3	4
SB	14	15	15	15	17	16	16	15	13	18	15	13	10	12	16	16	13	13	17	15	16	13	13	11	11	11	8	11	16
total	25	25	25	25	25	25	25	25	25	25	25	25	25	25	25	25	25	25	25	25	25	25	25	25	25	25	25	24	25

1:2:1 chisq	6.84	1.32	1	1	8.36	8.44	5.88	6.12	2.92	5.56	1.32	0.36	4.92	2.04	2.04	2.04	0.36	0.36	3.24	1.32	2.04	1.32	1.32	0.68	0.36	11.88	3.32	4.25	2.04
AB rel viabilit	1.33333333	0.75	0.625	0.625	0.88888889	1.05882353	0.94117647	1.125	1.28571429	0.52631579	0.75	1	0.72727273	0.61538462	0.47058824	0.47058824	1	0.71428571	0.44444444	0.5	0.47058824	1.14285714	1.14285714	1.33333333	1.16666667	2.16666667	1.77777778	1.66666667	0.58823529
std dev rv	0.54997194	0.33071891	0.28726213	0.28726213	0.35617231	0.42370239	0.38441022	0.460076	0.55256432	0.23088923	0.33071891	0.4472136	0.39368199	0.31680299	0.22557794	0.22557794	0.4472136	0.34149389	0.21013575	0.24333213	0.22557794	0.49989587	0.49989587	0.6181151	0.55160633	0.95146492	0.92592593	0.75124823	0.26581577
SD rel viab	0.13333333	0.5	0.625	0.625	0	0	0.11764706	0.125	0.42857143	0.21052632	0.5	0.71428571	2	1.38461539	0.58823529	0.58823529	0.71428571	1	0.44444444	0.75	0.58823529	0.57142857	0.57142857	1	1.16666667	0.16666667	2	0.5	0.47058824
std dev rv	0.10685169	0.24333213	0.28726213	0.28726213	0	0	0.09297598	0.09943689	0.23380488	0.12419738	0.24333213	0.34149389	0.9258201	0.61231271	0.26581577	0.26581577	0.34149389	0.4472136	0.21013575	0.33071891	0.26581577	0.28810524	0.28810524	0.48507125	0.55160633	0.13749299	1.02899151	0.28347336	0.22557794

20˚C Female

marker name	1	2a	2b	2c	3a	3b	3c	3d	3e	4a	4b	4c	5a	5b	6a	6b	7	8a	8b	8c	8d	9	10a	10b	12				11
	TcGDHad ger	TcQCR9p gen	TcmtMDH ge	P060_S200 g	P102_S185 g	TcQCR8p_2 g	Tc22708_p25	TcME2ad ger	Tc14140_p8(	TcCYC1 genot	TcQCR10p ge	P169_S291 g	TcGOT1Srg g(	Tc34449_p23	TcCYCad gen(	TcQCR7p gen	RPOL_864 ge	TcRISP_p167	TcGOT1p1 ge	TcGOT1p2 ge	TcGOT2_p39	TcQCR6p gen	TccytMDH ge	Tc06422_p28	TcME1ad genotype	Tc30317_p39	P125_S465 g	Tc28594_p74	Tc30264_p41
B	84	82	97	89	105	102	108	110	121	70	77	85	76	68	95	97	81	76	68	64	72	91	41	31	73	134	84	154	18
S	39	70	58	54	5	2	8	10	19	58	51	58	72	70	65	59	50	71	57	97	62	61	102	108	63	23	129	32	79
SB	191	173	169	175	210	213	203	200	183	190	186	178	171	180	160	162	181	177	194	156	184	164	175	180	189	157	112	118	231
total	314	325	324	318	320	317	319	320	323	318	314	321	319	318	320	318	312	324	319	317	318	316	318	319	325	314	325	304	328
1:2:1 chisq	27.6242038	2.24307692	9.99382716	10.9245283	93.75	100.570978	86.4231975	82.5	70.1455108	12.9937107	15.0191083	8.35825545	1.75862069	5.57232704	5.625	9.19496855	14.1730769	2.93209877	15.6833856	6.94952681	8.49056604	6.15189873	26.6226415	42.4420063	9.25846154	78.477707	43.8492308	113.131579	77.4329268
AB rel viabilit	0.875	0.94252874	1.14117647	1.01136364	0.99526066	0.95327103	1.05882353	1.09452736	1.31521739	0.73298429	0.82352941	0.94972067	0.88372093	0.75138122	1.18012422	1.19018405	0.89010989	0.85393258	0.6974359	0.81528662	0.77837838	1.1030303	0.46590909	0.34254144	0.76842105	1.69620253	1.48672566	2.58823529	0.15517241
std dev rv	0.10576426	0.11809858	0.14024521	0.12449225	0.11215349	0.10745223	0.12012979	0.12445676	0.15228382	0.09201429	0.10204094	0.11716354	0.11267451	0.09640177	0.14831156	0.14846969	0.1101687	0.10770738	0.08757797	0.11050681	0.09806173	0.13831496	0.06808176	0.05409324	0.09577114	0.20584716	0.21657846	0.35002442	0.02883729
SD rel viab	0.40625	0.8045977	0.68235294	0.61363636	0.04739337	0.01869159	0.07843137	0.09950249	0.20652174	0.60732984	0.54545455	0.64804469	0.8372093	0.77348066	0.80745342	0.72392638	0.54945055	0.79775281	0.58461539	1.23566879	0.67027027	0.73939394	1.15909091	1.19337017	0.6631579	0.29113924	2.28318584	0.53781513	0.68103448
std dev rv	0.05910107	0.10384047	0.09223775	0.08353019	0.01555068	0.00949832	0.020792	0.02393335	0.03857824	0.07954108	0.0741415	0.08636056	0.10783817	0.09864639	0.1082766	0.09866651	0.07556767	0.10196199	0.07647547	0.15624216	0.08718548	0.09971136	0.13966571	0.14118979	0.0853182	0.05192628	0.3195638	0.09208206	0.07879167

20˚C Male

marker name	1	2a	2b	2c	3a	3b	3c	3d	3e	4a	4b	4c	5a	5b	6a	6b	7	8a	8b	8c	8d	9	10a	10b	12				11
	TcGDHad ger	TcQCR9p gen	TcmtMDH ge	P060_S200 g	P102_S185 g	TcQCR8p_2 g	Tc22708_p25	TcME2ad ger	Tc14140_p8(	TcCYC1 genot	TcQCR10p ge	P169_S291 g	TcGOT1Srg g(	Tc34449_p23	TcCYCad gen(	TcQCR7p gen	RPOL_864 ge	TcRISP_p167	TcGOT1p1 ge	TcGOT1p2 ge	TcGOT2_p39	TcQCR6p gen	TccytMDH ge	Tc06422_p28	TcME1ad genotype	Tc30317_p39	P125_S465 g	Tc28594_p74	Tc30264_p41
B	18	10	11	11	17	16	16	16	18	9	9	9	14	10	7	7	13	8	11	11	11	15	11	16	9	23	15	21	6
S	4	16	14	14	2	0	1	1	2	10	8	9	10	11	17	16	11	13	11	15	13	5	6	6	17	4	23	4	13
SB	29	27	28	27	32	36	35	35	32	33	34	33	28	30	28	28	28	32	30	26	28	32	35	28	27	25	15	27	34
total	51	53	53	52	51	52	52	52	52	52	51	51	52	51	52	51	52	53	52	52	52	52	52	50	53	52	53	52	53
1:2:1 chisq	8.64705882	1.37735849	0.50943396	0.42307692	12.1372549	17.5384615	14.8846154	14.8846154	12.6153846	3.80769231	5.70588235	4.41176471	0.92307692	1.62745098	4.15384615	3.66666667	0.46153846	3.22641509	1.23076923	0.61538462	0.46153846	6.61538462	7.19230769	4.72	2.43396226	13.9615385	12.3962264	11.1923077	6.09433962
AB rel viabilit	1.2	0.71428571	0.75862069	0.78571429	1.03030303	0.86486487	0.88888889	0.88888889	1.09090909	0.52941177	0.51428571	0.52941177	0.96551724	0.64516129	0.48275862	0.48275862	0.89655172	0.48484849	0.70967742	0.81481482	0.75862069	0.90909091	0.61111111	1.10344828	0.64285714	1.76923077	1.875	1.5	0.34285714
std dev rv	0.3515316	0.23818715	0.2452744	0.25677486	0.29440509	0.24050263	0.24937049	0.24937049	0.30884774	0.17171039	0.16560591	0.17171039	0.29801096	0.20893569	0.17414117	0.17414117	0.28045323	0.16347644	0.22493278	0.26931523	0.2452744	0.26551028	0.18569776	0.33310526	0.21953602	0.53165606	0.71875	0.44194174	0.12432441
SD rel viab	0.26666667	1.14285714	0.96551724	1	0.12121212	0	0.05555556	0.05555556	0.12121212	0.58823529	0.45714286	0.52941177	0.68965517	0.70967742	1.17241379	1.10344828	0.75862069	0.78787879	0.70967742	1.11111111	0.89655172	0.3030303	0.33333333	0.4137931	1.21428571	0.30769231	2.875	0.28571429	0.74285714
std dev rv	0.11386487	0.34934535	0.29801096	0.31234752	0.06694549	0	0.04146614	0.04146614	0.06694549	0.18576169	0.15202096	0.17171039	0.22762622	0.22493278	0.35065235	0.33310526	0.2452744	0.23657101	0.22493278	0.34753449	0.28045323	0.1179225	0.12022262	0.15596891	0.36784746	0.13469916	1.06585673	0.12343252	0.21911167

AD adults

marker name	1	2a	2b	2c	3a	3b	3c	3d	3e	4a	4b	4c	5a	5b	6a	6b	7	8a	8b	8c	8d	9	10a	10b	12				11
	GDHad_1	QCR9p_2	mtMDH_2	P060_2	P102_3	QCR8p_3	l22708_3	ME2ad_3	14140_3	CYC1_4	QCR10p_4	P169_4	GOT1Srg_5	l34449_5	CYCad_6	QCR7p_6	RPOL_7	RISP_8	GOT1p1_8	GOT1p2_8	GOT2_8	QCR6p_9	cytMDH_10	l06422_10	ME1ad_12	l30317_3	P125_3	l28594_9	l30264_11
B	168	134	125	123	197	189	171	169	174	152	143	156	122	113	157	167	173	134	143	140	144	104	132	126	137	406	155	168	11
S	60	150	151	150	25	25	36	38	43	113	96	66	136	132	113	107	114	138	113	112	116	103	126	133	136	39	371	90	132
SB	333	276	288	284	347	351	347	351	344	295	307	342	300	314	297	297	277	292	304	305	299	350	307	300	288	97	27	302	430
total	561	560	564	557	569	565	554	558	561	560	546	564	558	559	567	571	564	564	560	557	559	557	565	559	561	542	553	560	573
NA	13	14	10	17	5	9	20	16	13	14	28	10	16	15	7	3	10	10	14	17	15	17	9	15	13	32	21	14	1
%NA	2.26%	2.44%	1.74%	2.96%	0.87%	1.57%	3.48%	2.79%	2.26%	2.44%	4.88%	1.74%	2.79%	2.61%	1.22%	0.52%	1.74%	1.74%	2.44%	2.96%	2.61%	2.96%	1.57%	2.61%	2.26%	5.57%	3.66%	2.44%	0.17%
1:2:1 chisq	61.2352941	1.02857143	2.65248227	2.83482944	131.446397	128.426549	101.173285	98.6702509	89.9304813	7.03928571	16.5604396	54.2553192	3.86379928	9.80858676	8.11463845	13.5359019	12.5212766	0.76595745	7.32857143	7.85816876	5.52593918	36.7163375	4.37699115	3.18246869	0.40463458	720.446495	619.01085	25.1857143	194.853403
AB rel viabilit	1.00598802	0.96750903	0.8650519	0.8631579	1.13218391	1.07386364	0.98275862	0.96022727	1.00869565	1.02702703	0.92857143	0.90962099	0.81063123	0.71746032	1.05369128	1.12080537	1.24460432	0.91467577	0.93770492	0.91503268	0.96	0.59259259	0.85714286	0.8372093	0.94809689	8.28571429	11.0714286	1.10891089	0.05104408
std dev rv	0.08988886	0.09557676	0.08535658	0.08580203	0.09727107	0.09248214	0.08636314	0.08423484	0.08863992	0.09716206	0.08760666	0.08161364	0.07935704	0.07039985	0.09893818	0.10423341	0.11804543	0.08872051	0.08874972	0.08683894	0.09124096	0.05753802	0.08206154	0.08144621	0.09201157	1.19178057	2.97731514	0.10243718	0.0113077
SD rel viab	0.35928144	1.08303249	1.0449827	1.05263158	0.14367816	0.14204546	0.20689655	0.21590909	0.24927536	0.76351351	0.62337662	0.38483965	0.90365449	0.83809524	0.75838926	0.71812081	0.82014389	0.94197952	0.74098361	0.73202614	0.77333333	0.58689459	0.81818182	0.88372093	0.94117647	0.79591837	26.5	0.59405941	0.612529
std dev rv	0.04109893	0.10502967	0.09977275	0.1010891	0.02247645	0.02219745	0.02805093	0.02864585	0.03170607	0.07628815	0.06384722	0.04253136	0.08666547	0.07967962	0.07562566	0.07243722	0.0833609	0.09089315	0.073389	0.07256876	0.07655079	0.05711879	0.07903479	0.08510012	0.09145714	0.13738882	7.09600797	0.06205495	0.05323567

16˚C

marker name	1	2a	2b	2c	3a	3b	3c	3d	3e	4a	4b	4c	5a	5b	6a	6b	7	8a	8b	8c	8d	9	10a	10b	12				11
	GDHad_1	QCR9p_2	mtMDH_2	P060_2	P102_3	QCR8p_3	l22708_3	ME2ad_3	14140_3	CYC1_4	QCR10p_4	P169_4	GOT1Srg_5	l34449_5	CYCad_6	QCR7p_6	RPOL_7	RISP_8	GOT1p1_8	GOT1p2_8	GOT2_8	QCR6p_9	cytMDH_10	l06422_10	ME1ad_12	l30317_3	P125_3	l28594_9	l30264_11
B	79	68	62	60	91	96	89	88	87	72	71	80	57	49	66	67	77	61	66	66	60	54	71	60	64	241	77	75	10
S	38	70	73	72	2	2	10	11	15	54	56	61	67	66	62	64	63	75	60	60	59	48	58	63	73	9	178	52	67
SB	154	136	140	139	182	178	170	173	171	146	139	128	147	155	144	145	136	137	145	145	151	169	142	148	139	17	15	147	198
total	271	274	275	271	275	276	269	272	273	272	266	269	271	270	272	276	276	273	271	271	270	271	271	271	276	267	270	274	275
1:2:1 chisq	17.4575646	0.04379562	0.97090909	1.24354244	86.4109091	87.2173913	65.1412639	63.7279412	55.4175824	3.85294118	2.23308271	3.31226766	2.6900369	8.06666667	1.05882353	0.77536232	1.47826087	1.43956044	1.59778598	1.59778598	3.8	16.8302583	1.87084871	2.37269373	0.60144928	606.505618	288.896296	5.32116788	76.8690909
AB rel viabilit	1.01935484	0.99270073	0.87943262	0.85714286	0.99453552	1.0726257	1.04093567	1.01149425	1.01162791	0.97959184	1.01428571	1.24031008	0.77027027	0.62820513	0.91034483	0.91780822	1.12408759	0.88405797	0.90410959	0.90410959	0.78947368	0.63529412	0.99300699	0.80536913	0.91428571	26.7777778	9.625	1.01351351	0.10050251
std dev rv	0.13356645	0.13900684	0.12400876	0.12188363	0.12038451	0.12967901	0.12938722	0.12522189	0.12596451	0.13269971	0.13998825	0.17299821	0.10877925	0.09035346	0.12577812	0.12618659	0.15430596	0.12589313	0.12464109	0.12464109	0.10906176	0.08728324	0.13608027	0.11233933	0.1284694	8.94900834	3.43665071	0.13604821	0.02419067
SD rel viab	0.49032258	1.02189781	1.03546099	1.02857143	0.02185792	0.02234637	0.11695906	0.12643678	0.17441861	0.73469388	0.8	0.94573643	0.90540541	0.84615385	0.85517241	0.87671233	0.91970803	1.08695652	0.82191781	0.82191781	0.77631579	0.56470588	0.81118881	0.84563758	1.04285714	1	22.25	0.7027027	0.67336683
std dev rv	0.07531312	0.14240634	0.14191878	0.1416336	0.01113746	0.01139109	0.028472	0.02952804	0.03596358	0.10513833	0.11529227	0.13763794	0.12393731	0.11423427	0.11953016	0.12154948	0.13050695	0.14943474	0.11536287	0.11536287	0.10800149	0.07982042	0.1153479	0.11684156	0.14327898	0.39223227	7.89422155	0.1011808	0.08435175

20˚C

marker name	1	2a	2b	2c	3a	3b	3c	3d	3e	4a	4b	4c	5a	5b	6a	6b	7	8a	8b	8c	8d	9	10a	10b	12				11
	GDHad_1	QCR9p_2	mtMDH_2	P060_2	P102_3	QCR8p_3	l22708_3	ME2ad_3	14140_3	CYC1_4	QCR10p_4	P169_4	GOT1Srg_5	l34449_5	CYCad_6	QCR7p_6	RPOL_7	RISP_8	GOT1p1_8	GOT1p2_8	GOT2_8	QCR6p_9	cytMDH_10	l06422_10	ME1ad_12	l30317_3	P125_3	l28594_9	l30264_11
B	89	66	63	63	106	93	82	81	87	80	72	76	65	64	91	100	96	73	77	74	84	50	61	66	73	165	78	93	1
S	22	80	78	78	23	23	26	27	28	59	40	5	69	66	51	43	51	63	53	52	57	55	68	70	63	30	193	38	65
SB	179	140	148	145	165	173	177	178	173	149	168	214	153	159	153	152	141	155	159	160	148	181	165	152	149	80	12	155	232
total	290	286	289	286	294	289	285	286	288	288	280	295	287	289	295	295	288	291	289	286	289	286	294	288	285	275	283	286	298
1:2:1 chisq	46.9034483	1.4965035	1.7266436	1.62937063	51.2721088	45.1522491	38.7122807	37.5244755	35.8541667	3.40972222	18.5142857	94.1389831	1.36933798	2.93771626	11.2576271	22.3016949	14.1875	1.92783505	6.89619377	7.42657343	5.21453287	20.3706294	4.7414966	1	1.29473684	180.636364	330.498233	23.1678322	119.959732
AB rel viabilit	0.98888889	0.93617021	0.84563758	0.8630137	1.27710843	1.06896552	0.92134832	0.90502793	1	1.06666667	0.85207101	0.70697674	0.84415584	0.8	1.18181818	1.30718954	1.35211268	0.93589744	0.9625	0.91925466	1.12751678	0.54945055	0.73493976	0.8627451	0.97333333	4.07407407	12	1.19230769	0.00858369
std dev rv	0.12081389	0.13052309	0.11684156	0.12000331	0.15631943	0.13115785	0.11459565	0.11261143	0.12403474	0.14104804	0.11035446	0.08428129	0.1147574	0.1078115	0.15184447	0.16616987	0.1776603	0.12403066	0.12532971	0.12029362	0.14831686	0.07556767	0.09893639	0.11718349	0.13066364	0.65431703	4.74692883	0.15200919	0.00612169
SD rel viab	0.24444444	1.13475177	1.04697987	1.06849315	0.27710843	0.26436782	0.29213483	0.30167598	0.32183908	0.78666667	0.47337278	0.04651163	0.8961039	0.825	0.66233766	0.5620915	0.71830986	0.80769231	0.6625	0.64596273	0.76510067	0.6043956	0.81927711	0.91503268	0.84	0.74074074	29.6923077	0.48717949	0.55793991
std dev rv	0.04339779	0.15331565	0.13932483	0.1431817	0.04903134	0.04643545	0.04901754	0.049934	0.05289321	0.10987711	0.07028823	0.01525116	0.12046609	0.11050924	0.09472048	0.0838673	0.10510098	0.11003206	0.09293658	0.09085646	0.10783304	0.08119254	0.10787885	0.12294702	0.11582159	0.14284692	11.6808538	0.07471869	0.06752525

AD F (both temps)

marker name	1	2a	2b	2c	3a	3b	3c	3d	3e	4a	4b	4c	5a	5b	6a	6b	7	8a	8b	8c	8d	9	10a	10b	12				11
	GDHad_1	QCR9p_2	mtMDH_2	P060_2	P102_3	QCR8p_3	l22708_3	ME2ad_3	14140_3	CYC1_4	QCR10p_4	P169_4	GOT1Srg_5	l34449_5	CYCad_6	QCR7p_6	RPOL_7	RISP_8	GOT1p1_8	GOT1p2_8	GOT2_8	QCR6p_9	cytMDH_10	l06422_10	ME1ad_12	l30317_3	P125_3	l28594_9	l30264_11
B	89	78	82	81	100	102	97	95	100	106	101	108	62	59	115	121	86	74	86	83	83	58	61	39	76	227	71	99	9
S	33	82	76	75	16	17	23	23	28	50	38	20	82	79	50	45	68	68	65	65	64	55	89	95	75	21	213	56	64
SB	197	156	162	158	205	201	193	197	189	162	171	193	171	179	156	157	165	177	167	167	169	203	169	184	167	57	25	160	252
total	319	316	320	314	321	320	313	315	317	318	310	321	315	317	321	323	319	319	318	315	316	316	319	318	318	305	309	315	325
1:2:1 chisq	37.2946709	0.15189873	0.275	0.24203822	68.6386293	66.16875	52.0159744	52.7269841	44.444795	19.836478	28.9096774	61.411215	4.85396825	7.82649842	26.576324	36.0154799	2.41065831	4.06583072	3.57861635	3.2031746	3.8164557	25.6898734	6.04702194	27.5849057	0.81132076	397.878689	347.601942	11.8190476	117.203077
AB rel viabilit	0.8989899	0.99363057	1.00613497	1.01886793	0.97087379	1.00990099	1	0.95959596	1.05263158	1.3006135	1.17441861	1.11340206	0.72093023	0.65555556	1.46496815	1.53164557	1.03614458	0.83146067	1.02380952	0.98809524	0.97647059	0.56862745	0.71764706	0.42162162	0.90476191	7.82758621	5.46153846	1.22981367	0.07114625
std dev rv	0.1064675	0.12991199	0.12882299	0.13181593	0.11119629	0.116036	0.11744404	0.11233609	0.12387974	0.16026932	0.14287958	0.12853603	0.09572644	0.08688805	0.18123445	0.18791422	0.13082235	0.10540995	0.12874162	0.1249875	0.12303245	0.07321726	0.09594569	0.06181488	0.11622638	1.4655368	1.5411958	0.15365205	0.01768498
SD rel viab	0.33333333	1.04458599	0.93251534	0.94339623	0.15533981	0.16831683	0.2371134	0.23232323	0.29473684	0.61349693	0.44186047	0.20618557	0.95348837	0.87777778	0.63694268	0.56962025	0.81927711	0.76404494	0.77380952	0.77380952	0.75294118	0.53921569	1.04705882	1.02702703	0.89285714	0.72413793	16.3846154	0.69565217	0.50592885
std dev rv	0.05075913	0.13545312	0.12096576	0.12366035	0.03063056	0.03246356	0.04098837	0.04004604	0.04771653	0.08679734	0.06629682	0.03752783	0.1199246	0.10952837	0.09102277	0.08335116	0.10787885	0.0985113	0.10244247	0.10244247	0.0996483	0.0703673	0.13040853	0.12298393	0.1149744	0.16612514	4.56136403	0.096229	0.06025674

AD M (both temps)

marker name	1	2a	2b	2c	3a	3b	3c	3d	3e	4a	4b	4c	5a	5b	6a	6b	7	8a	8b	8c	8d	9	10a	10b	12				11

	GDHad_1	QCR9p_2	mtMDH_2	P060_2	P102_3	QCR8p_3	l22708_3	ME2ad_3	14140_3	CYC1_4	QCR10p_4	P169_4	GOT1Srg_5	l34449_5	CYCad_6	QCR7p_6	RPOL_7	RISP_8	GOT1p1_8	GOT1p2_8	GOT2_8	QCR6p_9	cytMDH_10	l06422_10	ME1ad_12	l30317_3	P125_3	l28594_9	l30264_11
B	79	56	43	42	97	87	74	74	74	46	42	48	60	54	42	46	87	60	57	57	61	46	71	87	61	179	84	69	2
S	27	68	75	75	9	8	13	15	15	63	58	46	54	53	63	62	46	70	48	47	52	48	37	38	61	18	158	34	68
SB	136	120	126	126	142	150	154	154	155	133	136	149	129	135	141	140	112	115	137	138	130	147	138	116	121	40	2	142	178
total	242	244	244	243	248	245	241	243	244	242	236	243	243	242	246	248	245	245	242	242	243	241	246	241	243	237	244	245	248
1:2:1 chisq	26.0661157	1.24590164	8.65573771	9.2962963	67.6774194	63.2938776	49.5062241	46.037037	46.3852459	4.76859504	7.66101695	12.4814815	1.22222222	3.24793388	8.85365854	6.19354839	15.522449	1.73469388	4.90082645	5.60330579	1.85596708	11.6887967	13.0569106	20.2614108	0.00411523	322.746835	280.95082	16.2081633	82.1612903
AB rel viabilit	1.15328467	0.92561984	0.67716535	0.66141732	1.35664336	1.15231788	0.95483871	0.95483871	0.94871795	0.68656716	0.61313869	0.64	0.92307692	0.79411765	0.5915493	0.65248227	1.53982301	1.03448276	0.82608696	0.82014389	0.93129771	0.62162162	1.02158273	1.48717949	1	8.73170732	56	0.96503497	0.02234637
std dev rv	0.15770682	0.13964666	0.10616464	0.10424659	0.1775529	0.15007847	0.12650524	0.12650524	0.12542953	0.10445245	0.09467625	0.09347613	0.13439549	0.11629537	0.0904878	0.0978734	0.223415	0.15642156	0.11916082	0.11804115	0.13485803	0.09203096	0.14133677	0.21288291	0.14824986	1.94319582	45.9959553	0.13289253	0.01139109
SD rel viab	0.39416058	1.12396694	1.18110236	1.18110236	0.12587413	0.10596027	0.16774194	0.19354839	0.19230769	0.94029851	0.84671533	0.61333333	0.83076923	0.77941177	0.88732394	0.87943262	0.81415929	1.20689655	0.69565217	0.67625899	0.79389313	0.64864865	0.5323741	0.64957265	1	0.87804878	105.333333	0.47552448	0.75977654
std dev rv	0.0685576	0.16422739	0.16719442	0.16719442	0.03250645	0.02864063	0.03700918	0.04039164	0.04010165	0.13439199	0.12200224	0.09048052	0.12335259	0.11457266	0.12447223	0.12400876	0.13022716	0.17825066	0.10397932	0.10134721	0.11847914	0.09508578	0.08451898	0.10713342	0.14824986	0.23107459	86.2764667	0.07669667	0.09779866

16˚C Female

marker name	1	2a	2b	2c	3a	3b	3c	3d	3e	4a	4b	4c	5a	5b	6a	6b	7	8a	8b	8c	8d	9	10a	10b	12				11
	GDHad_1	QCR9p_2	mtMDH_2	P060_2	P102_3	QCR8p_3	l22708_3	ME2ad_3	14140_3	CYC1_4	QCR10p_4	P169_4	GOT1Srg_5	l34449_5	CYCad_6	QCR7p_6	RPOL_7	RISP_8	GOT1p1_8	GOT1p2_8	GOT2_8	QCR6p_9	cytMDH_10	l06422_10	ME1ad_12	l30317_3	P125_3	l28594_9	l30264_11
B	40	37	36	35	41	46	42	42	45	49	52	55	24	20	46	46	37	27	34	34	30	28	23	11	35	127	29	44	8
S	18	35	33	32	0	1	5	5	9	16	14	16	36	36	29	30	33	35	34	34	31	21	44	46	34	1	94	31	26
SB	82	69	73	72	100	95	92	93	86	75	72	67	79	83	65	66	72	79	72	72	78	91	72	83	73	10	14	65	107
total	140	141	142	139	141	142	139	140	140	140	138	138	139	139	140	142	142	141	140	140	139	140	139	140	142	138	137	140	141
1:2:1 chisq	11.0285714	0.12056738	0.23943662	0.30935252	48.5319149	44.7464789	34.2661871	34.6714286	25.8285714	16.2714286	21.1884058	22.1594203	4.66906475	8.92805755	4.84285714	4.30985916	0.25352113	2.95744681	0.11428571	0.11428571	2.09352518	13.3	6.52517986	22.3285714	0.12676056	330.985507	148.40146	3.12857143	42.3900709
AB rel viabilit	0.96385542	1.05714286	0.97297297	0.95890411	0.81188119	0.95833333	0.90322581	0.89361702	1.03448276	1.28947368	1.42465753	1.61764706	0.6	0.47619048	1.39393939	1.37313433	1.01369863	0.675	0.93150685	0.93150685	0.75949367	0.60869565	0.63013699	0.26190476	0.94594595	23.0909091	3.86666667	1.33333333	0.14814815
std dev rv	0.17455631	0.20543686	0.18640531	0.18544233	0.13748851	0.161449	0.15625578	0.15406393	0.18079233	0.23346999	0.25989313	0.30142069	0.12211724	0.10037236	0.26827093	0.2627664	0.19419393	0.13366379	0.18106577	0.18106577	0.14754956	0.11505368	0.13276383	0.06660922	0.18211766	9.89039009	1.4593711	0.25806726	0.04120948
SD rel viab	0.43373494	1	0.89189189	0.87671233	0	0.02083333	0.10752688	0.10638298	0.20689655	0.42105263	0.38356164	0.47058824	0.9	0.85714286	0.87878788	0.89552239	0.90410959	0.875	0.93150685	0.93150685	0.78481013	0.45652174	1.20547945	1.0952381	0.91891892	0.18181818	12.5333333	0.93939394	0.48148148
std dev rv	0.09442371	0.19611614	0.17353996	0.17230965	0	0.0150383	0.03687457	0.0364526	0.05632288	0.09666242	0.09239892	0.11073186	0.16808383	0.15761532	0.18165855	0.18307605	0.17668832	0.16426796	0.18106577	0.18106577	0.15144714	0.09299052	0.22482983	0.19306022	0.17782931	0.1519403	4.61026826	0.19184668	0.08913745

16˚C Male

marker name	1	2a	2b	2c	3a	3b	3c	3d	3e	4a	4b	4c	5a	5b	6a	6b	7	8a	8b	8c	8d	9	10a	10b	12				11
	GDHad_1	QCR9p_2	mtMDH_2	P060_2	P102_3	QCR8p_3	l22708_3	ME2ad_3	14140_3	CYC1_4	QCR10p_4	P169_4	GOT1Srg_5	l34449_5	CYCad_6	QCR7p_6	RPOL_7	RISP_8	GOT1p1_8	GOT1p2_8	GOT2_8	QCR6p_9	cytMDH_10	l06422_10	ME1ad_12	l30317_3	P125_3	l28594_9	l30264_11
B	39	31	26	25	50	50	47	46	42	23	19	25	33	29	20	21	40	34	32	32	30	26	48	49	29	114	48	31	2
S	20	35	40	40	2	1	5	6	6	38	42	45	31	30	33	34	30	40	26	26	28	27	14	17	39	8	84	21	41
SB	72	67	67	67	82	83	78	80	85	71	67	61	68	72	79	79	64	58	73	73	73	78	70	65	66	7	1	82	91
total	131	133	133	132	134	134	130	132	133	132	128	131	132	131	132	134	134	132	131	131	131	131	132	131	134	129	133	134	134
1:2:1 chisq	6.80152672	0.2481203	2.95488722	3.43939394	41.1044776	43.4776119	32.3384615	30.1818182	29.7819549	4.16666667	8.546875	6.72519084	0.18181818	1.30534351	7.68181818	6.82089552	1.76119403	2.48484849	2.26717557	2.26717557	1.77862595	4.78625954	18	15.6412214	1.52238806	276.72093	148.518797	8.20895522	39.8955224
AB rel viabilit	1.06849315	0.91176471	0.76470588	0.73529412	1.20481928	1.19047619	1.18987342	1.13580247	0.97674419	0.63888889	0.55882353	0.80645161	0.95652174	0.79452055	0.5	0.525	1.23076923	1.15254237	0.86486487	0.86486487	0.81081081	0.65822785	1.35211268	1.48484849	0.86567164	28.5	48	0.74698795	0.04347826
std dev rv	0.20294505	0.1844008	0.1600214	0.15513498	0.21064298	0.20723976	0.21365565	0.20278098	0.17335606	0.13511335	0.12565653	0.17493079	0.19040196	0.15916234	0.10660036	0.11049675	0.2426757	0.24092743	0.16924929	0.16924929	0.16066239	0.13192152	0.25178135	0.28358846	0.17809429	14.3169913	48.4974226	0.14203896	0.02256202
SD rel viab	0.54794521	1.02941177	1.17647059	1.17647059	0.04819277	0.02380952	0.12658228	0.14814815	0.13953488	1.05555556	1.23529412	1.4516129	0.89855073	0.82191781	0.825	0.85	0.92307692	1.3559322	0.7027027	0.7027027	0.75675676	0.6835443	0.3943662	0.51515152	1.1641791	2	84	0.5060241	0.89130435
std dev rv	0.11946213	0.20387532	0.22821882	0.22821882	0.02510686	0.01723673	0.04399571	0.04765372	0.04462193	0.20228166	0.23796145	0.28689592	0.1808739	0.16354712	0.15663212	0.16045083	0.19056875	0.27712055	0.14345214	0.14345214	0.15206494	0.13583548	0.0955404	0.12009271	0.22788118	1.09544512	84.4985207	0.10555989	0.15541304

20˚C Female

marker name	1	2a	2b	2c	3a	3b	3c	3d	3e	4a	4b	4c	5a	5b	6a	6b	7	8a	8b	8c	8d	9	10a	10b	12				11
	GDHad_1	QCR9p_2	mtMDH_2	P060_2	P102_3	QCR8p_3	l22708_3	ME2ad_3	14140_3	CYC1_4	QCR10p_4	P169_4	GOT1Srg_5	l34449_5	CYCad_6	QCR7p_6	RPOL_7	RISP_8	GOT1p1_8	GOT1p2_8	GOT2_8	QCR6p_9	cytMDH_10	l06422_10	ME1ad_12	l30317_3	P125_3	l28594_9	l30264_11
B	49	41	46	46	59	56	55	53	55	57	49	53	38	39	69	75	49	47	52	49	53	30	38	28	41	100	42	55	1
S	15	47	43	43	16	16	18	18	19	34	24	4	46	43	21	15	35	33	31	31	33	34	45	49	41	20	119	25	38
SB	115	87	89	86	105	106	101	104	103	87	99	126	92	96	91	91	93	98	95	95	91	112	97	101	94	47	11	95	145
total	179	175	178	175	180	178	174	175	177	178	172	183	176	178	181	181	177	178	178	175	177	176	180	178	176	167	172	175	184
1:2:1 chisq	27.4469274	0.41714286	0.1011236	0.15428571	25.5444444	24.4719101	20.2413793	20.2228571	19.3954802	6.03370787	11.1976744	52.2568306	1.09090909	1.28089888	25.4640884	39.7845304	2.67231638	4.02247191	5.76404494	4.98857143	4.66101695	13.2727273	1.63333333	8.19101124	0.81818182	108.556886	199.755814	11.5714286	75.9456522
AB rel viabilit	0.84482759	0.93181818	1.02222222	1.05747126	1.11320755	1.04672897	1.07843137	1.00952381	1.05769231	1.29545455	0.98	0.83464567	0.8172043	0.80412371	1.5	1.63043478	1.04255319	0.94949495	1.08333333	1.02083333	1.15217391	0.53097345	0.7755102	0.54901961	0.8631579	4.16666667	7	1.14583333	0.01369863
std dev rv	0.13240912	0.16482643	0.17596839	0.18415398	0.17410382	0.16452047	0.17281031	0.16113935	0.16835715	0.21772011	0.16111923	0.12527856	0.14407791	0.13923899	0.24206146	0.2606905	0.17501623	0.15775548	0.17884679	0.17014793	0.19251082	0.09361812	0.13457056	0.10105458	0.14897998	0.86985358	2.90737514	0.18754742	0.00981909
SD rel viab	0.25862069	1.06818182	0.95555556	0.98850575	0.30188679	0.29906542	0.35294118	0.34285714	0.36538462	0.77272727	0.48	0.06299213	0.98924731	0.88659794	0.45652174	0.32608696	0.74468085	0.66666667	0.64583333	0.64583333	0.7173913	0.60176991	0.91836735	0.96078431	0.8631579	0.83333333	19.8333333	0.52083333	0.52054795
std dev rv	0.05613773	0.18465408	0.16638385	0.17406906	0.06502833	0.06431825	0.07368118	0.07118795	0.0747223	0.14166493	0.09245487	0.02339923	0.16842311	0.15067296	0.09299052	0.07355053	0.13307489	0.11889606	0.11783134	0.11783134	0.13062806	0.10283452	0.15427509	0.15692681	0.14897998	0.20446238	8.13738646	0.10019369	0.08109703

20˚C Male

marker name	1	2a	2b	2c	3a	3b	3c	3d	3e	4a	4b	4c	5a	5b	6a	6b	7	8a	8b	8c	8d	9	10a	10b	12				11
	GDHad_1	QCR9p_2	mtMDH_2	P060_2	P102_3	QCR8p_3	l22708_3	ME2ad_3	14140_3	CYC1_4	QCR10p_4	P169_4	GOT1Srg_5	l34449_5	CYCad_6	QCR7p_6	RPOL_7	RISP_8	GOT1p1_8	GOT1p2_8	GOT2_8	QCR6p_9	cytMDH_10	l06422_10	ME1ad_12	l30317_3	P125_3	l28594_9	l30264_11
B	40	25	17	17	47	37	27	28	32	23	23	23	27	25	22	25	47	26	25	25	31	20	23	38	32	65	36	38	0
S	7	33	35	35	7	7	8	9	9	25	16	1	23	23	30	28	16	30	22	21	24	21	23	21	22	10	74	13	27
SB	64	53	59	59	60	67	76	74	70	62	69	88	61	63	62	61	48	57	64	65	57	69	68	51	55	33	1	60	87
total	111	111	111	111	114	111	111	111	111	110	108	112	111	111	114	114	111	113	111	111	112	110	114	110	109	108	111	111	114
1:2:1 chisq	22.2252252	1.37837838	6.27927928	6.27927928	28.3859649	20.981982	21.6486487	18.8378378	17.1081081	1.85454546	9.24074074	45.2142857	1.37837838	2.0990991	2	0.71929825	19.3423423	0.2920354	2.76576577	3.54054054	0.91071429	7.14545455	4.24561404	5.83636364	1.8440367	72.3518519	133.054054	11.990991	44.3684211
AB rel viabilit	1.23076923	0.92592593	0.56666667	0.56666667	1.54098361	1.08823529	0.7012987	0.74666667	0.90140845	0.73015873	0.65714286	0.51685393	0.87096774	0.78125	0.6984127	0.80645161	1.91836735	0.89655172	0.76923077	0.75757576	1.06896552	0.57142857	0.66666667	1.46153846	1.14285714	3.82352941	36	1.24590164	0
std dev rv	0.2426757	0.20983619	0.13528001	0.13528001	0.3049362	0.21361127	0.14038266	0.14944223	0.17874592	0.16035597	0.14005413	0.10350616	0.18613412	0.16783346	0.15486727	0.17493079	0.41470956	0.19711212	0.16448178	0.16125227	0.22803093	0.12591591	0.1426535	0.31543999	0.24560068	0.95276131	36.4965752	0.25323258	0
SD rel viab	0.21538462	1.22222222	1.16666667	1.16666667	0.2295082	0.20588235	0.20779221	0.24	0.25352113	0.79365079	0.45714286	0.02247191	0.74193548	0.71875	0.95238095	0.90322581	0.65306122	1.03448276	0.67692308	0.63636364	0.82758621	0.6	0.66666667	0.80769231	0.78571429	0.58823529	74	0.42622951	0.61363636
std dev rv	0.06694095	0.26491915	0.24138016	0.24138016	0.07196033	0.06360608	0.06007133	0.06667887	0.07101093	0.17131406	0.10686085	0.01624712	0.16371011	0.15712758	0.19864425	0.19173151	0.1669844	0.22184825	0.14877624	0.1407613	0.18473194	0.13063945	0.1426535	0.1914614	0.18038438	0.18576169	74.4983221	0.10898074	0.1183883

DA nauplii

marker name	1	2a	2b	2c	3a	3b	3c	3d	3e	4a	4b	4c	5a	5b	6a	6b	7	8a	8b	8c	8d	9	10a	10b	12				11
	GDHad_1	QCR9p_2	mtMDH_2	P060_2	P102_3	QCR8p_3	l22708_3	ME2ad_3	l14140_3	CYC1_4	QCR10p_4	P169_4	GOT1Srg_5	l34449_5	CYCad_6	QCR7p_6	RPOL_7	RISP_8	GOT1p1_8	GOT1p2_8	GOT2_8	QCR6p_9	cytMDH_10	l06422_10	ME1ad_12	l30317_3	P125_3	l28594_9	l30264_11
B	26	19	19	20	37	36	36	37	26	19	21	19	15	16	18	23	26	26	29	27	29	32	34	28	21	47	34	48	5
S	24	22	21	24	13	14	14	13	17	21	17	26	25	26	25	20	33	22	27	25	20	18	18	15	25	13	36	10	26
SB	40	46	47	44	40	38	37	36	47	47	48	49	48	47	48	44	29	38	34	34	36	38	39	43	41	26	20	28	64
total	90	87	87	88	90	88	87	86	90	87	86	94	88	89	91	87	88	86	90	86	85	88	91	86	87	86	90	86	95
NA	6	9	9	8	6	8	9	10	6	9	10	2	8	7	5	9	8	10	6	10	11	8	5	10	9	10	6	10	1
%NA	6.67%	10.34%	10.34%	9.09%	6.67%	9.09%	10.34%	11.63%	6.67%	10.34%	11.63%	2.13%	9.09%	7.87%	5.49%	10.34%	9.09%	11.63%	6.67%	11.63%	12.94%	9.09%	5.49%	11.63%	10.34%	11.63%	6.67%	11.63%	1.05%
1:2:1 chisq	1.2	0.49425287	0.65517241	0.36363636	13.9111111	12.6363636	13.0689655	15.6744186	1.97777778	0.65517241	1.53488372	1.21276596	3	2.52808989	1.35164835	0.21839081	11.3409091	1.53488372	5.46666667	3.86046512	3.89411765	6.09090909	7.48351648	3.93023256	0.65517241	40.3255814	27.8666667	44.0465116	20.7473684
AB rel viabilit	1.26829268	0.80851064	0.79166667	0.88888889	1.80487805	1.84615385	1.89473684	2	1.08333333	0.79166667	0.85714286	0.76	0.6122449	0.66666667	0.73469388	1.02222222	1.73333333	1.33333333	1.65714286	1.54285714	1.56756757	1.64102564	1.7	1.27272727	1	3.48148148	3.23809524	3.31034483	0.15384615
std dev rv	0.31443933	0.20170063	0.19622594	0.22260769	0.42943017	0.4495484	0.46636088	0.49656353	0.25379227	0.19622594	0.20698905	0.18606514	0.15893638	0.17144817	0.18302233	0.24978372	0.4852352	0.33678766	0.43094774	0.40440815	0.39872947	0.40437219	0.41202806	0.3042887	0.25400025	0.9786834	1.03781189	0.89958424	0.05448371
SD rel viab	1.17073171	0.93617021	0.875	1.06666667	0.63414634	0.71794872	0.73684211	0.7027027	0.70833333	0.875	0.69387755	1.04	1.02040816	1.08333333	1.02040816	0.88888889	2.2	1.12820513	1.54285714	1.42857143	1.08108108	0.92307692	0.9	0.68181818	1.19047619	0.96296296	3.42857143	0.68965517	0.8
std dev rv	0.29359243	0.22717811	0.21269185	0.25883937	0.1787507	0.20186231	0.2087673	0.2039044	0.17972209	0.21269185	0.17501157	0.2402239	0.23887167	0.25379227	0.23887167	0.22260769	0.60278922	0.29183132	0.40440815	0.3778991	0.28915062	0.24690028	0.23874673	0.1823473	0.29418934	0.30844987	1.09578725	0.22762622	0.16970563

DA and AD combined sex

F (combined all)

marker name	1	2a	2b	2c	3a	3b	3c	3d	3e	4a	4b	4c	5a	5b	6a	6b	7	8a	8b	8c	8d	9	10a	10b	12				11
	GDHad_1	QCR9p_2	mtMDH_2	P060_2	P102_3	QCR8p_3	l22708_3	ME2ad_3	14140_3	CYC1_4	QCR10p_4	P169_4	GOT1Srg_5	l34449_5	CYCad_6	QCR7p_6	RPOL_7	RISP_8	GOT1p1_8	GOT1p2_8	GOT2_8	QCR6p_9	cytMDH_10	l06422_10	ME1ad_12	l30317_3	P125_3	l28594_9	l30264_11
B	229	216	240	228	278	277	278	278	300	233	235	248	176	167	276	286	218	207	220	210	211	202	122	80	208	460	218	322	30
S	97	180	160	154	25	21	43	46	69	156	129	109	212	204	154	139	160	183	168	223	166	153	246	292	185	57	413	117	193
SB	515	457	458	460	550	550	520	521	484	457	464	493	455	473	422	428	463	467	459	408	466	487	478	473	462	309	214	389	644
total	841	853	858	842	853	848	841	845	853	846	828	850	843	844	852	853	841	857	847	841	843	842	846	845	855	826	845	828	867
1:2:1 chisq	83.9108205	7.40093787	18.8391608	20.2327791	221.602579	229.45283	178.419738	173.321894	140.61782	19.4822695	39.2173913	67.2211765	8.39976275	15.5710901	35.0140845	50.6764361	16.5909631	8.26254376	12.3364817	1.1450654	14.2004745	26.3966746	50.6524823	118.448521	6.80584795	445.619855	295.785799	104.528986	265.719723
AB rel viabilit	0.8875969	0.94323144	1.04575163	0.98915401	1.00907441	1.00544465	1.0671785	1.0651341	1.2371134	1.01746725	1.01075269	1.00404858	0.77192983	0.70464135	1.30496454	1.33333333	0.93965517	0.88461539	0.95652174	1.02689487	0.90364026	0.82786885	0.50939457	0.33755274	0.89848812	2.96774194	2.02790698	1.65128205	0.09302326
std dev rv	0.06519746	0.07274885	0.07918437	0.07542341	0.07013624	0.06992573	0.07559535	0.07540098	0.08886581	0.07747154	0.07646145	0.07376944	0.0619756	0.0565623	0.09966344	0.10083169	0.07205021	0.06827631	0.07343401	0.08262281	0.06954859	0.06336015	0.04397991	0.03304602	0.0695227	0.2464866	0.20745536	0.12777084	0.01284089

SD rel viab	0.37596899	0.7860262	0.69716776	0.6681128	0.0907441	0.07622505	0.16506718	0.17624521	0.28453608	0.68122271	0.55483871	0.44129555	0.92982456	0.86075949	0.72813239	0.64801865	0.68965517	0.78205128	0.73043478	1.09046455	0.71092077	0.62704918	1.02713988	1.23206751	0.79913607	0.36774194	3.84186047	0.6	0.59844961
std dev rv	0.03410429	0.06273886	0.05700319	0.05502577	0.01370245	0.01243466	0.01994928	0.02073791	0.02913751	0.05604535	0.04755995	0.0390229	0.07205356	0.06634937	0.06144279	0.055714	0.05621731	0.06181319	0.05906255	0.08690283	0.05738283	0.05092857	0.07635355	0.08957645	0.06322986	0.0433546	0.3790088	0.05509604	0.04274153

M (combined all)

	GDHad_1	QCR9p_2	mtMDH_2	P060_2	P102_3	QCR8p_3	l22708_3	ME2ad_3	14140_3	CYC1_4	QCR10p_4	P169_4	GOT1Srg_5	l34449_5	CYCad_6	QCR7p_6	RPOL_7	RISP_8	GOT1p1_8	GOT1p2_8	GOT2_8	QCR6p_9	cytMDH_10	l06422_10	ME1ad_12	l30317_3	P125_3	l28594_9	l30264_11
B	107	72	59	58	122	112	98	99	101	60	57	64	78	68	53	57	107	73	72	72	76	69	90	111	77	215	107	100	13
S	32	88	94	94	11	8	15	17	20	75	70	60	75	73	85	83	62	90	63	68	70	57	47	50	85	23	190	41	85
SB	179	162	169	168	191	202	205	204	200	184	185	195	167	177	185	184	153	160	184	179	174	192	186	155	159	76	25	180	228
total	318	322	322	320	324	322	318	320	321	319	312	319	320	318	323	324	322	323	319	319	320	318	323	316	321	314	322	321	326

M (combined all)																										l30317_3	P125_3	l28594_9	l30264_11
1:2:1 chisq	40.408805	1.60248447	8.40372671	8.9	86.4382716	88.0621118	69.9433962	66.225	60.3208723	8.93730408	11.8653846	15.9028213	0.66875	4.2327044	13.1795666	10.1481482	13.3726708	1.81733746	8.03448276	4.86833856	2.675	14.6037736	18.8823529	23.664557	0.42679128	318.382166	272.552795	26.4267913	83.6441718
AB rel viabilit	1.18888889	0.88343558	0.69411765	0.68639053	1.27083333	1.10344828	0.95145631	0.96585366	1.00497512	0.64864865	0.61290323	0.65306122	0.92857143	0.76404494	0.56989247	0.61621622	1.38961039	0.9068323	0.77837838	0.8	0.86857143	0.71502591	0.96256685	1.42307692	0.9625	5.58441558	8.23076923	1.10497238	0.11353712
std dev rv	0.1411274	0.11572583	0.0934744	0.0929372	0.14469629	0.12469327	0.10935331	0.11099043	0.11585196	0.08500373	0.08117019	0.08300725	0.11872997	0.0985113	0.07682063	0.08172548	0.17469761	0.1189592	0.09806173	0.10162034	0.11013934	0.08976589	0.11590052	0.17723136	0.12532971	0.91213602	2.30500112	0.13223399	0.02416936
SD rel viab	0.35555556	1.0797546	1.10588235	1.11242604	0.11458333	0.07881773	0.14563107	0.16585366	0.19900498	0.81081081	0.75268817	0.6122449	0.89285714	0.82022472	0.9139785	0.8972973	0.80519481	1.11801242	0.68108108	0.75555556	0.8	0.59067358	0.5026738	0.64102564	1.0625	0.5974026	14.6153846	0.45303867	0.74235808
std dev rv	0.05564547	0.13667989	0.13655597	0.1376469	0.02654591	0.0209002	0.02947545	0.0319382	0.03606132	0.10131715	0.09522306	0.07899935	0.1149744	0.10426089	0.11135845	0.10998867	0.11047279	0.14163866	0.08827538	0.09709555	0.1030677	0.07747386	0.06979589	0.0917648	0.13610169	0.12408816	4.07135132	0.0657883	0.08485393
