# Supplementary material for: Chromosome-Wide Impacts on the Expression of Incompatibilities in Hybrids of Tigriopus californicus
Source: G3 (Bethesda). 2016 Apr 11;6(6):1739–49. doi: 10.1534/g3.116.028050 (PMC4889669; doi:10.1534/g3.116.028050)
Supplement: Supplemental Material [file supp_g3.116.028050_TableS4.pdf]

Supplemental Table 4. Two-way interactions between iPlex markers. The table shows all combinations with a p<0.05 and a summary by chromosome of these interactions. Yellow highlights cells that exceed the Bonferroni corrected P-value<0.00018 which corresponds to a chi-square value of 22.3 (with 276 comparisons per cross and 4 d.f.), while green highlights values with P-value<0.001 (chi-square>18.47).
% Possible refers to the number of comparisons with p<0.05 compared to the total number of marker combinations for that pair of populations.

Female all (combined AD and DA)

		Two Locus combinations (first locus/second locus genotype)											Two Locus Totals		
first_locus	second_locus	AA AA	AA DD	AA AD	DD AA	DD DD	DD AD	AD AA	AD DD	AD AD	Total #	chi-square value	Chromosome combos	# p<0.05	% possible
P060_2	GOT2_8	64	56	105	43	34	75	103	76	279	835	15.3013883	1,10	2	100.0%
QCR10p_4	RISP_8	62	53	117	37	36	55	103	85	272	820	12.7486585	1,2	1	33.3%
mtMDH_2	GOT2_8	64	56	113	44	33	74	103	75	274	836	12.6100849	2,8	2	16.7%
RISP_8	cytMDH_10	36	57	110	36	52	92	48	134	270	835	12.0677621	3,4	1	
CYC1_4	RISP_8	63	48	119	27	47	81	112	85	256	838	12.0161608	3,6	2	20.0%
QCR6p_9	X06422_10	9	77	115	15	60	76	56	153	276	837	11.6961314	4,8	2	16.7%
GDHad_1	QCR9p_2	55	50	122	14	29	49	140	94	275	828	11.3439766	6,8	1	
X14140_3	CYCad_6	84	55	153	23	4	41	166	87	221	834	10.7848726	8,10	2	25.0%
QCR8p_3	QCR7p_6	92	40	141	13	0	8	175	95	274	838	10.4357171	9,10	1	
CYCad_6	GOT1p2_8	71	58	145	28	49	72	110	116	189	838	10.1604016			
GDHad_1	cytMDH_10	30	62	136	23	24	49	65	160	284	833	10.0244496			
GOT1p1_8	X06422_10	23	71	123	9	49	108	48	171	237	839	10.0135679			
GDHad_1	X06422_10	29	82	117	14	28	54	37	179	295	835	9.90707508			
X14140_3	P169_4	94	43	156	15	3	48	136	62	276	833	9.69024472			

Males all (combined AD and DA)

		Two Locus combi0tions (first locus/second locus genotype)											Two Locus Totals		
first_locus	second_locus	AA AA	AA DD	AA AD	DD AA	DD DD	DD AD	AD AA	AD DD	AD AD	Total #	chi-square value	Chromosome combos	# p<0.05	% possible
P060_2	QCR8p_3	33	1	24	15	4	75	64	3	99	318	28.6536367	1,10	1	
mtMDH_2	QCR8p_3	32	1	26	15	4	75	64	3	101	321	26.0715076	1,12	1	
P060_2	P102_3	34	0	24	22	6	66	64	5	98	319	21.3830431	2,10	1	
mtMDH_2	P102_3	33	0	26	22	6	65	63	5	100	320	18.8607867	2,3	13	86.7%
GDHad_1	cytMDH_10	16	13	78	11	8	13	60	25	94	318	18.6272588	2,6	1	
P060_2	X14140_3	25	1	31	17	10	67	59	9	99	318	15.7759226	3,10	1	
P060_2	X22708_3	26	1	28	17	7	70	55	7	106	317	15.5909734	3,12	2	40.0%
mtMDH_2	X22708_3	26	1	28	17	7	69	55	7	107	317	15.3272534	3,4	4	26.7%
QCR9p_2	QCR8p_3	34	2	36	18	4	66	60	2	99	321	15.1943387	3,8	2	10.0%
mtMDH_2	X14140_3	25	1	33	17	10	66	59	9	101	321	14.9103658	4,8	5	41.7%
mtMDH_2	ME2ad_3	26	1	29	17	7	69	56	7	106	318	14.9072617	8,10	1	
X22708_3	P169_4	24	29	45	3	2	10	37	29	137	316	14.8519027			
CYC1_4	RISP_8	11	26	22	23	20	32	37	42	104	317	14.6914382			
X22708_3	X06422_10	32	10	55	4	7	4	73	33	95	313	14.1387218			
P060_2	ME2ad_3	26	2	29	17	7	70	56	8	104	319	13.7865006			
P102_3	ME1ad_12	36	31	50	6	3	2	35	51	105	319	13.4597798			
P102_3	GOT1p2_8	22	19	77	6	3	2	44	46	99	318	13.3599001			
QCR10p_4	GOT2_8	10	21	26	24	13	33	40	36	109	312	13.2096243			
ME2ad_3	P169_4	23	29	47	3	2	11	38	29	135	317	13.1193262			
CYC1_4	GOT2_8	11	22	26	23	14	38	41	33	110	318	12.7758494			
QCR9p_2	ME2ad_3	29	4	36	17	7	64	53	5	103	318	12.0640366			
RISP_8	X06422_10	29	7	34	33	21	33	47	22	87	313	11.2609365			
X14140_3	P169_4	24	28	49	4	2	14	36	30	130	317	11.0540302			
P102_3	GOT1p1_8	22	20	76	6	3	2	44	40	105	318	10.840793			
QCR9p_2	X22708_3	28	4	36	17	6	64	53	5	103	316	10.621648			
CYC1_4	GOT1p1_8	11	20	28	19	10	46	42	32	110	318	10.1924814			
QCR9p_2	cytMDH_10	12	15	43	33	9	46	43	23	95	319	9.97779422			
P060_2	QCR7p_6	10	11	37	9	22	62	36	48	84	319	9.92597791			
GDHad_1	ME1ad_12	26	21	59	7	15	10	43	48	87	316	9.85581107			
QCR8p_3	P169_4	23	31	57	2	1	5	39	28	130	316	9.84428222			
QCR8p_3	ME1ad_12	35	29	47	4	1	3	38	55	108	320	9.79662181			

QCR10p_4	GOT1p1_8	10	19	28	21	10	39	40	34	111	312	9.71677386

DA all F2 adults

		Two Locus combi0tions (first locus/second locus genotype)											Two Locus Totals		
first_locus	second_locu	AA AA	AA DD	AA AD	DD AA	DD DD	DD AD	AD AA	AD DD	AD AD	Total #	chi-square value	Chromosome combos	# p<0.05	% possible
QCR10p_4	RISP_8	49	31	67	25	34	43	71	64	204	588	20.9292843	1,10	1	50.00%
CYC1_4	RISP_8	45	30	64	18	38	61	80	63	200	599	18.5960418	1,2	3	100.00%
l14140_3	P169_4	48	44	130	4	4	35	103	54	175	597	17.7714314	2,7	2	66.67%
GOT1Srg_5	CYCad_6	32	41	59	54	23	74	86	61	174	604	15.2354162	2,8	1	8.33%
GOT1Srg_5	QCR7p_6	31	37	59	54	23	73	85	53	174	589	14.2260248	3,4	1	6.67%
GDHad_1	QCR9p_2	37	39	92	10	19	38	101	53	202	591	12.9114053	4,7	1	33.33%
CYC1_4	RPOL_7	33	18	86	17	24	71	97	64	171	581	12.7657528	4,8	5	41.67%
QCR10p_4	GOT2_8	45	29	71	27	27	48	71	60	211	589	12.5760298	5,6	4	100.00%
mtMDH_2	RPOL_7	53	23	93	17	28	56	82	57	187	596	12.5031677	5,9	2	100.00%
P060_2	RPOL_7	50	23	85	16	27	52	82	56	191	582	12.4363563	6,10	1	25.00%
GDHad_1	P060_2	35	35	97	16	16	36	110	45	204	594	12.1142345	8,10	2	25.00%
l34449_5	CYCad_6	30	36	56	52	23	70	90	64	181	602	11.8412329			
RISP_8	cytMDH_10	24	42	78	26	35	70	30	88	205	598	11.830411			
GDHad_1	mtMDH_2	37	34	97	15	15	37	115	45	196	591	11.4806089			
P169_4	GOT2_8	46	32	70	22	27	53	74	61	211	596	11.4555486			
P169_4	RISP_8	47	34	73	20	31	51	76	67	199	598	10.8161916			
l34449_5	QCR7p_6	29	32	56	52	23	69	89	56	181	587	10.7570202			
QCR9p_2	GOT2_8	45	36	67	27	21	64	70	62	205	597	10.3700995			
l34449_5	QCR6p_9	41	18	63	49	20	76	76	68	187	598	10.0464981			
GOT1p2_8	cytMDH_10	16	46	79	28	58	93	34	63	181	598	10.0088016			
GOT1Srg_5	QCR6p_9	43	22	67	52	21	78	72	64	181	600	9.76687389			
GDHad_1	cytMDH_10	23	38	107	15	16	38	41	113	202	593	9.71616869			
CYCad_6	cytMDH_10	16	53	102	11	37	78	53	77	176	603	9.52818373			

DA 16˚

		Two Locus combi0tions (first locus/second locus genotype)											Two Locus Totals		
first_locus	second_locu	AA AA	AA DD	AA AD	DD AA	DD DD	DD AD	AD AA	AD DD	AD AD	Total #	chi-square value	Chromosome combos	# p<0.05	% possible
QCR10p_4	CYCad_6	9	21	33	16	5	23	42	17	63	229	16.625978	10,12	1	50.00%
CYC1_4	CYCad_6	9	18	35	23	7	20	38	18	67	235	16.3961471	2,6	2	33.33%
QCR10p_4	RISP_8	24	15	24	11	15	18	26	20	76	229	15.1560987	2,7	1	33.33%
CYC1_4	QCR7p_6	9	16	36	23	7	20	38	17	67	233	15.0883748	3,8	2	10.00%
P169_4	CYCad_6	11	18	33	8	4	24	50	21	64	233	13.609851	4,6	6	100.00%
QCR10p_4	QCR7p_6	10	18	34	16	5	23	42	16	63	227	12.5748873	4,8	2	16.67%
P169_4	QCR7p_6	11	16	34	8	4	24	50	20	64	231	12.1479839	5,9	2	100.00%
l34449_5	QCR6p_9	14	6	24	23	5	36	24	30	72	234	12.0367058	6,10	2	50.00%
GOT1Srg_5	l30264_11	1	5	36	3	9	57	4	38	81	234	11.6634189	8,10	1	12.50%
GOT1p2_8	cytMDH_10	6	22	38	11	21	35	10	16	73	232	11.1256257			
l22708_3	GOT1p1_8	33	19	29	4	3	6	32	27	80	233	10.789646			
P060_2	CYCad_6	14	18	31	5	6	19	51	20	71	235	10.5982359			
ME2ad_3	GOT1p1_8	34	18	30	4	4	6	32	28	78	234	10.4809722			
CYCad_6	cytMDH_10	4	13	53	4	15	25	20	31	69	234	10.0702507			
QCR9p_2	RPOL_7	22	9	30	4	4	22	31	34	77	233	10.0667152			
QCR7p_6	cytMDH_10	5	13	53	2	13	25	20	32	69	232	9.7613162			
l06422_10	ME1ad_12	6	2	10	19	20	55	41	31	47	231	9.71312407			
GOT1Srg_5	QCR6p_9	12	6	24	24	6	39	25	29	69	234	9.56643344			
mtMDH_2	CYCad_6	15	18	32	5	6	19	50	20	71	236	9.55638985			
CYC1_4	RISP_8	20	15	27	7	16	27	33	20	70	235	9.49617942			

DA 20˚

20 total

Two Locus combi0tions (first locus/second locus genotype)

Two Locus Totals

first_locus	second_locu	AA AA	AA DD	AA AD	DD AA	DD DD	DD AD	AD AA	AD DD	AD AD	Total #	chi-square value	Chromosome combos	# p<0.05	% possible
GDHad_1	QCR8p_3	27	0	74	9	2	27	80	0	132	351	19.7147092	1,2	3	100.00%
GDHad_1	P060_2	18	29	54	9	10	23	72	29	118	362	15.7926171	1,3	1	20.00%
GOT1Srg_5	CYCad_6	22	32	36	29	12	41	51	38	109	370	15.3219764	2,10	1	16.67%
GDHad_1	QCR9p_2	18	31	53	5	13	23	65	36	115	359	15.0385024	2,3	2	13.33%
GDHad_1	mtMDH_2	20	28	54	8	9	24	75	29	111	358	14.9285034	2,4	1	11.11%
ME2ad_3	P169_4	18	29	78	1	1	9	70	37	123	366	14.7128695	2,5	1	16.67%
l14140_3	P169_4	24	32	80	3	1	16	66	33	109	364	14.6915684	2,7	3	100.00%
QCR9p_2	P169_4	18	16	54	32	7	44	43	43	110	367	13.9073327	3,4	3	20.00%
RPOL_7	RISP_8	13	19	61	13	21	26	56	41	111	361	13.3424344	4,5	2	33.33%
GOT1Srg_5	QCR7p_6	21	28	36	29	13	40	50	32	108	357	13.2019942	4,8	6	50.00%
l22708_3	P169_4	18	29	76	1	1	7	70	37	126	365	12.8822043	5,6	3	75.00%
RPOL_7	GOT1p1_8	14	25	52	19	11	30	45	27	128	351	12.7988485	6,10	1	25.00%
QCR10p_4	GOT1Srg_5	20	22	44	21	18	20	48	41	129	363	12.6161553	6,7	1	50.00%
QCR6p_9	l06422_10	8	45	52	8	20	37	31	48	115	364	12.0810499	7,8	2	50.00%
P169_4	GOT2_8	30	19	38	11	17	38	42	39	130	364	11.9617593	9,10	2	100.00%
QCR9p_2	RPOL_7	26	16	46	16	23	45	51	22	116	361	11.8482632			
P060_2	GOT1Srg_5	26	32	42	14	16	38	50	33	117	368	11.3782053			
P169_4	GOT1Srg_5	20	21	48	25	18	24	45	42	122	365	11.3739883			
QCR10p_4	GOT2_8	28	17	39	14	16	28	41	41	138	362	11.274403			
l34449_5	CYCad_6	20	26	32	29	13	39	53	41	115	368	11.2333893			
CYCad_6	cytMDH_10	12	40	49	7	22	53	33	46	107	369	11.1940371			
P169_4	GOT1p1_8	30	15	44	11	15	41	38	38	134	366	10.9484319			
CYC1_4	RISP_8	25	15	37	11	22	34	47	43	130	364	10.5129971			
QCR6p_9	cytMDH_10	8	40	58	8	20	37	36	47	111	365	10.4440585			
P060_2	RPOL_7	30	14	52	12	19	35	50	26	113	351	10.09796			
ME2ad_3	P060_2	39	12	73	2	3	6	59	53	122	369	10.0536972			
mtMDH_2	ME2ad_3	41	2	62	12	3	51	72	6	116	365	9.8105691			
P060_2	cytMDH_10	17	39	43	9	19	40	26	50	124	367	9.72311642			
CYCad_6	RPOL_7	32	9	55	20	18	37	40	33	108	352	9.63249258			
P169_4	GOT1p2_8	28	22	37	10	22	35	37	68	104	363	9.59481032			
QCR10p_4	GOT1p1_8	28	13	44	13	13	33	38	41	141	364	9.55388956			
mtMDH_2	RPOL_7	32	14	58	13	20	38	49	27	110	361	9.5455326			

DA females

		Two Locus combi0tions (first locus/second locus genotype)											Two Locus Totals		
first_locus	second_locu	AA AA	AA DD	AA AD	DD AA	DD DD	DD AD	AD AA	AD DD	AD AD	Total #	chi-square value	Chromosome combos	# p<0.05	% possible
QCR10p_4	RISP_8	46	28	58	24	29	37	62	53	175	512	20.0676257	1,10	1	
l14140_3	P169_4	41	37	117	4	3	31	94	48	146	521	18.5953953	1,2	1	
CYC1_4	RISP_8	42	25	58	15	34	56	73	53	166	522	17.7855664	1,3	1	
GOT1Srg_5	QCR7p_6	29	30	51	52	19	58	78	43	153	513	14.4329758	2,7	1	
GOT1Srg_5	CYCad_6	30	33	51	52	19	59	79	51	153	527	14.344367	2,8	1	
P169_4	GOT2_8	43	28	61	20	24	44	64	50	186	520	13.7561009	3,4	3	20.0%
QCR10p_4	GOT2_8	42	24	64	25	24	41	61	50	182	513	13.2516282	4,10	1	
RISP_8	cytMDH_10	22	41	68	19	33	60	20	81	177	521	13.2239992	4,8	7	58.3%
QCR9p_2	GOT2_8	42	33	57	22	17	53	63	51	182	520	12.5383014	5,6	4	
QCR10p_4	l06422_10	17	40	77	2	42	46	22	109	158	513	12.024646	5,9	1	
l34449_5	QCR7p_6	28	27	49	49	19	56	82	44	158	512	11.3873652	7,8	1	
l34449_5	CYCad_6	29	30	49	49	19	57	83	52	158	526	11.3512809	8,10	2	25.0%
RPOL_7	RISP_8	26	26	79	27	28	36	77	58	162	519	11.1133684			
GDHad_1	QCR9p_2	32	31	77	10	17	35	90	44	179	515	11.0197432			
QCR9p_2	RPOL_7	44	21	68	16	24	54	70	47	173	517	10.955379			
ME2ad_3	P169_4	38	31	112	2	2	18	95	56	169	523	10.8322654			
l34449_5	QCR6p_9	37	17	54	43	18	64	64	62	163	522	10.6861058			
P169_4	RISP_8	44	31	63	19	26	43	67	56	173	522	10.5942597			

CYC1_4	GOT2_8	38	22	64	21	30	55	68	49	177	524	10.4142338
GDHad_1	QCR8p_3	38	1	101	20	2	36	115	1	194	508	10.3341917
GOT1p1_8	l06422_10	13	50	71	3	30	68	25	117	147	524	10.0147248
QCR10p_4	GOT1p1_8	44	25	64	27	21	43	63	53	176	516	9.75633188
GDHad_1	cytMDH_10	18	35	87	13	16	35	29	106	178	517	9.64776485
l22708_3	P169_4	39	31	110	2	1	16	94	56	172	521	9.6163518

DA males

		Two Locus combi0tions (first locus/second locus genotype)											Two Locus Totals		
first_locus	second_locu	AA AA	AA DD	AA AD	DD AA	DD DD	DD AD	AD AA	AD DD	AD AD	Total #	chi-square value	Chromosome combos	# p<0.05	% possible
P060_2	QCR6p_9	2	1	13	2	2	15	19	6	17	77	13.0971926	1,10	1	
l14140_3	cytMDH_10	6	7	14	1	2	2	12	1	32	77	11.9197401	2,3	4	26.7%
mtMDH_2	QCR6p_9	2	1	13	2	2	14	19	6	18	77	11.8845663	2,9	2	66.7%
P102_3	GOT1p2_8	5	4	16	2	0	0	8	17	24	76	11.2695666	3,10	1	
P060_2	ME2ad_3	10	0	6	2	1	16	13	1	28	77	11.1958772	3,8	2	10.0%
CYCad_6	GOT1p2_8	2	2	7	7	1	14	6	18	20	77	11.1707317	5,7	1	
QCR7p_6	GOT1p2_8	2	2	7	7	1	13	6	18	20	76	10.9746444	6,8	2	25.0%
mtMDH_2	ME2ad_3	10	0	6	2	1	15	13	1	29	77	10.9083333			
GOT1Srg_5	RPOL_7	9	1	8	6	3	12	5	11	21	76	10.5993258			
P060_2	l14140_3	9	0	7	2	3	14	16	2	24	77	10.5404159			
GDHad_1	l06422_10	7	9	11	2	1	2	15	2	26	75	10.4936891			
mtMDH_2	l14140_3	9	0	7	2	3	13	16	2	25	77	10.3587807			
l14140_3	GOT1p2_8	8	6	13	0	4	1	7	11	27	77	9.79420656			

DA nauplii

		Two Locus combi0tions (first locus/second locus genotype)											Two Locus Totals		
first_locus	second_locu	AA AA	AA DD	AA AD	DD AA	DD DD	DD AD	AD AA	AD DD	AD AD	Total #	chi-square value	Chromosome combos	# p<0.05	% possible
QCR10p_4	QCR6p_9	10	7	4	9	1	6	12	8	28	85	13.2471315	1,12	1	100.00%
l22708_3	P169_4	5	4	27	4	3	7	9	15	13	87	13.2266923	2,12	2	66.67%
QCR10p_4	RPOL_7	6	5	9	2	12	2	16	14	17	83	12.5090517	2,5	3	50.00%
ME2ad_3	P169_4	5	5	27	4	2	7	9	14	13	86	11.7204712	3,4	2	13.33%
mtMDH_2	l34449_5	3	5	11	8	5	7	3	14	29	85	11.6826508	3,9	1	20.00%
P060_2	l34449_5	3	6	11	9	6	8	3	13	28	87	11.6320851	4,7	1	33.33%
l14140_3	QCR6p_9	5	8	11	6	5	3	20	4	23	85	11.122383	4,8	1	8.33%
QCR9p_2	ME1ad_12	9	2	8	1	8	11	10	14	21	84	10.9885673	4,9	1	33.33%
QCR7p_6	RISP_8	7	3	9	9	0	10	8	19	17	82	10.2551467	5,8	1	12.50%
l34449_5	GOT1p2_8	5	4	6	13	3	8	9	18	20	86	10.2429868			
mtMDH_2	ME1ad_12	9	2	8	2	6	12	9	16	20	84	10.1135673			
P060_2	GOT1Srg_5	4	5	11	8	7	8	3	12	29	87	9.73577569			
GDHad_1	ME1ad_12	6	3	16	7	4	10	8	17	14	85	9.69186813			

AD all F2 adults

		Two Locus combi0tions (first locus/second locus genotype)											Two Locus Totals		
first_locus	second_locu	AA AA	AA DD	AA AD	DD AA	DD DD	DD AD	AD AA	AD DD	AD AD	Total #	chi-square value	Chromosome combos	# p<0.05	% possible
X14140_3	P169_4	70	27	75	15	1	27	69	38	234	556	35.6113317	1,10	1	50.00%
ME2ad_3	P169_4	66	27	76	8	2	27	82	37	232	557	24.3885367	2,10	1	16.67%
X14140_3	QCR10p_4	65	32	71	12	7	24	66	57	210	544	24.0771574	2,3	1	6.67%
l22708_3	P169_4	66	27	78	8	2	26	81	37	229	554	22.6710406	3,10	1	10.00%
GDHad_1	cytMDH_10	23	37	107	19	16	24	84	72	177	559	14.7826093	3,4	6	40.00%
QCR9p_2	cytMDH_10	19	33	79	48	26	74	59	66	151	555	13.8761505	3,7	1	20.00%
QCR10p_4	GOT2_8	29	35	80	38	16	42	75	61	172	548	12.9763009	3,8	2	10.00%
P102_3	GOT2_8	40	34	118	12	4	9	93	77	174	561	12.6112846	4,8	2	16.67%
ME2ad_3	QCR10p_4	59	32	78	7	6	24	78	58	204	546	11.6501197	5,12	1	50.00%

P060_2	QCR8p_3	54	6	63	38	8	104	96	10	179	558	11.4355942	6,7	2	100.00%
RISP_8	QCR6p_9	30	24	76	34	21	81	39	59	193	557	11.0250194	8,9	1	25.00%
I34449_5	ME1ad_12	24	32	55	43	34	55	69	67	177	556	10.8971845	9,10	0	
QCR7p_6	RPOL_7	55	39	67	22	22	63	97	53	148	566	10.6388861			
CYCad_6	RPOL_7	56	36	65	22	22	62	97	53	147	560	10.1217657			
QCR8p_3	RPOL_7	47	38	105	13	3	9	115	73	164	567	9.98832715			
QCR10p_4	RISP_8	26	43	74	32	20	44	70	69	169	547	9.83378937			
P102_3	GOT1p2_8	39	33	118	11	4	10	91	73	179	558	9.7712174			
I22708_3	I06422_10	40	38	93	8	16	12	77	79	191	554	9.70755134			
I22708_3	QCR10p_4	59	31	81	7	7	22	78	58	201	544	9.61919392			

AD 16˚

		Two Locus combi0tions (first locus/second locus genotype)											Two Locus Totals		
first_locus	second_locus	AA AA	AA DD	AA AD	DD AA	DD DD	DD AD	AD AA	AD DD	AD AD	Total #	chi-square value	Chromosome combos	# p<0.05	% possible
QCR7p_6	RISP_8	6	25	34	10	14	40	45	36	63	273	18.4952154	2,10	1	16.7%
X14140_3	P169_4	35	25	26	6	1	8	38	35	93	267	18.0888882	2,12	1	33.3%
CYCad_6	RISP_8	6	24	34	9	14	39	44	36	63	269	17.5484603	2,7	1	33.3%
QCR9p_2	ME1ad_12	13	16	39	20	28	22	31	29	76	274	14.240973	3,4	4	26.7%
X14140_3	QCR10p_4	30	22	34	7	1	7	33	33	97	264	13.7197064	3,7	1	20.0%
GOT1Srg_5	I06422_10	13	5	38	20	19	28	27	39	81	270	12.9437241	3,8	1	5.0%
QCR9p_2	cytMDH_10	11	14	40	28	10	30	31	34	71	269	12.7683446	4,8	5	41.7%
CYCad_6	I06422_10	10	19	37	7	18	37	43	26	74	271	12.6876852	5,10	2	50.0%
QCR7p_6	I06422_10	10	19	37	7	18	37	43	26	74	271	12.6876852	6,10	2	50.0%
P102_3	GOT2_8	14	15	61	2	0	0	44	43	90	269	12.4363327	6,8	2	25.0%
ME2ad_3	P169_4	32	25	30	1	1	8	47	35	90	269	11.7852637			
QCR8p_3	RPOL_7	19	19	58	2	0	0	56	44	78	276	10.9578767			
I22708_3	P169_4	32	25	31	1	1	8	47	35	87	267	10.6550455			
QCR9p_2	RPOL_7	17	11	40	28	16	26	32	36	68	274	10.2424323			
I34449_5	I06422_10	11	4	33	18	19	29	30	39	86	269	10.2254718			
QCR10p_4	GOT1p1_8	13	24	34	18	7	31	35	28	76	266	9.86259611			
QCR10p_4	GOT1p2_8	13	24	34	18	7	31	35	28	76	266	9.86259611			
QCR10p_4	GOT2_8	11	21	39	20	9	27	29	28	81	265	9.72950089			
CYC1_4	GOT1p1_8	18	24	29	15	8	31	33	28	85	271	9.60891853			
CYC1_4	GOT1p2_8	18	24	29	15	8	31	33	28	85	271	9.60891853			

AD 20˚

		Two Locus combi0tions (first locus/second locus genotype)											Two Locus Totals		
first_locus	second_locus	AA AA	AA DD	AA AD	DD AA	DD DD	DD AD	AD AA	AD DD	AD AD	Total #	chi-square value	Chromosome combos	# p<0.05	% possible
X14140_3	QCR10p_4	35	10	37	5	6	17	33	24	113	280	17.7828685	1,10	1	50.0%
X14140_3	P169_4	35	2	49	9	0	19	31	3	141	289	16.8957881	1,3	1	20.0%
I22708_3	P169_4	34	2	47	7	1	18	34	2	142	287	15.8629689	1,4	1	33.3%
ME2ad_3	P169_4	34	2	46	7	1	19	35	2	142	288	15.6957263	2,12	1	33.3%
QCR8p_3	ME1ad_12	35	21	37	7	3	13	31	39	102	288	14.7286816	2,3	1	6.7%
CYCad_6	GOT1p1_8	23	7	61	16	11	18	38	35	80	289	14.2892179	2,6	2	33.3%
GDHad_1	QCR10p_4	22	9	54	6	8	6	44	23	109	281	13.7449741	3,10	2	20.0%
P169_4	QCR6p_9	15	17	43	0	4	1	35	34	139	288	13.6710131	3,12	2	40.0%
I22708_3	QCR10p_4	33	8	41	5	6	15	35	26	111	280	13.5970598	3,4	8	53.3%
GDHad_1	cytMDH_10	11	17	60	4	10	7	41	40	99	289	13.3497589	3,8	2	10.0%
X14140_3	CYC1_4	36	14	35	7	7	14	37	38	99	287	13.0524942	4,10	2	33.3%
QCR7p_6	GOT1p2_8	23	7	63	13	11	19	38	33	80	287	12.3461864	4,8	1	8.3%
CYCad_6	GOT1p2_8	23	7	61	13	12	19	38	33	81	287	12.3259712	4,9	1	33.3%
I22708_3	I06422_10	21	15	47	6	13	7	39	42	96	286	12.2270681	6,10	2	50.0%
QCR10p_4	cytMDH_10	21	15	37	10	12	18	22	39	108	282	11.5519589	6,8	4	50.0%
CYCad_6	cytMDH_10	23	23	45	16	6	28	22	38	94	295	11.4841927	7,10	1	50.0%

QCR7p_6	GOT1p1_8	25	8	62	13	11	18	38	34	80	289	11.3695555
GDHad_1	QCR8p_3	37	3	49	5	4	11	49	16	114	288	11.1179732
QCR9p_2	ME1ad_12	25	14	26	15	14	51	33	35	73	286	11.081516
ME2ad_3	QCR10p_4	32	9	40	5	5	17	36	26	110	280	10.9507213
X14140_3	GOT1p2_8	26	14	44	10	9	9	39	27	108	286	10.6602179
P102_3	ME1ad_12	34	24	41	7	3	13	32	36	98	288	10.6005964
X14140_3	GOT1p1_8	27	14	44	10	9	9	39	28	107	287	10.5670797
RPOL_7	cytMDH_10	23	26	49	15	11	25	18	30	92	289	10.5281315
QCR10p_4	RISP_8	14	20	39	17	7	16	38	32	99	282	10.4977267
QCR7p_6	cytMDH_10	25	25	46	12	5	27	22	38	93	293	10.3870868
QCR8p_3	I06422_10	25	17	50	7	10	6	34	43	96	288	10.273117
P169_4	cytMDH_10	19	18	39	0	4	1	41	46	127	295	10.074095
I22708_3	CYC1_4	33	15	34	7	6	13	39	37	101	285	9.64888469
QCR9p_2	QCR8p_3	27	8	31	18	5	57	49	10	83	288	9.55903813
mtMDH_2	QCR7p_6	15	5	44	25	13	40	55	25	69	291	9.51724996
mtMDH_2	CYCad_6	13	6	44	25	13	40	53	25	69	288	9.49934227

AD females

		Two Locus combi0tions (first locus/second locus genotype)											Two Locus Totals		
first_locus	second_locu	AA AA	AA DD	AA AD	DD AA	DD DD	DD AD	AD AA	AD DD	AD AD	Total #	chi-square value	Chromosome combos	# p<0.05	% possible
X14140_3	P169_4	53	6	39	11	0	17	42	14	130	312	30.9294158	2,8	1	
X14140_3	QCR10p_4	49	8	38	9	4	15	42	26	115	306	23.4115335	3,4	8	53.3%
ME2ad_3	P169_4	49	5	40	5	1	17	54	14	128	313	19.2717801	3,6	1	
QCR7p_6	RPOL_7	36	30	51	2	11	32	48	27	82	319	17.1090067	3,7	1	
ME2ad_3	QCR10p_4	46	9	39	5	4	14	50	25	115	307	16.2157747	6,7	2	100.0%
CYCad_6	RPOL_7	36	28	50	2	11	31	48	27	81	314	16.158164	9,10	1	
I22708_3	P169_4	48	5	43	5	1	17	54	14	124	311	15.8094091			
I22708_3	QCR10p_4	46	9	41	5	4	14	50	25	112	306	14.5001075			
QCR8p_3	QCR10p_4	45	11	43	5	4	8	51	23	120	310	13.5861724			
QCR8p_3	RPOL_7	20	20	62	9	2	6	57	46	97	319	10.5187733			
QCR8p_3	QCR7p_6	37	13	52	12	0	5	69	32	100	320	10.0471123			
X14140_3	CYC1_4	44	10	45	10	5	13	51	35	101	314	9.88847012			
QCR6p_9	I06422_10	4	15	39	4	24	27	31	56	116	316	9.70989981			
P060_2	GOT2_8	21	22	37	23	19	33	39	23	95	312	9.54863071			

AD males

		Two Locus combi0tions (first locus/second locus genotype)											Two Locus Totals		
first_locus	second_locu	AA AA	AA DD	AA AD	DD AA	DD DD	DD AD	AD AA	AD DD	AD AD	Total #	chi-square value	Chromosome combos	# p<0.05	% possible
P060_2	QCR8p_3	24	1	17	13	4	58	50	3	72	242	20.8410717	1,10	1	
GDHad_1	cytMDH_10	11	10	58	9	8	10	48	18	70	242	19.2261722	1,3	1	
mtMDH_2	QCR8p_3	23	1	19	13	4	58	50	3	73	244	18.401355	2,10	1	
P060_2	P102_3	25	0	17	18	5	52	52	4	70	243	16.4792111	2,3	5	33.3%
QCR9p_2	cytMDH_10	9	13	32	30	6	32	30	18	72	242	14.9855782	2,6	3	50.0%
P102_3	ME1ad_12	32	21	39	5	2	2	24	38	80	243	14.5466164	2,8	4	33.3%
mtMDH_2	P102_3	24	0	19	18	5	52	51	4	71	244	14.2410923	3,10	2	20.0%
QCR9p_2	QCR8p_3	27	2	27	13	4	51	47	2	71	244	14.2273675	3,12	2	40.0%
I22708_3	I06422_10	23	7	43	4	6	3	58	25	69	238	13.6822648	3,4	2	13.3%
QCR10p_4	GOT2_8	7	16	19	22	10	26	30	26	80	236	13.64316	3,8	3	15.0%
QCR8p_3	GOT2_8	15	12	59	1	3	4	45	36	67	242	13.5261158	4,10	1	
QCR10p_4	GOT1p2_8	7	16	19	19	7	32	30	24	82	236	13.460074	4,8	11	91.7%
P169_4	GOT1p2_8	5	17	26	12	6	28	40	23	84	241	13.1407199			
QCR8p_3	ME1ad_12	31	20	35	4	1	3	26	40	83	243	13.0756077			
P060_2	GOT1p1_8	6	5	29	12	14	49	39	29	58	241	13.0314114			
QCR10p_4	GOT1p1_8	7	16	19	19	7	32	30	25	81	236	12.9371403			

P169_4	GOT1p1_8	5	17	26	12	6	28	40	24	83	241	12.6308373
mtMDH_2	GOT1p1_8	6	5	29	12	14	49	39	28	59	241	12.3728163
mtMDH_2	GOT1p2_8	6	5	29	12	14	49	39	28	59	241	12.3728163
P060_2	GOT1p2_8	6	5	29	12	14	49	39	28	59	241	12.3728163
CYC1_4	GOT1p1_8	8	17	20	16	8	39	33	22	78	241	12.1903183
CYC1_4	GOT1p2_8	8	17	20	16	8	39	33	22	78	241	12.1903183
l22708_3	P169_4	18	22	34	3	1	9	27	23	103	240	11.6471577
P169_4	GOT2_8	5	17	26	14	7	25	42	27	79	242	11.2601185
CYC1_4	GOT2_8	8	17	20	20	12	31	32	22	79	241	11.2348659
CYC1_4	RISP_8	8	21	16	20	16	27	30	32	70	240	11.0751096
P060_2	QCR7p_6	8	6	28	7	18	50	29	36	61	243	11.0442664
P169_4	RISP_8	10	22	16	14	11	20	35	35	78	241	10.6820506
ME2ad_3	P169_4	17	22	35	3	1	10	28	23	102	241	10.652116
QCR8p_3	GOT1p1_8	14	12	59	1	3	4	42	32	74	241	10.2524479
QCR8p_3	GOT1p2_8	14	12	59	1	3	4	42	32	74	241	10.2524479
QCR8p_3	l06422_10	26	9	50	4	3	1	56	26	64	239	10.2333788
mtMDH_2	CYCad_6	6	7	28	7	18	50	28	36	62	242	9.8583151
GDHad_1	l22708_3	22	4	53	14	3	10	38	6	91	241	9.74203572
P060_2	CYCad_6	7	7	28	7	18	50	28	36	62	243	9.57116427
CYC1_4	cytMDH_10	15	5	26	26	8	29	28	23	82	242	9.52526289
