# Supplementary material for: Chromosome-Wide Impacts on the Expression of Incompatibilities in Hybrids of Tigriopus californicus
Source: G3 (Bethesda). 2016 Apr 11;6(6):1739–49. doi: 10.1534/g3.116.028050 (PMC4889669; doi:10.1534/g3.116.028050)
Supplement: Supplemental Material [file supp_g3.116.028050_TableS5.pdf]

Supplemental Table 5. Three-way interactions between iPlex markers. The table shows all combinations with a p<0.05 and a summary by chromosome of these interactions. Green highlights the interactions that have P-value<0.001 (chi-square>32.9 with 12 d.f.). No values exceed the Bonferroni corrected P-valueof 0.00003 (chi-square value of 42.4 with 1757 comparisons).

All females

			Three Locus combinations (first/second/third locus genotype)																													Three Locus Totals	
first_locus	second_locus	third_locus	AA AA AA	AA AA DD	AA AA AD	AA DD AA	AA DD DD	AA DD AD	AA AD AA	AA AD DD	AA AD AD	DD AA AA	DD AA DD	DD AA AD	DD DD AA	DD DD DD	DD DD AD	DD AD AA	DD AD DD	DD AD AD	AD AA AA	AD AA DD	AD AA AD	AD DD AA	AD DD DD	AD DD AD	AD AD AA	AD AD DD	AD AD AD	Total #	chi-square value	Chromosome Combos	# p<0.05
CYC1_4	RPOL_7	cytMDH_10	16	10	31	3	9	28	14	38	78	5	16	8	8	10	13	8	28	55	11	47	68	13	28	47	37	56	129	814	36.0956	1,2,10	4
QCR10p_4	GOT1p1_8	X06422_10	7	26	34	2	15	29	22	30	69	0	14	27	0	8	19	6	29	25	16	31	61	6	24	56	20	106	137	819	32.5001	1,2,12	1
QCR9p_2	QCR10p_4	cytMDH_10	5	24	28	4	11	21	19	25	69	12	5	35	3	9	9	14	28	55	26	29	66	12	20	39	20	87	136	811	32.3842	1,2,5	1
CYCad_6	GOT1p1_8	ME1ad_12	18	9	43	10	8	24	31	43	84	11	7	12	6	11	18	28	17	36	23	37	54	27	12	50	47	37	128	831	31.8943	1,3,10	2
QCR10p_4	RISP_8	cytMDH_10	12	16	34	14	9	30	18	32	66	12	6	19	3	14	19	4	20	31	12	35	55	17	27	40	26	77	165	813	31.4982	1,3,5	4
QCR7p_6	GOT1p1_8	ME1ad_12	19	9	43	10	8	24	32	43	83	11	7	11	6	10	17	26	14	33	23	37	54	27	12	50	47	38	128	822	31.0132	1,3,6	4
CYCad_6	GOT1p2_8	ME1ad_12	18	9	43	14	10	32	27	41	75	11	7	10	9	12	26	25	16	30	23	35	52	31	18	67	42	32	112	827	30.9021	1,4,8	1
X14140_3	CYC1_4	QCR7p_6	17	12	53	13	5	28	55	31	72	9	3	8	7	1	2	7	1	30	37	24	62	37	15	47	93	43	110	822	30.8003	1,9,10	1
X14140_3	CYC1_4	CYCad_6	17	15	53	13	5	28	54	35	72	9	2	9	7	1	2	7	1	30	38	26	61	37	15	47	91	46	111	832	30.7199	2,3,12	5
QCR10p_4	GOT2_8	X06422_10	7	27	29	1	14	28	23	28	74	1	14	26	0	10	19	5	27	25	15	34	57	4	28	51	23	100	147	817	30.2338	2,3,5	2
CYC1_4	RISP_8	cytMDH_10	9	16	38	9	9	30	17	31	70	9	5	13	5	21	21	8	29	44	17	36	58	22	22	40	23	74	155	831	29.6024	2,3,8	2
QCR7p_6	GOT1p2_8	ME1ad_12	19	9	43	14	10	32	28	41	74	11	7	9	9	11	24	23	13	29	23	35	52	31	18	67	42	33	111	818	29.4928	2,3,9	2
P102_3	CYC1_4	RPOL_7	21	8	46	4	12	33	40	34	70	3	1	4	6	0	1	3	3	4	34	31	80	19	19	57	83	50	154	820	29.4555	2,4,10	5
QCR10p_4	RPOL_7	cytMDH_10	20	12	32	3	10	28	20	36	68	5	13	14	5	4	13	8	22	40	8	47	61	15	29	47	31	63	144	798	28.9367	2,4,5	2
X14140_3	CYCad_6	X06422_10	14	33	37	5	23	26	15	51	86	2	5	15	0	3	1	5	21	15	14	69	82	7	26	54	18	58	143	828	28.2558	2,4,8	2
X22708_3	QCR7p_6	RISP_8	20	15	46	10	16	17	43	23	81	8	0	11	0	0	2	5	8	9	35	43	97	17	17	56	61	54	129	823	28.1932	2,5,8	2
mtMDH_2	ME2ad_3	ME1ad_12	15	25	41	1	6	5	44	22	74	14	10	20	1	3	6	16	26	55	32	34	85	9	0	15	71	53	148	831	28.1079	2,7,10	1
GDHad_1	QCR8p_3	X06422_10	15	29	27	1	2	0	13	51	90	4	12	13	0	3	1	9	13	34	8	61	104	3	5	6	26	111	179	820	28.0598	2,7,8	1
QCR9p_2	RPOL_7	X06422_10	2	25	35	1	10	22	12	37	59	5	13	25	8	12	22	6	29	50	14	30	62	5	46	34	26	81	142	813	27.7728	2,8,10	2
X22708_3	GOT2_8	X06422_10	12	27	39	1	22	30	17	57	71	1	6	5	0	7	0	5	6	13	10	42	69	5	25	73	29	98	163	833	27.5049	2,8,12	2
X22708_3	CYCad_6	RISP_8	20	15	45	12	17	19	43	23	81	8	0	11	0	0	2	5	8	9	34	43	97	19	19	57	60	54	131	832	27.28	3,4,10	6
QCR10p_4	X34449_5	GOT2_8	10	5	25	10	12	42	43	26	57	8	8	18	15	7	6	18	15	33	20	18	52	26	23	58	60	44	159	818	27.034	3,4,6	18
X14140_3	QCR7p_6	X06422_10	14	33	38	5	21	22	15	51	86	2	5	15	0	3	2	5	21	14	14	70	82	7	26	49	18	58	143	819	26.9316	3,4,7	1
ME2ad_3	GOT2_8	X06422_10	12	27	39	1	21	30	16	57	72	1	6	5	0	8	1	5	6	13	10	42	69	5	25	72	30	97	163	833	26.7955	3,4,8	5
X34449_5	QCR7p_6	GOT1p1_8	14	5	36	3	7	23	18	18	38	22	9	43	7	6	14	23	33	44	37	29	81	18	20	35	72	37	131	823	26.7523	3,4,9	5
P102_3	CYC1_4	X06422_10	9	27	39	2	19	28	15	56	76	5	2	1	0	4	3	0	5	5	14	53	79	7	30	57	27	91	174	828	26.2634	3,5,6	4
P102_3	CYCad_6	QCR6p_9	26	12	52	16	11	20	31	28	75	3	6	4	2	0	0	0	0	9	38	29	101	21	15	57	61	51	157	825	26.0018	3,6,10	2
X34449_5	CYCad_6	GOT1p1_8	13	5	37	4	8	25	18	18	38	21	9	42	7	6	15	23	33	44	37	30	81	19	21	41	72	37	131	835	25.9357	3,6,8	6
P060_2	P102_3	GOT1Srg_5	14	17	46	0	1	6	26	48	66	9	15	18	4	0	2	24	26	51	39	35	73	1	2	8	54	63	172	820	25.8526	3,6,9	2
RPOL_7	X06422_10	ME1ad_12	5	1	15	20	11	37	23	32	68	5	3	6	11	15	42	24	20	34	13	11	20	23	37	86	75	45	133	815	25.8068	3,7,10	1
X34449_5	GOT1p1_8	cytMDH_10	5	11	19	5	6	19	15	37	47	3	19	30	5	10	33	17	35	50	25	36	66	10	33	45	33	58	160	832	25.7941	3,7,12	1
mtMDH_2	P102_3	GOT1Srg_5	14	18	50	0	1	6	27	48	69	9	15	18	4	0	2	24	26	51	39	35	72	1	3	8	53	64	169	826	25.7819	3,8,10	5
mtMDH_2	X22708_3	ME1ad_12	15	26	43	1	5	5	44	21	71	13	10	20	1	3	5	17	26	56	32	35	83	8	0	15	71	53	150	829	25.7138	3,8,9	1
X34449_5	GOT1p2_8	cytMDH_10	4	11	19	6	14	24	14	29	43	3	19	30	6	12	37	15	33	47	24	36	62	18	45	57	26	47	148	829	25.5783	4,5,10	2
CYC1_4	GOT2_8	X06422_10	9	29	28	0	18	23	20	34	68	1	9	25	3	12	25	5	34	41	13	36	60	3	24	55	26	93	142	836	25.5012	4,5,12	1
GDHad_1	P060_2	X06422_10	5	21	31	7	20	17	17	40	69	1	3	13	2	5	15	11	20	25	15	52	83	7	22	57	15	103	151	827	25.4917	4,5,6	1
QCR8p_3	QCR10p_4	X06422_10	12	28	49	3	19	21	12	52	71	4	2	0	0	3	2	0	5	5	14	40	81	3	27	44	30	105	174	806	25.4553	4,5,7	1
GDHad_1	QCR6p_9	X06422_10	7	21	26	4	22	15	18	39	73	1	9	19	3	5	14	10	14	20	1	46	70	8	33	47	28	99	176	828	25.3346	4,5,8	4
X22708_3	RISP_8	X06422_10	11	22	42	5	22	28	14	61	71	1	9	3	2	5	1	3	5	14	7	38	69	9	29	76	28	97	159	831	25.2486	4,6,8	2
GOT1p1_8	QCR6p_9	X06422_10	0	25	27	6	17	22	17	28	74	4	13	29	0	8	18	5	28	61	5	39	59	9	35	35	34	96	139	833	25.1847	4,7,10	3
RPOL_7	GOT1p2_8	cytMDH_10	12	24	23	6	25	32	15	24	53	8	10	23	2	11	26	14	26	39	11	32	64	22	32	53	24	58	141	810	25.0524	4,7,8	2
ME2ad_3	QCR7p_6	RISP_8	22	15	46	10	15	17	43	23	80	8	1	11	0	0	2	5	9	10	34	43	97	18	18	56	60	53	129	825	24.9776		
QCR9p_2	P169_4	cytMDH_10	7	22	28	3	13	16	18	27	74	13	10	36	0	7	5	16	26	58	24	32	66	9	18	37	26	89	143	823	24.9721		
P102_3	QCR7p_6	QCR6p_9	26	12	52	13	10	18	31	28	75	4	6	4	2	0	0	0	0	9	38	30	101	19	16	54	61	49	158	816	24.9439		
P169_4	QCR7p_6	GOT1p2_8	20	12	41	14	13	21	30	30	54	9	11	12	1	2	14	16	21	21	42	34	91	12	29	30	64	64	108	816	24.8204		
GOT1p2_8	cytMDH_10	ME1ad_12	7	6	18	16	18	32	30	26	55	8	4	18	10	6	53	35	30	54	11	16	28	29	26	54	55	47	133	825	24.6147		
CYC1_4	GOT1p1_8	X06422_10	8	28	34	2	17	24	19	37	62	0	10	29	2	11	22	7	34	40	15	31	60	5	21	62	22	100	134	836	24.5948		
X22708_3	CYC1_4	QCR6p_9	15	14	56	9	14	24	52	23	70	1	0	10	2	1	4	5	7	13	33	28	74	25	16	59	59	49	170	833	24.4292		
ME2ad_3	CYCad_6	RISP_8	21	15	45	13	16	19	43	23	80	8	1	11	0	0	2	5	9	10	33	43	97	20	20	57	59	53	131	834	24.4268		
GDHad_1	mtMDH_2	X06422_10	5	23	31	7	20	17	17	39	69	1	4	11	2	5	14	11	19	26	15	52	88	7	22	57	15	103	148	828	24.3399		
QCR10p_4	X34449_5	cytMDH_10	10	4	26	13	16	36	19	39	68	4	14	16	5	7	16	10	19	38	10	35	43	7	39	61	38	66	156	815	24.3346		
P060_2	X22708_3	ME1ad_12	14	23	42	1	5	5	43	20	71	13	10	20	1	3	5	17	25	57	33	35	82	8	0	14	72	55	151	825	24.3025		
X14140_3	QCR10p_4	CYCad_6	20	16	55	13	6	27	49	31	67	9	1	7	3	0	4	10	3	29	37	32	54	28	9	38	98	42	124	812	24.0657		
ME2ad_3	CYC1_4	QCR7p_6	16	10	55	16	7	24	50	25	68	6	0	5	6	1	1	8	1	18	41	30	63	35	13	51	98	49	128	825	23.8872		
QCR7p_6	RISP_8	ME1ad_12	15	9	39	12	22	25	36	31	88	10	9	10	5	9	19	30	14	33	23	29	57	17	15	54	57	44	125	837	23.8348		
QCR10p_4	RISP_8	X06422_10	4	26	32	8	12	33	19	32	66	3	12	22	1	17	17	2	22	31	12	31	60	6	26	51	24	103	143	815	23.8227		
CYCad_6	RISP_8	ME1ad_12	14	9	38	11	22	25	34	29	89	11	10	11	5	10	21	29	15	34	23	28	56	17	15	53	56	43	123	831	23.7751		
mtMDH_2	QCR10p_4	cytMDH_10	7	22	31	7	13	26	20	39	64	11	5	33	3	8	8	12	28	40	26	31	66	9	19	35	23	73	156	815	23.7487		
X34449_5	RISP_8	ME1ad_12	7	8	23	6	9	22	24	19	44	14	8	29	7	20	17	34	24	49	27	31	53	20	18	60	62	42	151	828	23.5274		
X14140_3	P169_4	CYCad_6	23	17	52	11	6	26	50	32	73	8	1	6	2	0	1	12	3	33	42	33	58	19	11	31	104	42	129	825	23.4829		
ME2ad_3	QCR10p_4	QCR7p_6	20	11	55	13	6	22	47	23	69	6	0	2	4	0	3	10	2	19	41	35	59	27	8	44	103	46	134	809	23.4331		
X14140_3	GOT1Srg_5	CYCad_6	13	18	36	30	7	34	41	29	82	1	0	6	7	2	9	15	2	25	42	21	36	43	19	59	81	47	122	827	23.4245		
P060_2	QCR10p_4	cytMDH_10	7	21	33	7	13	24	20	39	58	11	6	32	3	8	8	12	29	40	26	29	65	9	19	38	23	72	162	814	23.4018		
P060_2	RISP_8	ME1ad_12	15	12	24	8	9	39	35	27	56	10	9	20	8	11	21	13	19	41	24	26	60	17	27	39	72	38	147	827	23.3767		
QCR7p_6	GOT2_8	ME1ad_12	20	11	43	11	17	24	30	32	83	11	6	10	7	8	16	24	17	35	16	33	58	21	11	47	60	42	129	822	23.3292		
QCR8p_3	P169_4	X06422_10	10	30	47	1	16	18	16	55	78	4	2	0	0	1	0	0	7	7	16	44	86	4	28	36	28	103	182	819	23.3282		
ME2ad_3	CYC1_4	CYCad_6	16	13	55	16	7	24	49	28	68	6	0	5	6	1	1	8	1	18	43	31	63	36	13	51	96	53	129	837	23.3115		
P060_2	GOT2_8	cytMDH_10	14	25	25	10	13	32	10	35	58	8	10	25	2	13	18	15	21	39	15	28	60	12	20	44	32	77	168	829	23.2362		
P060_2	QCR8p_3	GOT2_8	18	17	44	2	2	3	44	36	55	19	11	16	2	0	3	22	22	54	31	23	89	4	0	4	67	50	181	819	23.1778		
P169_4	GOT1Srg_5	RPOL_7	19	2	23	15	12	34	32	27	73	10	2	15	10	11	9	12	9	27	20	18	60	34	20	61	62	57	136	810	23.1397		
X14140_3	GOT1Srg_5	QCR7p_6	13	16	36	31	7	34	41	25	82	1	0	6	7	3	8	15	2	25	42	21	36	43	18	59	82	43	122	818	23.0359		
CYCad_6	GOT2_8	ME1ad_12	18	11	43	11	17	24	30	32	84	11	7	11	7	9	17	26	19	37	16	33	58	21	11	47	60	41	130	831	23.0184		
ME2ad_3	P169_4	CYCad_6	21	15	51	12	6	18	48	27	76	5	0	2	3	0	0	11	2	22	48	38	63	18	11	40	108	48	140	833	23.0038		
P102_3	RPOL_7	ME1ad_12	13	12	40	19	12	23	34	43	75	1	0	11	1	1	2	2	2	5	36	32	71	20	25	56	78	53	166	833	22.9956		
QCR10p_4	RPOL_7	QCR6p_9	14	16	34	5	7	29	39	16	69	5	11	16	7	3	12	14	14	42	27	15	74	31	15	45	54	48	138	800	22.9799		
QCR9p_2	QCR10p_4	GOT2_8	20	9	28	14	11	10	29	24	59	11	11	29	12	1	8	25	20	53	32	22	66	15	18	38	51	39	157	812	22.9774		
GDHad_1	X22708_3	GOT1Srg_5	12	13	56	2	1	5	28	37	74	9	8	10	0	0	8	15	12	33	37	51	81	5	7	14	66	79	165	828	22.9494		
GDHad_1	X14140_3	GOT1Srg_5	13	13	58	1	2	9	28	36	66	11	10	11	0	1	7	12	9	31	43	49	83	6	14	26	59	74	150	822	22.9478		
P060_2	ME2ad_3	ME1ad_12	14	22	40	1	5	5	43	21	74	14	10	20	1	3	6	16	25	56	33	34	84	9	1	14	72	55	149	827	22.8683		
GDHad_1	mtMDH_2	ME1ad_12	16	18	25	10	8	26	39	21	64	2	2	12	4	10	7	11	9	36	42	33	81	17	22	47	62	58	145	827	22.8611		
CYCad_6	RPOL_7	cytMDH_10	10	32	34	13	7	29	18	37	84	3	11	13	4	13	15	10	21	51	20	30	61	7	26	44	31	64	127	815	22.844		
QCR10p_4	GOT1p2_8	X06422_10	7	23	33	6	17	34	18	30	64	0	14	25	0	13	22	6	24	24	16	31	60	10	38	73	16	91	119	814	22.8203		
GDHad_1	P102_3	X06422_10	14	29	31	1	2	2	14	50	84	4	13	11	0	2	0	9	13	37	8	60	100	4	7	7	25	110	185	822	22.7022		
GDHad_1	QCR9p_2	X34449_5	9	10	36	6	8	36	22	32	67	5	1	7	5	10	14	11	9	29	22	39	78	20	31	42	62	61	150	822	22.6612		
ME2ad_3	QCR10p_4	CYCad_6	19	15	55	13	6	22	46	26	69	6	0	2	4	0	3	10	2	19	42	37	59	28	9	44	102	48	135	821	22.6494		
mtMDH_2	QCR10p_4	X34449_5	5	14	41	13	12	21	21	34	70	5	18	25	5	3	11	23	17	40	29	33	60	15	13	35	46	56	150	815	22.6254		
QCR8p_3	CYC1_4	X06422_10	10	30	42	2	20	27	15	52	75	4	2	0	0	1	2	0	7	5	14	50	77	7	32	59	27	94	171	825	22.6198		
ME2ad_3	CYC1_4	QCR6p_9	15	15	54	9	14	24	52	23	70	1	0	10	2	2	4	6	7	14	33	27	76	25	16	59	58	49	170	835	22.6064		
ME2ad_3	QCR10p_4	GOT2_8	27	16	44	9	11	20	42	22	78	3	0	5	5	2	0	4	8	19	33	27	76	27	17	37	60	55	171	818	22.591		
X14140_3	QCR10p_4	QCR7p_6	20	14	55	13	6	27	50	27	67	9	2	6	3	0	4	10	3	29	37	29	55	28	8	38	99	41	123	803	22.5715		
X14140_3	CYCad_6	RISP_8	22	16	46	12	20	23	41	26	85	7	3	13	0	0	4	10	13	17	33	40	93	20	16	51	57	46	116	830	22.5575		
CYC1_4	GOT1Srg_5	ME1ad_12	9	9	22	12	16	34	40	31	55	10	8	23	15	13	11	11	16	47	22	18	51	29	22	60	54	47	145	830	22.5011		

P169_4	CYCad_6	GOT1p2_8	20	13	41	14	16	23	30	30	53	9	11	13	1	2	14	16	21	21	41	34	90	13	30	34	64	64	110	828	22.4399
X22708_3	QCR10p_4	QCR7p_6	18	12	56	13	6	22	47	23	69	6	0	2	3	0	3	10	2	17	42	34	58	27	8	44	103	45	135	805	22.4096
QCR9p_2	X34449_5	RISP_8	16	4	16	8	12	30	32	27	63	5	8	18	19	12	19	21	24	47	17	25	53	24	20	58	58	47	143	826	22.3911
QCR10p_4	GOT1Srg_5	CYCad_6	10	18	15	21	10	38	36	23	62	14	3	18	15	4	17	16	8	34	33	18	44	43	12	47	82	46	131	818	22.3834
QCR9p_2	CYC1_4	cytMDH_10	5	21	35	6	15	18	17	26	67	8	6	32	2	11	9	19	26	60	21	30	69	14	29	51	24	80	127	828	22.3559
ME2ad_3	RISP_8	X06422_10	11	23	43	4	22	28	14	60	71	1	9	3	2	5	2	3	6	14	7	38	68	10	29	75	28	96	160	832	22.3478
mtMDH_2	QCR8p_3	GOT2_8	17	16	49	2	2	3	44	37	59	19	11	16	2	0	3	23	22	53	33	24	87	4	0	5	66	48	178	823	22.3422
X22708_3	QCR10p_4	GOT2_8	27	17	44	9	11	20	42	22	78	3	0	5	5	1	0	4	6	19	33	26	76	27	17	37	60	57	170	816	22.3383
QCR7p_6	RPOL_7	cytMDH_10	11	32	34	13	8	30	18	37	84	2	11	13	4	12	12	9	19	48	20	30	60	7	26	44	31	65	126	806	22.3279
mtMDH_2	RISP_8	ME1ad_12	15	14	25	9	10	39	39	29	57	10	11	20	8	11	22	14	21	42	24	23	62	17	26	38	71	40	149	846	22.2597
ME2ad_3	QCR10p_4	RISP_8	24	16	48	11	15	15	42	22	77	4	1	3	4	2	1	5	7	19	34	36	66	22	19	39	55	56	174	817	22.2185
GDHad_1	QCR10p_4	RISP_8	18	19	31	13	8	10	26	16	83	8	6	11	7	4	7	7	14	28	32	28	75	17	24	37	70	55	156	810	22.2027
QCR8p_3	QCR7p_6	RISP_8	19	21	52	9	13	18	43	24	73	6	0	7	0	0	0	1	4	3	38	38	98	19	19	57	66	57	147	832	22.1588
X14140_3	QCR10p_4	GOT2_8	28	16	46	8	12	25	41	25	82	6	2	9	4	3	0	6	11	25	29	23	69	29	15	31	59	48	158	810	22.1575
GDHad_1	mtMDH_2	cytMDH_10	6	22	31	4	10	30	20	30	75	4	6	6	4	5	12	15	12	29	24	47	83	17	27	41	24	86	156	826	22.1343
QCR10p_4	GOT1Srg_5	RISP_8	15	6	21	16	19	34	30	28	61	5	10	19	14	11	11	18	15	25	21	22	52	23	16	62	57	47	155	813	22.1246
QCR10p_4	GOT1Srg_5	X06422_10	3	10	30	11	22	36	17	38	66	2	17	16	0	12	24	4	22	31	12	27	56	8	44	50	22	90	146	816	22.1048
X14140_3	X34449_5	CYCad_6	11	17	31	24	7	32	49	30	88	2	0	10	7	2	9	14	2	22	41	18	33	41	19	60	84	48	128	829	22.0774
ME2ad_3	QCR10p_4	QCR6p_9	18	15	55	9	12	20	49	23	69	1	0	7	1	3	3	7	6	18	40	26	72	16	13	52	59	50	175	819	22.0741
X14140_3	GOT2_8	X06422_10	14	24	40	2	19	34	18	64	74	1	8	7	0	10	6	6	11	18	8	43	66	4	25	60	27	85	152	826	22.0546
X22708_3	CYC1_4	QCR7p_6	15	11	56	16	7	24	50	25	68	6	0	5	5	1	1	8	1	16	42	29	62	35	13	51	98	48	130	823	21.9808
GDHad_1	ME2ad_3	QCR7p_6	22	7	50	1	1	7	55	30	54	9	4	13	4	0	4	18	10	31	51	31	83	14	1	13	101	52	155	821	21.9532
P102_3	RPOL_7	cytMDH_10	10	22	32	12	13	29	17	45	85	2	8	2	1	0	3	2	1	6	21	43	74	11	33	57	40	76	171	816	21.8869
ME2ad_3	P169_4	QCR7p_6	21	14	51	12	6	18	49	22	76	5	0	2	3	0	0	11	2	22	47	34	64	17	11	40	109	47	138	821	21.8633
X22708_3	QCR10p_4	CYCad_6	18	15	56	13	6	22	46	26	69	6	0	2	3	0	3	10	2	17	43	36	58	28	9	44	102	47	136	817	21.8489
QCR8p_3	QCR10p_4	QCR7p_6	21	11	54	17	5	21	47	22	66	5	0	1	2	0	3	6	0	4	39	34	61	24	8	43	107	47	153	801	21.8129
CYC1_4	X34449_5	GOT2_8	12	4	22	10	9	37	44	28	62	6	13	21	10	11	12	19	17	46	20	14	53	31	24	59	58	45	149	836	21.7879
X22708_3	P169_4	CYCad_6	20	15	52	12	6	18	48	28	76	5	0	2	2	0	0	11	2	20	49	37	62	18	11	40	108	46	141	829	21.762
X22708_3	CYC1_4	CYCad_6	15	14	56	16	7	24	49	28	68	6	0	5	5	1	1	8	1	16	44	30	62	36	13	51	96	52	131	835	21.7604
mtMDH_2	GOT2_8	cytMDH_10	14	25	25	9	13	33	11	36	63	8	10	26	2	12	18	15	20	39	15	28	60	13	20	42	31	80	162	830	21.7339
QCR8p_3	RISP_8	QCR6p_9	13	10	50	17	10	31	42	29	71	2	3	2	1	1	2	0	3	7	35	17	66	36	18	61	53	59	182	821	21.7106
X22708_3	QCR10p_4	QCR6p_9	18	14	57	9	12	20	49	23	69	1	0	7	1	2	3	6	6	17	40	27	70	16	13	52	60	50	174	816	21.685
ME2ad_3	CYC1_4	X06422_10	10	33	41	2	21	24	17	51	78	5	4	2	0	2	5	1	14	12	14	45	78	7	32	61	24	87	166	836	21.6254
X22708_3	CYC1_4	X06422_10	11	33	41	2	21	24	17	52	77	5	4	2	0	2	5	1	13	11	13	45	78	7	32	61	24	88	166	835	21.6128
GDHad_1	ME2ad_3	CYCad_6	21	8	50	1	1	7	55	30	55	9	5	13	4	0	4	19	11	31	51	35	83	14	1	13	100	57	155	833	21.6115
P169_4	RPOL_7	cytMDH_10	18	13	34	4	12	25	21	39	70	3	14	15	4	6	12	4	17	30	11	46	59	16	29	50	33	66	157	808	21.5722
P060_2	QCR10p_4	X34449_5	7	14	39	12	12	20	21	33	66	5	18	25	5	3	11	24	17	40	28	32	59	17	13	36	45	55	157	814	21.5679
QCR10p_4	GOT1Srg_5	GOT2_8	13	5	24	11	15	42	39	23	58	10	7	18	16	8	11	15	15	28	22	17	55	27	19	57	57	49	155	816	21.5057
QCR9p_2	CYC1_4	GOT2_8	21	9	31	11	14	14	31	22	55	13	11	21	10	3	9	25	20	61	32	19	68	14	24	56	52	39	144	829	21.4719
X34449_5	GOT1p1_8	X06422_10	3	15	16	2	6	23	11	34	55	4	14	34	5	15	28	10	50	42	16	40	72	2	28	56	27	86	138	832	21.4652
GDHad_1	ME2ad_3	GOT1Srg_5	13	13	54	2	1	6	27	37	75	9	8	10	0	0	8	15	12	33	37	51	80	5	8	15	67	78	166	830	21.4264
RPOL_7	RISP_8	cytMDH_10	12	18	21	8	13	20	13	41	67	9	8	21	4	14	28	11	25	39	15	30	66	21	24	43	23	67	153	814	21.4038
QCR9p_2	X34449_5	GOT1p1_8	12	4	20	8	13	29	39	29	54	3	6	22	16	12	22	30	13	49	19	21	55	28	23	51	59	42	147	826	21.3826
P060_2	QCR8p_3	QCR6p_9	18	12	50	1	2	4	39	19	79	9	10	28	0	4	1	25	15	58	43	27	73	2	1	5	60	59	174	818	21.3371
X14140_3	X34449_5	QCR7p_6	11	15	31	25	7	32	49	26	88	2	0	10	7	3	8	14	2	22	41	18	33	42	18	60	84	44	128	820	21.3299
mtMDH_2	X14140_3	ME1ad_12	19	25	46	2	7	7	42	21	68	16	14	24	1	3	8	14	25	52	37	36	82	13	4	24	63	49	142	844	21.3218
GDHad_1	P060_2	cytMDH_10	6	22	29	4	10	30	20	30	76	5	6	6	4	7	11	14	11	31	23	46	80	17	27	41	25	84	161	826	21.3107
X34449_5	CYCad_6	GOT1p2_8	13	9	33	3	13	21	18	23	33	21	11	40	7	8	13	23	36	41	37	38	72	17	27	37	69	55	114	832	21.2556
P060_2	RPOL_7	GOT2_8	21	14	25	11	11	16	32	28	62	11	11	16	10	4	22	22	18	35	27	16	69	21	22	41	54	34	160	813	21.2553
CYC1_4	RPOL_7	GOT1p2_8	20	9	29	12	12	16	35	33	59	12	12	5	6	8	17	19	26	46	28	40	58	23	19	46	53	49	121	813	21.2344
GDHad_1	X22708_3	QCR7p_6	21	8	51	1	1	6	56	29	54	9	4	13	4	0	4	18	10	31	51	31	84	13	1	12	101	51	156	820	21.207
mtMDH_2	QCR8p_3	QCR6p_9	19	11	53	1	2	4	39	21	81	9	10	28	0	4	1	25	15	58	44	28	72	2	1	6	61	58	169	822	21.1902
CYC1_4	RPOL_7	GOT2_8	18	6	34	11	13	15	37	21	71	12	9	8	7	8	16	16	21	54	29	26	70	24	16	48	55	37	135	817	21.1885
X14140_3	QCR10p_4	RISP_8	23	17	51	10	17	19	41	25	81	6	2	9	3	3	1	8	11	22	32	33	57	24	16	35	54	49	162	811	21.1408
QCR8p_3	QCR10p_4	CYCad_6	21	14	54	17	5	21	47	23	66	5	0	1	2	0	3	5	0	4	39	35	61	24	8	43	106	50	154	808	21.112
QCR8p_3	QCR10p_4	QCR6p_9	17	13	59	9	11	23	46	22	68	1	1	4	0	2	3	2	4	4	40	26	69	17	15	43	66	51	190	806	21.0763
X34449_5	QCR6p_9	X06422_10	2	9	32	1	13	16	13	33	46	3	26	28	3	11	15	13	42	63	4	42	55	11	35	45	30	77	163	831	21.0708
GDHad_1	X22708_3	CYCad_6	20	9	51	1	1	6	56	29	55	9	5	13	4	0	4	19	10	31	51	35	84	13	1	12	100	56	156	831	21.0378
GDHad_1	X22708_3	X34449_5	12	14	55	3	2	3	23	34	82	8	8	10	0	0	8	14	12	34	31	45	92	7	7	12	67	78	167	828	21.0329

All males

			Three Locus combinations (first/second/third locus genotype)																													Three Locus Totals	
first_locus	second_locus	third_locus	AA AA AA	AA AA DD	AA AA AD	AA DD AA	AA DD DD	AA DD AD	AA AD AA	AA AD DD	AA AD AD	DD AA AA	DD AA DD	DD AA AD	DD DD AA	DD DD DD	DD DD AD	DD AD AA	DD AD DD	DD AD AD	AD AA AA	AD AA DD	AD AA AD	AD DD AA	AD DD DD	AD DD AD	AD AD AA	AD AD DD	AD AD AD	Total #	chi-square value	Chromosome Combos	# p<0.05
mtMDH_2	P102_3	P169_4	5	5	23	0	0	0	7	8	9	6	5	10	2	0	4	8	13	44	13	21	29	1	1	3	22	7	70	316	35.4847	1,10,12	1
P060_2	P102_3	P169_4	5	5	23	0	0	0	7	8	9	6	5	10	2	0	4	8	13	45	13	21	30	1	1	3	22	7	68	316	35.0296	1,2,10	4
P060_2	QCR8p_3	P169_4	6	5	21	1	0	0	5	8	11	4	5	6	0	1	3	12	12	50	13	21	30	1	0	2	22	8	68	315	34.1509	1,3,10	1
mtMDH_2	QCR8p_3	P169_4	6	5	21	1	0	0	5	8	11	4	5	6	0	1	3	12	12	49	13	21	30	1	0	2	22	8	70	316	34.0628	1,3,12	3
QCR9p_2	X34449_5	QCR7p_6	4	4	6	3	2	7	7	9	27	1	4	11	0	1	23	7	15	23	2	14	21	8	14	14	20	18	50	315	31.2896	1,3,4	2
QCR9p_2	X34449_5	CYCad_6	4	4	6	3	2	7	7	9	27	1	5	11	0	1	23	7	15	23	2	14	21	8	14	14	20	18	50	316	30.9558	1,3,8	2
mtMDH_2	P102_3	QCR10p_4	2	5	25	0	0	0	6	9	9	6	4	12	2	2	2	8	18	37	12	17	30	0	1	4	21	14	64	310	30.5552	1,3,9	2
P060_2	QCR8p_3	CYC1_4	6	4	22	1	0	0	4	6	14	6	5	4	0	3	1	13	20	42	8	16	40	1	0	2	21	20	58	317	30.5474	1,4,10	1
P060_2	P102_3	X06422_10	15	2	16	0	0	0	10	6	8	8	1	12	1	2	3	24	11	30	12	12	39	5	0	0	35	15	46	313	30.3803	1,4,5	2
P060_2	QCR8p_3	X06422_10	15	2	15	0	1	0	10	5	9	7	0	8	1	2	1	25	12	36	14	10	39	3	0	0	35	17	45	312	30.3023	1,5,10	1
P060_2	P102_3	QCR10p_4	2	5	25	0	0	0	6	9	9	6	4	12	2	2	2	8	18	38	12	17	31	0	1	4	21	14	62	310	30.098	1,6,10	2
mtMDH_2	QCR8p_3	CYC1_4	5	4	22	1	0	0	4	6	14	6	5	4	0	3	1	13	20	41	8	16	40	1	0	2	21	20	60	317	30.0453	1,9,10	1
P102_3	P169_4	ME1ad_12	3	9	12	8	10	13	24	12	24	1	1	1	0	1	0	5	1	1	10	9	18	10	10	8	15	32	76	314	29.7506	2,3,10	9
P060_2	QCR8p_3	QCR10p_4	3	5	23	1	0	0	4	9	11	4	4	7	0	2	2	12	18	43	10	16	34	0	0	3	23	16	59	309	29.5632	2,3,12	6
mtMDH_2	QCR8p_3	QCR10p_4	3	5	23	1	0	0	4	9	11	4	4	7	0	2	2	12	18	42	10	16	34	0	0	3	23	16	61	310	29.367	2,3,4	24
mtMDH_2	P102_3	X06422_10	14	2	16	0	0	0	10	6	8	8	1	12	1	2	3	24	11	29	12	12	38	5	0	0	35	15	48	312	29.1287	2,3,5	12
mtMDH_2	QCR8p_3	X06422_10	14	2	15	0	1	0	10	5	9	7	0	8	1	2	1	25	12	35	14	10	39	3	0	0	35	17	47	312	28.8904	2,3,6	4
GDHad_1	P060_2	cytMDH_10	3	3	10	5	0	27	8	10	40	1	0	3	8	2	4	2	6	6	11	7	18	17	8	23	32	10	53	317	28.5981	2,3,7	3
QCR9p_2	P102_3	P169_4	5	6	22	0	0	1	14	7	15	9	5	9	2	0	3	9	11	38	10	20	31	1	1	3	13	10	70	315	28.5488	2,3,8	13
GDHad_1	X22708_3	P169_4	7	9	18	2	1	2	9	7	50	2	8	4	0	0	3	7	1	7	15	12	23	1	1	5	21	21	79	315	28.4107	2,4,10	2
GDHad_1	QCR9p_2	X06422_10	6	3	13	11	2	14	15	19	22	2	1	3	7	1	5	3	3	7	19	7	15	16	9	18	30	5	56	312	28.3812	2,4,8	2
GDHad_1	mtMDH_2	cytMDH_10	3	3	10	5	0	26	8	10	42	1	0	3	8	2	4	2	6	6	11	7	18	17	8	23	32	10	52	317	28.3405	2,5,10	3
QCR10p_4	GOT1Srg_5	cytMDH_10	5	2	5	7	1	11	1	5	20	12	1	7	3	3	7	11	8	18	5	7	30	12	7	24	29	12	59	312	28.1664	2,5,6	12
P060_2	QCR8p_3	ME1ad_12	15	6	11	1	0	0	3	7	14	2	5	8	1	1	2	18	22	35	18	18	28	2	0	1	17	25	56	316	27.1236	2,5,7	1
P060_2	X22708_3	X06422_10	11	2	13	0	1	0	13	5	10	8	0	9	1	5	1	24	9	35	13	8	33	3	1	3	36	19	49	312	27.109	2,8,10	3
QCR9p_2	QCR8p_3	P169_4	7	6	21	1	0	1	11	7	16	7	5	5	0	1	3	13	10	42	9	20	31	1	0	1	14	11	72	315	27.0084	3,4,10	2
mtMDH_2	X22708_3	X06422_10	11	2	13	0	1	0	13	5	10	8	0	9	1	5	1	24	9	34	13	8	33	3	1	3	36	19	50	312	26.8467	3,4,12	2
P060_2	QCR8p_3	RPOL_7	15	5	13	0	1	0	7	3	14	3	5	7	2	0	2	27	17	31	16	12	36	2	0	1	34	17	48	318	26.6881	3,4,7	1
P060_2	QCR8p_3	QCR7p_6	7	7	19	0	0	1	3	4	17	2	2	10	0	0	4	7	20	48	11	21	32	0	2	1	24	24	51	317	26.6483	3,4,8	5
P102_3	QCR10p_4	GOT1p1_8	5	2	13	6	5	15	11	13	44	2	0	0	2	0	1	2	3	1	3	17	15	13	5	23	27	18	65	311	26.3684	3,5,10	1
P060_2	QCR8p_3	GOT1Srg_5	8	8	16	0	0	1	9	3	12	6	1	8	1	1	2	15	25	35	17	17	30	1	2	0	20	18	61	317	26.3149	3,5,12	1
mtMDH_2	QCR8p_3	GOT1Srg_5	7	8	16	0	0	1	9	3	12	6	1	8	1	1	2	14	25	35	17	17	30	1	2	0	22	18	61	317	26.0723	3,6,10	4
QCR9p_2	RISP_8	X06422_10	6	2	6	10	9	8	11	0	17	7	2	12	9	6	8	18	4	17	16	3	15	14	6	17	18	18	52	311	25.9591	3,6,12	2
QCR10p_4	X34449_5	cytMDH_10	4	2	5	6	0	10	3	6	20	11	1	5	4	3	8	11	8	19	4	7	26	11	7	24	31	12	63	311	25.8768	3,8,10	3
QCR9p_2	GOT1Srg_5	X06422_10	4	1	12	9	0	4	14	10	15	12	4	5	9	5	10	14	3	22	7	5	27	13	5	19	28	17	39	313	25.8106	3,8,12	1
mtMDH_2	GOT1Srg_5	QCR7p_6	2	4	10	2	3	6	4	4	21	3	5	12	0	2	25	6	14	25	3	18	19	9	10	18	23	21	48	317	25.7441	4,5,10	4

P060_2	GOT1Srg_5	QCR7p_6	3	4	10	2	3	6	4	4	21	3	6	12	0	2	25	6	14	25	3	17	18	9	10	18	24	21	48	318	25.7311	4,8,10	4
GDHad_1	CYC1_4	cytMDH_10	5	3	10	4	3	19	7	7	49	2	0	6	3	3	1	6	5	6	11	4	18	22	3	17	27	18	58	317	25.5241	5,6,8	2
QCR9p_2	P102_3	QCR10p_4	2	6	24	0	0	1	12	9	15	9	4	11	2	1	2	7	15	34	9	16	32	0	2	3	15	17	61	309	25.4468	5,8,10	1
mtMDH_2	GOT1Srg_5	CYCad_6	2	4	10	2	3	6	4	4	21	3	6	12	0	2	25	6	14	25	3	18	19	9	10	18	23	21	48	318	25.3673	6,9,10	2
mtMDH_2	QCR8p_3	ME1ad_12	14	6	11	1	0	0	3	7	16	2	5	8	1	1	2	18	21	36	18	18	28	2	0	1	17	27	56	319	25.3521		
mtMDH_2	QCR8p_3	RPOL_7	14	5	13	0	1	0	7	4	15	3	5	7	2	0	2	27	18	30	16	12	36	2	0	1	35	17	49	321	25.2241		
P060_2	GOT1Srg_5	CYCad_6	3	4	10	2	3	6	4	4	21	3	7	12	0	2	25	6	14	25	3	17	18	9	10	18	23	21	49	319	25.1708		
P169_4	GOT1Srg_5	cytMDH_10	5	1	6	7	2	10	3	9	21	10	0	10	3	2	4	10	7	14	8	9	28	12	7	28	29	9	63	317	25.1047		
mtMDH_2	X34449_5	QCR7p_6	2	3	9	2	3	6	4	5	22	2	4	12	0	3	25	7	14	24	3	15	17	9	11	14	23	23	54	316	25.0116		
P060_2	QCR8p_3	RISP_8	7	12	13	0	1	0	6	8	10	3	1	11	0	0	4	22	19	34	12	19	32	0	2	1	22	26	51	316	24.9211		
P060_2	QCR8p_3	CYCad_6	6	8	19	0	0	1	3	4	17	2	3	10	0	0	4	7	20	48	11	21	32	0	2	1	24	24	51	318	24.9067		
GDHad_1	QCR9p_2	cytMDH_10	3	4	15	6	1	21	7	8	41	1	2	3	8	1	4	2	5	6	8	8	25	18	7	20	34	10	48	316	24.8144		
mtMDH_2	ME2ad_3	GOT1Srg_5	6	6	14	0	0	1	10	5	14	6	1	10	0	4	3	15	22	32	15	16	25	3	3	1	22	18	66	318	24.776		
mtMDH_2	QCR8p_3	RISP_8	7	12	13	0	1	0	6	9	11	3	1	11	0	0	4	22	20	33	12	19	32	0	2	1	22	26	53	320	24.7254		
mtMDH_2	X34449_5	CYCad_6	2	3	9	2	3	6	4	5	22	2	5	12	0	3	25	7	14	24	3	15	17	9	11	14	23	23	54	317	24.6033		
P102_3	QCR10p_4	GOT1p2_8	5	2	13	6	5	15	11	12	45	2	0	0	2	0	1	2	3	1	3	17	15	13	5	23	27	24	59	311	24.5804		
P060_2	X34449_5	QCR7p_6	2	3	9	2	3	6	4	5	22	2	5	12	0	3	25	7	14	24	3	14	16	9	11	14	24	23	54	316	24.5037		
P060_2	P102_3	ME1ad_12	15	6	12	0	0	0	4	7	13	3	7	12	3	2	1	15	19	32	18	18	26	3	1	1	16	24	58	316	24.4421		
QCR9p_2	QCR8p_3	QCR10p_4	3	6	24	1	0	1	10	9	15	7	4	6	0	2	2	11	14	39	7	15	34	0	0	2	17	20	60	309	24.4124		
P060_2	X22708_3	GOT1Srg_5	6	7	13	0	0	1	9	4	15	6	1	10	1	4	2	15	22	33	15	16	24	3	3	1	20	18	68	317	24.3683		
QCR9p_2	GOT1Srg_5	RPOL_7	6	1	10	6	0	7	11	10	18	10	0	11	10	3	11	14	12	17	18	8	14	10	13	14	22	12	49	317	24.3579		
P060_2	QCR8p_3	cytMDH_10	8	8	17	0	0	1	8	3	13	5	1	9	0	2	2	25	7	43	14	14	36	1	0	2	27	12	60	318	24.3561		
QCR8p_3	CYCad_6	cytMDH_10	3	2	14	3	10	19	21	11	29	0	0	0	1	0	1	0	2	4	12	8	14	11	6	31	37	8	72	319	24.1598		
P060_2	X34449_5	CYCad_6	2	3	9	2	3	6	4	5	22	2	6	12	0	3	25	7	14	24	3	14	16	9	11	14	23	23	55	317	24.1389		
mtMDH_2	QCR8p_3	cytMDH_10	7	8	17	0	0	1	8	3	13	5	1	9	0	2	2	25	7	42	14	14	36	1	0	2	27	12	62	318	24.1196		
GDHad_1	ME2ad_3	P169_4	7	9	20	2	1	3	9	7	47	2	8	4	0	0	2	7	1	8	14	12	23	1	1	6	22	21	79	316	24.0977		
P060_2	ME2ad_3	P169_4	4	5	17	1	0	0	7	8	14	4	7	6	1	1	5	11	10	48	15	17	24	1	1	6	20	11	72	316	24.0282		
mtMDH_2	ME2ad_3	P169_4	4	5	17	1	0	0	7	8	14	4	7	6	1	1	5	11	10	47	15	17	24	1	1	5	20	11	74	316	23.9909		
GDHad_1	CYCad_6	cytMDH_10	5	1	14	0	4	24	11	8	40	0	2	2	2	3	6	9	3	5	9	7	12	13	8	22	38	10	60	318	23.9049		
mtMDH_2	QCR8p_3	QCR7p_6	6	7	19	0	0	1	4	5	17	2	2	10	0	0	4	7	19	48	11	21	32	0	2	1	24	25	52	319	23.8972		
QCR9p_2	QCR10p_4	GOT1p2_8	1	5	8	2	1	12	10	7	23	4	6	8	2	2	16	8	14	25	5	8	11	17	7	11	22	18	57	310	23.6715		
mtMDH_2	X22708_3	GOT1Srg_5	6	7	13	0	0	1	9	4	15	6	1	10	1	4	2	14	22	33	15	16	24	3	3	1	22	18	67	317	23.6653		
mtMDH_2	QCR8p_3	CYCad_6	5	8	19	0	0	1	3	4	17	2	3	10	0	0	4	7	19	48	11	21	32	0	2	1	24	25	52	318	23.6037		
P060_2	X22708_3	P169_4	5	5	16	1	0	0	6	8	14	4	7	6	1	1	5	11	10	48	15	17	23	1	1	5	20	11	74	315	23.5097		
QCR8p_3	P169_4	ME1ad_12	4	7	12	8	10	13	22	12	22	1	0	1	0	0	1	3	1	1	9	12	18	10	11	7	19	32	78	314	23.5044		
P060_2	QCR8p_3	GOT1p2_8	5	5	21	0	0	1	5	5	14	0	1	14	0	1	3	17	18	40	15	11	38	1	2	0	29	25	45	316	23.4946		
P060_2	ME2ad_3	GOT1Srg_5	6	6	14	1	0	1	10	5	14	6	1	10	0	4	3	16	22	32	15	16	25	3	3	2	20	18	66	319	23.4934		
QCR9p_2	ME2ad_3	RISP_8	4	15	10	0	2	2	10	10	16	7	1	9	0	4	2	15	19	30	11	14	28	0	1	4	23	22	57	316	23.4798		
ME2ad_3	CYC1_4	RISP_8	3	5	9	9	7	12	10	18	26	0	4	1	0	0	3	0	3	4	8	17	12	14	13	17	27	21	74	317	23.4263		
mtMDH_2	X22708_3	P169_4	5	5	16	1	0	0	6	8	14	4	7	6	1	1	5	11	10	47	15	17	23	1	1	5	20	11	75	315	23.4135		
QCR9p_2	QCR8p_3	RPOL_7	15	4	15	0	1	1	8	8	20	7	4	7	3	0	1	24	11	31	12	14	34	1	0	1	37	20	42	321	23.1609		
GDHad_1	P102_3	ME1ad_12	13	9	18	0	2	1	13	10	39	2	7	5	2	0	0	3	8	5	20	15	27	4	1	1	19	32	59	315	23.1347		
QCR9p_2	QCR10p_4	GOT1p1_8	1	5	8	2	1	12	10	6	24	4	6	8	2	2	16	8	11	28	5	8	11	17	7	11	22	16	59	310	23.1244		
GDHad_1	ME2ad_3	QCR6p_9	7	3	25	1	0	5	21	18	26	3	3	8	1	1	0	2	2	12	10	10	29	1	0	7	23	19	80	317	23.1077		
QCR8p_3	RISP_8	X06422_10	12	1	8	6	7	19	17	4	34	0	0	0	2	1	0	2	2	1	17	6	26	25	13	14	28	15	51	311	23.0717		
mtMDH_2	QCR8p_3	GOT1p2_8	5	5	21	0	0	1	5	5	14	0	1	14	0	1	3	17	18	39	15	11	38	1	2	0	29	25	47	317	23.0529		
GDHad_1	X14140_3	QCR6p_9	7	2	23	2	0	5	20	19	28	3	2	9	1	1	0	2	3	11	14	10	29	1	0	10	19	19	76	316	23.0232		
GDHad_1	QCR7p_6	cytMDH_10	5	1	14	0	4	23	11	8	40	0	2	2	2	3	6	9	3	5	9	7	13	13	8	22	38	10	59	317	23.021		
P169_4	X34449_5	cytMDH_10	4	1	6	6	1	9	5	10	21	9	0	8	3	2	5	11	7	15	7	9	24	12	7	28	30	9	67	316	23.0101		
X22708_3	CYC1_4	RISP_8	3	5	10	9	7	11	10	17	26	0	3	1	0	0	4	0	3	4	8	18	11	14	13	17	27	22	73	316	22.9615		
GDHad_1	X22708_3	cytMDH_10	6	7	21	0	0	5	10	6	52	5	3	6	0	2	1	6	3	6	13	10	27	1	1	5	45	14	62	317	22.9387		
QCR9p_2	GOT1p1_8	X06422_10	6	1	6	9	2	1	12	8	24	5	0	9	7	5	6	22	7	22	17	8	18	8	3	20	23	16	47	312	22.8953		
P060_2	QCR8p_3	X34449_5	7	8	16	0	0	1	7	3	14	5	2	7	1	1	2	14	25	36	14	17	33	0	1	2	19	16	64	315	22.799		
mtMDH_2	QCR8p_3	X34449_5	7	8	16	0	0	1	7	3	14	5	2	7	1	1	2	13	25	36	14	17	33	0	1	2	21	16	64	316	22.757		
GDHad_1	QCR10p_4	GOT1Srg_5	1	6	9	5	4	12	21	13	34	0	3	7	4	0	5	2	5	6	11	10	10	11	9	20	19	25	59	311	22.7473		
P060_2	QCR8p_3	GOT2_8	4	5	23	0	0	1	5	4	15	2	2	11	0	1	3	19	18	38	14	13	37	1	2	0	31	24	44	317	22.722		
P060_2	QCR8p_3	GOT1p1_8	5	5	21	0	0	1	5	4	15	0	1	14	0	1	3	17	15	43	15	11	38	1	2	0	29	23	47	316	22.7201		
P102_3	QCR7p_6	ME1ad_12	8	4	7	5	8	17	23	18	26	0	1	0	4	0	0	2	2	2	6	6	21	9	10	28	20	35	56	318	22.6737		
mtMDH_2	P102_3	ME1ad_12	14	6	12	0	0	0	4	7	15	3	7	12	3	2	1	15	18	32	18	18	26	3	1	1	16	26	58	318	22.579		
X22708_3	QCR10p_4	X06422_10	7	2	8	10	4	13	14	4	34	0	3	0	1	2	1	3	2	3	15	1	20	12	11	16	45	20	56	307	22.544		
P060_2	QCR10p_4	X06422_10	4	3	1	6	1	7	14	4	16	9	2	5	9	5	10	14	7	30	9	1	22	8	11	13	34	15	47	307	22.516		
P102_3	CYCad_6	ME1ad_12	8	4	7	5	9	17	23	18	26	0	1	0	4	0	0	2	2	2	6	6	20	9	10	27	20	35	56	317	22.4901		
GDHad_1	X34449_5	cytMDH_10	3	5	14	3	0	19	10	8	45	1	3	3	4	1	3	6	4	6	16	2	21	14	9	20	30	14	53	317	22.4764		
QCR9p_2	X14140_3	ME1ad_12	11	6	10	3	0	1	7	9	24	4	7	7	0	4	4	15	16	30	14	11	29	0	2	6	22	30	46	318	22.4509		
P060_2	P102_3	GOT1p2_8	6	6	20	0	0	0	4	4	16	0	2	20	3	1	2	14	17	35	16	11	37	3	2	0	26	25	47	317	22.4451		
mtMDH_2	P102_3	GOT1p2_8	6	6	20	0	0	0	4	4	16	0	2	20	3	1	2	14	17	34	16	11	36	3	2	0	26	25	49	317	22.301		
X22708_3	RISP_8	X06422_10	12	0	10	6	6	17	14	4	28	0	0	0	2	3	1	2	4	3	17	7	24	25	12	15	31	14	55	312	22.282		
GDHad_1	QCR8p_3	ME1ad_12	13	10	15	0	0	2	13	11	41	2	6	5	2	0	0	3	9	5	19	13	27	2	1	1	22	34	59	315	22.2759		
mtMDH_2	QCR8p_3	GOT2_8	4	5	23	0	0	1	5	4	15	2	2	11	0	1	3	19	18	37	14	13	37	1	2	0	31	24	46	318	22.2406		
mtMDH_2	QCR8p_3	GOT1p1_8	5	5	21	0	0	1	5	4	15	0	1	14	0	1	3	17	15	42	15	11	38	1	2	0	29	23	49	317	22.2324		
mtMDH_2	GOT1Srg_5	X06422_10	6	2	8	8	0	3	10	6	13	10	2	9	10	7	10	13	5	25	6	6	27	13	3	21	33	19	38	313	22.1925		
mtMDH_2	QCR10p_4	X06422_10	4	3	1	6	1	7	14	4	16	9	2	5	9	5	10	14	7	29	9	1	22	8	11	13	34	15	48	307	22.1769		
QCR9p_2	GOT1Srg_5	CYCad_6	3	6	8	3	2	8	8	7	24	3	8	10	0	1	23	6	12	25	3	14	23	8	12	17	19	20	45	318	22.1762		
GDHad_1	QCR10p_4	X34449_5	0	6	10	3	4	14	19	12	37	1	3	5	4	0	5	2	5	6	10	7	14	10	11	19	16	25	62	310	22.0741		
CYC1_4	GOT1p1_8	X06422_10	1	0	9	9	3	8	12	2	14	6	5	7	5	2	3	18	8	20	21	4	17	10	5	16	27	21	60	313	22.0658		
QCR9p_2	QCR8p_3	cytMDH_10	6	9	19	0	0	2	6	6	22	8	2	8	1	2	1	24	5	37	13	12	35	0	0	2	30	11	57	318	22.0501		
X34449_5	CYCad_6	GOT1p2_8	1	5	1	3	5	15	10	9	19	2	2	7	9	3	5	8	12	25	6	4	24	13	8	21	20	20	61	318	22.0435		
ME2ad_3	QCR10p_4	X06422_10	7	2	8	10	5	13	14	5	33	0	3	0	1	1	1	3	2	5	15	1	20	12	11	16	45	19	56	308	22.013		
X34449_5	RISP_8	cytMDH_10	9	2	6	7	1	11	4	7	21	7	1	8	7	0	12	7	9	22	10	3	25	10	11	28	26	12	51	317	22.0048		
QCR9p_2	X22708_3	GOT1Srg_5	5	7	16	2	1	1	9	5	22	6	1	10	2	3	1	12	20	32	16	16	21	0	3	2	24	18	61	316	22.0042		
P060_2	X14140_3	GOT1Srg_5	6	6	12	0	0	1	10	5	16	6	1	10	1	6	3	15	20	32	14	15	30	4	3	2	20	19	60	317	21.9774		
X14140_3	X34449_5	ME1ad_12	9	4	10	8	2	11	12	18	25	0	1	3	1	5	2	2	0	6	8	15	18	14	11	19	22	29	61	316	21.9518		
CYCad_6	QCR6p_9	X06422_10	3	1	6	1	4	4	16	4	11	4	6	8	5	1	9	10	6	32	19	8	13	9	3	19	42	17	52	313	21.8888		
QCR8p_3	QCR7p_6	cytMDH_10	3	3	14	3	9	18	21	11	29	0	0	0	1	0	1	0	2	4	12	8	14	11	6	31	37	8	72	318	21.8809		
CYC1_4	GOT2_8	X06422_10	2	0	9	10	3	9	10	2	13	7	7	8	7	2	5	15	6	17	19	3	19	10	7	15	29	20	59	313	21.8635		
QCR9p_2	GOT1Srg_5	QCR7p_6	3	6	8	3	2	8	8	7	24	3	7	10	0	1	23	6	12	25	3	14	23	8	12	17	19	20	45	317	21.8421		
P060_2	ME2ad_3	CYC1_4	4	4	18	2	0	0	5	6	18	5	8	4	3	2	2	11	18	41	8	16	32	1	1	5	21	20	63	318	21.8047		
P060_2	P102_3	GOT1p1_8	6	6	20	0	0	0	4	3	17	0	2	20	3	1	2	14	14	38	16	12	36	3	2	0	26	23	49	317	21.7671		
mtMDH_2	P102_3	GOT1p1_8	6	6	20	0	0	0	4	3	17	0	2	20	3	1	2	14	14	37	16	11	36	3	2	0	26	23	51	317	21.7102		
X34449_5	QCR7p_6	GOT1p2_8	1	5	1	3	5	14	10	9	19	2	2	7	9	3	5	8	12	25	6	4	25	13	8	21	20	20	60	317	21.6855		
GDHad_1	X22708_3	ME1ad_12	10	8	15	0	1	4	16	12	40	3	7	4	2	0	1	2	8	5	17	9	24	3	3	1	23	35	62	315	21.6427		
X22708_3	CYCad_6	cytMDH_10	1	2	10	4	7	18	19	11	26	0	0	1	1	0	3	0	3	7	13	8	17	10	8	31	38	7	73	318	21.6151		
mtMDH_2	X14140_3	GOT1Srg_5	6	6	12	0	0	1	10	5	16	6	1	10	1	6	3	14	20	32	14	15	30	4	3	2	22	19	60	318	21.6078		
P102_3	GOT1p2_8	ME1ad_12	8	5	9	6	5	8	21	21	33	4	2	0	1	1	1	1	0	1	8	6	30	8	13	25	19	32	48	316	21.5456		
GDHad_1	QCR6p_9	cytMDH_10	6	5	18	4	4	13	6	4	46	1	1	4	2	1	3	8	6	6	7	9	18	11	5	13	42	11	63	317	21.5075		
GDHad_1	X22708_3	GOT2_8	6	6	22	0	4	1	17	9	42	3	2	9	2	0	1	4	6	5	10	11	29	1	2	4	32	29	60	317	21.4847		
X14140_3	P169_4	RPOL_7	8	6	10	8	7	13	14	7	28	2	1	1	1	0	1	1	2	11	14	4	18	13	1	16	45	31	53	316	21.4664		
ME2ad_3	RISP_8	X06422_10	12	0	10	6	7	17	14	5	27	0	0	0	2	3	2	2	3	4	17	7	24	25	11	14	31	14	56	313	21.4622		
GDHad_1	ME2ad_3	GOT1p2_8	8	4	24	0	4	2	14	11	40	2	3	9	2	0	0	4	6	6	12	10	27	1	2	5	29	27	66	318	21.4616		

QCR9p_2	QCR8p_3	GOT1Srg_5	6	7	20	0	1	1	11	5	18	7	2	9	2	1	1	12	21	33	18	17	25	0	1	1	22	19	57	317	21.4182
QCR9p_2	X22708_3	P169_4	7	5	16	1	1	2	11	7	18	6	7	4	1	1	4	13	8	42	11	17	25	1	0	4	12	14	76	314	21.3709
P102_3	QCR10p_4	GOT2_8	6	4	10	7	5	14	10	14	44	1	0	1	2	0	1	2	3	1	3	17	15	15	8	18	28	19	63	311	21.3498
P169_4	GOT2_8	cytMDH_10	1	3	4	4	2	15	10	7	18	9	3	4	6	1	3	8	5	21	17	4	31	8	7	23	24	15	65	318	21.3328
mtMDH_2	X14140_3	P169_4	4	5	16	1	0	0	7	8	16	4	7	6	1	1	8	11	10	44	16	16	27	2	1	6	18	12	70	317	21.33
P060_2	X14140_3	P169_4	4	5	16	1	0	0	7	8	16	4	7	6	1	1	8	11	10	45	16	16	27	2	1	6	18	12	68	316	21.2796
mtMDH_2	X34449_5	X06422_10	6	2	6	8	0	3	10	6	15	7	2	9	10	7	11	15	5	24	4	5	25	12	3	19	36	20	42	312	21.2649
mtMDH_2	ME2ad_3	QCR10p_4	3	5	18	1	0	0	4	9	16	3	6	8	2	1	4	11	17	39	12	17	26	0	2	5	21	13	68	311	21.2618
CYC1_4	GOT1p2_8	X06422_10	1	0	9	9	3	8	12	2	14	6	5	7	5	2	3	18	8	20	21	4	17	11	7	19	26	19	57	313	21.2575
P060_2	ME2ad_3	QCR10p_4	3	5	18	1	0	0	4	9	16	3	6	8	2	1	4	11	17	40	12	17	26	0	2	6	21	13	66	311	21.2438
QCR9p_2	QCR8p_3	ME1ad_12	13	6	14	2	0	0	6	9	21	3	6	9	2	1	1	15	20	31	19	17	24	0	0	2	17	26	55	319	21.1937
P102_3	CYCad_6	cytMDH_10	3	2	15	5	10	19	21	11	36	0	0	1	2	0	2	2	2	2	12	8	12	10	6	30	35	8	68	322	21.1874
QCR7p_6	QCR6p_9	X06422_10	3	1	6	1	4	4	16	4	12	4	6	8	5	1	9	10	6	31	19	8	13	9	3	19	42	17	51	312	21.118
QCR9p_2	GOT1p2_8	X06422_10	6	1	6	9	2	2	12	8	23	5	0	9	7	6	8	22	6	20	17	8	18	9	4	20	22	15	47	312	21.0998
ME2ad_3	X34449_5	X06422_10	6	0	16	9	2	12	17	10	26	0	0	1	1	4	1	3	2	4	11	9	23	20	4	20	41	19	52	313	21.0824
GDHad_1	cytMDH_10	ME1ad_12	4	2	10	4	5	4	18	14	45	2	6	3	3	2	3	2	7	4	17	14	29	4	11	10	22	23	48	316	21.0262

DA all F2 adults

			Three Locus combinations (first/second/third locus genotype)																													Three Locus Totals	
first_locus	second_locus	third_locus	AA AA AA	AA AA DD	AA AA AD	AA DD AA	AA DD DD	AA DD AD	AA AD AA	AA AD DD	AA AD AD	DD AA AA	DD AA DD	DD AA AD	DD DD AA	DD DD DD	DD DD AD	DD AD AA	DD AD DD	DD AD AD	AD AA AA	AD AA DD	AD AA AD	AD DD AA	AD DD DD	AD DD AD	AD AD AA	AD AD DD	AD AD AD	Total #	chi-square value	Chromosome Combos	# p<0.05
QCR10p_4	RISP_8	I06422_10	2	20	27	7	6	18	12	16	39	0	11	14	3	18	12	3	16	24	10	20	41	4	23	34	23	71	107	581	30.5	1,2,4	1
QCR10p_4	GOT1Srg_5	CYCad_6	3	18	8	12	6	24	23	14	39	9	4	18	10	5	12	11	7	27	20	18	32	31	11	38	49	38	102	589	29.8771	1,2,5	1
GDHad_1	P169_4	GOT2_8	12	17	19	8	3	14	26	12	55	8	6	9	5	3	5	7	4	21	25	9	42	9	20	33	41	43	132	588	29.8512	1,2,8	1
CYCad_6	GOT1p2_8	I06422_10	4	15	25	1	16	26	12	41	29	7	6	11	1	13	23	4	24	36	8	26	40	16	26	54	12	40	79	595	29.6666	1,2,12	1
GDHad_1	QCR10p_4	RISP_8	16	13	17	8	7	9	20	12	64	7	6	6	5	2	5	3	11	21	22	12	44	12	25	28	48	40	114	577	29.1988	1,3,6	3
QCR7p_6	GOT1p2_8	I06422_10	4	15	25	1	17	25	12	41	28	7	6	10	1	12	20	4	22	30	8	26	40	16	26	54	12	40	78	580	28.9223	1,4,5	1
CYC1_4	RISP_8	cytMDH_10	6	14	25	6	6	18	8	13	42	6	2	10	4	16	18	6	20	35	11	26	42	16	13	33	16	55	127	594	28.4256	1,4,7	2
QCR10p_4	I34449_5	GOT2_8	7	3	16	5	10	24	33	15	30	7	9	14	7	6	7	13	12	27	11	8	46	22	19	42	38	33	122	586	27.883	1,4,8	5
I34449_5	GOT1p1_8	cytMDH_10	2	11	13	3	3	15	7	27	40	1	14	22	6	8	23	13	24	33	14	21	47	6	23	31	26	35	127	595	27.5501	1,6,10	2
QCR9p_2	GOT1Srg_5	GOT1p1_8	10	2	15	7	12	19	25	21	38	1	0	21	7	9	14	19	7	35	18	16	47	25	16	41	34	34	102	595	27.452	2,3,4	2
QCR7p_6	GOT1p1_8	I06422_10	4	16	25	0	8	19	13	50	34	7	6	12	0	8	14	5	26	33	8	28	42	9	16	38	19	49	94	583	27.4446	2,3,7	4
CYCad_6	GOT1p1_8	I06422_10	4	15	25	0	8	20	13	50	35	7	7	13	0	8	16	5	28	41	8	28	42	9	16	38	19	49	94	598	27.4347	2,3,8	7
P102_3	CYC1_4	RPOL_7	13	4	30	4	9	24	36	27	50	1	0	2	3	0	0	0	2	3	19	14	54	10	15	47	61	35	118	581	27.3875	2,3,9	1
I14140_3	RPOL_7	GOT1p1_8	11	21	30	15	6	20	32	16	65	5	1	4	0	2	9	6	6	11	17	14	42	20	12	23	40	33	117	578	27.1217	2,3,12	3
P169_4	I34449_5	GOT2_8	9	3	14	4	11	21	33	17	34	5	9	13	6	7	7	11	11	32	11	8	50	23	18	45	40	35	115	592	26.9152	2,4,7	3
QCR10p_4	GOT1Srg_5	GOT1p1_8	10	4	15	6	13	22	29	11	36	6	7	18	9	5	13	14	12	19	13	7	50	24	18	38	35	37	117	588	26.4332	2,4,8	8
I14140_3	P169_4	RISP_8	11	11	26	5	16	23	36	22	71	4	0	0	2	1	1	6	8	20	32	23	47	13	14	27	34	37	103	593	26.287	2,5,6	4
I14140_3	RPOL_7	GOT1p2_8	11	27	24	15	8	18	31	24	56	5	2	4	0	5	6	5	11	6	17	22	35	18	13	23	38	54	97	575	26.1488	2,5,7	2
I34449_5	CYCad_6	GOT1p1_8	8	3	19	3	5	28	15	13	28	15	7	30	7	6	10	15	24	30	21	19	50	15	13	36	47	28	104	599	25.9557	2,5,8	6
I34449_5	GOT1p2_8	cytMDH_10	2	11	13	4	12	21	6	18	34	1	14	22	8	11	27	10	21	30	13	21	43	15	35	44	18	24	115	593	25.6303	2,7,8	2
QCR9p_2	I34449_5	GOT1p1_8	8	3	14	5	10	18	29	22	40	1	1	19	9	9	15	17	6	35	17	17	40	23	18	37	37	31	112	593	25.5758	2,7,10	2
GDHad_1	P169_4	RISP_8	15	14	19	6	6	13	23	12	58	8	7	7	5	3	5	2	10	19	22	13	43	9	22	31	51	44	118	585	25.2511	2,8,9	1
I34449_5	QCR7p_6	GOT1p1_8	8	3	18	3	4	25	15	13	28	15	7	30	7	6	9	15	24	30	21	18	50	14	12	30	47	28	104	584	25.1406	3,4,6	3
QCR10p_4	GOT1Srg_5	RISP_8	11	3	14	10	11	21	27	17	31	2	12	16	8	9	10	15	13	17	14	12	44	18	12	49	38	40	110	584	25.0759	3,4,7	1
GDHad_1	P169_4	RPOL_7	8	5	35	6	5	14	25	12	53	2	2	16	7	3	3	7	10	12	25	13	39	18	10	32	50	44	113	569	24.9135	3,4,8	7
CYC1_4	RISP_8	ME1ad_12	11	17	17	5	8	17	21	11	32	6	2	10	5	9	24	20	13	28	21	16	41	13	16	34	40	40	118	595	24.8557	3,5,6	2
P060_2	ME2ad_3	GOT1p1_8	17	11	32	2	0	3	24	27	47	9	1	12	2	3	2	14	14	41	33	29	61	0	3	10	45	30	128	600	24.8254	3,5,8	1
mtMDH_2	CYC1_4	RPOL_7	10	4	23	9	6	26	33	13	41	0	5	19	2	4	9	13	18	23	23	9	44	6	14	36	51	33	105	579	24.7957	3,6,8	1
QCR10p_4	I34449_5	CYCad_6	3	15	8	12	6	22	23	16	40	9	4	17	6	5	9	15	7	31	18	17	30	33	11	39	49	39	104	588	24.7223	3,6,10	2
GDHad_1	CYCad_6	cytMDH_10	10	12	29	5	8	22	8	18	56	3	6	11	1	3	8	11	7	19	3	35	60	4	26	48	34	52	94	593	24.628	3,7,8	3
QCR9p_2	I34449_5	RISP_8	10	4	11	4	9	20	24	23	44	2	2	17	12	9	12	16	17	25	14	20	40	19	14	45	40	33	107	593	24.6171	3,7,12	1
I14140_3	CYC1_4	CYCad_6	10	11	24	10	7	26	41	30	62	4	1	6	5	0	0	4	1	24	22	19	42	19	13	37	55	42	82	597	24.561	3,8,9	1
QCR10p_4	GOT1Srg_5	QCR7p_6	3	15	8	12	6	23	22	12	40	8	4	18	10	5	12	11	6	27	20	17	32	31	11	38	49	33	101	574	24.5127	3,8,10	3
mtMDH_2	GOT1Srg_5	CYCad_6	9	7	19	17	8	22	20	25	43	4	5	13	5	3	17	12	11	25	18	28	27	32	12	35	53	25	103	598	24.4742	4,5,6	4
GDHad_1	QCR7p_6	cytMDH_10	10	12	29	5	8	20	8	18	55	3	5	11	1	3	7	11	7	19	3	35	60	3	23	43	34	53	93	579	24.4573	4,5,7	1
CYC1_4	RISP_8	I06422_10	1	18	26	5	9	16	13	19	32	0	5	13	3	19	15	5	24	32	11	26	43	6	20	35	21	65	111	593	24.4437	4,5,8	11
QCR10p_4	RISP_8	cytMDH_10	10	14	25	7	4	20	6	14	46	6	4	15	5	11	18	3	15	25	8	24	38	14	19	30	21	55	126	583	24.4136	4,5,9	1
mtMDH_2	ME2ad_3	ME1ad_12	12	17	34	0	3	3	32	18	50	8	6	7	1	2	4	9	18	40	23	30	69	5	0	7	53	37	108	596	24.3814	4,5,10	2
QCR10p_4	GOT1Srg_5	GOT2_8	9	3	16	6	12	23	30	14	31	6	8	17	8	7	11	13	12	20	13	9	48	23	16	41	35	35	120	586	24.3793	4,6,8	4
mtMDH_2	RPOL_7	GOT2_8	15	13	23	5	5	13	26	19	45	2	5	8	9	2	15	15	11	25	14	15	52	18	16	23	38	26	120	578	24.3501	4,7,8	2
I14140_3	CYC1_4	QCR7p_6	10	9	24	10	7	26	41	25	62	4	2	5	5	0	0	4	1	24	21	17	43	19	13	37	56	39	81	585	24.2438	4,7,10	1
P169_4	QCR7p_6	GOT1p2_8	16	6	21	10	7	15	18	25	26	8	10	7	1	2	13	12	23	24	19	27	53	12	24	28	44	50	78	579	24.0619	4,7,12	1
mtMDH_2	ME2ad_3	GOT1p1_8	18	11	34	2	1	3	24	28	49	9	1	12	2	3	2	15	13	39	34	29	59	0	2	10	44	29	124	597	24.0459	4,8,10	7
CYC1_4	RPOL_7	cytMDH_10	7	8	17	0	3	15	12	22	52	3	9	5	5	8	11	7	20	44	6	33	58	12	15	37	24	46	97	576	24.016	4,8,12	1
I14140_3	CYCad_6	I06422_10	8	24	29	5	22	21	15	32	64	0	4	8	0	2	0	3	15	12	9	45	41	7	18	48	18	45	97	592	24.0063	4,10,12	1
P169_4	GOT1Srg_5	GOT1p1_8	11	4	14	5	13	20	30	14	39	5	6	20	9	6	13	8	13	23	13	8	51	24	18	40	40	36	112	595	23.9825	5,6,8	7
QCR9p_2	GOT1Srg_5	RISP_8	10	3	14	7	10	21	21	23	40	2	2	18	8	9	13	20	18	23	15	22	44	21	14	47	37	31	102	595	23.9386	5,8,10	2
CYC1_4	I34449_5	GOT2_8	5	2	14	4	8	21	32	16	34	5	9	14	7	7	11	12	16	36	15	9	49	23	21	42	39	30	117	598	23.9305	6,8,10	4
I34449_5	CYCad_6	GOT1p2_8	8	8	14	3	9	24	15	20	21	15	9	28	7	8	8	15	29	25	21	27	41	13	19	32	44	48	86	597	23.792	6,8,12	2
I14140_3	P169_4	GOT2_8	14	12	19	6	13	24	34	19	77	3	0	1	2	1	1	4	9	22	29	19	50	14	13	27	36	33	106	588	23.7908	6,9,10	2
P102_3	GOT1p1_8	I06422_10	9	12	31	3	14	19	10	49	50	2	0	1	0	1	1	0	3	3	8	38	48	6	16	51	26	73	112	586	23.7561	6,10,12	2
P169_4	GOT1Srg_5	GOT2_8	11	3	14	5	12	20	30	17	35	6	7	18	9	8	10	7	12	25	11	10	51	22	16	45	41	35	113	593	23.7509	7,8,10	1
I22708_3	QCR10p_4	RISP_8	13	11	23	7	15	15	32	16	65	3	0	1	2	0	1	2	5	8	32	20	43	16	18	27	37	43	130	585	23.7359	8,9,12	1
QCR9p_2	CYC1_4	GOT2_8	13	5	14	11	13	10	21	18	43	9	8	11	5	1	9	13	12	44	19	13	44	8	18	42	42	30	118	594	23.7095		
P060_2	RPOL_7	GOT2_8	15	13	21	5	5	13	25	18	42	2	5	8	9	2	15	14	11	27	14	15	53	17	16	23	40	28	122	578	23.6782		
P169_4	QCR7p_6	GOT2_8	15	7	21	13	3	15	18	20	32	9	7	8	2	2	12	11	18	30	18	19	63	12	14	38	42	27	105	581	23.5157		
CYC1_4	RPOL_7	ME1ad_12	9	5	19	2	9	7	25	22	39	9	1	7	4	7	13	17	15	39	16	21	60	16	12	36	41	35	91	577	23.4862		
QCR10p_4	I34449_5	cytMDH_10	5	3	18	7	9	24	9	21	48	5	10	15	4	5	11	5	15	33	2	27	35	9	31	43	32	41	117	584	23.4861		
QCR9p_2	QCR10p_4	RISP_8	13	7	14	7	10	12	19	19	47	9	11	13	6	1	5	15	16	36	26	13	40	12	23	26	35	29	119	583	23.4587		
P060_2	I22708_3	GOT1p2_8	17	18	25	2	0	3	22	33	43	8	1	12	2	2	2	14	29	27	32	38	49	0	4	7	43	51	111	595	23.4557		
CYC1_4	GOT1p1_8	I06422_10	3	16	24	3	9	13	13	21	38	1	8	19	2	10	16	5	30	26	15	24	37	4	13	45	19	76	106	596	23.4522		
QCR9p_2	GOT1Srg_5	GOT1p2_8	10	5	12	7	15	16	23	24	37	1	6	15	7	14	9	19	12	29	18	22	41	25	20	37	32	59	79	594	23.4477		
P169_4	RISP_8	I06422_10	2	19	26	7	7	20	12	17	40	0	10	10	3	13	14	4	18	29	10	20	46	4	28	32	23	72	102	588	23.4434		
QCR9p_2	GOT1Srg_5	GOT2_8	12	4	10	10	11	16	23	21	40	2	1	19	5	9	16	20	11	29	14	15	52	22	16	44	34	31	107	594	23.3835		
P060_2	GOT1Srg_5	CYCad_6	9	8	18	17	8	22	18	24	39	4	6	13	5	3	17	14	11	24	19	27	28	31	12	35	54	26	108	600	23.3742		
QCR9p_2	CYC1_4	RPOL_7	9	4	19	7	6	20	31	15	34	1	6	21	3	2	10	15	19	32	23	8	46	7	16	41	50	30	102	577	23.2747		
P060_2	CYC1_4	RPOL_7	9	4	21	8	6	25	33	13	39	0	5	18	2	4	9	13	18	25	23	8	47	7	14	37	51	33	106	578	23.2707		
ME2ad_3	QCR10p_4	RISP_8	14	11	22	7	15	15	33	17	66	3	0	1	2	1	1	2	6	9	32	20	44	16	18	27	35	41	129	587	23.2641		
mtMDH_2	I22708_3	GOT1p2_8	18	18	27	2	0	3	22	34	45	8	1	12	2	2	2	14	28	26	33	38	47	0	4	7	43	50	106	592	23.2191		
P169_4	GOT1Srg_5	RPOL_7	12	1	14	8	6	23	15	14	53	10	1	18	9	10	8	12	7	24	17	13	40	21	15	46	44	38	94	573	23.1562		
mtMDH_2	QCR10p_4	RISP_8	14	6	14	8	14	17	18	24	54	13	7	9	4	2	5	11	12	31	22	18	44	13	18	21	42	27	116	584	23.019		
QCR10p_4	I34449_5	GOT1p1_8	7	5	14	6	11	22	31	12	36	8	8	14	7	4	9	14	12	27	11	8	46	24	21	38	38	33	121	587	23.0122		
CYC1_4	RPOL_7	GOT2_8	8	5	20	7	7	3	26	14	45	5	6	6	5	8	11	14	15	42	18	22	56	19	8	37	39	27	105	578	22.8689		
P060_2	I14140_3	RPOL_7	25	7	33	5	1	1	20	15	51	6	7	12	0	4	6	9	16	34	31	26	68	6	6	16	44	24	105	578	22.804		
mtMDH_2	I22708_3	GOT1p1_8	18	12	33	2	0	3	24	27	50	8	1	12	2	2	2	15	14	39	34	29	57	0	1	10	45	30	125	595	22.7668		
RPOL_7	RISP_8	cytMDH_10	8	8	13	4	8	18	5	33	50	6	7	16	3	9	18	8	10	29	10	26	47	17	18	33	16	44	114	578	22.6739		
CYCad_6	I06422_10	ME1ad_12	4	4	9	15	11	46	18	19	41	6	2	4	13	9	20	14	20	35	9	6	21	10	24	59	54	35	85	593	22.6317		
mtMDH_2	I14140_3	QCR6p_9	16	15	38	4	3	1	25	10	57	4	4	17	2	2	6	17	9	34	45	27	55	8	1	18	46	35	94	593	22.5728		
GDHad_1	I22708_3	QCR7p_6	13	6	39	0	0	2	38	27	40	6	3	10	2	0	2	11	8	25	36	29	56	5	1	9	57	39	118	582	22.5418		

QCR9p_2	I14140_3	P169_4	13	11	33	0	3	10	24	13	42	9	5	26	0	0	8	31	6	29	26	28	70	4	1	17	48	35	102	594	22.5349
I14140_3	P169_4	CYCad_6	11	11	24	11	6	27	39	31	60	3	0	1	2	0	2	7	2	26	29	25	46	12	11	30	54	36	85	591	22.5018
P060_2	I22708_3	GOT1p1_8	17	12	31	2	0	3	24	26	48	8	1	12	2	2	2	14	15	41	33	29	59	0	1	10	46	31	129	598	22.4944
QCR7p_6	QCR6p_9	cytMDH_10	4	20	26	2	3	20	10	28	55	2	10	23	0	10	14	7	14	33	9	23	44	12	19	24	32	36	102	582	22.4343
mtMDH_2	QCR10p_4	GOT2_8	14	5	14	12	12	14	20	22	54	11	6	10	3	0	8	12	11	31	20	17	46	12	15	25	39	26	123	582	22.4306
P060_2	QCR10p_4	RISP_8	14	6	13	8	13	16	16	24	51	12	7	9	4	2	5	11	12	33	23	18	43	13	19	22	44	28	118	584	22.3704
QCR10p_4	I34449_5	QCR6p_9	12	3	11	10	6	24	23	12	44	9	7	14	8	5	7	7	9	37	20	7	38	31	9	43	45	44	100	585	22.3529
mtMDH_2	I22708_3	ME1ad_12	12	18	33	0	2	3	32	17	51	8	6	7	1	2	3	9	18	41	23	30	67	4	0	7	53	38	110	595	22.3515
GDHad_1	P169_4	GOT1Srg_5	5	15	28	7	9	9	25	12	56	5	3	15	5	2	6	7	7	18	19	19	39	19	17	27	40	63	111	588	22.3446
mtMDH_2	RPOL_7	I06422_10	7	14	30	4	9	10	8	32	48	1	4	11	4	5	17	5	21	25	9	27	45	3	31	23	23	56	105	577	22.3121
QCR7p_6	GOT2_8	ME1ad_12	10	8	24	5	12	16	22	15	55	12	5	10	3	6	10	15	15	36	11	25	35	15	10	40	47	31	91	584	22.3031
P169_4	CYCad_6	GOT1p2_8	16	7	21	10	9	17	18	25	25	8	10	8	1	2	14	12	23	24	19	27	53	13	25	32	44	50	80	593	22.2872
GDHad_1	QCR10p_4	GOT2_8	12	14	20	9	3	12	25	15	56	6	5	10	6	3	3	8	5	22	25	10	41	12	20	33	38	38	129	580	22.284
I14140_3	QCR7p_6	I06422_10	8	24	29	5	20	16	15	32	64	0	4	8	0	2	1	3	15	11	9	45	41	7	18	43	18	45	97	580	22.228
CYC1_4	RPOL_7	RISP_8	8	8	17	6	7	5	30	14	42	1	4	12	5	11	8	11	21	39	19	18	59	17	12	34	43	33	95	579	22.2129
P060_2	I14140_3	P169_4	13	14	38	0	0	7	25	19	45	2	4	19	1	0	8	25	8	27	30	26	72	3	4	20	49	27	103	589	22.1636
P102_3	RPOL_7	ME1ad_12	12	10	31	14	6	20	22	32	52	0	0	4	0	1	1	2	1	2	24	17	53	8	21	36	62	41	120	592	22.1409
GDHad_1	QCR9p_2	P169_4	10	6	21	14	5	19	24	14	53	3	3	3	11	1	7	8	9	21	23	17	60	12	5	36	43	40	117	585	22.1011
QCR8p_3	QCR10p_4	RISP_8	13	10	26	8	15	13	27	18	62	1	0	0	1	0	0	0	2	0	33	20	41	15	17	28	43	44	136	573	22.0997
P060_2	ME2ad_3	GOT1p2_8	17	17	26	2	0	3	22	34	42	8	2	12	2	3	2	14	28	27	32	38	51	0	6	7	43	51	109	598	22.0852
P102_3	QCR7p_6	RISP_8	6	10	39	10	11	18	30	23	47	0	0	5	1	1	0	0	1	3	33	25	55	15	14	41	46	42	115	591	22.0777
I14140_3	GOT1p2_8	QCR6p_9	14	13	30	22	18	21	28	15	58	2	1	7	4	2	12	8	3	5	22	19	34	24	14	58	40	21	95	590	22.0496
mtMDH_2	I14140_3	RPOL_7	27	7	36	5	1	2	21	15	55	7	7	14	0	4	6	9	16	36	31	27	66	6	6	16	44	24	103	591	22.0441
GDHad_1	I14140_3	CYCad_6	16	9	39	0	0	5	35	26	36	7	5	11	2	0	3	10	6	23	38	34	62	10	2	22	50	42	98	591	22.0441
QCR8p_3	RPOL_7	RISP_8	8	14	31	14	8	19	26	21	57	1	0	0	0	2	0	1	0	0	19	17	60	15	21	27	57	49	125	592	22.0433
P060_2	QCR10p_4	GOT2_8	14	5	14	12	11	13	19	22	50	10	7	10	3	0	8	12	11	33	20	17	46	12	16	27	40	27	126	585	22.0358
I34449_5	QCR7p_6	GOT1p2_8	8	8	13	3	8	21	15	20	21	15	9	28	7	7	9	15	29	24	21	26	41	12	18	26	44	48	86	582	21.9755
CYC1_4	I06422_10	ME1ad_12	8	1	10	10	17	19	19	18	37	2	1	5	8	10	30	21	12	27	9	10	19	20	17	75	46	44	97	592	21.9648
CYCad_6	GOT2_8	ME1ad_12	10	8	24	5	12	16	22	15	56	12	6	11	3	7	11	17	18	38	11	25	35	15	10	40	47	30	92	596	21.9393
I22708_3	GOT1Srg_5	GOT1p1_8	6	9	30	18	13	26	35	20	47	0	1	2	1	2	2	3	0	11	23	8	53	20	22	46	40	42	119	599	21.9339
CYCad_6	QCR6p_9	cytMDH_10	4	20	26	2	3	19	10	29	57	3	10	27	0	10	15	7	17	36	9	23	44	12	19	26	32	35	102	597	21.8763
GDHad_1	QCR10p_4	RPOL_7	8	5	33	4	5	15	27	13	53	1	3	13	6	2	4	9	9	15	28	12	37	13	10	39	51	43	103	561	21.8649
P060_2	I22708_3	ME1ad_12	11	15	33	0	2	3	31	16	50	8	6	7	1	2	3	9	18	42	24	30	67	4	0	7	54	39	112	594	21.8399
GOT2_8	QCR6p_9	ME1ad_12	10	11	22	5	12	11	18	16	37	3	12	23	3	2	13	17	15	31	23	15	47	19	6	33	43	41	104	592	21.8003
P169_4	GOT1Srg_5	CYCad_6	7	15	7	10	6	22	27	15	41	8	6	17	9	5	14	9	6	28	17	20	35	33	12	38	50	38	101	596	21.7643
I14140_3	QCR10p_4	RISP_8	13	11	24	7	17	19	32	21	71	5	0	4	2	1	1	6	8	15	30	20	39	16	16	23	33	35	113	582	21.7439
CYC1_4	GOT1p2_8	I06422_10	3	15	23	7	14	17	9	17	34	1	8	17	2	14	24	5	26	20	15	24	36	9	26	61	14	62	91	594	21.7061
P169_4	CYCad_6	GOT2_8	15	8	21	13	4	18	18	20	31	9	7	9	2	2	13	11	18	30	18	19	63	14	15	41	42	27	107	595	21.6799
QCR9p_2	QCR10p_4	GOT2_8	14	6	13	11	9	8	20	20	45	8	7	17	4	0	8	15	14	38	23	15	40	12	18	31	35	26	125	582	21.6481
QCR10p_4	GOT1Srg_5	I06422_10	0	8	21	10	11	21	11	22	43	2	15	14	2	10	15	2	20	22	7	20	43	5	33	41	25	62	98	583	21.6181
I14140_3	GOT1Srg_5	CYCad_6	8	18	26	22	8	26	31	22	59	0	0	6	5	1	11	8	1	12	23	22	27	27	14	37	46	38	98	596	21.5989
P102_3	GOT1p2_8	I06422_10	9	12	30	5	22	24	8	40	45	2	0	1	0	2	2	0	2	2	8	35	45	13	30	72	19	61	94	583	21.5626
P169_4	GOT1Srg_5	GOT1p2_8	11	5	13	5	16	16	28	20	34	5	9	17	9	7	12	7	19	18	13	19	40	24	26	33	39	57	91	593	21.5064
P060_2	ME2ad_3	RPOL_7	21	8	30	4	1	0	25	14	55	5	6	11	0	2	5	11	19	36	30	25	64	2	4	7	50	27	118	580	21.4673
QCR9p_2	I34449_5	RPOL_7	9	2	14	12	2	18	26	21	41	8	4	8	5	8	19	6	15	35	16	9	47	21	18	40	43	28	101	576	21.4671
P060_2	GOT1Srg_5	QCR7p_6	9	7	18	17	8	22	18	20	40	4	5	13	5	2	17	13	9	24	18	25	28	31	13	34	54	24	107	585	21.4625
QCR10p_4	GOT1Srg_5	GOT1p2_8	10	5	14	6	16	19	27	17	31	6	9	16	9	6	12	12	17	16	13	19	38	24	26	30	35	59	94	586	21.4557
mtMDH_2	GOT1Srg_5	QCR7p_6	9	7	19	17	8	22	19	20	44	4	4	13	5	2	17	12	9	25	18	26	27	32	13	34	54	24	102	586	21.4111
GDHad_1	I14140_3	QCR7p_6	16	7	39	0	1	4	35	25	36	7	5	11	2	0	3	10	6	23	38	29	62	10	2	22	51	38	98	580	21.3834
GDHad_1	mtMDH_2	RISP_8	13	7	17	8	5	21	23	20	54	4	4	7	5	7	3	6	9	22	23	33	59	13	11	21	46	34	113	588	21.377
GDHad_1	mtMDH_2	ME1ad_12	11	10	16	9	8	17	27	18	52	2	3	10	1	9	5	8	7	22	31	25	58	8	9	28	46	42	106	588	21.3414
ME2ad_3	P169_4	RISP_8	12	11	21	5	14	19	36	18	68	2	0	0	1	1	1	3	6	10	33	23	48	14	16	31	36	43	121	593	21.2939
QCR9p_2	GOT2_8	QCR6p_9	9	8	28	11	8	17	21	6	39	6	5	16	9	1	11	14	10	39	28	14	28	18	9	35	50	43	109	592	21.2599
P102_3	GOT1p2_8	cytMDH_10	10	16	24	6	23	22	12	24	58	0	0	3	0	3	1	0	1	3	6	30	52	21	31	66	20	37	115	584	21.2312
GDHad_1	P169_4	GOT1p2_8	11	16	21	6	4	15	25	23	45	4	6	13	4	3	6	9	11	11	28	19	29	11	27	25	42	66	107	587	21.2146
GDHad_1	QCR9p_2	I34449_5	8	6	23	7	5	27	17	22	52	4	1	5	4	6	8	9	7	22	13	25	62	10	21	22	48	49	103	586	21.191
mtMDH_2	I22708_3	RPOL_7	22	8	32	4	1	0	26	14	57	4	6	11	0	2	4	11	19	36	30	26	60	2	3	6	49	28	118	579	21.1785
I14140_3	I34449_5	CYCad_6	6	16	24	19	8	23	36	24	64	1	0	8	5	1	10	7	1	12	22	19	24	28	14	37	46	39	101	595	21.1759
GOT1Srg_5	CYCad_6	GOT2_8	8	5	19	6	5	29	14	10	35	17	7	29	7	6	10	13	23	37	18	22	46	16	10	35	44	32	97	600	21.1757
GOT1Srg_5	QCR7p_6	GOT1p1_8	9	3	19	6	3	28	14	11	34	16	8	30	7	6	9	16	23	34	19	17	49	12	13	28	46	31	95	586	21.1592
QCR9p_2	P169_4	GOT2_8	13	8	14	10	9	7	22	19	44	9	8	19	1	1	9	16	12	35	24	15	37	11	17	36	35	30	129	590	21.1509
P060_2	RPOL_7	I06422_10	7	13	29	4	9	10	8	31	45	1	4	11	4	5	17	5	21	26	9	28	45	3	30	23	23	58	107	576	21.1154
QCR7p_6	I06422_10	ME1ad_12	4	4	9	15	11	46	18	19	40	6	2	4	12	9	19	13	15	32	9	6	21	10	24	59	54	36	84	581	21.0673
GOT1Srg_5	CYCad_6	GOT1p1_8	9	3	20	6	4	31	14	11	34	16	8	30	7	6	10	16	23	34	19	18	49	13	14	34	46	31	95	601	21.0613
QCR9p_2	GOT1Srg_5	RPOL_7	8	2	16	13	3	21	26	20	35	7	5	9	4	8	17	8	14	37	24	8	47	21	20	41	36	27	100	577	21.0379

DA 16*

			Three Locus combinations (first/second/third locus genotype)																													Three Locus Totals	
first_locus	second_locus	third_locus	AA AA AA	AA AA DD	AA AA AD	AA DD AA	AA DD DD	AA DD AD	AA AD AA	AA AD DD	AA AD AD	DD AA AA	DD AA DD	DD AA AD	DD DD AA	DD DD DD	DD DD AD	DD AD AA	DD AD DD	DD AD AD	AD AA AA	AD AA DD	AD AA AD	AD DD AA	AD DD DD	AD DD AD	AD AD AA	AD AD DD	AD AD AD	Total #	chi-square value	Chromosome Combos	# p<0.05
P060_2	QCR10p_4	RISP_8	8	4	4	4	5	6	6	8	17	9	0	1	1	1	3	2	4	8	7	11	18	6	9	9	18	8	51	228	31.8499	1,2,9	1
mtMDH_2	QCR10p_4	RISP_8	8	4	5	4	6	6	6	8	17	9	0	1	1	1	3	2	4	8	7	11	18	6	8	9	18	8	51	229	31.2949	1,2,10	2
I22708_3	RPOL_7	QCR6p_9	6	2	13	3	6	12	16	10	13	2	1	0	3	0	0	0	0	7	6	3	23	6	6	11	18	12	51	230	30.4936	1,2,12	2
I14140_3	RPOL_7	QCR6p_9	7	3	13	2	6	13	17	11	14	2	1	3	5	0	0	2	1	11	5	2	20	5	6	10	15	11	46	231	30.1403	1,3,7	1
QCR10p_4	CYCad_6	RISP_8	4	1	4	11	5	5	9	9	15	2	7	7	1	2	2	8	6	9	9	2	31	2	5	10	15	13	35	229	29.463	1,4,8	1
CYC1_4	RISP_8	cytMDH_10	2	7	11	2	2	11	3	4	19	3	1	3	0	8	8	2	8	17	2	13	17	7	3	10	6	13	51	233	29.0253	1,8,12	1
CYCad_6	I06422_10	ME1ad_12	2	2	0	9	3	22	11	9	11	2	0	2	7	3	7	5	7	10	2	0	8	3	14	26	25	15	26	231	28.5179	2,3,6	1
ME2ad_3	RPOL_7	QCR6p_9	6	2	13	3	6	12	16	10	13	2	1	0	3	0	0	0	1	7	6	3	23	6	6	11	18	12	50	230	28.4121	2,3,8	2
mtMDH_2	ME2ad_3	QCR6p_9	7	0	15	2	2	0	9	6	24	1	2	6	0	0	4	4	2	10	17	16	17	3	0	3	18	13	53	234	28.1369	2,3,9	11
QCR10p_4	RISP_8	cytMDH_10	4	8	12	2	2	11	2	3	18	3	1	7	1	7	7	1	6	11	1	12	12	6	4	10	8	14	54	227	28.0195	2,4,6	3
P060_2	P169_4	RISP_8	9	5	5	3	2	7	6	10	16	6	0	0	0	1	3	4	5	9	7	11	18	5	7	8	19	10	56	232	27.7987	2,4,8	4
QCR7p_6	I06422_10	ME1ad_12	2	2	0	9	3	22	11	9	11	2	0	2	7	3	7	5	6	8	2	0	8	3	14	26	25	16	26	229	27.5112	2,6,9	3
CYC1_4	GOT2_8	QCR6p_9	2	8	7	3	0	8	8	2	23	6	1	4	4	2	7	2	9	15	10	4	18	6	4	11	20	11	39	234	26.895	2,7,10	3
I14140_3	CYC1_4	CYCad_6	2	5	15	6	3	9	12	9	25	3	0	2	5	0	0	2	1	12	4	13	18	12	4	11	24	8	30	235	26.7733	2,8,10	4
mtMDH_2	P169_4	RISP_8	9	5	6	3	3	7	6	10	16	6	0	0	0	1	3	4	5	9	7	11	18	5	6	8	19	10	56	233	26.0261	2,8,12	2
P169_4	CYCad_6	ME1ad_12	5	1	5	4	8	6	10	8	15	1	2	5	2	2	0	8	3	13	16	12	22	8	0	13	12	18	33	232	25.9567	3,4,6	6
mtMDH_2	I22708_3	QCR6p_9	7	0	15	2	1	0	9	6	24	1	2	6	0	0	4	4	2	10	17	16	17	3	0	3	18	13	54	234	25.5854	3,4,8	1
CYCad_6	GOT1p2_8	ME1ad_12	7	4	11	7	3	5	8	8	16	7	1	1	1	4	8	6	5	10	6	15	15	8	5	25	16	9	21	232	25.5131	3,4,9	3
mtMDH_2	QCR8p_3	QCR6p_9	3	0	18	1	1	0	14	7	20	1	2	7	0	0	0	3	2	13	19	14	18	0	0	0	19	15	56	233	25.2923	3,4,10	1
QCR9p_2	RISP_8	cytMDH_10	3	9	6	2	2	12	3	2	21	1	1	8	3	3	1	0	3	12	3	11	17	4	8	16	8	20	54	233	25.2738	3,5,9	3
P060_2	I22708_3	QCR6p_9	7	0	14	2	1	0	9	6	24	1	2	6	0	0	4	4	2	10	17	16	17	3	0	3	18	13	54	233	25.229	3,6,9	2
GDHad_1	QCR9p_2	QCR6p_9	2	7	10	0	1	7	15	4	20	1	1	3	4	0	2	7	3	5	10	3	23	2	3	12	19	19	49	232	25.12	3,7,8	1
P169_4	CYCad_6	RISP_8	4	1	6	10	5	3	8	10	15	2	3	3	0	1	3	6	6	12	8	7	35	3	6	12	18	12	34	233	24.9986	3,7,9	3
QCR7p_6	GOT1p2_8	ME1ad_12	7	4	11	7	3	5	8	8	16	7	1	1	1	4	6	6	4	10	6	15	15	8	5	25	16	10	21	230	24.932	3,8,10	7
P060_2	RISP_8	ME1ad_12	9	4	4	1	3	13	5	6	17	2	5	4	0	1	5	4	3	5	8	7	16	7	10	11	30	14	39	233	24.9147	4,5,8	3
QCR10p_4	RISP_8	I06422_10	0	10	14	4	5	6	3	7	14	0	4	7	0	9	5	0	8	10	3	11	12	0	6	13	8	30	37	226	24.8572	4,5,9	1
I14140_3	CYC1_4	QCR7p_6	2	5	15	6	3	9	12	8	25	3	0	2	5	0	0	2	1	12	4	11	19	12	4	11	24	8	30	233	24.6591	4,5,12	2
P060_2	QCR8p_3	QCR6p_9	3	0	17	1	1	0	14	6	20	1	2	7	0	0	0	3	2	13	19	14	18	0	0	0	19	16	56	232	24.2763	4,6,8	4
ME2ad_3	QCR10p_4	QCR6p_9	6	3	18	4	5	5	15	8	14	0	0	3	1	2	0	4	0	4	10	3	19	6	2	19	15	14	48	228	24.256	4,6,10	2
P169_4	QCR7p_6	ME1ad_12	5	1	5	4	7	5	10	9	15	1	2	5	2	2	0	8	3	13	16	12	22	8	0	12	12	18	33	230	24.2252	4,6,12	2

l14140_3	CYCad_6	QCR6p_9	7	4	9	8	6	3	11	10	28	4	1	5	1	0	0	4	1	9	3	6	31	8	1	16	15	12	32	235	24.1645	4,8,9	1
QCR10p_4	QCR7p_6	RISP_8	5	1	4	8	5	5	10	9	15	2	7	7	1	2	2	8	6	9	9	2	31	2	4	10	15	13	35	227	24.1419	4,8,10	4
l22708_3	l34449_5	QCR6p_9	4	0	8	12	3	7	9	15	22	1	1	2	1	0	4	3	0	1	9	5	14	10	2	25	12	14	49	233	24.0598	5,7,9	1
mtMDH_2	P102_3	QCR6p_9	3	0	18	0	0	2	15	8	18	2	2	5	0	0	0	2	2	15	18	14	19	1	0	1	19	15	54	233	24.0248	6,8,12	2
l14140_3	RPOL_7	GOT1p2_8	6	10	7	9	4	8	13	7	20	5	0	1	0	1	4	3	7	4	8	6	13	7	4	10	15	24	33	229	23.9208	6,10,12	2
P060_2	CYCad_6	QCR6p_9	1	0	13	9	0	9	8	7	16	0	1	4	1	0	4	4	3	12	13	10	28	7	7	6	18	13	40	234	23.8071	7,8,10	1
l14140_3	GOT1p2_8	cytMDH_10	6	6	16	5	5	11	2	9	24	0	2	6	0	4	4	0	2	7	0	14	16	5	12	20	8	5	42	231	23.655		
GDHad_1	P169_4	RISP_8	6	7	6	3	3	6	9	3	23	4	2	3	3	0	0	0	3	11	12	7	15	2	7	11	20	19	47	232	23.5249		
QCR9p_2	RPOL_7	cytMDH_10	1	5	15	5	2	2	2	6	21	0	2	2	0	1	3	4	4	14	4	11	15	2	10	22	9	18	49	229	23.4964		
l14140_3	QCR10p_4	cytMDH_10	5	3	17	4	8	5	5	8	28	0	0	3	0	0	3	0	8	9	2	10	21	1	6	17	10	14	39	226	23.402		
l22708_3	QCR10p_4	GOT2_8	10	8	9	5	1	8	12	6	18	2	1	0	2	0	0	1	2	5	5	3	23	6	9	12	17	11	50	226	23.3964		
P060_2	ME2ad_3	QCR6p_9	7	0	14	2	1	0	9	6	24	1	2	6	0	0	4	4	2	10	17	16	17	3	1	3	18	13	53	233	23.3762		
QCR10p_4	GOT1Srg_5	GOT2_8	1	0	8	2	7	11	14	5	12	2	3	5	3	1	5	8	7	10	6	2	14	14	6	19	10	11	40	226	23.2474		
GDHad_1	RISP_8	ME1ad_12	11	3	4	1	6	6	11	7	17	0	4	3	1	0	4	1	3	10	8	9	16	6	8	19	27	14	33	232	23.2409		
QCR9p_2	ME2ad_3	QCR6p_9	4	3	15	3	2	0	6	6	22	3	4	5	0	0	2	3	0	15	18	11	18	2	0	5	21	15	50	233	23.1931		
mtMDH_2	QCR10p_4	CYCad_6	1	8	8	7	3	6	7	7	17	2	4	4	1	1	3	1	1	12	6	9	21	8	1	14	34	9	34	229	23.029		
P169_4	CYCad_6	l06422_10	1	3	7	0	6	12	6	10	17	0	5	2	0	3	1	0	8	16	3	25	22	4	8	9	4	24	34	230	22.9298		
mtMDH_2	l14140_3	QCR6p_9	7	2	14	2	2	0	9	4	25	1	2	7	1	0	5	3	2	8	18	16	19	6	0	9	14	13	46	235	22.9086		
l14140_3	QCR7p_6	QCR6p_9	7	4	9	7	6	3	11	10	28	4	1	5	1	0	0	4	1	9	3	6	31	8	1	14	15	12	33	233	22.8369		
mtMDH_2	l22708_3	GOT1p2_8	10	4	8	2	0	1	8	11	20	3	1	5	2	2	0	2	9	5	19	15	14	0	1	5	21	22	42	232	22.8059		
P060_2	P102_3	QCR6p_9	3	0	17	0	0	2	15	7	18	2	2	5	0	0	0	2	2	15	18	14	19	1	0	1	19	16	54	232	22.717		
GDHad_1	mtMDH_2	ME1ad_12	7	5	5	3	0	3	13	11	19	0	0	7	0	4	2	2	3	8	8	10	21	3	5	8	30	16	39	232	22.609		
mtMDH_2	RISP_8	ME1ad_12	9	4	5	1	4	13	5	7	17	2	5	4	0	1	5	4	3	6	8	7	16	7	9	11	30	14	40	237	22.5301		
ME2ad_3	l34449_5	QCR6p_9	4	0	8	12	3	7	9	15	22	1	1	2	1	0	4	3	1	1	9	5	14	10	2	25	12	14	48	233	22.5146		
ME2ad_3	CYC1_4	CYCad_6	2	5	18	6	3	7	11	7	22	2	0	1	4	0	0	1	1	5	5	13	16	13	4	13	26	10	39	234	22.4792		
mtMDH_2	RPOL_7	l06422_10	2	6	12	3	4	2	0	14	18	0	2	2	0	2	6	0	8	9	4	12	16	1	19	10	8	26	43	229	22.4113		
CYC1_4	GOT1Srg_5	ME1ad_12	1	1	4	5	5	8	11	12	14	2	1	8	4	5	1	7	5	17	11	7	7	11	11	19	14	7	35	233	22.3906		
P060_2	l22708_3	GOT1p2_8	9	4	8	2	0	1	8	11	20	3	1	5	2	2	0	2	9	5	19	15	14	0	1	5	21	22	42	231	22.3821		
l22708_3	QCR10p_4	QCR6p_9	6	3	19	4	5	5	15	8	13	0	0	3	1	1	0	4	0	4	10	3	18	6	2	19	15	14	49	227	22.3664		
QCR9p_2	GOT1p2_8	cytMDH_10	4	9	7	3	1	11	1	3	21	0	1	6	2	3	3	2	3	11	2	12	25	5	17	21	7	10	41	231	22.3476		
GDHad_1	P060_2	ME1ad_12	7	5	5	3	0	3	13	11	19	0	0	7	0	4	2	2	3	8	8	8	21	3	5	8	30	17	39	231	22.3458		
l14140_3	l34449_5	QCR6p_9	5	1	11	11	3	7	10	16	21	2	1	3	4	0	8	3	1	3	7	4	10	8	2	21	11	13	48	234	22.2882		
l22708_3	GOT1p2_8	cytMDH_10	6	8	17	4	5	11	2	6	19	0	0	4	0	2	1	0	2	4	0	14	17	6	14	22	8	8	50	230	22.203		
QCR10p_4	l34449_5	GOT2_8	1	0	9	2	6	10	14	6	12	4	4	5	2	1	3	7	6	12	5	2	13	13	7	19	12	10	41	226	22.0812		
P060_2	QCR10p_4	CYCad_6	1	8	7	6	3	6	7	7	17	2	4	4	1	1	3	1	1	12	6	9	21	9	1	14	34	9	34	228	21.9576		
ME2ad_3	P169_4	CYCad_6	3	5	18	2	1	6	14	9	23	1	0	0	2	0	0	3	1	6	7	13	15	4	3	18	33	11	35	233	21.9258		
GDHad_1	mtMDH_2	l06422_10	3	8	5	0	4	2	6	15	22	0	1	6	0	0	6	2	8	3	2	15	21	0	6	10	5	36	44	230	21.9113		
P060_2	RPOL_7	l06422_10	2	6	11	3	4	2	0	14	18	0	2	2	0	2	6	0	8	9	4	12	16	1	19	10	8	26	43	228	21.9059		
l14140_3	RISP_8	cytMDH_10	5	3	10	4	6	10	5	11	32	0	2	7	0	1	4	0	5	6	2	16	14	5	6	15	6	9	49	233	21.9035		
GDHad_1	P060_2	l06422_10	3	8	5	0	4	2	6	15	22	0	1	6	0	0	6	2	8	3	2	15	20	0	6	10	5	36	44	229	21.858		
GDHad_1	QCR8p_3	RPOL_7	1	9	12	1	0	0	11	3	28	3	2	7	0	0	0	2	6	5	15	10	23	0	1	0	23	16	52	230	21.8431		
P060_2	GOT2_8	l06422_10	0	9	9	0	4	8	5	11	15	0	2	6	0	5	0	0	4	11	4	18	12	2	7	17	7	34	40	230	21.8233		
P169_4	l34449_5	GOT1p1_8	2	2	5	0	5	10	14	9	14	2	4	1	3	2	2	6	4	12	9	1	18	13	10	18	18	13	34	231	21.7714		
l22708_3	CYC1_4	CYCad_6	2	5	19	6	3	7	10	7	22	2	0	1	3	0	0	1	1	5	5	13	15	13	4	13	27	10	40	234	21.7609		
mtMDH_2	CYCad_6	QCR6p_9	1	1	13	9	0	9	8	7	17	0	1	4	1	0	4	4	3	12	13	9	28	7	7	6	18	13	40	235	21.7507		
RPOL_7	GOT1p2_8	cytMDH_10	1	10	7	3	7	7	2	1	18	4	3	9	1	4	4	2	6	14	1	9	21	7	10	21	6	9	41	228	21.6714		
P169_4	QCR7p_6	l06422_10	1	3	7	0	6	10	6	10	18	0	5	2	0	3	1	0	8	16	3	25	22	4	8	8	4	24	34	228	21.6442		
P060_2	QCR7p_6	QCR6p_9	1	0	13	8	0	8	8	7	17	0	1	4	1	0	3	4	3	12	13	10	28	7	7	6	18	13	40	232	21.6308		
l14140_3	QCR10p_4	QCR6p_9	7	3	15	4	5	8	15	9	17	0	0	3	1	2	0	8	0	9	9	3	22	6	2	16	11	13	40	228	21.6237		
mtMDH_2	GOT2_8	l06422_10	0	9	9	0	4	8	5	11	16	0	2	6	0	5	0	0	4	11	4	18	12	2	7	17	7	34	40	231	21.6226		
QCR9p_2	l14140_3	CYCad_6	7	7	8	4	0	4	7	9	15	4	0	10	2	0	1	3	6	6	9	10	31	4	1	9	30	10	37	234	21.5901		
l14140_3	GOT1p1_8	cytMDH_10	7	6	16	5	4	10	2	10	26	0	2	6	0	2	4	0	4	7	0	14	17	3	6	16	10	10	45	232	21.5654		
mtMDH_2	QCR10p_4	QCR7p_6	1	7	9	7	3	6	7	6	17	3	2	4	1	1	3	1	1	12	6	9	21	8	1	14	34	9	34	227	21.565		
CYC1_4	CYCad_6	RISP_8	4	1	4	7	5	6	9	9	17	1	8	14	1	3	3	5	5	10	10	2	26	5	4	9	18	14	35	235	21.5373		
CYC1_4	l34449_5	QCR6p_9	2	0	5	3	1	13	8	9	20	6	1	6	2	1	5	4	10	15	6	5	13	18	3	18	12	11	37	234	21.5005		
QCR9p_2	l22708_3	QCR6p_9	4	3	15	3	1	0	6	6	22	3	4	5	0	0	2	3	0	15	18	11	18	2	0	5	21	15	51	233	21.4934		
ME2ad_3	GOT1p1_8	cytMDH_10	8	8	17	4	4	10	2	7	21	0	0	4	0	2	2	0	2	4	0	14	18	4	6	18	10	15	52	232	21.4018		
l22708_3	GOT1p1_8	cytMDH_10	7	8	17	4	4	11	2	7	20	0	0	4	0	2	1	0	2	4	0	14	18	4	6	17	10	15	54	231	21.3993		
ME2ad_3	GOT1p2_8	cytMDH_10	6	8	17	5	5	10	2	6	20	0	0	4	0	2	2	0	2	4	0	14	17	6	14	23	8	8	48	231	21.2255		
l14140_3	P169_4	CYCad_6	4	5	15	2	1	9	14	11	25	1	0	0	2	0	1	6	1	12	6	13	18	4	3	14	30	9	27	233	21.2137		
l34449_5	RPOL_7	QCR6p_9	1	2	9	2	2	5	10	2	10	9	1	8	5	0	5	9	4	23	4	3	19	5	10	13	15	17	37	230	21.123		
CYC1_4	l34449_5	ME1ad_12	1	1	5	5	5	7	11	12	14	3	1	9	4	3	1	6	7	16	9	8	7	9	10	20	18	7	34	233	21.0802		
QCR10p_4	GOT2_8	l06422_10	2	9	6	2	6	4	3	6	23	0	8	5	0	2	8	0	11	9	2	12	16	0	6	12	9	29	34	224	21.0216		

DA 20˚

			Three Locus combinations (first/second/third locus genotype)																													Three Locus Totals	
first_locus	second_locus	third_locus	AA AA AA	AA AA DD	AA AA AD	AA DD AA	AA DD DD	AA DD AD	AA AD AA	AA AD DD	AA AD AD	DD AA AA	DD AA DD	DD AA AD	DD DD AA	DD DD DD	DD DD AD	DD AD AA	DD AD DD	DD AD AD	AD AA AA	AD AA DD	AD AA AD	AD DD AA	AD DD DD	AD DD AD	AD AD AA	AD AD DD	AD AD AD	Total #	chi-square value	Chromosome Combos	# p<0.05
QCR10p_4	GOT1Srg_5	CYCad_6	2	12	6	7	1	14	20	5	19	6	3	12	5	4	9	3	4	13	14	16	18	17	6	18	27	28	73	362	36.3794	1,2,3	2
mtMDH_2	P102_3	GOT1Srg_5	7	10	25	0	0	0	19	22	21	4	1	8	1	0	2	8	14	27	19	20	25	0	1	3	29	12	79	357	34.7178	1,2,4	1
P060_2	P102_3	GOT1Srg_5	7	10	22	0	0	0	18	22	19	4	1	8	1	0	2	9	14	27	19	20	27	0	1	3	29	11	82	356	34.3007	1,2,5	1
ME2ad_3	QCR10p_4	RISP_8	4	2	13	3	12	8	25	12	42	1	0	0	0	0	1	0	4	5	20	14	30	11	7	16	19	28	81	358	33.0724	1,2,7	1
l14140_3	P169_4	RISP_8	5	2	17	3	13	16	26	14	39	3	0	0	0	0	1	1	4	10	17	16	32	9	8	16	20	24	64	360	31.5576	1,2,10	1
l22708_3	QCR10p_4	RISP_8	4	2	13	3	12	8	24	12	41	1	0	0	0	0	1	0	3	4	20	14	30	11	7	16	21	29	82	358	30.2902	1,2,12	3
GDHad_1	QCR9p_2	RPOL_7	4	2	12	4	8	19	18	1	32	0	1	3	1	4	7	10	2	10	21	13	29	10	11	14	23	17	67	343	30.0833	1,3,6	6
CYC1_4	GOT1Srg_5	CYCad_6	3	9	6	5	1	11	20	4	20	6	4	9	4	4	13	2	5	21	13	19	21	20	7	17	29	29	65	367	29.8167	1,3,10	1
l14140_3	QCR10p_4	RISP_8	6	2	15	3	13	10	25	14	44	3	0	3	0	0	1	1	4	7	16	14	25	11	6	14	19	26	72	354	29.6724	1,4,7	1
mtMDH_2	GOT1Srg_5	CYCad_6	6	5	15	12	3	17	13	14	20	3	3	7	4	2	10	9	9	19	12	23	14	13	7	14	28	15	67	364	28.9421	1,4,8	2
QCR10p_4	GOT1Srg_5	QCR7p_6	2	9	6	7	2	13	19	4	19	5	3	12	5	4	9	3	3	13	14	15	18	17	6	18	27	24	72	349	28.6387	2,3,4	2
P060_2	GOT1Srg_5	CYCad_6	6	6	14	12	3	17	12	13	17	3	4	7	4	2	10	11	9	18	13	22	15	12	7	14	28	16	72	367	28.2471	2,3,5	11
QCR9p_2	GOT1Srg_5	RPOL_7	3	1	14	8	3	14	14	12	15	6	3	5	2	8	9	7	12	27	17	3	33	10	8	17	24	10	63	348	28.0444	2,3,7	7
P060_2	QCR8p_3	GOT1Srg_5	9	11	19	0	0	0	16	21	21	4	1	9	0	0	1	10	14	27	17	20	24	0	0	1	30	12	86	353	27.6566	2,3,8	1
CYC1_4	l34449_5	CYCad_6	3	7	4	6	1	10	19	5	22	6	1	8	2	4	11	4	7	24	11	18	20	21	8	18	30	29	67	366	27.5073	2,4,5	1
QCR10p_4	l34449_5	CYCad_6	2	9	5	7	2	13	20	6	20	6	2	9	3	4	7	5	5	18	12	15	18	19	6	19	27	29	73	361	27.3581	2,4,8	5
P169_4	QCR7p_6	GOT1p2_8	11	5	16	6	2	8	11	13	12	5	7	5	0	1	11	5	14	16	6	16	28	8	19	17	22	33	53	350	27.2194	2,4,10	2
P060_2	GOT1Srg_5	QCR7p_6	6	5	14	12	3	17	12	11	17	3	3	7	4	2	10	10	7	18	12	20	15	12	8	13	28	14	71	354	27.0002	2,5,6	6
l34449_5	GOT1p1_8	cytMDH_10	1	5	7	1	1	12	6	20	24	1	7	12	4	5	11	9	16	15	9	12	24	2	16	16	19	26	83	364	26.6802	2,5,7	4
ME2ad_3	P169_4	RISP_8	4	2	12	3	11	15	25	13	38	1	0	0	0	0	1	0	4	5	20	16	33	9	10	17	21	25	75	360	26.3349	2,5,8	4
mtMDH_2	GOT1Srg_5	QCR7p_6	6	5	15	12	3	17	12	11	20	3	2	7	4	2	10	9	7	19	12	21	14	13	8	13	29	14	66	354	26.3115	2,6,7	1
QCR9p_2	QCR10p_4	RISP_8	6	2	8	3	5	8	12	13	31	3	9	12	5	0	2	12	12	25	16	5	23	6	14	15	20	19	70	356	26.2834	3,4,6	3
P169_4	GOT1Srg_5	CYCad_6	4	10	6	7	1	13	22	7	19	7	6	12	6	3	9	5	4	14	11	16	18	15	8	19	24	25	73	364	26.1895	3,4,7	3
P060_2	GOT1Srg_5	RPOL_7	5	1	18	9	4	18	16	9	16	4	3	7	1	6	8	7	10	20	17	3	27	10	9	13	23	14	71	349	25.8521	3,4,8	10
QCR9p_2	P169_4	RISP_8	5	3	10	3	4	9	13	13	28	4	9	19	3	1	3	13	12	19	16	6	20	6	16	21	20	17	70	363	25.675	3,6,8	1
mtMDH_2	QCR8p_3	GOT1Srg_5	9	11	21	0	0	0	17	21	23	4	1	9	0	0	1	9	14	27	17	20	24	0	0	1	30	13	81	353	25.4473	3,6,10	1
GDHad_1	l14140_3	QCR7p_6	9	2	24	0	1	1	19	19	22	4	3	3	1	0	2	8	3	17	28	20	36	2	1	12	29	24	59	349	25.4078	3,7,9	2
P169_4	QCR7p_6	cytMDH_10	6	8	17	3	6	7	6	6	26	1	8	8	2	7	3	6	8	21	5	23	22	2	8	34	20	32	56	351	25.2062	3,8,9	1
QCR7p_6	RPOL_7	ME1ad_12	7	6	21	4	2	3	5	13	37	2	2	15	2	4	8	13	9	14	10	9	20	4	10	19	29	17	64	349	25.0465	3,9,10	1
QCR9p_2	l34449_5	RPOL_7	3	0	10	7	2	13	15	14	21	6	3	5	3	8	10	6	12	25	12	3	32	10	8	18	28	10	63	347	25.0426	4,5,6	8
QCR9p_2	P169_4	GOT2_8	7	3	6	5	5	5	14	14	26	7	8	14	1	0	6	11	10	23	16	7	18	5	12	26	16	15	79	359	25.0292	4,5,7	1
QCR10p_4	l34449_5	QCR6p_9	8	2	6	5	6	11	16	7	23	3	6	8	6	4	4	4	2	22	16	4	25	15	5	24	31	29	66	358	24.9657	4,5,8	1

QCR9p_2	GOT1Srg_5	CYCad_6	5	4	10	8	2	16	12	12	19	2	5	7	4	4	12	13	10	24	14	22	19	17	6	13	25	15	64	364	24.9621	4,5,9	1
ME2ad_3	P169_4	RPOL_7	8	4	6	11	3	13	16	12	46	0	0	1	0	0	1	3	4	2	10	9	48	11	7	18	33	19	61	346	24.7816	4,5,10	1
QCR10p_4	GOT1Srg_5	RISP_8	8	1	10	4	5	13	13	10	20	1	8	11	5	7	6	8	4	8	10	9	29	8	5	27	26	30	71	357	24.7634	4,6,8	3
GOT1Srg_5	CYCad_6	RPOL_7	6	2	13	10	3	16	10	2	23	12	3	12	2	3	7	6	13	21	14	4	30	8	12	14	24	18	62	350	24.6464	4,6,10	2
I14140_3	P169_4	GOT1p2_8	7	6	9	3	13	16	19	21	40	2	0	1	0	0	1	0	10	5	19	15	27	7	9	17	18	36	54	355	24.5898	4,7,8	1
ME2ad_3	QCR10p_4	GOT2_8	5	4	11	3	10	9	20	10	50	0	0	1	0	0	1	0	3	6	23	13	27	11	6	18	21	28	81	361	24.5844	4,7,12	1
QCR9p_2	ME2ad_3	P169_4	2	9	18	0	1	2	15	6	33	4	3	13	0	0	5	25	4	26	12	17	45	1	0	2	29	26	63	361	24.5539	4,8,9	1
QCR10p_4	QCR7p_6	GOT1p2_8	10	4	14	5	1	9	11	13	12	5	6	2	1	1	8	6	12	16	7	18	33	8	19	18	21	35	52	347	24.4212	4,10,12	1
GDHad_1	I22708_3	QCR7p_6	8	2	23	0	0	0	20	20	26	3	2	2	1	0	2	9	4	18	26	20	32	1	0	5	32	25	71	352	24.396	5,6,7	2
mtMDH_2	I14140_3	RPOL_7	17	3	26	3	0	1	12	11	31	5	3	10	0	3	1	8	13	27	19	14	37	2	3	8	27	10	63	357	24.3226	5,6,8	1
QCR9p_2	I14140_3	RPOL_7	14	6	16	3	0	2	9	10	28	7	6	14	0	5	1	9	12	30	20	8	41	2	1	7	28	12	66	357	24.2404	5,7,8	1
QCR9p_2	GOT1Srg_5	GOT1p1_8	6	2	11	5	9	12	9	11	23	0	0	14	5	7	8	15	6	26	10	11	34	10	5	20	18	16	70	363	24.2368	5,7,10	1
P169_4	CYCad_6	GOT1p2_8	11	6	16	6	3	9	11	13	12	5	7	6	0	1	12	5	14	16	6	16	28	9	19	21	22	33	55	362	24.1925	5,8,10	2
P169_4	GOT1Srg_5	QCR7p_6	4	9	6	7	1	13	21	6	19	6	5	12	6	3	9	5	4	14	11	14	18	15	9	18	24	21	72	352	24.099	6,7,8	1
QCR9p_2	GOT1Srg_5	QCR7p_6	5	4	10	8	2	16	13	10	18	2	4	7	4	4	12	13	8	24	14	20	19	17	7	12	24	14	64	355	24.0303	6,7,12	2
QCR9p_2	QCR10p_4	GOT2_8	8	2	5	6	5	4	12	15	29	5	7	12	3	0	4	11	11	27	15	7	21	5	11	19	17	15	80	356	23.9483	6,8,10	2
QCR9p_2	GOT1Srg_5	RISP_8	7	1	11	6	6	14	7	13	23	1	1	12	4	6	10	15	15	17	11	16	28	7	6	22	24	17	62	362	23.9472	7,8,9	1
QCR9p_2	ME2ad_3	RPOL_7	10	4	15	2	0	1	13	12	27	5	6	9	0	4	1	10	13	31	20	9	40	1	0	2	30	12	72	349	23.9169	7,8,10	1
CYC1_4	RPOL_7	ME1ad_12	5	3	11	0	2	5	14	13	22	7	1	3	2	5	6	8	6	25	6	14	41	9	10	20	22	20	67	347	23.8965	8,9,10	2
GDHad_1	I14140_3	CYCad_6	9	4	24	0	0	2	19	20	22	4	3	3	1	0	2	8	3	17	28	24	36	2	1	12	28	26	60	358	23.7831		
I34449_5	CYCad_6	RPOL_7	5	2	12	7	2	15	9	2	20	13	3	12	2	4	7	5	11	22	14	4	31	11	12	14	25	20	65	349	23.7634		
P060_2	I22708_3	GOT1Srg_5	10	13	16	1	0	1	15	19	25	4	0	8	0	0	2	10	16	28	18	20	33	1	0	4	31	13	78	366	23.6986		
I22708_3	P169_4	RISP_8	4	2	12	3	11	15	24	13	37	1	0	0	0	0	1	0	3	4	20	16	33	9	10	17	23	26	76	360	23.6287		
P169_4	CYCad_6	cytMDH_10	6	9	17	3	6	9	6	6	26	1	8	9	2	7	4	6	8	21	5	23	22	2	9	38	20	32	58	363	23.5601		
P060_2	ME2ad_3	GOT1Srg_5	10	13	16	1	0	1	15	19	25	4	0	8	0	1	2	10	15	28	19	20	34	1	0	5	30	13	78	368	23.4924		
GDHad_1	QCR9p_2	ME1ad_12	3	3	12	9	12	10	12	5	36	1	1	3	3	4	6	5	7	11	15	14	35	4	7	25	24	23	66	356	23.484		
GDHad_1	ME2ad_3	QCR7p_6	9	2	23	0	0	1	19	20	25	3	2	2	1	0	2	9	4	18	26	20	32	1	0	6	32	25	70	352	23.4451		
P060_2	I14140_3	RPOL_7	15	3	24	3	0	1	12	11	27	4	3	8	0	3	1	8	13	26	20	13	39	2	3	7	27	10	65	348	23.4306		
P169_4	GOT1Srg_5	RPOL_7	8	0	11	3	5	12	7	8	32	8	1	14	4	7	6	10	2	12	10	6	27	13	7	21	29	22	60	345	23.383		
I22708_3	P169_4	RPOL_7	8	4	6	11	3	13	16	12	44	0	0	1	0	0	1	3	3	1	10	9	48	11	7	18	33	20	64	346	23.3658		
I22708_3	QCR10p_4	GOT2_8	5	4	11	3	10	9	20	10	48	0	0	1	0	0	1	0	1	6	23	13	27	11	6	18	21	30	82	360	23.3575		
QCR9p_2	I34449_5	GOT1p1_8	3	2	8	3	8	12	14	12	26	0	1	13	7	7	8	13	5	26	10	11	28	10	5	20	18	16	75	361	23.3183		
QCR10p_4	I34449_5	cytMDH_10	3	2	11	5	4	13	7	14	25	4	6	7	3	2	9	2	8	18	1	18	25	6	21	17	21	30	77	359	23.2505		
QCR9p_2	P102_3	GOT1Srg_5	4	7	19	0	0	1	15	19	22	6	4	10	1	1	2	7	14	35	20	20	27	0	0	2	34	15	71	356	23.2263		
GOT1p2_8	QCR6p_9	I06422_10	0	10	10	5	3	10	7	6	24	2	14	17	2	9	11	11	8	36	6	20	24	1	8	16	13	34	53	360	23.0908		
GDHad_1	QCR9p_2	P169_4	4	3	11	12	3	15	13	7	32	1	1	2	8	1	4	4	8	11	12	12	40	9	3	24	23	27	64	354	23.0621		
QCR9p_2	I14140_3	P169_4	5	10	20	0	1	4	13	5	30	7	3	16	0	0	6	25	4	22	12	19	43	3	0	6	28	24	56	362	22.9778		
mtMDH_2	QCR10p_4	cytMDH_10	2	7	8	6	5	12	10	27	27	2	1	16	0	3	3	7	12	21	11	11	26	3	8	18	11	30	69	356	22.973		
P102_3	CYCad_6	cytMDH_10	3	12	19	1	10	21	13	19	20	0	2	2	0	0	2	0	1	0	8	24	27	5	12	27	19	26	84	357	22.9418		
RPOL_7	GOT1p2_8	I06422_10	2	0	12	5	5	26	4	20	18	2	4	11	2	7	8	3	8	13	8	14	21	8	18	24	12	30	61	346	22.8712		
I14140_3	GOT1p2_8	QCR6p_9	5	5	19	14	13	13	20	8	38	1	0	1	2	1	7	2	3	2	14	13	17	17	9	33	29	13	58	357	22.8507		
GDHad_1	P169_4	GOT2_8	9	11	9	3	2	8	13	8	37	4	5	5	2	3	5	4	3	11	16	3	24	6	12	24	25	26	79	357	22.8264		
QCR9p_2	I22708_3	GOT1Srg_5	6	9	14	2	0	1	11	17	28	6	3	11	0	0	3	8	17	33	19	21	32	0	0	3	36	15	68	363	22.8161		
CYC1_4	I06422_10	ME1ad_12	6	1	6	4	10	7	10	7	26	2	1	5	5	6	14	11	6	17	5	8	13	10	8	48	24	30	71	361	22.7341		
P102_3	QCR6p_9	I06422_10	3	18	19	2	6	13	9	17	31	0	1	2	2	0	0	0	0	2	5	25	30	4	14	23	21	29	77	353	22.7288		
GDHad_1	P060_2	GOT1Srg_5	3	5	10	8	5	16	13	5	36	5	2	2	0	2	8	5	4	14	18	24	30	6	9	14	32	23	61	360	22.6646		
CYCad_6	GOT1p2_8	I06422_10	3	5	14	1	10	18	9	24	17	4	2	9	1	7	15	3	16	24	5	12	21	13	14	32	8	23	54	364	22.6475		
QCR9p_2	CYC1_4	GOT2_8	9	3	7	6	8	6	11	11	26	5	7	5	4	1	6	10	10	33	10	5	24	3	10	23	23	18	77	361	22.6003		
GDHad_1	mtMDH_2	ME1ad_12	4	5	11	6	8	14	14	7	33	2	3	3	1	5	3	6	4	14	23	15	37	5	4	20	16	26	67	356	22.5415		
QCR9p_2	ME2ad_3	GOT1Srg_5	6	9	14	2	0	1	11	17	28	6	3	11	0	1	4	8	16	32	20	21	33	0	0	3	35	15	69	365	22.429		
GOT1Srg_5	CYCad_6	GOT2_8	4	5	13	5	5	21	10	5	21	8	3	17	5	3	4	5	15	20	11	14	26	8	4	26	27	21	61	367	22.4101		
mtMDH_2	I22708_3	RPOL_7	12	4	24	2	0	0	17	10	32	3	2	7	0	2	0	9	15	27	20	13	33	1	1	3	28	13	71	349	22.3271		
GOT1p1_8	QCR6p_9	I06422_10	0	11	10	5	3	10	7	7	26	1	10	12	0	3	8	5	3	25	7	24	30	3	14	18	19	38	64	363	22.2936		
GDHad_1	I22708_3	CYCad_6	8	4	23	0	0	0	20	20	27	3	3	2	1	0	2	10	4	18	26	23	32	1	0	5	32	28	72	364	22.2676		
QCR9p_2	I22708_3	RPOL_7	10	4	15	2	0	1	13	12	28	5	6	9	0	3	0	10	14	32	20	9	38	1	0	2	30	12	73	349	22.2658		
GDHad_1	P060_2	ME1ad_12	3	4	11	6	9	14	15	6	33	2	3	3	1	4	4	6	5	12	22	13	37	5	4	20	17	27	70	356	22.2051		
I22708_3	RPOL_7	QCR6p_9	11	6	18	10	1	8	17	12	34	1	0	2	0	2	1	1	0	2	8	11	35	12	3	23	42	28	60	348	22.1501		
QCR7p_6	GOT1p2_8	I06422_10	3	5	14	1	10	17	9	24	16	4	2	8	1	7	14	3	14	19	5	12	21	13	14	32	8	23	52	351	22.1364		
QCR9p_2	ME2ad_3	RISP_8	8	10	11	0	0	3	12	10	34	9	3	8	0	3	2	11	16	29	14	13	46	1	1	1	27	25	66	363	22.123		
mtMDH_2	I22708_3	GOT1Srg_5	9	13	19	1	0	1	16	19	27	4	0	8	0	0	2	9	16	27	18	20	32	1	0	4	30	14	73	363	22.0303		
RPOL_7	GOT1p2_8	QCR6p_9	1	7	6	8	5	23	11	5	26	6	1	10	7	1	9	8	4	13	13	9	21	17	16	18	29	15	56	345	22.0021		
P102_3	P169_4	CYCad_6	8	4	10	10	6	11	16	20	31	3	0	0	1	0	0	0	2	1	20	14	28	5	7	23	34	23	77	354	21.9893		
GOT1Srg_5	RPOL_7	RISP_8	1	7	18	1	0	6	16	10	26	2	2	16	4	8	6	10	7	23	10	9	26	8	12	14	29	23	55	349	21.9063		
GDHad_1	P102_3	I06422_10	8	9	12	0	0	3	7	22	39	1	4	4	0	1	0	5	7	16	5	28	46	2	0	1	18	39	74	351	21.8832		
P102_3	QCR7p_6	RISP_8	5	7	23	5	8	13	17	12	24	0	0	4	1	1	0	0	0	1	17	17	28	10	6	28	26	26	77	356	21.8665		
P060_2	QCR10p_4	cytMDH_10	2	7	9	6	5	11	9	27	23	2	2	15	0	3	3	7	13	22	11	11	26	3	8	20	12	29	73	359	21.8615		
mtMDH_2	ME2ad_3	GOT1Srg_5	9	13	19	1	0	1	16	19	27	4	0	8	0	1	2	9	15	27	19	20	33	1	0	5	29	14	73	365	21.831		
QCR9p_2	I34449_5	GOT1p2_8	3	4	6	3	9	11	12	16	23	0	5	9	7	10	5	13	9	22	10	18	21	10	6	19	16	33	59	359	21.829		
I14140_3	P169_4	RPOL_7	8	5	11	13	3	14	19	12	46	0	0	3	0	0	1	5	6	5	11	8	45	9	7	17	27	17	57	349	21.7717		
P060_2	I22708_3	RPOL_7	12	4	22	2	0	0	16	10	30	3	2	7	0	2	0	9	15	28	20	12	35	1	1	3	29	13	74	350	21.7398		
CYC1_4	RISP_8	QCR6p_9	8	2	14	4	1	10	10	9	18	2	1	8	10	1	11	10	9	15	10	10	26	13	13	17	38	19	70	359	21.6199		
GDHad_1	P169_4	RPOL_7	4	3	22	4	2	7	18	5	33	1	1	10	6	1	3	4	5	6	13	8	22	12	7	21	30	25	67	340	21.6029		
P169_4	RPOL_7	GOT2_8	5	4	9	5	1	7	20	13	21	2	4	15	4	3	3	5	9	18	8	14	30	10	10	15	24	12	74	345	21.5669		
I14140_3	P169_4	GOT2_8	7	5	10	3	10	18	20	12	48	2	0	1	0	0	1	0	4	12	21	13	27	8	7	18	22	23	64	356	21.5632		
P060_2	I34449_5	RPOL_7	5	1	16	8	3	17	17	10	19	5	3	7	1	6	8	6	10	19	11	2	24	11	9	15	27	15	73	348	21.5607		
GDHad_1	P060_2	cytMDH_10	4	7	7	2	6	21	10	12	32	2	4	3	3	4	3	5	3	15	11	28	32	4	9	16	11	35	70	359	21.544		
mtMDH_2	P169_4	I34449_5	3	6	9	10	4	8	10	19	33	1	6	14	3	3	2	9	7	19	13	10	24	6	6	23	21	19	68	356	21.5239		
P102_3	QCR10p_4	QCR7p_6	8	2	10	6	3	12	19	20	31	3	0	0	1	0	0	0	2	1	16	13	28	5	6	21	39	21	75	342	21.5214		
GDHad_1	QCR10p_4	GOT1p2_8	6	9	11	2	3	5	12	19	33	0	3	9	3	2	4	6	7	8	19	8	18	7	14	19	19	44	65	355	21.5079		
QCR7p_6	RPOL_7	RISP_8	5	8	21	0	3	6	17	13	26	1	5	13	6	2	6	8	8	20	6	6	26	7	13	12	30	20	61	349	21.3467		
I34449_5	RPOL_7	cytMDH_10	3	6	12	1	0	5	3	20	23	1	8	11	1	7	10	11	13	17	7	18	25	8	6	22	14	27	68	347	21.3338		
P102_3	P169_4	QCR7p_6	10	3	10	10	5	11	16	17	31	3	0	0	1	0	0	0	2	1	21	14	29	5	7	23	34	22	75	350	21.3174		
QCR9p_2	CYCad_6	RPOL_7	10	1	14	3	6	6	12	9	24	6	3	8	3	9	7	6	11	26	16	5	33	14	3	23	21	13	57	349	21.31		
GDHad_1	ME2ad_3	CYCad_6	9	4	23	0	0	1	19	20	26	3	3	2	1	0	2	10	4	18	26	23	32	1	0	6	32	28	71	364	21.2555		
CYC1_4	GOT1Srg_5	QCR7p_6	3	6	6	5	2	10	18	4	20	5	4	9	4	4	13	2	5	21	13	18	21	20	7	17	30	23	64	354	21.2344		
CYCad_6	RPOL_7	ME1ad_12	5	6	21	4	2	3	5	12	37	3	3	14	3	5	10	11	11	15	10	9	21	4	10	19	28	16	62	349	21.1616		
I34449_5	GOT1p2_8	cytMDH_10	1	5	7	2	9	16	5	12	20	1	7	12	6	5	14	6	16	13	8	12	21	9	23	27	13	19	73	362	21.0898		
ME2ad_3	RPOL_7	QCR6p_9	11	6	18	10	1	8	17	12	36	1	0	2	0	2	2	2	0	2	8	11	35	12	3	22	41	28	58	348	21.0768		
GDHad_1	mtMDH_2	QCR8p_3	8	0	12	5	0	23	14	0	39	1	0	6	3	1	4	5	1	17	32	0	41	6	0	23	42	0	64	347	21.0525		
GDHad_1	P060_2	QCR8p_3	7	0	11	5	0	24	14	0	39	1	0	6	3	1	4	5	1	16	31	0	40	6	0	23	42	0	69	348	21.0347		

DA females

			Three Locus combinations (first/second/third locus genotype)																													Three Locus Totals	
first_locus	second_locus	third_locus	AA AA AA	AA AA DD	AA AA AD	AA DD AA	AA DD DD	AA DD AD	AA AD AA	AA AD DD	AA AD AD	DD AA AA	DD AA DD	DD AA AD	DD DD AA	DD DD DD	DD DD AD	DD AD AA	DD AD DD	DD AD AD	AD AA AA	AD AA DD	AD AA AD	AD DD AA	AD DD DD	AD DD AD	AD AD AA	AD AD DD	AD AD AD	Total #	chi-square value		Chromosome Combos	# p<0.05
CYC1_4	RISP_8	cytMDH_10	5	13	24	4	5	16	7	13	37	6	2	7	3	15	16	4	20	32	10	26	36	12	13	27	9	48	107	517	29.5594	4,8,10	1,4,10	1
P169_4	QCR7p_6	GOT1p2_8	15	6	21	9	7	12	16	23	19	7	10	6	1	2	11	12	20	18	19	25	47	6	23	21	40	37	71	504	28.9662	4,6,8	1,4,8	3
GOT2_8	QCR6p_9	ME1ad_12	10	9	20	4	12	9	16	13	34	3	11	22	2	1	12	15	12	23	20	9	39	17	6	32	38	33	93	515	28.5888	8,9,12	1,6,10	2
QCR7p_6	GOT2_8	ME1ad_12	10	8	23	5	12	14	20	11	53	11	4	5	3	6	9	14	11	28	9	21	34	12	5	33	40	26	81	508	28.3315	6,8,12	1,7,10	1
CYCad_6	GOT2_8	ME1ad_12	10	8	23	5	12	14	20	11	54	11	5	6	3	7	10	16	13	30	9	21	34	12	5	33	40	25	82	519	27.5401	6,8,12	1,8,10	1

GDHad_1	QCR10p_4	RISP_8	15	11	13	8	6	8	18	7	53	6	5	6	5	2	4	3	11	19	21	12	39	11	21	24	41	35	98	502	27.2477	1,4,8	2,10,12	1
P102_3	CYC1_4	RPOL_7	13	3	26	3	9	21	30	23	44	1	0	1	3	0	0	0	2	2	17	13	49	10	14	41	50	27	103	505	27.135	3,4,7	2,3,12	6
QCR10p_4	RISP_8	X06422_10	2	20	24	5	6	17	10	14	34	0	11	13	1	16	11	1	15	21	6	20	36	2	20	29	14	67	92	507	27.1114	4,8,10	2,3,8	2
QCR9p_2	GOT2_8	QCR6p_9	8	7	27	11	8	14	19	6	31	5	4	13	8	0	9	11	10	31	26	13	24	17	7	27	38	40	101	515	27.0113	2,8,9	2,4,10	1
P169_4	X34449_5	GOT2_8	9	3	13	4	10	19	30	15	28	5	7	10	5	7	4	10	10	29	10	8	43	21	13	39	33	29	103	517	26.907	4,5,8	2,4,7	1
QCR10p_4	GOT1Srg_5	CYCad_6	3	16	8	12	6	19	22	12	34	9	3	14	9	4	9	11	6	26	18	13	28	30	8	31	44	31	87	513	26.7425	4,5,6	2,4,8	6
QCR10p_4	X34449_5	GOT2_8	7	3	15	5	8	22	30	13	26	7	7	12	5	6	4	13	11	25	10	8	38	21	15	36	30	27	107	511	26.5588	4,5,8	2,5,7	1
CYC1_4	GOT2_8	QCR6p_9	10	10	18	7	2	13	13	10	40	10	3	8	13	3	14	5	17	33	18	12	38	16	10	23	51	30	92	519	26.4089	4,8,9	2,5,8	3
mtMDH_2	ME2ad_3	ME1ad_12	8	16	29	0	3	3	32	16	46	7	5	7	1	2	3	5	14	33	22	25	62	4	0	7	48	26	95	519	26.2861	2,3,12	2,7,10	3
GDHad_1	QCR7p_6	cytMDH_10	9	12	24	5	7	13	4	16	48	3	5	11	1	3	6	9	7	17	3	32	58	1	21	35	24	51	79	504	26.2454	1,6,10	2,7,8	2
CYCad_6	GOT1p1_8	ME1ad_12	10	8	23	6	6	15	19	17	54	9	4	6	2	9	12	20	12	28	14	25	33	15	4	33	32	23	81	520	26.1788	6,8,12	2,8,10	1
CYC1_4	RISP_8	ME1ad_12	11	15	16	4	7	14	20	10	28	4	2	9	4	7	23	20	12	24	17	15	39	11	14	28	35	26	103	518	26.1606	4,8,12	2,8,9	2
GDHad_1	P169_4	GOT2_8	11	14	16	7	3	10	24	10	44	7	5	9	5	2	5	6	4	20	24	9	36	8	18	28	34	35	119	513	26.0691	1,4,8	3,4,6	3
P169_4	CYCad_6	GOT1p2_8	15	7	21	9	9	14	16	23	18	7	10	7	1	2	11	12	20	18	19	25	47	7	24	25	40	37	73	517	26.0048	4,6,8	3,4,7	1
X34449_5	GOT1p2_8	cytMDH_10	2	11	12	3	11	18	4	18	28	1	13	19	5	9	24	7	20	26	11	20	36	13	34	39	12	21	100	517	25.9756	5,8,10	3,4,8	4
QCR8p_3	RISP_8	QCR6p_9	6	7	30	13	3	17	37	21	38	1	1	0	1	1	0	0	0	0	25	12	44	24	14	37	35	36	104	507	25.9187	3,8,9	3,5,12	1
GDHad_1	CYCad_6	cytMDH_10	9	12	24	5	7	14	4	16	49	3	6	11	1	3	7	9	7	17	3	32	58	2	24	40	24	50	80	517	25.795	1,6,10	3,7,10	1
X34449_5	GOT1p1_8	cytMDH_10	2	11	12	2	3	14	5	26	32	1	13	19	4	7	20	9	22	29	12	20	40	5	22	26	19	32	112	519	25.7462	5,8,10	3,7,12	1
mtMDH_2	X22708_3	ME1ad_12	8	17	29	0	2	3	32	15	46	7	5	7	1	2	2	5	14	34	22	25	60	3	0	7	48	27	97	518	25.5301	2,3,12	3,7,8	2
GOT1p1_8	QCR6p_9	ME1ad_12	10	8	17	7	12	11	16	17	34	2	8	26	4	1	12	17	10	22	21	13	38	13	6	29	36	32	95	517	25.446	8,9,12	3,8,10	2
QCR10p_4	RISP_8	cytMDH_10	9	13	24	7	4	17	6	13	38	6	4	14	3	10	16	2	14	21	7	24	30	9	18	25	12	50	111	507	25.4009	4,8,10	3,8,9	2
mtMDH_2	RPOL_7	GOT2_8	15	10	20	5	5	10	24	18	41	1	5	6	7	1	12	13	8	21	13	11	46	16	15	20	33	21	105	502	25.2953	2,7,8	4,10,12	1
QCR7p_6	GOT1p1_8	ME1ad_12	10	8	23	6	6	15	19	17	53	9	4	5	2	8	11	18	9	25	14	25	33	15	4	33	32	24	81	509	25.2284	6,8,12	4,5,6	2
X14140_3	P169_4	GOT2_8	13	10	15	5	11	20	30	16	71	3	0	1	2	1	0	4	8	19	27	17	45	13	12	23	30	26	90	512	24.8394	3,4,8	4,5,8	5
P060_2	RPOL_7	GOT2_8	15	10	18	5	5	10	23	17	38	1	5	6	7	1	12	12	8	22	13	11	47	15	15	20	35	23	108	502	24.776	2,7,8	4,6,8	4
X14140_3	P169_4	RISP_8	10	9	22	5	13	19	31	18	67	4	0	0	2	1	0	6	8	16	30	22	41	12	12	24	30	30	85	517	24.7442	3,4,8	4,8,10	6
QCR10p_4	X34449_5	CYCad_6	3	14	8	12	6	18	22	13	35	9	3	14	5	4	6	15	6	29	17	13	26	31	8	33	44	31	88	513	24.6733	4,5,6	4,8,12	1
P060_2	X22708_3	ME1ad_12	7	14	29	0	2	3	31	14	45	7	5	7	1	2	2	5	13	35	23	25	60	3	0	7	49	29	99	517	24.5394	2,3,12	4,8,9	1
GDHad_1	P169_4	RISP_8	14	12	15	6	5	9	21	7	50	7	6	7	5	3	4	2	10	17	21	13	37	8	18	28	44	39	102	510	24.5254	1,4,8	5,6,8	2
QCR9p_2	QCR10p_4	GOT2_8	14	5	11	10	8	7	18	19	38	5	5	15	4	0	6	13	12	31	23	13	37	11	16	27	29	19	110	506	24.5223	2,4,8	5,8,10	2
CYC1_4	GOT2_8	X06422_10	3	16	19	0	11	11	12	17	35	0	7	14	0	10	19	2	28	25	8	26	34	1	14	33	15	65	95	520	24.3403	4,8,10	6,8,12	4
mtMDH_2	QCR10p_4	RISP_8	14	6	13	8	11	15	16	21	49	12	6	5	3	2	4	9	11	24	20	16	40	13	16	18	37	20	99	508	24.1863	2,4,8	8,9,12	3
QCR9p_2	P169_4	GOT2_8	13	7	11	10	8	6	19	18	38	6	6	17	1	1	6	14	10	30	24	14	33	9	15	31	30	22	115	514	24.1588	2,4,8		
ME2ad_3	QCR10p_4	RISP_8	13	9	20	7	12	13	28	12	62	3	0	0	2	1	0	2	6	9	30	19	38	15	16	24	31	35	104	511	24.1098	3,4,8		
QCR8p_3	RPOL_7	RISP_8	8	11	26	13	7	17	22	15	54	1	0	0	0	2	0	1	0	0	16	15	53	14	19	18	53	43	107	515	24.0987	3,7,8		
QCR9p_2	RPOL_7	X06422_10	2	13	27	1	8	12	5	24	34	2	5	8	4	6	14	4	22	24	8	23	38	1	30	16	13	57	99	500	23.9768	2,7,10		
X22708_3	QCR10p_4	RISP_8	12	9	21	7	12	13	27	12	61	3	0	0	2	0	0	2	5	8	30	19	37	15	16	24	33	36	105	509	23.9664	3,4,8		
QCR9p_2	X34449_5	RISP_8	10	4	10	4	9	17	23	18	38	2	2	14	11	8	8	12	16	20	11	16	37	17	12	38	38	28	94	517	23.7927	2,5,8		
QCR9p_2	X34449_5	GOT1p1_8	8	3	13	4	9	17	26	20	33	1	1	16	9	7	11	12	5	31	16	15	33	20	15	32	35	27	98	517	23.7256	2,5,8		
X14140_3	RPOL_7	GOT1p1_8	10	17	27	13	6	17	27	14	58	5	1	4	0	2	8	6	5	8	16	12	33	17	11	18	37	28	102	502	23.5855	3,7,8		
P102_3	GOT1p1_8	X06422_10	6	12	29	1	14	17	6	46	42	1	0	0	0	1	1	0	3	3	6	38	42	2	14	47	18	67	96	512	23.5193	3,8,10		
QCR9p_2	ME2ad_3	ME1ad_12	7	11	24	0	3	4	26	16	41	10	7	11	2	1	4	7	16	35	19	27	63	3	1	5	52	24	100	519	23.5106	2,3,12		
P060_2	QCR10p_4	RISP_8	14	6	12	8	10	14	14	21	46	11	6	5	3	2	4	9	11	25	21	16	39	13	17	19	39	21	102	508	23.5091	2,4,8		
X22708_3	X34449_5	ME1ad_12	4	8	23	6	16	23	27	22	49	0	3	2	2	0	2	2	1	8	22	8	36	22	17	37	40	31	106	517	23.4995	3,5,12		
GDHad_1	RPOL_7	X06422_10	7	11	17	3	6	8	7	31	47	1	4	10	0	3	10	5	13	12	4	26	47	3	34	24	10	58	96	497	23.4903	1,7,10		
QCR10p_4	GOT2_8	X06422_10	4	17	21	1	7	16	12	14	38	0	12	13	0	8	15	2	22	17	7	21	33	0	18	30	15	70	95	508	23.4082	4,8,10		
mtMDH_2	QCR8p_3	ME1ad_12	9	14	30	0	1	1	33	18	47	8	6	10	1	0	0	5	16	35	22	22	53	0	0	1	52	28	109	521	23.373	2,3,12		
GOT1p2_8	QCR6p_9	ME1ad_12	10	8	16	7	12	11	16	15	31	5	11	31	6	2	23	23	15	39	17	10	32	11	5	19	30	28	80	513	23.3236	8,9,12		
GDHad_1	CYC1_4	X06422_10	3	8	22	0	14	11	14	28	38	4	6	6	1	4	9	2	10	22	8	30	37	1	27	37	8	67	99	516	23.3221	1,4,10		
P169_4	QCR7p_6	GOT2_8	15	7	20	11	3	13	17	16	26	8	7	7	2	2	10	10	15	25	18	17	57	7	13	30	37	19	94	506	23.2291	4,6,8		
X14140_3	GOT1p2_8	QCR6p_9	11	12	26	22	16	17	22	14	52	2	1	7	3	2	9	8	3	4	21	17	30	22	13	50	30	19	80	513	23.157	3,8,9		
mtMDH_2	RPOL_7	X06422_10	3	13	29	1	9	10	6	31	44	1	4	8	3	4	14	3	19	20	8	25	37	2	31	18	13	52	95	503	23.0731	2,7,10		
CYC1_4	X34449_5	GOT2_8	5	2	13	4	6	19	29	14	31	5	9	13	5	7	9	11	14	32	14	7	40	22	17	35	32	25	102	522	23.0384	4,5,8		
QCR9p_2	GOT1Srg_5	GOT1p1_8	9	2	13	6	10	18	23	20	32	1	0	17	7	7	10	14	6	31	17	14	39	22	13	36	32	30	89	518	23.0216	2,5,8		
P060_2	ME2ad_3	GOT1p1_8	14	8	28	2	0	3	23	26	43	8	1	11	2	2	2	10	12	31	29	27	54	0	3	9	43	24	108	523	23.001	2,3,8		
QCR9p_2	P169_4	cytMDH_10	4	11	16	3	11	11	11	20	44	4	6	20	0	5	3	7	12	35	14	15	42	8	15	32	8	61	96	514	22.9849	2,4,10		
X22708_3	GOT2_8	cytMDH_10	9	12	28	4	6	24	10	37	47	0	0	5	0	2	1	3	2	7	9	29	36	9	20	35	16	48	120	519	22.7708	3,8,10		
P102_3	RPOL_7	ME1ad_12	11	6	29	13	5	17	20	27	46	0	0	4	0	1	1	1	0	2	22	15	44	7	17	31	54	34	109	516	22.7338	3,7,12		
QCR9p_2	QCR10p_4	RISP_8	13	6	12	7	9	10	18	16	41	7	10	9	5	1	4	13	14	29	25	12	37	12	19	23	29	23	103	507	22.7065	2,4,8		
QCR10p_4	QCR7p_6	GOT1p2_8	13	5	19	9	6	14	18	22	19	9	10	9	1	2	9	15	17	17	20	25	46	6	22	19	35	41	69	497	22.6525	4,6,8		
QCR9p_2	X34449_5	RPOL_7	9	2	13	10	2	17	24	17	35	7	4	6	4	7	15	4	13	29	11	9	42	18	16	34	39	22	92	501	22.6477	2,5,7		
P060_2	X22708_3	GOT1p2_8	14	15	22	2	0	3	21	31	39	7	1	11	2	1	2	10	24	20	28	36	42	0	4	6	41	43	93	518	22.6243	2,3,8		
QCR8p_3	RPOL_7	cytMDH_10	4	14	26	8	8	21	11	35	43	0	0	1	1	0	1	0	0	1	9	31	42	1	18	33	23	48	121	500	22.6086	3,7,10		
P060_2	GOT2_8	cytMDH_10	7	21	15	5	8	21	6	23	37	2	4	14	0	8	7	6	12	24	9	16	39	8	12	33	17	52	113	519	22.5428	2,8,10		
P060_2	cytMDH_10	ME1ad_12	2	4	12	17	14	22	19	12	42	4	1	3	2	5	16	7	14	25	12	5	17	13	15	52	51	35	96	517	22.4982	2,10,12		
QCR9p_2	CYC1_4	RPOL_7	9	4	18	6	6	19	28	11	28	1	5	16	3	2	7	11	17	27	21	7	42	7	15	36	40	24	91	501	22.4688	2,4,7		
P169_4	GOT1Srg_5	GOT2_8	11	3	12	5	10	18	27	15	30	6	5	14	7	8	7	7	11	23	9	9	44	20	11	40	35	30	100	517	22.4579	4,5,8		
mtMDH_2	QCR10p_4	GOT2_8	14	5	13	11	9	13	19	21	46	9	3	9	3	0	6	9	10	25	19	15	41	11	15	21	33	18	108	506	22.4018	2,4,8		
P060_2	RPOL_7	X06422_10	3	12	28	1	9	10	6	30	41	1	4	8	3	4	14	3	19	20	8	26	37	2	30	18	13	54	98	502	22.3971	2,7,10		
X14140_3	P169_4	CYCad_6	11	10	18	9	4	24	37	27	53	3	0	1	2	0	1	7	2	22	28	22	41	12	10	25	48	26	72	515	22.3555	3,4,6		
X14140_3	CYC1_4	CYCad_6	9	11	21	10	5	21	38	25	54	4	1	5	5	0	0	4	1	20	20	16	38	19	11	34	50	32	66	520	22.2905	3,4,6		
X14140_3	CYC1_4	QCR7p_6	9	9	21	10	5	21	38	21	54	4	2	4	5	0	0	4	1	20	19	14	39	19	11	34	51	29	65	509	22.2254	3,4,6		
CYC1_4	RISP_8	X06422_10	1	18	23	3	9	13	11	18	29	0	5	10	1	17	15	1	23	32	7	26	40	4	17	31	13	60	91	518	22.1952	4,8,10		
P060_2	ME2ad_3	ME1ad_12	7	13	29	0	2	3	31	15	45	7	5	7	1	2	3	5	13	34	23	25	62	4	1	7	49	28	97	518	22.1934	2,3,12		
CYC1_4	X06422_10	ME1ad_12	6	1	8	10	17	18	19	14	32	1	0	1	8	8	29	19	12	26	5	4	15	18	15	71	41	36	83	517	22.1932	4,10,12		
QCR9p_2	GOT1p1_8	QCR6p_9	6	8	25	10	8	14	22	6	34	4	6	12	7	0	6	13	8	36	25	16	31	19	9	29	37	34	92	517	22.1549	2,8,9		
X34449_5	QCR7p_6	GOT1p1_8	8	3	17	3	4	20	14	11	24	14	7	28	4	5	9	15	19	22	20	17	45	10	12	22	42	22	92	509	22.1028	5,6,8		
GDHad_1	RISP_8	X06422_10	6	12	23	2	9	13	9	29	36	0	6	8	5	5	9	2	9	18	2	30	41	1	29	37	14	63	93	511	22.0985	1,8,10		
QCR10p_4	GOT1Srg_5	RISP_8	10	3	13	10	10	17	25	15	27	2	9	14	7	9	6	15	11	17	12	11	36	16	10	42	33	32	96	508	22.0886	4,5,8		
X34449_5	CYCad_6	GOT1p1_8	8	3	18	3	5	22	14	11	24	14	7	28	4	5	10	15	19	22	20	18	45	11	13	28	42	22	92	523	22.0684	5,6,8		

DA males

			Three Locus combinations (first/second/third locus genotype)																														Three Locus Totals	
first_locus	second_locus	third_locus	AA AA AA	AA AA DD	AA AA AD	AA DD AA	AA DD DD	AA DD AD	AA AD AA	AA AD DD	AA AD AD	DD AA AA	DD AA DD	DD AA AD	DD DD AA	DD DD DD	DD DD AD	DD AD AA	DD AD DD	DD AD AD	AD AA AA	AD AA DD	AD AA AD	AD DD AA	AD DD DD	AD DD AD	AD AD AA	AD AD DD	AD AD AD	Total #	chi-square value		Chromosome Combos	# p<0.05
mtMDH_2	ME2ad_3	QCR10p_4	0	2	8	0	0	0	1	3	2	0	0	2	1	0	0	5	2	8	5	3	4	0	1	0	3	1	25	76	28.0357	2,3,4	1,4,10	1
P060_2	ME2ad_3	QCR10p_4	0	2	8	0	0	0	1	3	2	0	0	2	1	0	0	5	2	9	5	3	4	0	1	0	3	1	24	76	27.5201	2,3,4	1,4,7	1
QCR10p_4	X06422_10	ME1ad_12	1	0	3	0	1	1	0	5	4	1	1	2	0	3	0	4	0	1	5	5	5	2	0	5	3	7	15	74	25.8363	4,10,12	1,4,8	1
QCR9p_2	P060_2	QCR7p_6	2	3	6	0	0	0	0	2	3	0	0	0	1	4	9	0	1	4	0	2	3	1	0	3	7	9	16	76	25.8021	2,2,6	2,3,4	6
mtMDH_2	X22708_3	QCR10p_4	0	2	7	0	0	0	1	3	3	0	0	2	1	0	0	5	2	8	5	3	4	0	1	0	3	1	25	76	25.7336	2,3,4	2,3,5	5
P060_2	X22708_3	QCR10p_4	0	2	7	0	0	0	1	3	3	0	0	2	1	0	0	5	2	9	5	3	4	0	1	0	3	1	24	76	25.2271	2,3,4	2,4,12	2
mtMDH_2	X14140_3	GOT1Srg_5	2	2	5	0	0	0	3	2	2	1	0	1	0	3	0	1	3	9	1	6	9	2	0	0	8	5	12	77	24.903	2,3,5	3,7,10	1
QCR10p_4	CYCad_6	cytMDH_10	0	0	1	1	1	2	0	1	9	0	1	0	1	1	1	2	0	6	1	2	5	0	1	14	14	3	9	76	24.2419	4,6,10	4,10,12	2
QCR10p_4	GOT1Srg_5	cytMDH_10	1	1	0	0	0	5	0	1	7	2	0	3	1	1	3	0	1	1	0	1	10	5	3	3	10	2	15	76	24.1317	4,5,10	4,5,10	4
P169_4	X06422_10	ME1ad_12	1	0	4	0	1	0	1	5	4	1	2	1	0	3	0	4	2	1	5	5	5	2	0	6	2	5	15	75	24.009	4,10,12	4,5,12	1
CYCad_6	GOT1p2_8	X06422_10	0	0	2	0	0	2	3	3	1	4	0	3	0	0	1	1	4	8	2	0	4	7	4	6	7	1	12	75	23.9923	6,8,10	4,6,10	2
QCR7p_6	GOT1p2_8	X06422_10	0	0	2	0	0	2	3	3	1	4	0	3	0	0	1	1	4	7	2	0	4	7	4	6	7	1	12	74	23.6915	6,8,10	5,6,8	2
QCR10p_4	QCR7p_6	cytMDH_10	0	0	1	1	1	2	0	1	9	0	1	0	1	1	1	2	0	6	1	2	5	0	1	13	14	3	9	75	23.5773	4,6,10	6,8,10	2
mtMDH_2	X14140_3	QCR10p_4	0	2	7	0	0	0	1	3	3	0	0	2	1	0	2	5	2	6	6	3	6	0	1	1	2	1	22	76	23.55	2,3,4	6,9,10	1
P060_2	X14140_3	GOT1Srg_5	2	2	5	0	0	0	3	2	2	1	0	1	0	3	0	2	3	9	1	6	9	2	0	0	7	5	12	77	23.5138	2,3,5		

CYC1_4	X34449_5	X06422_10	0	0	1	3	1	0	0	0	8	0	1	0	2	0	2	4	2	1	2	4	6	3	0	9	9	4	12	74	23.2689	4,5,10
X34449_5	CYCad_6	GOT1p2_8	0	1	0	0	0	6	1	4	2	1	0	2	3	1	0	0	7	6	1	1	5	4	0	8	5	7	11	76	23.2316	5,6,8
X14140_3	RPOL_7	cytMDH_10	1	5	2	1	0	4	4	2	8	0	0	0	1	0	0	0	2	2	2	0	10	5	0	4	5	1	17	76	23.2263	3,7,10
QCR9p_2	ME2ad_3	GOT1Srg_5	0	2	7	1	0	0	2	2	2	2	0	2	0	0	0	2	6	8	3	6	3	0	1	0	8	4	16	77	23.22	2,3,5
GDHad_1	QCR10p_4	RPOL_7	0	1	6	0	0	2	3	5	9	0	0	2	1	0	0	0	2	0	3	3	0	4	0	5	9	4	15	74	23.1031	1,4,7
P060_2	X14140_3	QCR10p_4	0	2	7	0	0	0	1	3	3	0	0	2	1	0	2	5	2	7	6	3	6	0	1	1	2	1	21	76	22.7937	2,3,4
QCR10p_4	X34449_5	ME1ad_12	0	0	1	0	0	4	1	6	2	1	3	0	2	0	3	2	1	0	3	2	4	3	3	5	4	8	17	75	22.6653	4,5,12
mtMDH_2	X14140_3	X34449_5	2	2	5	0	0	0	3	2	2	1	0	1	0	3	0	1	3	8	0	5	11	1	0	1	6	5	14	76	22.6309	2,3,5
GDHad_1	P169_4	X06422_10	3	0	4	2	1	2	2	8	5	1	0	1	0	1	0	1	0	1	1	1	5	2	1	5	12	0	16	75	22.4744	1,4,10
QCR9p_2	P169_4	ME1ad_12	1	1	2	2	0	0	0	1	9	0	4	3	2	1	0	2	4	3	1	1	3	1	6	2	7	6	14	76	22.2709	2,4,12
QCR9p_2	QCR10p_4	ME1ad_12	1	1	1	2	0	1	0	1	9	0	4	3	2	0	0	2	5	4	0	1	4	1	4	2	8	7	13	76	22.1725	2,4,12
X34449_5	QCR7p_6	GOT1p2_8	0	1	0	0	0	5	1	4	2	1	0	2	3	1	0	0	7	6	1	1	5	4	0	8	5	7	11	75	21.9221	5,6,8
QCR10p_4	X34449_5	cytMDH_10	1	0	0	0	0	4	0	2	7	2	0	2	1	1	3	0	1	2	0	1	8	5	3	3	10	2	17	75	21.9055	4,5,10
CYC1_4	GOT1Srg_5	X06422_10	0	0	1	2	1	0	2	0	8	0	1	0	2	0	2	4	2	1	2	5	9	4	0	10	8	3	8	75	21.6433	4,5,10
GDHad_1	QCR10p_4	GOT1p1_8	2	3	2	0	0	2	4	0	14	0	0	2	0	1	0	1	0	1	1	0	5	2	2	5	5	8	15	75	21.6012	1,4,8
RISP_8	GOT2_8	ME1ad_12	3	3	3	0	0	0	3	0	1	0	0	0	3	2	6	1	3	4	0	2	4	0	3	4	6	11	15	77	21.5657	8,8,12
CYCad_6	QCR6p_9	X06422_10	1	0	2	0	1	0	2	2	3	1	3	3	2	0	0	2	1	9	6	3	4	0	0	5	10	2	13	75	21.4712	6,9,10
P060_2	X14140_3	X34449_5	2	2	5	0	0	0	3	2	2	1	0	1	0	3	0	2	3	8	0	5	11	1	0	1	5	5	14	76	21.364	2,3,5

DA F2 nauplii

			Three Locus combinations (first/second/third locus genotype)																														Three Locus Totals	
first_locus	second_locus	third_locus	AA AA AA	AA AA DD	AA AA AD	AA DD AA	AA DD DD	AA DD AD	AA AD AA	AA AD DD	AA AD AD	DD AA AA	DD AA DD	DD AA AD	DD DD AA	DD DD DD	DD DD AD	DD AD AA	DD AD DD	DD AD AD	AD AA AA	AD AA DD	AD AA AD	AD DD AA	AD DD DD	AD DD AD	AD AD AA	AD AD DD	AD AD AD	Total #		chi-square value	Chromosome Combos	# p<0.05
l34449_5	CYCad_6	RISP_8	5	1	0	0	0	2	0	3	1	0	1	1	4	0	2	6	3	8	2	1	6	6	0	6	2	13	10	83		33.574	1,3,9	1
l34449_5	QCR7p_6	RISP_8	5	1	0	0	0	2	0	3	1	0	1	3	3	0	2	6	3	6	2	1	6	6	0	6	2	13	10	82		32.176	1,4,9	2
ME2ad_3	P169_4	GOT1Srg_5	1	3	1	2	1	2	2	7	18	0	4	0	0	0	2	2	1	4	1	1	7	1	5	8	6	1	6	86		30.9937	1,4,10	1
l22708_3	P169_4	GOT1Srg_5	1	3	1	1	1	2	2	7	18	0	4	0	1	0	2	2	1	4	1	1	7	1	6	8	6	1	6	87		30.6554	1,6,8	1
QCR10p_4	QCR6p_9	ME1ad_12	2	4	4	1	0	5	0	3	1	4	0	5	0	1	0	1	4	1	4	4	3	1	4	3	7	3	16	81		28.7045	2,3,5	2
l14140_3	QCR10p_4	GOT1Srg_5	2	1	2	1	0	0	4	1	12	0	5	0	0	0	3	3	0	3	0	4	7	1	5	6	3	6	14	83		28.137	2,3,7	2
P169_4	QCR6p_9	ME1ad_12	2	4	4	0	0	5	1	2	0	4	1	4	0	2	0	1	6	2	4	4	4	2	4	4	6	2	16	84		28.111	2,3,9	3
l34449_5	CYCad_6	GOT2_8	5	1	1	1	0	1	0	2	3	1	1	0	3	0	2	9	2	6	1	0	8	6	3	4	3	11	11	85		28.0129	2,4,9	1
GDHad_1	QCR10p_4	QCR6p_9	3	3	1	5	0	0	0	2	9	0	2	2	3	1	2	6	1	6	7	2	1	1	0	4	6	5	13	85		27.6482	2,5,8	7
GOT1Srg_5	QCR7p_6	GOT2_8	6	1	0	1	0	2	0	2	2	1	1	2	2	0	2	6	3	5	1	0	8	7	2	3	5	10	11	83		27.6242	2,5,10	1
ME2ad_3	QCR10p_4	GOT1Srg_5	2	4	2	1	1	2	2	5	17	0	4	0	0	0	2	2	1	4	0	1	7	1	4	5	7	2	8	84		26.8352	2,6,8	4
mtMDH_2	l34449_5	cytMDH_10	2	1	0	1	2	2	2	1	8	4	0	4	1	0	4	3	3	1	0	0	3	8	4	2	9	5	15	85		26.033	2,6,12	1
P102_3	l34449_5	RPOL_7	2	0	3	6	4	4	3	6	6	1	0	0	1	0	2	0	6	3	2	6	0	0	4	3	10	6	7	85		25.7407	3,3,6	1
P169_4	RISP_8	l06422_10	1	0	3	1	3	3	3	0	4	4	3	2	4	0	0	1	2	5	3	1	7	0	3	8	10	1	10	82		25.5467	3,4,5	6
QCR10p_4	RISP_8	l06422_10	1	0	2	1	3	3	6	0	4	3	2	1	4	0	0	1	0	5	4	2	9	0	3	8	7	2	10	81		25.4414	3,4,9	3
ME2ad_3	GOT1Srg_5	ME1ad_12	0	3	2	3	2	5	5	2	14	2	0	0	0	0	5	1	2	3	2	2	3	4	2	1	3	11	6	83		25.3926	3,5,7	1
GOT1Srg_5	CYCad_6	RISP_8	5	1	0	1	0	2	0	3	2	0	1	2	2	0	3	5	4	6	2	1	5	6	0	6	3	12	11	83		25.3234	3,5,9	1
l14140_3	GOT1Srg_5	QCR6p_9	3	2	2	2	1	0	0	5	9	2	1	0	2	3	0	2	1	3	0	0	4	6	3	6	14	1	13	85		25.1107	3,5,12	5
mtMDH_2	GOT1Srg_5	GOT1p1_8	3	0	0	4	1	0	3	2	6	0	1	6	1	2	3	3	3	1	1	2	1	5	3	5	7	11	12	86		24.8184	3,6,8	2
P169_4	RPOL_7	GOT2_8	3	1	1	2	2	1	1	2	5	0	0	3	2	3	7	5	0	0	7	4	5	6	2	7	2	6	6	83		24.5419	4,6,8	1
mtMDH_2	GOT1Srg_5	GOT1p2_8	3	0	0	4	1	0	3	2	6	0	1	6	1	2	3	3	3	1	1	1	2	5	2	5	7	12	11	85		24.4939	4,6,10	1
GOT1Srg_5	CYCad_6	GOT2_8	5	1	0	1	0	2	1	2	3	1	1	1	2	0	2	6	3	6	1	0	8	7	3	3	5	10	11	85		24.274	4,7,8	1
l34449_5	QCR7p_6	GOT2_8	5	1	1	1	0	1	0	2	2	2	1	1	3	0	2	8	2	5	1	0	8	6	2	4	3	11	11	83		24.2271	4,8,10	2
ME2ad_3	P169_4	QCR6p_9	4	1	0	2	0	3	6	5	16	1	3	0	0	1	1	2	0	5	5	1	3	7	1	6	5	4	4	86		24.0722	4,9,12	2
l34449_5	CYCad_6	GOT1p2_8	4	0	3	1	1	1	0	3	2	1	1	0	4	0	1	8	2	7	1	1	7	5	5	3	3	12	10	86		24.0206	5,6,8	9
l14140_3	GOT1Srg_5	ME1ad_12	0	4	3	0	2	1	3	2	9	2	0	1	0	0	4	1	2	3	2	1	1	7	2	6	5	11	11	83		23.9955		
QCR9p_2	CYC1_4	QCR6p_9	3	1	0	2	0	0	4	0	9	2	2	1	1	2	3	3	0	7	3	0	6	7	2	2	6	8	10	84		23.9561		
GDHad_1	P169_4	QCR6p_9	3	2	1	4	1	0	1	3	9	0	2	2	3	1	3	6	1	5	7	1	0	2	1	7	6	5	11	87		23.8682		
l22708_3	P169_4	QCR6p_9	4	1	0	2	0	2	6	5	16	1	3	0	0	1	2	2	0	5	5	1	3	7	2	6	5	4	4	87		23.8083		
GOT1Srg_5	QCR7p_6	RISP_8	5	1	1	1	0	2	0	3	1	0	1	3	2	0	2	5	4	5	2	1	5	6	0	6	3	12	11	82		23.5392		
l14140_3	QCR7p_6	RISP_8	3	0	3	4	0	2	0	4	7	3	0	1	2	0	1	0	4	2	1	3	5	3	0	7	7	11	8	81		23.3916		
ME2ad_3	l14140_3	QCR7p_6	5	5	9	0	2	0	4	3	7	0	0	0	3	1	5	1	0	3	1	1	2	1	0	1	5	7	16	82		23.2121		
QCR9p_2	l22708_3	QCR6p_9	1	0	7	1	1	0	7	0	2	2	1	3	2	1	1	2	2	7	9	5	8	0	2	6	7	4	4	85		23.1838		
l22708_3	QCR10p_4	GOT1Srg_5	1	4	2	1	1	2	2	5	17	1	4	0	0	0	2	2	1	4	0	2	7	1	4	5	7	2	8	85		23.1438		
P060_2	l34449_5	GOT2_8	3	0	0	4	1	1	3	0	8	1	2	5	2	1	2	1	2	5	2	1	0	7	1	5	6	12	10	85		22.8831		
mtMDH_2	l14140_3	GOT1Srg_5	0	0	2	2	0	1	1	5	8	4	1	3	0	3	0	3	2	4	3	2	9	1	2	5	0	8	16	85		22.7534		
QCR9p_2	QCR8p_3	QCR6p_9	1	0	6	2	1	0	6	0	3	2	1	3	3	0	1	1	3	7	10	4	8	0	2	4	6	5	6	85		22.7225		
l22708_3	GOT1Srg_5	ME1ad_12	0	2	2	3	2	5	5	2	14	2	1	0	0	0	5	1	2	3	2	2	3	4	2	1	3	11	6	83		22.6998		
l14140_3	P169_4	GOT1Srg_5	1	1	1	2	0	1	4	2	12	0	4	0	0	1	2	3	0	4	1	3	7	1	6	9	2	6	12	85		22.6348		
GDHad_1	l14140_3	QCR6p_9	1	4	3	3	1	0	4	1	7	1	0	2	1	4	1	6	0	6	3	4	6	2	0	2	10	3	10	85		22.6155		
GDHad_1	CYC1_4	l06422_10	0	0	5	4	2	2	2	1	7	1	3	1	2	0	3	5	2	6	6	0	3	3	1	3	5	6	13	86		22.4942		
CYC1_4	CYCad_6	GOT1p1_8	0	0	2	3	0	0	2	7	5	0	1	5	1	3	2	4	3	2	6	1	3	6	3	4	5	8	11	87		22.4246		
QCR8p_3	GOT1Srg_5	ME1ad_12	0	2	1	4	3	4	5	2	13	2	0	0	0	0	4	1	2	4	2	3	4	3	1	3	3	11	6	83		22.1976		
mtMDH_2	QCR7p_6	GOT1p2_8	5	1	1	3	1	1	2	1	4	1	0	5	1	1	3	2	5	2	0	1	6	6	3	1	7	10	11	84		22.1975		
ME2ad_3	l34449_5	ME1ad_12	0	2	2	3	3	5	5	2	13	2	0	0	0	0	3	1	2	5	2	2	3	5	2	2	2	11	5	82		22.1622		
l14140_3	CYCad_6	RISP_8	3	0	2	5	0	3	0	4	8	3	0	1	2	0	1	1	4	2	1	3	4	3	0	7	7	11	9	84		22.1186		
GDHad_1	CYCad_6	RISP_8	0	2	1	5	0	5	0	5	6	4	0	2	3	0	0	4	5	3	3	1	4	2	0	6	4	9	10	84		21.8938		
QCR9p_2	ME2ad_3	QCR6p_9	1	0	7	1	1	0	7	0	2	2	1	3	2	1	1	2	2	7	9	5	9	0	2	5	7	4	4	85		21.8685		
P060_2	GOT1Srg_5	GOT1p1_8	3	1	0	4	1	0	3	2	6	0	2	6	1	2	3	3	3	2	1	1	1	5	2	5	7	11	11	86		21.8388		
mtMDH_2	l34449_5	GOT2_8	3	0	0	3	1	1	3	0	8	1	2	4	2	1	2	1	2	4	2	1	0	8	1	5	6	12	11	84		21.7851		
QCR9p_2	QCR7p_6	RISP_8	4	1	2	0	0	4	1	6	1	1	1	2	2	0	3	2	4	5	2	1	5	7	0	3	5	9	11	82		21.701		
mtMDH_2	QCR7p_6	ME1ad_12	4	0	3	1	1	3	4	1	2	1	0	5	0	3	1	1	3	5	2	5	0	1	2	6	6	8	13	81		21.6274		
ME2ad_3	QCR10p_4	QCR6p_9	4	2	2	3	0	1	5	3	16	1	3	0	0	1	1	2	0	5	5	1	2	6	0	4	5	5	7	84		21.5352		
QCR9p_2	l22708_3	RPOL_7	2	1	5	1	1	0	2	4	3	4	1	1	0	0	4	4	6	1	7	8	7	2	4	1	2	7	6	84		21.5065		
QCR9p_2	ME2ad_3	RPOL_7	2	1	5	1	1	0	2	4	3	4	1	1	0	0	4	4	6	1	7	8	7	2	4	1	2	7	6	84		21.5065		
QCR9p_2	QCR7p_6	GOT1p1_8	5	1	1	2	1	1	1	3	4	1	0	4	2	0	3	3	6	2	0	2	6	6	2	2	7	7	11	83		21.417		
P060_2	GOT1Srg_5	GOT2_8	4	0	0	3	1	1	3	0	8	1	2	5	1	1	3	2	2	4	2	1	0	5	2	5	8	11	10	85		21.3303		
P060_2	GOT1Srg_5	GOT1p2_8	3	0	1	4	1	0	3	2	6	0	2	6	1	2	3	3	3	2	1	1	1	5	2	5	7	12	10	86		21.3152		
P169_4	CYCad_6	l06422_10	1	0	2	1	0	4	3	3	4	1	2	2	1	4	1	7	0	4	4	1	5	4	0	6	6	5	15	86		21.2849		
P060_2	l14140_3	GOT1Srg_5	1	0	2	2	0	1	1	5	8	4	1	4	0	3	0	3	2	4	2	1	8	1	2	5	0	8	16	84		21.2484		
QCR9p_2	CYCad_6	RISP_8	4	1	2	0	0	4	1	6	1	1	1	1	2	0	3	2	4	6	2	1	4	7	0	4	5	9	12	83		21.2083		

AD all F2 adults

			Three Locus combinations (first/second/third locus genotype)																														Three Locus Totals	
first_locus	second_locus	third_locus	AA AA AA	AA AA DD	AA AA AD	AA DD AA	AA DD DD	AA DD AD	AA AD AA	AA AD DD	AA AD AD	DD AA AA	DD AA DD	DD AA AD	DD DD AA	DD DD DD	DD DD AD	DD AD AA	DD AD DD	DD AD AD	AD AA AA	AD AA DD	AD AA AD	AD DD AA	AD DD DD	AD DD AD	AD AD AA	AD AD DD	AD AD AD	Total #		chi-square value	Chromosome Combos	# p<0.05
X14140_3	P169_4	GOT1p2_8	18	11	41	5	3	19	21	17	35	1	7	7	0	0	1	12	7	8	10	17	42	13	5	20	60	39	130	549		36.0416	1,2,3	1
X14140_3	P169_4	GOT1p1_8	18	11	41	5	3	19	22	17	35	1	7	7	0	0	1	12	7	8	10	17	42	13	5	20	60	40	130	551		35.7888	1,2,4	2
X14140_3	P169_4	RISP_8	14	14	42	9	5	13	23	22	30	1	8	6	0	0	1	4	6	17	12	23	33	10	10	17	57	46	128	551		33.8249	1,2,10	2
GDHad_1	l22708_3	P169_4	20	7	27	4	2	5	19	11	71	6	8	7	0	0	7	8	2	20	40	12	43	4	0	14	54	24	136	551		32.3507	1,2,12	1
X14140_3	P169_4	GOT2_8	16	10	44	6	3	18	23	19	32	2	6	7	0	0	1	9	7	11	10	17	42	15	6	17	63	43	124	551		32.0465	1,3,4	3
GDHad_1	RPOL_7	cytMDH_10	11	7	35	6	11	19	6	18	53	6	9	7	0	0	6	13	7	10	31	25	41	18	15	37	35	32	97	555		31.7423	1,3,10	2
GDHad_1	ME2ad_3	P169_4	20	7	27	4	2	6	19	11	70	6	8	7	0	0	6	9	2	21	40	12	41	4	0	15	54	24	139	554		30.7796	1,3,12	2
GDHad_1	X14140_3	P169_4	19	8	25	7	1	6	17	11	72	9	7	7	0	0	5	5	3	21	42	12	41	8	0	16	47	24	138	551		30.7464	1,4,10	1
X14140_3	P169_4	RPOL_7	22	14	34	5	9	13	19	14	41	7	4	4	1	0	0	7	4	16	26	13	30	17	3	18	68	51	111	551		30.6497	1,4,12	1
X14140_3	P169_4	l06422_10	16	18	35	10	4	13	12	17	44	5	7	3	1	0	0	4	8	15	15	16	38	6	10	22	54	52	123	548		30.1699	1,5,9	2
QCR9p_2	QCR10p_4	cytMDH_10	3	15	20	3	6	13	13	10	45	16	2	19	11	8	10	18	16	43	15	17	34	17	8	20	26	41	90	539		30.0597	1,7,10	1
QCR9p_2	l22708_3	P169_4	16	5	24	3	0	7	22	13	37	20	6	10	1	2	7	21	9	70	30	16	42	4	0	12	36	15	117	545		29.5135	1,8,10	1

P102_3	CYCad_6	RPOL_7	11	8	36	5	9	19	31	21	50	5	0	4	1	1	2	6	1	4	39	28	25	15	11	41	60	31	92	556	29.4093	2,3,4	17
QCR9p_2	ME2ad_3	P169_4	16	5	23	3	0	7	22	13	40	20	6	10	1	2	7	21	9	70	30	16	41	4	0	12	36	15	118	547	28.6491	2,3,5	2
X14140_3	QCR10p_4	ME1ad_12	15	14	36	10	7	15	24	18	28	3	5	4	0	0	7	5	6	13	14	21	29	12	17	28	51	39	118	539	28.2847	2,3,7	1
QCR9p_2	QCR8p_3	P169_4	17	6	30	4	0	4	20	12	38	18	4	15	0	2	5	24	11	68	26	19	50	3	0	7	41	12	115	551	28.2488	2,3,10	3
GDHad_1	I34449_5	QCR6p_9	7	4	17	8	4	27	20	25	55	0	1	10	1	7	6	11	4	17	8	17	47	14	9	56	35	32	113	555	28.1628	2,4,5	1
QCR9p_2	X14140_3	P169_4	16	6	23	4	0	6	21	12	42	22	7	11	4	1	8	16	9	68	32	14	40	7	0	13	30	17	119	548	28.0387	2,4,10	4
X14140_3	QCR10p_4	GOT1p2_8	19	13	33	7	5	20	17	13	41	1	4	7	4	2	1	8	8	8	10	19	36	22	7	28	50	34	122	539	27.8485	2,5,7	1
X14140_3	QCR10p_4	GOT1p1_8	19	13	33	7	5	20	17	13	41	1	4	7	4	2	1	8	8	8	10	19	36	22	7	28	50	35	122	540	27.7789	2,5,8	1
GOT1Srg_5	RPOL_7	RISP_8	8	13	22	7	4	5	15	14	32	12	17	18	8	3	18	14	16	29	25	10	46	13	24	30	27	34	87	551	27.6725	2,5,10	1
X14140_3	QCR10p_4	GOT2_8	17	11	37	8	6	18	18	14	39	2	3	7	4	2	1	5	8	11	9	20	36	26	8	23	52	37	118	540	27.6413	2,7,10	1
QCR8p_3	CYCad_6	RPOL_7	10	8	34	5	11	21	31	19	49	6	1	4	1	0	1	6	1	4	39	27	27	15	11	40	60	33	93	557	27.5859	3,4,5	4
RISP_8	QCR6p_9	I06422_10	6	9	15	2	9	12	28	8	39	8	4	22	3	5	13	24	20	37	8	10	20	12	17	29	33	50	108	551	27.1212	3,4,6	6
P102_3	QCR10p_4	GOT2_8	14	7	32	10	5	19	15	20	62	1	0	4	4	0	1	7	4	4	13	28	44	24	11	22	53	36	104	544	27.1018	3,4,7	3
P102_3	QCR7p_6	RPOL_7	11	9	37	5	8	19	31	21	50	5	1	4	1	1	2	6	1	4	39	29	26	15	12	42	60	31	93	563	27.0975	3,4,8	41
CYC1_4	I34449_5	ME1ad_12	8	8	11	6	16	18	23	15	44	8	8	14	6	6	9	10	19	33	8	16	29	31	12	28	36	33	98	553	26.8766	3,4,9	3
X14140_3	QCR10p_4	RISP_8	14	12	39	11	7	14	19	20	32	1	5	6	2	2	3	2	7	15	10	25	29	19	11	27	49	42	117	540	26.6829	3,4,10	7
ME2ad_3	CYC1_4	RISP_8	16	7	33	10	11	14	19	23	36	1	5	4	2	0	5	3	5	11	12	31	40	20	18	32	47	36	112	553	26.5368	3,4,12	2
ME2ad_3	QCR10p_4	GOT1p1_8	18	11	30	7	5	20	18	16	43	1	1	5	4	1	1	8	8	8	11	25	41	22	8	28	49	34	119	542	26.189	3,5,6	1
QCR8p_3	QCR7p_6	RPOL_7	10	9	35	5	10	21	31	19	49	6	2	4	1	0	1	6	1	4	39	28	28	15	12	41	60	33	94	564	26.1254	3,6,7	8
mtMDH_2	X14140_3	P169_4	15	5	24	4	0	5	19	9	41	21	7	14	3	1	8	14	12	66	34	15	36	8	0	14	36	17	124	552	26.1055	3,6,9	1
P060_2	X14140_3	P169_4	14	5	24	4	0	5	19	9	41	21	7	14	3	1	8	14	12	66	33	15	36	8	0	14	35	17	123	548	26.0215	3,6,12	2
GDHad_1	P169_4	ME1ad_12	13	7	22	8	1	11	23	24	54	1	5	8	2	7	1	10	5	19	26	28	44	9	9	18	43	48	106	552	26.0147	3,7,8	1
GDHad_1	QCR9p_2	QCR10p_4	13	6	20	13	5	20	10	17	57	2	4	4	6	9	8	8	2	14	23	12	44	18	15	50	48	26	87	541	25.9453	4,5,8	3
ME2ad_3	P169_4	I06422_10	13	20	32	9	4	14	16	14	45	4	4	0	1	1	0	3	10	14	19	17	46	7	9	21	51	53	124	551	25.9084	4,5,10	1
P060_2	ME2ad_3	P169_4	12	6	25	2	0	5	23	8	40	20	6	13	2	2	6	17	12	69	32	15	37	3	0	16	42	17	120	550	25.8269	4,5,12	5
I22708_3	QCR10p_4	GOT1p2_8	18	11	30	6	5	20	18	16	46	1	1	5	4	1	2	8	7	7	11	25	41	23	8	27	48	33	117	539	25.8221	4,6,10	2
mtMDH_2	I22708_3	P169_4	14	6	27	2	0	5	22	8	35	19	6	13	2	2	6	18	12	69	33	15	37	4	0	15	41	17	122	550	25.751	4,7,10	1
X14140_3	P169_4	GOT1Srg_5	17	16	37	8	2	17	16	19	38	2	3	10	0	0	1	4	7	16	10	24	35	10	10	18	53	53	123	549	25.7505	4,8,10	3
P060_2	I22708_3	P169_4	13	6	27	2	0	5	22	8	36	19	6	13	2	2	6	18	12	69	32	15	37	3	0	15	41	17	121	547	25.7033	5,7,8	3
GDHad_1	mtMDH_2	cytMDH_10	4	14	20	7	4	30	12	19	57	1	0	4	9	5	6	9	11	13	20	16	42	28	20	40	36	36	93	556	25.589	5,7,12	1
P102_3	QCR10p_4	GOT1p2_8	16	9	28	8	5	21	15	18	63	1	0	4	4	0	1	6	4	5	13	28	44	21	9	27	54	34	104	542	25.5523	6,7,8	2
mtMDH_2	ME2ad_3	P169_4	13	6	25	2	0	5	23	8	39	20	6	13	2	2	6	17	12	69	33	15	37	4	0	15	41	17	122	552	25.5368	6,7,10	2
ME2ad_3	P169_4	RISP_8	14	14	38	9	6	12	22	20	33	1	5	2	1	0	1	4	5	18	12	27	41	9	9	18	57	48	125	551	25.528	7,8,10	1
I22708_3	P169_4	RISP_8	14	14	38	9	6	12	22	21	34	1	5	2	1	0	1	4	4	18	12	27	41	9	9	18	56	48	123	549	25.3649	8,9,10	1
ME2ad_3	QCR10p_4	RISP_8	13	10	36	12	6	14	20	24	33	1	3	3	2	1	3	3	6	15	11	30	35	18	13	27	47	39	117	542	25.3115		
GDHad_1	QCR9p_2	CYC1_4	12	5	23	14	6	19	10	26	50	1	2	7	5	7	11	9	3	12	26	10	44	19	17	50	51	37	77	553	25.2673		
I22708_3	QCR10p_4	GOT1p1_8	18	11	30	6	5	20	18	16	46	1	1	5	4	1	2	8	7	7	11	25	41	23	8	27	48	35	116	540	25.257		
P102_3	QCR10p_4	GOT1p1_8	16	9	28	8	5	21	15	19	62	1	0	4	4	0	1	6	4	5	13	28	44	21	9	27	54	35	104	543	25.2514		
ME2ad_3	QCR7p_6	RPOL_7	5	11	23	6	10	15	31	18	50	8	1	4	1	2	3	6	3	9	42	26	38	14	9	44	60	32	84	555	25.2217		
QCR9p_2	I22708_3	QCR10p_4	12	6	27	3	1	6	23	15	32	18	8	10	1	4	5	18	17	62	29	17	42	3	2	11	34	26	104	536	25.102		
ME2ad_3	QCR10p_4	GOT1p2_8	18	11	30	7	5	20	18	16	43	1	1	5	4	1	1	8	7	9	11	25	41	22	8	28	49	33	119	541	25.0171		
I34449_5	RPOL_7	ME1ad_12	11	14	13	4	2	10	9	16	31	11	10	23	12	5	12	20	19	20	21	15	53	12	22	33	36	30	89	553	24.9999		
GDHad_1	P060_2	cytMDH_10	4	14	20	7	4	30	12	19	56	2	0	3	9	5	6	8	11	15	20	15	42	28	20	40	36	34	93	553	24.9642		
ME2ad_3	CYC1_4	GOT1p1_8	20	11	25	5	5	25	18	16	44	2	2	6	5	0	2	6	7	6	16	25	43	20	12	38	48	33	114	554	24.959		
ME2ad_3	CYCad_6	RPOL_7	5	10	23	6	10	15	31	18	50	8	1	4	1	2	3	6	3	9	42	25	38	14	9	44	60	32	84	553	24.9587		
QCR9p_2	GOT1Srg_5	cytMDH_10	6	4	18	2	7	22	11	21	38	18	3	12	15	6	23	15	17	38	12	15	31	13	16	31	34	34	87	549	24.7546		
QCR9p_2	X14140_3	QCR10p_4	14	6	24	4	1	5	20	15	38	19	9	11	2	4	7	16	16	59	32	17	36	6	2	12	27	26	110	538	24.7378		
CYC1_4	GOT1Srg_5	ME1ad_12	10	8	13	6	15	24	22	16	36	9	8	14	7	6	8	8	19	33	7	16	35	26	12	32	41	34	88	553	24.659		
ME2ad_3	P169_4	GOT1p1_8	17	11	38	5	3	19	21	17	37	1	3	4	1	0	1	11	7	9	11	22	48	12	5	20	61	42	126	552	24.5489		
ME2ad_3	CYC1_4	GOT1p2_8	20	11	25	5	5	25	18	16	44	2	2	6	5	0	2	6	7	6	16	24	43	20	12	38	48	33	114	553	24.5354		
ME2ad_3	QCR10p_4	GOT2_8	16	9	34	9	5	18	18	16	43	1	1	5	4	1	1	5	8	11	11	25	41	25	10	23	52	37	113	542	24.4685		
I22708_3	QCR7p_6	RPOL_7	5	10	24	7	10	16	30	19	50	7	1	4	1	2	2	7	3	9	42	27	37	13	9	43	60	31	83	552	24.4538		
I22708_3	P169_4	I06422_10	13	19	33	9	4	14	17	14	46	4	4	0	1	1	0	3	11	12	19	18	44	7	9	21	50	52	123	548	24.4443		
I22708_3	CYCad_6	RPOL_7	5	9	24	7	10	16	30	19	50	7	1	4	1	2	2	7	3	9	42	26	37	13	9	43	60	31	83	550	24.3195		
ME2ad_3	P169_4	GOT2_8	15	10	41	6	2	19	22	18	35	1	3	4	1	0	1	8	7	12	12	21	48	14	7	16	64	46	119	552	24.3076		
I22708_3	CYC1_4	GOT1p1_8	20	11	26	4	5	25	18	16	46	2	2	5	5	0	3	6	7	6	16	25	43	21	12	37	47	33	111	552	24.3017		
ME2ad_3	P169_4	GOT1p2_8	17	11	38	5	3	19	21	17	37	1	3	4	1	0	1	11	6	10	11	23	48	12	5	20	61	41	126	552	24.2816		
X14140_3	P169_4	cytMDH_10	16	21	33	9	3	15	15	16	43	2	3	10	0	0	1	6	6	15	16	18	35	11	11	16	51	48	131	551	24.2533		
GDHad_1	QCR9p_2	ME1ad_12	9	10	21	7	6	26	28	16	41	3	5	2	7	8	8	3	4	17	26	15	39	20	27	39	33	43	89	552	24.153		
mtMDH_2	P102_3	GOT1Srg_5	6	11	34	0	1	4	16	19	30	10	14	18	4	3	2	23	27	48	30	16	49	2	3	6	29	42	107	554	24.1367		
P169_4	I34449_5	ME1ad_12	7	9	11	9	17	15	24	14	48	4	6	4	5	1	8	10	10	18	13	17	39	29	16	31	34	43	108	550	24.0767		
I22708_3	CYC1_4	RISP_8	16	8	33	10	11	13	19	23	38	1	4	4	2	0	6	3	5	11	12	31	40	20	18	32	46	36	109	551	24.0366		
CYC1_4	RPOL_7	cytMDH_10	19	5	21	4	6	18	10	20	48	14	11	12	7	3	11	14	12	29	16	25	50	13	17	33	30	25	83	556	23.996		
P060_2	P102_3	GOT1Srg_5	7	10	34	0	1	4	16	19	30	10	14	18	4	3	2	23	27	48	30	16	49	2	2	6	28	42	106	551	23.8957		
QCR10p_4	RISP_8	cytMDH_10	7	4	14	12	8	23	15	21	38	17	4	11	4	7	9	10	11	23	13	13	44	16	13	40	29	41	95	542	23.8898		
I22708_3	CYC1_4	GOT1p2_8	20	11	26	4	5	25	18	16	46	2	2	5	5	0	3	6	7	6	16	24	43	21	12	37	47	33	111	551	23.8798		
GDHad_1	CYC1_4	cytMDH_10	6	8	24	6	9	22	11	20	61	6	0	9	4	6	2	9	10	12	20	23	54	25	11	28	39	38	93	556	23.8449		
X14140_3	P169_4	QCR7p_6	16	12	42	3	8	16	19	13	42	5	2	8	0	0	1	7	5	15	21	16	32	12	8	18	75	39	117	552	23.8437		
X14140_3	P169_4	CYCad_6	16	12	42	3	8	16	17	15	42	5	2	8	0	0	1	7	5	15	21	16	32	12	8	18	74	39	117	551	23.7775		
P060_2	QCR8p_3	P169_4	15	6	32	3	0	3	19	8	36	16	4	17	1	2	5	22	14	67	29	19	47	2	0	8	45	13	118	551	23.7454		
QCR9p_2	GOT1Srg_5	RPOL_7	9	2	17	10	4	17	20	13	37	14	1	19	20	7	17	24	22	24	20	13	25	17	18	25	38	32	86	551	23.5557		
I34449_5	RPOL_7	RISP_8	8	12	19	7	4	5	14	14	28	10	14	20	9	2	18	13	15	30	27	14	47	12	25	30	30	35	90	552	23.527		
P169_4	QCR7p_6	cytMDH_10	13	6	23	7	10	14	14	26	42	1	6	8	5	4	7	14	4	17	27	27	48	11	9	38	36	34	106	557	23.4574		
X14140_3	P169_4	QCR6p_9	13	13	44	6	6	15	13	7	52	3	2	10	1	0	0	3	4	20	17	13	39	8	10	20	40	46	144	549	23.3789		
X14140_3	CYC1_4	QCR7p_6	10	7	41	4	4	24	23	22	36	5	3	6	2	2	5	5	2	13	22	17	38	26	13	33	60	33	98	554	23.3701		
I22708_3	CYCad_6	ME1ad_12	7	9	21	6	12	15	33	20	46	2	2	8	3	2	0	5	4	10	26	25	53	21	9	35	31	49	94	548	23.3651		
QCR8p_3	QCR10p_4	GOT1p1_8	17	12	28	6	5	21	17	14	61	1	0	5	3	1	2	3	4	6	12	25	43	24	8	26	55	40	104	543	23.3138		
QCR8p_3	QCR10p_4	GOT1p2_8	17	12	28	6	5	21	17	14	61	1	0	5	3	1	2	3	4	6	12	25	43	24	8	26	55	39	104	542	23.3091		
X14140_3	QCR10p_4	cytMDH_10	17	19	29	12	5	15	9	15	46	3	1	8	2	2	3	3	6	15	14	14	37	17	15	25	45	46	116	539	23.2774		
GDHad_1	QCR9p_2	QCR8p_3	16	0	24	13	2	24	31	2	53	5	3	2	4	1	18	11	0	13	31	5	44	20	4	62	54	8	103	553	23.189		
GDHad_1	I22708_3	ME1ad_12	14	11	29	2	2	7	28	19	53	3	8	10	5	0	2	5	9	16	30	22	43	3	6	9	46	55	113	550	23.1794		
I22708_3	P169_4	GOT1p2_8	17	11	38	5	3	19	20	17	40	1	3	4	1	0	1	11	6	9	11	22	48	12	5	20	61	41	123	549	23.1543		
QCR9p_2	QCR8p_3	QCR10p_4	15	7	29	4	1	3	19	14	36	17	7	13	0	4	3	20	18	62	25	18	49	2	1	7	39	26	102	541	23.1129		
I22708_3	QCR10p_4	GOT2_8	16	9	34	8	5	18	18	16	46	1	1	5	4	1	2	5	7	10	11	25	41	26	10	22	52	38	110	541	23.0524		
X14140_3	CYC1_4	GOT1p1_8	21	12	26	6	5	21	18	15	48	2	4	8	5	1	3	6	9	5	16	21	40	19	11	42	48	31	111	554	22.9823		
I22708_3	P169_4	GOT1p1_8	17	11	38	5	3	19	20	17	40	1	3	4	1	0	1	11	6	9	11	22	48	12	5	20	61	43	122	550	22.9804		
I22708_3	QCR7p_6	ME1ad_12	8	9	21	6	12	15	33	20	46	2	2	8	3	2	0	5	4	10	27	25	53	21	9	35	31	49	94	550	22.9634		
P169_4	GOT1Srg_5	cytMDH_10	11	2	16	13	9	21	10	31	42	8	3	7	2	3	7	10	8	18	17	17	39	15	17	48	41	34	104	553	22.8916		
P060_2	P102_3	cytMDH_10	10	15	27	3	0	2	14	14	36	10	5	26	2	5	2	32	19	48	16	20	60	4	1	5	36	43	98	553	22.8494		
QCR8p_3	QCR10p_4	GOT2_8	15	10	32	8	5	19	16	16	60	1	0	5	3	1	2	4	4	5	12	25	43	27	10	21	55	40	105	544	22.8206		
CYCad_6	RPOL_7	cytMDH_10	14	18	23	14	5	17	13	13	37	6	5	10	5	6	11	11	13	38	29	18	50	5	15	33	30	31	85	555	22.801		
QCR10p_4	GOT1Srg_5	RISP_8	7	6	13	11	15	20	7	22	41	8	5	11	12	4	6	12	11	27	15	20	32	11	17	37	43	32	96	541	22.7996		
P169_4	GOT1Srg_5	ME1ad_12	7	8	13	8	18	17	25	14	44	5	7	6	4	1	7	10	9	17	13	17	43	27	14	39	36	46	95	550	22.7875		
I22708_3	P169_4	GOT2_8	15	10	41	6	2	19	21	18	38	1	3	4	1	0	1	8	6	12	12	21	48	14	7	16	65	47	115	551	22.7258		
QCR9p_2	RPOL_7	cytMDH_10	5	11	23	3	7	10	11	15	46	22	15	21	7	4	19	19	7	34	22	15	38	14	16	33	23	35	78	553	22.5956		
X14140_3	CYC1_4	CYCad_6	10	8	41	4	4	24	22	22	36	5	3	6	2	2	5	5	2	13	22	17	38	26	13	33	59	33	98	553	22.5851		
GDHad_1	GOT1Srg_5	QCR6p_9	8	7	17	8	5	25	19	21	56	0	1	12	2	7	6	10	4	16	10	15	50	16	10	57	31	33	109	555	22.581		
ME2ad_3	CYC1_4	QCR7p_6	10	5	41	6	5	24	23	21	34	4	3	4	2	1	4	7	2	10	24	20	40	24	13	33	59	34	103	556	22.5478		
QCR9p_2	QCR8p_3	CYC1_4	14	6	32	4	0	4	21	11	38	16	7	15	1	4	2	22	19	63	25	23	48	2	2	6	43	41	85	554	22.509		

mtMDH_2	QCR8p_3	P169_4	16	6	31	3	0	3	19	8	37	16	4	17	1	2	5	22	14	67	29	19	48	3	0	8	46	13	119	556	22.4705
QCR8p_3	CYC1_4	GOT1p1_8	18	12	24	3	4	29	19	15	62	1	1	5	3	0	3	3	4	5	19	25	45	24	13	34	50	38	97	556	22.4513
ME2ad_3	CYC1_4	CYCad_6	10	5	41	6	5	24	22	21	34	4	3	4	2	1	4	7	2	10	24	20	40	24	13	33	58	34	103	554	22.4492
QCR9p_2	ME2ad_3	QCR10p_4	12	6	26	3	1	6	23	15	35	18	8	10	1	3	6	18	18	61	29	18	40	3	2	11	34	25	106	538	22.3801
mtMDH_2	P102_3	cytMDH_10	8	16	28	3	0	2	14	14	36	10	5	26	2	5	2	32	19	48	17	20	59	4	2	5	36	44	99	556	22.3055
P102_3	RPOL_7	RISP_8	12	10	23	12	12	14	22	25	58	4	3	5	3	0	0	0	0	10	29	27	58	14	18	40	37	40	83	559	22.1918
ME2ad_3	P169_4	QCR6p_9	14	12	40	6	8	13	13	9	52	0	0	8	1	1	0	3	5	19	19	16	46	8	7	22	40	44	146	552	22.1824
QCR8p_3	CYC1_4	GOT1p2_8	18	12	24	3	4	29	19	15	62	1	1	5	3	0	3	3	4	5	19	24	45	24	13	34	50	38	97	555	22.1688
I22708_3	P169_4	QCR6p_9	14	11	41	6	8	13	12	9	55	0	0	8	1	1	0	3	5	18	19	17	45	8	7	22	41	44	142	550	22.1014
RPOL_7	RISP_8	cytMDH_10	14	14	17	12	8	20	22	19	45	10	2	17	4	7	20	10	18	25	14	6	37	17	13	34	23	37	88	553	22.0404
P102_3	CYCad_6	QCR6p_9	11	10	33	6	5	21	16	20	66	0	5	4	2	1	1	0	0	11	16	17	59	10	11	46	41	34	108	554	22.0391
P102_3	CYC1_4	GOT1p1_8	17	11	24	4	4	30	18	18	62	3	1	4	4	0	3	4	3	3	18	26	46	22	13	33	50	35	99	555	21.8551
X14140_3	P169_4	ME1ad_12	18	17	35	9	6	12	22	18	32	3	5	7	0	0	1	5	6	16	18	18	32	10	11	17	49	52	129	548	21.8313
GDHad_1	X14140_3	cytMDH_10	8	18	27	1	3	10	14	16	70	9	5	9	1	1	3	8	10	11	22	16	56	6	5	13	56	51	103	552	21.7941
GDHad_1	I22708_3	cytMDH_10	8	18	29	1	4	6	14	15	72	8	4	9	1	3	3	10	9	11	23	17	54	4	4	10	56	50	109	552	21.7853
X14140_3	CYC1_4	GOT1p2_8	20	12	26	6	5	21	18	15	48	2	4	8	5	1	3	6	9	5	16	20	40	19	11	42	48	31	111	552	21.7755
ME2ad_3	P169_4	GOT1Srg_5	15	14	37	8	2	17	17	21	37	0	4	4	1	0	1	6	7	14	14	25	42	9	10	18	50	51	128	552	21.7411
QCR10p_4	GOT1p2_8	cytMDH_10	4	5	21	7	10	20	23	19	34	14	6	13	3	4	7	14	12	23	21	17	37	11	11	34	26	39	107	542	21.7241
QCR10p_4	I34449_5	GOT2_8	6	5	14	5	9	27	17	21	39	6	5	10	14	2	7	18	9	25	14	19	29	14	13	39	47	29	100	543	21.6593
P060_2	QCR10p_4	cytMDH_10	5	11	19	5	2	14	16	15	31	14	4	18	12	7	13	16	18	43	15	16	38	14	13	16	26	34	104	539	21.6421
P102_3	CYC1_4	I06422_10	11	15	26	7	8	23	21	19	59	6	2	0	1	3	3	2	4	4	16	24	50	22	11	34	39	46	99	555	21.6387
QCR10p_4	GOT1p1_8	cytMDH_10	4	5	21	7	10	20	23	19	34	14	6	13	3	4	7	14	12	23	21	17	37	11	11	36	26	39	106	543	21.6357
P169_4	CYCad_6	cytMDH_10	13	6	23	7	10	15	14	26	42	1	6	8	5	4	7	14	4	17	26	25	46	14	10	38	36	34	107	558	21.5822
ME2ad_3	P169_4	RPOL_7	20	13	33	5	8	14	17	17	41	4	2	2	2	0	0	8	4	14	31	16	34	16	4	17	69	47	113	551	21.5644
ME2ad_3	GOT1Srg_5	CYCad_6	7	9	24	9	5	23	22	17	52	6	1	1	3	4	4	4	1	14	21	17	35	25	11	50	60	39	89	553	21.5617
X14140_3	QCR10p_4	GOT1Srg_5	14	18	33	8	5	19	19	15	37	1	2	9	1	1	5	4	7	13	11	26	28	15	16	26	44	43	118	538	21.5386
P102_3	CYC1_4	GOT1p2_8	17	11	24	4	4	30	18	18	62	3	1	4	4	0	3	4	3	3	18	25	46	22	13	33	50	35	99	554	21.4639
GDHad_1	GOT2_8	cytMDH_10	9	9	23	5	8	21	9	20	63	3	7	7	5	1	6	11	8	10	31	13	41	15	17	36	38	42	98	556	21.3158
QCR10p_4	I34449_5	RISP_8	8	6	11	9	13	19	8	24	44	7	5	9	12	3	8	13	12	27	13	19	30	11	15	39	46	35	96	542	21.244
I22708_3	QCR10p_4	I06422_10	13	16	29	11	4	16	15	18	47	4	3	0	1	4	2	3	9	10	15	16	46	11	15	32	49	45	105	539	21.212
QCR10p_4	I34449_5	ME1ad_12	5	8	11	7	17	17	21	15	41	5	6	10	7	3	13	10	15	27	14	16	32	28	13	25	38	34	103	541	21.1931
QCR9p_2	P102_3	cytMDH_10	6	12	32	2	2	2	11	18	45	13	3	25	2	4	1	33	19	48	16	25	56	5	1	6	38	40	87	552	21.1909
mtMDH_2	P169_4	GOT1Srg_5	5	6	27	3	3	8	14	22	34	7	16	16	6	1	13	24	26	39	17	21	40	9	8	15	35	32	106	553	21.185
X14140_3	P169_4	I34449_5	15	16	39	7	4	16	14	14	44	2	3	10	0	0	1	5	7	15	11	22	36	7	10	21	50	54	127	550	21.1724
P060_2	GOT1Srg_5	GOT2_8	9	2	10	4	9	17	14	16	39	11	9	17	12	13	19	16	14	38	13	18	29	18	5	37	46	30	85	550	21.1378
CYCad_6	RPOL_7	GOT1p2_8	16	6	32	6	5	25	14	13	37	6	5	10	9	6	6	14	16	32	27	22	48	7	9	37	40	28	76	552	21.1169
QCR9p_2	I22708_3	RPOL_7	15	6	24	2	2	6	22	11	39	13	12	11	6	1	3	38	17	46	14	21	54	7	3	6	54	39	76	548	21.1156
QCR7p_6	RPOL_7	GOT1p2_8	16	6	32	7	5	26	14	13	37	6	5	10	9	6	6	14	16	32	27	22	48	7	9	37	40	28	76	554	21.1113
QCR9p_2	P169_4	cytMDH_10	4	15	22	2	5	11	13	13	46	17	5	20	8	4	5	22	17	48	13	22	35	10	5	16	36	39	96	549	21.0715
I22708_3	P169_4	RPOL_7	21	12	33	5	8	14	16	18	43	4	2	2	2	0	0	9	4	13	30	17	34	16	4	17	69	46	110	549	21.071
GOT1Srg_5	RPOL_7	GOT2_8	10	9	24	6	4	6	17	16	27	14	13	20	9	2	18	11	12	37	29	11	42	10	16	41	37	31	81	553	21.0541
mtMDH_2	QCR10p_4	cytMDH_10	5	12	18	5	2	14	15	15	33	14	4	18	12	7	13	16	18	43	15	18	39	14	13	16	27	34	103	543	21.0499
GDHad_1	QCR8p_3	ME1ad_12	20	15	25	0	0	4	24	17	60	2	7	12	3	0	1	8	10	15	32	27	46	4	4	9	43	54	113	555	21.0477
QCR7p_6	RPOL_7	cytMDH_10	14	18	23	14	7	18	13	13	37	6	5	10	5	5	11	11	13	38	29	18	50	5	15	33	30	31	85	557	21.0338

AD 16˚

first_locus	second_locus	third_locus	Three Locus combinations (first/second/third locus genotype)																											Total #	chi-square value	Three Locus Totals	
			AA AA AA	AA AA DD	AA AA AD	AA DD AA	AA DD DD	AA DD AD	AA AD AA	AA AD DD	AA AD AD	DD AA AA	DD AA DD	DD AA AD	DD DD AA	DD DD DD	DD DD AD	DD AD AA	DD AD DD	DD AD AD	AD AA AA	AD AA DD	AD AA AD	AD DD AA	AD DD DD	AD DD AD	AD AD AA	AD AD DD	AD AD AD			Chromosome Combos	# p<0.05
CYC1_4	RISP_8	cytMDH_10	4	0	10	3	3	18	5	11	16	9	0	6	7	5	2	6	6	13	7	7	16	9	9	18	20	16	42	268	26.1509	1,2,10	1
CYCad_6	RISP_8	I06422_10	3	2	1	4	7	13	2	10	22	1	1	7	1	4	9	5	13	21	12	5	27	16	4	16	15	16	31	268	26.0427	1,3,4	1
QCR7p_6	RISP_8	I06422_10	3	2	1	4	7	13	2	10	22	1	1	7	1	4	9	5	13	21	12	5	27	16	4	16	15	16	31	268	26.0427	1,3,12	3
mtMDH_2	I34449_5	RISP_8	2	1	3	3	5	6	9	14	16	3	5	7	1	9	10	12	6	19	5	12	11	12	2	18	12	20	45	268	25.3334	1,7,10	1
P060_2	I34449_5	RISP_8	2	1	3	3	5	6	8	14	17	3	5	7	1	9	10	12	6	19	5	12	11	12	2	18	12	20	44	267	24.9961	2,3,7	1
I22708_3	CYCad_6	RPOL_7	1	2	13	5	7	10	11	12	28	2	0	1	0	2	1	1	1	2	17	15	14	6	6	24	33	17	38	269	24.8063	2,5,7	1
I22708_3	QCR7p_6	RPOL_7	1	2	13	5	7	10	11	12	28	2	0	1	0	2	1	1	1	2	17	15	14	6	6	24	33	17	38	269	24.8063	2,5,8	2
CYC1_4	GOT1Srg_5	RISP_8	4	4	5	8	8	7	2	12	20	4	8	5	3	2	3	8	4	17	4	6	16	4	10	22	21	20	41	268	24.7374	2,5,10	2
CYCad_6	RISP_8	QCR6p_9	2	0	4	5	6	13	5	10	19	5	3	1	2	2	10	4	6	29	8	8	28	11	3	22	11	10	42	269	24.3065	2,6,10	2
QCR7p_6	RISP_8	QCR6p_9	2	0	4	5	6	13	5	10	19	5	3	1	2	2	10	4	6	29	8	8	28	11	3	22	11	10	42	269	24.3065	2,6,12	2
CYC1_4	GOT1p1_8	I06422_10	1	6	10	6	9	9	6	5	18	4	2	9	2	2	4	11	1	19	9	6	18	6	4	18	14	28	43	270	24.0692	2,7,8	3
CYC1_4	GOT1p2_8	I06422_10	1	6	10	6	9	9	6	5	18	4	2	9	2	2	4	11	1	19	9	6	18	6	4	18	14	28	43	270	24.0692	2,7,10	1
X14140_3	I34449_5	CYCad_6	1	9	8	4	4	11	8	10	31	1	0	2	0	3	1	5	1	2	7	6	15	10	5	28	28	23	45	268	24.0149	3,4,10	1
X14140_3	I34449_5	QCR7p_6	1	9	8	4	4	11	8	10	31	1	0	2	0	3	1	5	1	2	7	6	15	10	5	28	28	23	45	268	24.0149	3,5,6	10
ME2ad_3	CYCad_6	RPOL_7	1	3	12	4	7	10	11	12	28	3	0	1	0	2	1	1	1	2	17	14	15	7	6	25	33	17	39	272	23.8197	3,5,7	4
ME2ad_3	QCR7p_6	RPOL_7	1	3	12	4	7	10	11	12	28	3	0	1	0	2	1	1	1	2	17	14	15	7	6	25	33	17	39	272	23.8197	3,6,7	4
P169_4	CYCad_6	cytMDH_10	4	4	14	5	9	9	6	11	18	1	3	8	5	4	6	14	3	17	12	7	11	1	5	18	22	12	39	268	23.532	3,6,10	4
P169_4	QCR7p_6	cytMDH_10	4	4	14	5	9	9	6	11	18	1	3	8	5	4	6	14	3	17	12	7	11	1	5	18	22	12	39	268	23.532	3,7,9	1
GDHad_1	RPOL_7	cytMDH_10	5	3	16	3	8	11	4	9	20	4	2	6	0	0	6	11	4	5	16	10	14	6	8	20	21	14	44	270	23.4292	3,7,10	1
CYCad_6	RISP_8	cytMDH_10	3	0	3	6	9	9	8	5	20	1	1	7	4	4	6	6	13	20	16	6	22	9	4	23	17	15	31	268	23.3084	3,8,10	2
QCR7p_6	RISP_8	cytMDH_10	3	0	3	6	9	9	8	5	20	1	1	7	4	4	6	6	13	20	16	6	22	9	4	23	17	15	31	268	23.3084	4,5,8	5
CYCad_6	GOT2_8	QCR6p_9	3	0	9	4	5	10	5	11	18	5	5	3	2	2	12	4	4	24	6	5	24	5	1	18	20	15	50	270	23.1926	4,5,10	1
QCR7p_6	GOT2_8	QCR6p_9	3	0	9	4	5	10	5	11	18	5	5	3	2	2	12	4	4	24	6	5	24	5	1	18	20	15	50	270	23.1926	4,5,12	1
ME2ad_3	GOT1Srg_5	RPOL_7	9	4	8	1	5	10	6	13	32	1	1	1	0	2	2	3	0	1	10	4	19	19	9	19	28	24	40	271	23.0395	4,6,10	2
GDHad_1	QCR9p_2	cytMDH_10	3	6	14	4	2	12	5	12	20	1	1	3	10	0	5	3	5	9	7	7	23	13	8	13	23	17	42	268	23.0337	4,8,10	4
ME2ad_3	I34449_5	RPOL_7	7	4	7	1	5	12	8	13	30	0	1	1	0	2	2	3	0	1	8	4	17	20	7	17	29	26	45	270	22.9293	4,10,12	1
I22708_3	GOT1Srg_5	RPOL_7	9	4	8	2	5	10	6	12	33	0	1	1	0	2	2	3	0	1	10	4	17	18	9	19	28	25	39	268	22.89	5,6,10	2
GDHad_1	ME2ad_3	ME1ad_12	3	5	16	1	1	3	14	9	27	3	7	6	2	0	0	2	6	12	15	13	20	0	3	0	23	28	52	271	22.8849	6,8,9	8
I22708_3	I34449_5	CYCad_6	1	8	9	3	5	11	12	9	30	1	0	1	0	3	1	2	0	2	7	7	15	11	4	28	28	25	45	268	22.6702	6,8,10	4
I22708_3	I34449_5	QCR7p_6	1	8	9	3	5	11	12	9	30	1	0	1	0	3	1	2	0	2	7	7	15	11	4	28	28	25	45	268	22.6702	7,8,12	1
CYC1_4	I34449_5	GOT1p1_8	4	2	7	5	8	7	9	14	15	2	5	8	5	0	4	8	3	19	1	8	12	9	4	24	23	16	48	270	22.6353		
CYC1_4	I34449_5	GOT1p2_8	4	2	7	5	8	7	9	14	15	2	5	8	5	0	4	8	3	19	1	8	12	9	4	24	23	16	48	270	22.6353		
CYCad_6	GOT1p1_8	QCR6p_9	4	0	10	4	4	10	4	12	17	6	5	5	2	3	11	3	3	24	6	5	25	6	3	17	19	13	50	271	22.6129		
CYCad_6	GOT1p2_8	QCR6p_9	4	0	10	4	4	10	4	12	17	6	5	5	2	3	11	3	3	24	6	5	25	6	3	17	19	13	50	271	22.6129		
QCR7p_6	GOT1p1_8	QCR6p_9	4	0	10	4	4	10	4	12	17	6	5	5	2	3	11	3	3	24	6	5	25	6	3	17	19	13	50	271	22.6129		
QCR7p_6	GOT1p2_8	QCR6p_9	4	0	10	4	4	10	4	12	17	6	5	5	2	3	11	3	3	24	6	5	25	6	3	17	19	13	50	271	22.6129		
X14140_3	CYCad_6	I06422_10	2	6	5	4	4	15	14	12	24	0	1	5	0	2	2	3	2	0	7	12	26	3	12	19	26	12	50	268	22.5858		
X14140_3	QCR7p_6	I06422_10	2	6	5	4	4	15	14	12	24	0	1	5	0	2	2	3	2	0	7	12	26	3	12	19	26	12	50	268	22.5858		
GOT1Srg_5	CYCad_6	I06422_10	3	1	6	4	1	12	6	3	20	4	6	6	0	6	6	16	7	16	3	12	25	3	11	19	21	16	37	270	22.55		
GOT1Srg_5	QCR7p_6	I06422_10	3	1	6	4	1	12	6	3	20	4	6	6	0	6	6	16	7	16	3	12	25	3	11	19	21	16	37	270	22.55		
ME2ad_3	GOT1Srg_5	CYCad_6	1	8	12	3	3	10	12	10	29	2	0	1	0	3	1	2	0	2	7	9	17	13	6	28	26	23	43	271	22.3113		
ME2ad_3	GOT1Srg_5	QCR7p_6	1	8	12	3	3	10	12	10	29	2	0	1	0	3	1	2	0	2	7	9	17	13	6	28	26	23	43	271	22.3113		
QCR9p_2	RPOL_7	cytMDH_10	4	2	11	1	5	4	6	7	25	12	6	10	3	2	11	13	2	9	10	7	15	5	9	22	16	18	34	269	22.2663		
QCR10p_4	cytMDH_10	ME1ad_12	3	8	2	5	3	11	10	9	20	7	6	8	1	4	5	6	4	15	6	6	24	8	5	15	15	25	34	265	22		
RPOL_7	GOT2_8	ME1ad_12	4	3	17	4	2	8	9	16	13	2	2	4	1	2	11	11	10	19	7	7	14	10	11	10	15	19	39	270	21.9981		
QCR9p_2	QCR7p_6	ME1ad_12	0	5	14	7	3	8	6	8	17	8	9	3	3	5	4	9	14	15	6	4	17	9	8	17	16	17	42	274	21.9302		
QCR10p_4	I34449_5	GOT1p1_8	3	2	9	2	8	7	8	14	18	2	4	5	7	0	7	9	3	19	2	9	11	10	4	20	23	15	44	265	21.9218		
QCR10p_4	I34449_5	GOT1p2_8	3	2	9	2	8	7	8	14	18	2	4	5	7	0	7	9	3	19	2	9	11	10	4	20	23	15	44	265	21.9218		
P169_4	RISP_8	cytMDH_10	4	2	10	7	4	13	4	17	18	8	2	6	4	6	5	8	2	19	8	3	15	8	7	19	19	14	34	266	21.9066		
X14140_3	QCR10p_4	cytMDH_10	6	11	13	10	2	10	4	7	22	1	1	5	0	0	1	0	2	5	6	7	20	11	8	14	31	19	47	263	21.8896		
QCR9p_2	RPOL_7	GOT2_8	5	2	10	1	4	5	6	7	24	7	5	15	1	2	13	5	12	8	12	7	13	6	8	22	17	11	40	268	21.8529		

ME2ad_3	l34449_5	CYCad_6	1	8	9	3	4	11	12	9	30	1	0	1	0	3	1	2	0	2	7	7	15	11	5	28	28	26	46	270	21.8001
ME2ad_3	l34449_5	QCR7p_6	1	8	9	3	4	11	12	9	30	1	0	1	0	3	1	2	0	2	7	7	15	11	5	28	28	26	46	270	21.8001
QCR9p_2	ME2ad_3	RPOL_7	4	3	15	0	1	1	13	6	22	7	7	5	4	0	2	17	9	18	5	12	29	0	2	1	27	22	38	270	21.7637
GDHad_1	l22708_3	ME1ad_12	3	5	16	1	1	3	14	9	27	3	7	6	2	0	0	2	6	12	14	14	21	0	3	0	24	26	50	269	21.7358
QCR9p_2	CYCad_6	ME1ad_12	0	5	13	7	3	7	6	8	16	8	9	3	3	4	4	9	14	15	6	4	17	9	8	17	16	17	42	270	21.7231
QCR9p_2	RPOL_7	GOT1p1_8	4	2	11	2	4	4	7	7	24	7	3	17	2	3	11	5	11	9	12	9	11	7	9	20	20	11	37	269	21.6746
QCR9p_2	RPOL_7	GOT1p2_8	4	2	11	2	4	4	7	7	24	7	3	17	2	3	11	5	11	9	12	9	11	7	9	20	20	11	37	269	21.6746
ME2ad_3	RISP_8	l06422_10	8	2	14	5	5	13	6	17	17	0	1	0	0	2	1	2	0	4	8	5	21	16	8	24	14	22	53	268	21.6335
X14140_3	RPOL_7	cytMDH_10	9	4	4	0	7	16	11	9	26	0	0	5	0	2	3	1	1	3	16	11	27	9	7	18	23	17	39	268	21.6195
l22708_3	l34449_5	RPOL_7	7	4	7	2	5	12	8	12	31	0	1	1	0	2	2	3	0	1	8	4	17	19	7	17	29	27	42	268	21.6094
P060_2	CYCad_6	l06422_10	3	5	9	1	5	11	8	1	17	0	8	10	2	4	8	17	6	17	7	6	18	4	9	18	18	19	39	270	21.5618
P060_2	QCR7p_6	l06422_10	3	5	9	1	5	11	8	1	17	0	8	10	2	4	8	17	6	17	7	6	18	4	9	18	18	19	39	270	21.5618
X14140_3	GOT1Srg_5	CYCad_6	1	9	11	4	3	10	8	11	30	1	0	2	0	3	1	5	1	2	7	8	17	12	6	28	26	20	42	268	21.5467
X14140_3	GOT1Srg_5	QCR7p_6	1	9	11	4	3	10	8	11	30	1	0	2	0	3	1	5	1	2	7	8	17	12	6	28	26	20	42	268	21.5467
P060_2	l34449_5	l06422_10	3	1	2	3	4	7	5	6	28	4	1	10	7	8	5	8	9	20	4	2	21	8	7	17	17	24	37	268	21.539
X14140_3	RPOL_7	QCR6p_9	3	4	10	7	2	14	8	6	33	4	1	0	0	1	4	0	0	5	12	11	31	7	8	19	13	14	52	269	21.4868
X14140_3	CYCad_6	cytMDH_10	1	3	8	7	5	11	12	12	27	0	0	6	0	2	2	1	1	3	15	11	19	4	11	19	29	13	46	268	21.4702
X14140_3	QCR7p_6	cytMDH_10	1	3	8	7	5	11	12	12	27	0	0	6	0	2	2	1	1	3	15	11	19	4	11	19	29	13	46	268	21.4702
CYC1_4	GOT1Srg_5	l06422_10	4	0	9	4	7	12	6	13	16	5	0	12	4	0	4	8	5	16	4	5	17	12	12	12	13	21	49	270	21.4014
GDHad_1	ME2ad_3	P169_4	8	6	9	1	1	3	9	11	30	2	8	6	0	0	2	7	1	12	22	11	15	0	0	3	31	23	48	269	21.3807
mtMDH_2	l34449_5	l06422_10	3	1	2	3	4	7	5	6	28	4	1	10	7	8	5	8	9	20	4	2	21	8	7	17	17	24	38	269	21.2518
X14140_3	RISP_8	l06422_10	9	2	12	6	3	13	5	17	19	0	1	0	1	3	1	2	1	6	7	5	23	14	9	23	15	21	48	266	21.2133
QCR9p_2	GOT1Srg_5	RPOL_7	4	0	10	4	3	10	9	7	18	8	0	7	7	3	8	13	13	9	8	9	11	9	10	12	15	17	45	269	21.0898
P169_4	l34449_5	ME1ad_12	3	7	5	4	9	4	14	7	27	3	5	4	5	1	8	9	10	16	2	7	13	12	7	15	10	19	42	268	21.0858
GDHad_1	X14140_3	ME1ad_12	4	4	16	2	2	3	12	9	27	3	8	6	2	0	0	2	5	11	15	12	19	0	4	2	22	28	51	269	21.0822

AD 20˚

			Three Locus combinations (first/second/third locus genotype)																													Three Locus Totals	
first_locus	second_locus	third_locus	AA AA AA	AA AA DD	AA AA AD	AA DD AA	AA DD DD	AA DD AD	AA AD AA	AA AD DD	AA AD AD	DD AA AA	DD AA DD	DD AA AD	DD DD AA	DD DD DD	DD DD AD	DD AD AA	DD AD DD	DD AD AD	AD AA AA	AD AA DD	AD AA AD	AD DD AA	AD DD DD	AD DD AD	AD AD AA	AD AD DD	AD AD AD	Total #	chi-square value	Chromosome Combos	# p<0.05
CYCad_6	RPOL_7	cytMDH_10	8	15	11	10	1	8	5	6	25	2	2	6	4	1	2	5	3	18	13	9	30	1	8	14	8	21	48	284	36.3255	1,2,3	2
QCR7p_6	RPOL_7	cytMDH_10	8	15	11	10	3	9	5	6	25	2	2	6	4	0	2	5	3	18	13	9	30	1	8	14	8	21	48	286	34.1268	1,2,10	2
P169_4	QCR6p_9	cytMDH_10	4	7	4	7	3	7	8	8	27	0	0	0	0	4	0	0	0	1	8	8	19	3	11	19	26	25	85	284	33.9151	1,2,12	3
X14140_3	QCR10p_4	GOT2_8	13	5	17	2	4	4	11	7	19	1	0	4	4	2	0	4	7	6	3	9	20	12	1	11	31	18	62	277	32.4837	1,3,4	8
P169_4	CYCad_6	l06422_10	10	1	9	0	1	8	10	16	21	0	3	0	1	0	0	0	1	0	14	20	33	12	4	18	18	23	62	285	32.198	1,3,10	8
P169_4	QCR7p_6	l06422_10	10	1	9	0	1	7	10	16	21	0	3	0	1	0	0	0	1	0	14	21	35	12	4	18	18	23	61	286	31.6555	1,3,12	2
X14140_3	QCR10p_4	ME1ad_12	7	5	23	3	3	4	17	7	12	1	2	2	0	0	6	3	3	11	7	13	11	5	7	12	30	17	64	275	31.1246	1,4,5	2
X14140_3	CYC1_4	GOT2_8	13	6	16	3	6	5	11	5	19	1	0	6	4	1	2	4	8	2	6	9	22	11	5	22	30	16	52	285	30.6282	1,4,9	1
GDHad_1	QCR9p_2	ME1ad_12	4	5	8	3	1	17	19	11	18	2	2	1	4	0	4	0	2	5	19	7	17	8	13	30	14	21	48	283	30.479	1,4,10	1
RISP_8	QCR6p_9	l06422_10	4	5	6	1	8	3	15	5	22	2	0	14	2	3	5	10	11	15	2	7	10	8	7	17	21	24	56	283	30.2738	1,5,8	2
X14140_3	QCR10p_4	GOT1p2_8	13	5	17	2	3	5	10	6	21	1	0	4	4	2	0	5	7	5	3	8	21	9	2	13	25	15	69	275	30.1675	1,5,9	1
X14140_3	QCR10p_4	GOT1p1_8	13	5	17	2	3	5	10	6	21	1	0	4	4	2	0	5	7	5	3	8	21	9	2	13	25	16	69	276	29.9756	1,6,10	2
QCR9p_2	ME2ad_3	P169_4	9	0	13	3	0	5	9	2	23	12	0	5	0	1	4	12	0	45	13	2	27	4	0	9	12	0	70	280	29.7069	1,7,10	1
QCR9p_2	l22708_3	P169_4	9	0	13	3	0	5	9	2	23	12	0	5	0	1	4	12	0	45	13	2	28	4	0	9	12	0	69	280	29.5621	1,8,9	3
GDHad_1	l22708_3	cytMDH_10	5	10	16	1	2	3	5	5	41	3	0	2	0	3	2	1	7	2	10	8	28	4	4	7	27	27	61	284	29.4529	1,8,10	3
GDHad_1	QCR8p_3	ME1ad_12	16	10	11	0	0	3	10	7	29	0	1	4	3	0	1	3	3	5	19	9	21	4	3	9	18	29	66	284	28.9613	1,9,10	2
QCR9p_2	QCR8p_3	P169_4	8	0	19	4	0	4	9	2	20	11	0	7	0	1	4	13	0	44	11	2	34	3	0	7	15	0	66	284	28.7238	2,3,4	11
P169_4	QCR6p_9	l06422_10	1	4	10	3	4	10	16	10	17	0	0	0	0	4	0	1	0	0	7	8	20	7	10	15	29	30	77	283	28.5712	2,3,5	2
ME2ad_3	QCR10p_4	ME1ad_12	7	4	21	2	4	3	18	5	15	1	1	3	0	0	5	5	3	8	7	15	12	6	6	14	27	19	63	274	28.3034	2,3,10	1
GDHad_1	l22708_3	l06422_10	7	6	18	2	4	0	14	15	23	3	0	2	1	4	0	0	1	9	10	9	27	3	5	7	25	26	62	283	28.2521	2,3,12	7
l22708_3	P169_4	ME1ad_12	9	3	22	2	0	0	16	12	17	2	1	4	0	0	1	5	3	10	8	13	12	0	1	1	31	28	79	280	28.0614	2,4,6	2
X14140_3	CYC1_4	GOT1p1_8	14	6	16	3	3	8	10	5	20	2	0	5	4	1	2	4	8	2	5	8	24	8	5	25	25	15	57	285	27.6889	2,4,10	3
ME2ad_3	P169_4	ME1ad_12	9	3	22	2	0	0	16	11	17	2	1	4	0	0	1	4	3	11	8	13	12	0	1	1	32	29	78	280	27.4811	2,5,9	1
QCR10p_4	l34449_5	GOT2_8	5	2	4	3	3	18	9	9	19	3	0	7	7	1	1	8	6	7	12	10	18	7	9	16	27	14	54	279	27.4604	2,6,8	8
mtMDH_2	QCR8p_3	ME1ad_12	13	7	6	2	2	2	8	5	16	2	4	11	1	1	4	14	10	31	20	10	19	4	0	7	9	24	53	285	27.3442	2,8,12	2
GOT1Srg_5	QCR7p_6	GOT1p2_8	6	2	16	2	5	3	10	3	17	8	1	13	5	1	2	4	13	21	9	4	34	6	5	13	24	17	39	283	27.3378	3,4,5	1
GDHad_1	P102_3	ME1ad_12	16	9	10	0	0	4	10	8	29	0	1	4	3	0	0	3	3	6	18	13	26	4	3	9	19	25	61	284	27.274	3,4,8	29
GDHad_1	ME2ad_3	cytMDH_10	5	10	16	1	2	4	5	5	40	3	0	2	0	2	2	1	8	3	10	7	28	4	4	8	27	28	60	285	27.2521	3,4,10	6
CYCad_6	GOT1p1_8	cytMDH_10	7	8	7	1	2	4	15	13	33	8	0	7	3	3	5	2	2	14	7	8	23	3	7	25	12	23	44	286	27.1866	3,4,12	9
QCR10p_4	GOT1Srg_5	RISP_8	4	2	6	5	6	15	4	12	18	5	0	5	8	2	1	4	5	10	9	11	21	6	8	16	23	13	58	277	27.1818	3,5,9	1
l22708_3	QCR10p_4	GOT1p2_8	12	4	17	1	3	4	9	7	24	1	0	4	4	1	1	5	6	4	4	9	21	10	3	13	26	15	67	275	27.1668	3,6,8	1
ME2ad_3	QCR10p_4	GOT1p1_8	12	4	16	2	3	4	9	7	23	1	0	4	4	1	0	5	7	5	4	9	22	9	3	14	26	16	66	276	26.9992	3,6,9	2
P060_2	QCR8p_3	ME1ad_12	13	7	7	2	2	2	8	5	15	2	4	11	1	1	4	14	10	31	20	9	18	4	0	6	9	23	52	280	26.7976	3,8,12	1
GOT1Srg_5	QCR7p_6	GOT1p1_8	7	2	16	2	5	3	10	3	17	8	1	13	5	1	2	4	13	21	9	5	33	6	5	13	24	18	39	285	26.7149	4,5,8	4
l22708_3	QCR10p_4	GOT1p1_8	12	4	17	1	3	4	9	7	24	1	0	4	4	1	1	5	6	4	4	9	21	10	3	13	26	17	66	276	26.537	4,5,9	2
X14140_3	CYC1_4	GOT1p2_8	13	6	16	3	3	8	10	5	20	2	0	5	4	1	2	4	8	2	5	7	24	8	5	25	25	15	57	283	26.4379	4,5,10	1
GDHad_1	l22708_3	QCR10p_4	13	4	14	1	1	4	8	4	36	4	0	1	0	3	2	2	5	3	16	4	25	4	2	9	24	17	71	277	26.4142	4,6,8	1
l22708_3	QCR10p_4	ME1ad_12	7	5	21	2	4	2	18	5	16	1	1	3	1	0	5	5	3	7	7	14	12	5	6	15	27	19	63	274	26.2821	4,6,10	4
P060_2	QCR7p_6	GOT1p2_8	4	2	8	1	2	2	10	11	21	11	3	11	4	2	7	7	11	22	8	2	43	8	7	9	21	11	34	282	26.2313	4,7,9	1
GDHad_1	CYCad_6	cytMDH_10	7	3	17	0	2	13	4	11	30	1	6	1	1	0	3	2	4	3	15	14	26	10	3	11	16	23	60	286	26.1397	4,8,10	3
P060_2	CYCad_6	GOT1p2_8	3	2	8	1	2	2	10	11	21	11	3	11	4	2	7	7	11	22	8	2	41	8	8	9	21	11	35	281	26.0984	4,8,12	1
GOT1Srg_5	CYCad_6	GOT1p2_8	6	2	16	2	5	3	10	3	17	7	1	13	5	1	2	4	13	21	9	4	32	6	5	13	24	17	40	281	26.061	4,9,10	3
QCR9p_2	QCR8p_3	ME1ad_12	14	5	7	4	2	2	7	7	17	3	3	12	2	1	2	10	10	37	18	13	17	1	0	9	14	22	45	284	25.9759	5,6,8	7
GDHad_1	QCR6p_9	l06422_10	6	8	5	5	5	5	12	12	30	0	1	6	2	2	1	2	2	4	2	3	19	4	11	19	32	26	60	284	25.8308	5,7,8	2
GDHad_1	X14140_3	cytMDH_10	5	10	14	1	1	5	5	6	41	3	1	2	0	1	2	1	8	2	11	8	30	6	4	8	24	28	57	284	25.7461	5,7,12	1
QCR10p_4	l34449_5	RISP_8	5	2	4	4	6	14	4	12	21	4	0	6	7	1	1	6	6	9	9	10	21	5	8	18	24	14	57	278	25.721	6,7,10	2
GOT1Srg_5	CYCad_6	GOT1p1_8	6	2	16	3	5	3	10	3	17	7	1	13	5	1	2	4	13	21	9	4	32	6	5	13	24	19	39	283	25.7054	6,8,10	4
ME2ad_3	QCR10p_4	GOT2_8	12	4	16	3	3	3	10	8	21	1	0	4	4	1	0	4	7	6	4	10	21	11	3	12	32	18	59	277	25.6673	8,9,10	3
ME2ad_3	QCR10p_4	GOT1p2_8	12	4	16	2	3	4	9	7	23	1	0	4	4	1	0	5	6	6	4	9	22	9	3	14	26	15	66	275	25.3662		
QCR10p_4	GOT1Srg_5	GOT2_8	5	2	5	4	4	18	8	8	18	5	0	5	8	1	2	5	6	8	14	10	17	7	9	15	25	14	55	278	25.3034		
GDHad_1	QCR8p_3	P169_4	10	1	26	2	1	0	13	0	35	4	0	1	0	0	4	1	1	9	16	1	32	5	0	11	24	1	88	286	25.3025		
P102_3	CYCad_6	QCR6p_9	7	4	22	2	1	11	6	15	30	0	5	4	2	1	1	0	0	9	8	7	33	4	4	16	20	18	54	284	25.2775		
mtMDH_2	CYCad_6	GOT1p2_8	2	2	8	1	2	2	10	11	21	11	3	11	4	2	7	7	11	22	9	2	42	8	7	9	21	11	36	282	25.1998		
mtMDH_2	QCR7p_6	GOT1p2_8	3	2	8	1	2	2	10	11	21	11	3	11	4	2	7	7	11	22	9	2	43	8	7	9	21	11	36	284	25.1251		
GDHad_1	QCR8p_3	cytMDH_10	4	9	24	1	1	1	6	7	35	3	0	2	0	2	2	1	8	2	10	10	29	5	4	7	26	26	61	286	25.0936		
GDHad_1	l22708_3	P169_4	12	1	18	3	1	2	10	0	41	4	0	1	0	0	5	1	1	8	18	1	27	4	0	11	23	1	91	284	25.0727		
GDHad_1	l34449_5	QCR6p_9	3	2	8	4	1	16	12	12	30	0	1	5	1	3	0	6	1	3	3	10	30	7	5	29	14	20	59	285	25.0172		
mtMDH_2	CYCad_6	GOT1p1_8	2	2	8	1	2	2	10	11	21	11	3	11	4	2	7	7	11	22	9	2	42	8	7	9	21	12	36	283	24.9132		
P060_2	CYCad_6	GOT1p1_8	3	2	8	1	2	2	10	11	21	11	3	11	4	2	7	7	11	22	8	2	41	8	7	9	21	13	34	281	24.8507		
QCR8p_3	P169_4	ME1ad_12	10	3	17	2	0	0	23	17	19	2	1	4	0	0	1	5	2	8	7	13	17	0	1	1	24	25	81	283	24.8389		
mtMDH_2	QCR7p_6	GOT1p1_8	3	2	8	1	2	2	10	11	21	11	3	11	4	2	7	7	11	22	9	2	43	8	7	9	21	12	36	285	24.8388		
l22708_3	QCR10p_4	GOT2_8	12	4	17	2	3	3	10	8	22	1	0	4	4	1	1	4	6	5	4	10	20	12	3	11	32	19	59	277	24.8095		
l34449_5	RPOL_7	ME1ad_12	9	7	7	3	2	2	4	4	23	6	4	13	5	3	7	11	10	7	11	7	31	6	10	12	18	14	47	283	24.6437		
GDHad_1	QCR6p_9	cytMDH_10	4	5	10	4	2	9	3	10	40	0	4	3	1	3	1	3	3	2	8	6	10	5	13	17	28	20	71	285	24.617		
P169_4	CYCad_6	cytMDH_10	9	2	9	2	1	6	8	15	24	0	3	0	0	0	1	0	1	0	14	18	35	13	5	20	14	22	68	290	24.504		
GOT1Srg_5	RPOL_7	GOT2_8	5	4	15	5	1	1	14	7	12	7	10	10	5	0	8	7	5	17	17	5	23	7	7	16	16	16	44	284	24.4207		
GOT1Srg_5	RPOL_7	RISP_8	3	7	14	4	1	2	11	5	17	6	11	10	4	0	9	9	5	14	12	6	27	6	11	13	16	15	45	283	24.4006		
GDHad_1	QCR8p_3	QCR10p_4	12	5	19	1	1	1	9	3	34	4	0	1	0	2	2	2	6	3	14	5	29	5	2	9	25	16	69	279	24.3606		
P060_2	QCR7p_6	GOT1p1_8	4	2	8	1	2	2	10	11	21	11	3	11	4	2	7	7	11	22	8	3	42	8	7	9	21	12	34	283	24.2489		
QCR7p_6	GOT1p2_8	cytMDH_10	7	9	7	1	2	4	15	13	35	6	0	7	3	3	5	2	2	14	7	8	23	3	7	23	12	23	44	285	24.099		

GDHad_1	mtMDH_2	cytMDH_10	1	9	12	3	2	14	7	6	34	0	0	1	1	5	1	3	5	4	11	8	19	15	13	24	15	19	54	286	24.0862
GDHad_1	QCR9p_2	QCR8p_3	11	0	6	7	1	13	19	2	28	0	3	2	1	1	6	4	0	3	15	5	23	10	3	38	24	8	51	284	23.9997
QCR10p_4	RISP_8	cytMDH_10	3	3	7	8	4	8	10	8	21	8	3	6	0	2	5	2	7	7	6	8	24	5	5	22	11	26	59	278	23.9697
GDHad_1	QCR7p_6	cytMDH_10	7	4	17	0	2	13	4	11	30	1	6	1	1	0	2	2	4	3	15	14	28	10	3	11	16	23	59	287	23.9395
GDHad_1	GOT2_8	cytMDH_10	3	5	14	2	4	11	6	8	35	1	6	3	0	0	3	3	4	0	18	8	25	7	10	19	16	22	54	287	23.9316
QCR7p_6	GOT1p1_8	cytMDH_10	8	9	7	1	2	5	15	13	34	6	0	7	3	3	5	2	2	14	7	8	23	3	7	24	12	23	44	287	23.9145
QCR9p_2	P102_3	P169_4	5	0	20	3	0	3	13	2	20	11	0	12	0	1	5	13	0	38	12	2	37	2	0	9	15	0	61	284	23.913
GDHad_1	QCR8p_3	l06422_10	9	9	19	2	1	0	12	15	22	3	0	2	1	3	0	0	2	9	12	8	29	4	6	6	22	26	63	285	23.8213
ME2ad_3	CYC1_4	GOT2_8	12	5	15	3	6	7	10	5	18	1	1	6	4	0	1	4	6	3	7	9	23	11	6	20	31	18	52	284	23.7389
l34449_5	CYCad_6	GOT1p2_8	5	2	16	1	5	3	9	5	17	4	1	12	6	1	3	4	12	20	13	4	33	6	6	12	25	15	43	283	23.7222
GDHad_1	P102_3	cytMDH_10	4	9	24	1	1	2	6	7	34	3	0	2	0	2	1	1	8	3	10	11	37	6	4	6	25	25	55	287	23.6757
X14140_3	CYC1_4	ME1ad_12	9	5	21	5	6	3	13	6	15	2	2	3	0	0	7	2	3	9	8	12	16	7	11	20	27	16	54	282	23.6362
GDHad_1	P060_2	cytMDH_10	1	9	12	3	2	14	7	6	33	0	0	1	1	5	1	3	5	5	11	8	19	15	13	24	15	17	54	284	23.6009
GDHad_1	ME2ad_3	QCR10p_4	12	5	14	1	1	5	9	3	35	4	0	1	0	2	2	2	6	3	16	4	24	4	2	10	24	17	71	277	23.5269
QCR10p_4	QCR6p_9	cytMDH_10	5	6	4	7	3	7	9	6	25	2	3	4	0	5	2	8	4	12	5	5	15	2	10	17	15	23	73	277	23.5122
P102_3	QCR7p_6	QCR6p_9	7	4	23	2	1	11	6	15	29	1	5	4	2	1	1	0	0	9	8	7	34	4	4	16	20	18	54	286	23.4913
mtMDH_2	l22708_3	P169_4	6	0	17	2	0	4	9	0	23	11	0	6	1	1	4	10	0	44	17	2	23	4	0	10	15	2	72	283	23.3964
QCR9p_2	QCR8p_3	QCR10p_4	7	2	16	4	1	3	7	4	20	12	2	4	0	3	2	11	9	34	11	6	29	2	1	7	17	12	51	277	23.3668
X14140_3	QCR10p_4	l34449_5	5	8	22	1	1	8	12	7	18	0	1	4	1	1	4	3	4	10	6	15	11	8	7	9	25	20	66	277	23.2818
ME2ad_3	P169_4	GOT1p1_8	10	4	20	0	0	2	13	10	22	1	2	4	1	0	0	8	6	5	2	6	26	0	0	2	39	23	77	283	23.2653
QCR9p_2	QCR10p_4	cytMDH_10	1	8	9	1	2	4	6	8	25	9	1	13	3	6	5	6	9	25	11	6	13	6	4	9	10	22	54	276	23.2195
l22708_3	P169_4	l06422_10	9	7	18	1	1	0	10	7	29	4	3	0	0	1	0	2	9	7	7	8	19	0	2	0	31	32	75	282	23.0387
QCR8p_3	GOT2_8	ME1ad_12	6	5	13	4	7	5	25	8	18	3	1	4	1	1	3	3	1	6	10	8	33	10	6	18	11	25	49	284	23.0354
P060_2	QCR10p_4	cytMDH_10	2	5	9	2	0	5	8	11	17	8	2	11	4	5	5	5	13	22	11	6	16	4	7	8	9	15	65	275	22.95
QCR8p_3	CYC1_4	l06422_10	8	7	14	3	6	9	13	4	27	5	2	0	0	2	3	2	6	3	6	12	25	10	9	16	18	22	53	285	22.9356
X14140_3	P169_4	ME1ad_12	9	5	21	2	0	0	16	12	18	2	2	5	0	0	0	2	3	14	8	10	12	0	1	2	34	28	75	281	22.9152
GDHad_1	GOT1p2_8	cytMDH_10	3	5	13	1	3	11	7	9	36	1	6	3	0	0	3	3	4	1	16	6	21	6	9	19	19	25	56	286	22.8388
l22708_3	CYC1_4	GOT2_8	12	5	16	2	6	7	10	5	19	1	1	5	4	0	2	4	6	3	7	9	23	12	6	19	31	18	51	284	22.8246
l22708_3	P169_4	GOT1p2_8	10	4	20	0	0	2	12	10	24	1	2	4	1	0	0	8	5	5	2	6	26	0	0	2	40	22	76	282	22.8213
P169_4	GOT1Srg_5	QCR6p_9	3	1	10	7	7	11	5	9	22	0	3	0	0	0	0	0	1	1	6	9	32	7	5	32	22	19	72	284	22.7752
l22708_3	P169_4	GOT1p1_8	10	4	20	0	0	2	12	10	24	1	2	4	1	0	0	8	5	5	2	6	26	0	0	2	40	24	75	283	22.7539
ME2ad_3	P169_4	l06422_10	9	7	18	1	1	0	9	7	29	4	3	0	0	1	0	2	8	9	7	8	20	0	2	0	32	33	73	283	22.7504
CYC1_4	CYCad_6	RISP_8	5	3	11	3	8	2	7	9	32	7	7	7	2	0	6	8	8	14	13	9	29	7	4	11	18	15	40	285	22.7186
GDHad_1	mtMDH_2	ME1ad_12	6	7	9	4	2	13	16	8	21	0	0	1	4	0	3	2	4	6	17	7	14	9	13	30	15	21	52	284	22.6838
GDHad_1	P060_2	ME1ad_12	6	7	9	4	2	13	16	7	21	0	0	1	4	0	3	2	4	6	17	7	14	9	13	30	15	21	49	280	22.6799
mtMDH_2	ME2ad_3	P169_4	6	0	16	2	0	4	9	0	24	11	0	6	1	1	4	10	0	44	17	2	23	4	0	10	15	2	72	283	22.6789
P169_4	QCR7p_6	cytMDH_10	9	2	9	2	1	5	8	15	24	0	3	0	0	0	1	0	1	0	15	20	37	10	4	20	14	22	67	289	22.6761
QCR10p_4	GOT1p2_8	l06422_10	2	5	10	2	2	9	15	8	19	1	5	9	1	2	4	6	8	4	15	6	19	7	8	14	15	23	57	276	22.6714
X14140_3	QCR10p_4	RISP_8	8	6	21	3	3	4	10	9	18	1	2	2	2	2	2	1	5	11	4	12	16	12	2	10	27	18	66	277	22.6412
GDHad_1	QCR10p_4	cytMDH_10	5	4	13	1	2	6	5	10	38	3	1	2	1	5	2	0	4	2	13	10	21	8	5	10	17	25	66	279	22.5941
P060_2	l22708_3	P169_4	6	0	17	2	0	4	9	0	24	11	0	6	1	1	4	10	0	44	16	2	23	3	0	10	15	2	71	281	22.5938
GDHad_1	QCR10p_4	GOT1Srg_5	3	4	15	1	2	6	10	14	29	2	1	3	2	3	3	1	1	4	7	21	16	7	6	10	30	16	61	278	22.5808
GDHad_1	ME2ad_3	l06422_10	6	7	18	2	4	1	15	14	22	3	0	2	1	3	0	0	2	10	10	8	27	3	5	8	25	27	61	284	22.5132
QCR10p_4	RPOL_7	QCR6p_9	3	9	13	1	3	9	11	5	18	4	5	3	0	1	4	5	1	17	6	8	41	7	6	18	12	15	52	277	22.5053
l22708_3	P169_4	RISP_8	6	7	21	1	0	1	14	12	20	1	4	2	1	0	0	3	2	13	4	11	19	1	0	1	40	25	75	284	22.4821
X14140_3	P169_4	l06422_10	10	7	18	1	1	0	7	10	30	4	4	1	0	0	0	3	6	10	6	7	18	0	3	0	33	32	71	282	22.4196
P060_2	P102_3	ME1ad_12	12	7	6	1	2	2	10	5	16	3	6	13	2	1	5	12	8	28	19	10	20	4	0	5	10	22	51	280	22.3505
GDHad_1	QCR9p_2	ME2ad_3	8	0	9	6	2	13	17	5	27	0	3	2	1	0	7	4	1	2	14	5	23	10	3	37	21	7	54	281	22.3496
ME2ad_3	CYC1_4	ME1ad_12	9	4	19	4	7	5	14	3	15	3	1	4	0	0	5	3	3	7	7	14	17	8	10	19	25	19	56	281	22.2877
ME2ad_3	P169_4	GOT1p2_8	10	4	20	0	0	2	13	10	22	1	2	4	1	0	0	8	5	6	2	7	26	0	0	2	39	22	77	283	22.2648
CYCad_6	GOT1p2_8	cytMDH_10	7	8	7	1	2	4	15	13	33	6	0	7	3	3	6	2	2	14	7	8	23	3	7	23	12	23	45	284	22.2573
X14140_3	P169_4	RISP_8	5	7	23	1	0	1	17	13	19	1	5	3	0	0	0	3	4	12	5	10	16	2	0	1	38	23	77	286	22.2566
P060_2	QCR8p_3	P169_4	6	0	22	3	0	3	8	0	21	10	0	7	1	1	4	11	0	44	14	2	31	2	0	8	18	2	65	283	22.1967
mtMDH_2	P102_3	ME1ad_12	12	7	6	1	2	2	10	5	16	3	6	13	2	1	5	12	8	28	19	11	21	4	0	6	10	23	52	285	22.1242
GDHad_1	GOT1Srg_5	GOT1p2_8	4	0	10	5	5	10	12	10	31	0	0	5	3	0	2	7	2	1	14	10	21	9	10	24	20	14	54	283	22.0349
GDHad_1	GOT1p2_8	QCR6p_9	5	6	10	1	3	11	13	6	32	5	3	2	0	0	2	2	2	4	4	7	32	7	6	21	13	21	65	283	22.0226
QCR10p_4	GOT1p1_8	l06422_10	2	5	10	2	2	9	15	8	19	1	5	9	1	2	4	6	8	4	15	6	19	7	8	16	15	23	56	277	22.0196
mtMDH_2	ME2ad_3	ME1ad_12	10	5	7	1	3	2	12	6	14	3	3	11	0	1	5	14	10	30	14	6	21	5	0	9	14	27	48	281	21.9955
QCR9p_2	l22708_3	QCR10p_4	6	2	14	3	1	4	9	4	20	12	2	3	0	3	2	11	9	34	15	4	23	2	2	9	13	13	54	274	21.9934
QCR9p_2	CYC1_4	QCR7p_6	5	0	14	3	1	4	11	5	22	6	8	11	7	2	7	11	3	25	7	4	22	11	5	19	30	14	27	284	21.9813
mtMDH_2	QCR10p_4	cytMDH_10	2	5	8	2	0	5	8	11	18	8	2	11	4	5	5	5	13	22	11	8	17	4	7	8	9	15	65	278	21.9683
GDHad_1	GOT1p1_8	cytMDH_10	3	5	13	1	3	11	7	9	36	1	6	3	0	0	2	3	4	1	16	6	21	6	9	21	19	25	55	286	21.9627
GDHad_1	GOT1p1_8	QCR6p_9	5	6	10	1	3	11	13	6	32	5	3	2	0	0	2	2	2	4	4	7	32	7	7	22	13	21	64	284	21.924
X14140_3	P169_4	GOT1p2_8	10	4	21	0	0	2	16	10	21	1	3	5	0	0	0	9	6	4	2	5	24	1	0	2	35	21	80	282	21.8716
l34449_5	QCR7p_6	GOT1p2_8	5	2	16	1	4	3	9	5	17	5	1	13	6	1	3	4	12	20	13	4	34	6	6	12	25	15	42	284	21.7877
X14140_3	P169_4	GOT1p1_8	10	4	21	0	0	2	17	10	21	1	3	5	0	0	0	9	6	4	2	5	24	1	0	2	35	22	80	284	21.7662
QCR10p_4	GOT2_8	ME1ad_12	2	4	11	5	3	6	8	13	19	3	1	14	0	2	5	5	7	3	14	8	24	10	7	15	26	13	49	277	21.7395
GDHad_1	RPOL_7	cytMDH_10	6	4	19	3	3	8	2	9	33	2	7	1	0	0	0	2	3	5	15	15	27	12	7	17	14	18	53	285	21.7358
mtMDH_2	GOT1p1_8	ME1ad_12	5	4	5	5	3	6	13	7	11	4	2	16	5	7	4	8	6	26	9	6	23	4	2	15	20	25	41	282	21.7341
P169_4	GOT1Srg_5	cytMDH_10	5	1	8	8	5	12	6	12	18	0	3	0	0	0	0	0	1	1	11	11	26	5	11	28	22	22	69	285	21.7201
GDHad_1	P169_4	l34449_5	2	6	17	0	0	2	11	15	35	4	1	1	1	0	0	2	3	9	8	17	20	1	0	1	34	24	73	287	21.7086
GDHad_1	P169_4	QCR6p_9	5	2	18	0	1	1	14	11	35	1	1	3	0	1	0	6	3	5	9	14	22	0	2	0	15	19	97	285	21.6749
QCR10p_4	GOT1Srg_5	QCR6p_9	2	1	9	7	7	12	6	9	19	0	3	7	5	2	4	4	2	13	7	9	25	2	3	26	16	17	60	277	21.6723
P060_2	ME2ad_3	P169_4	6	0	16	2	0	4	9	0	25	11	0	6	1	1	4	10	0	44	16	2	23	3	0	11	16	2	70	282	21.6723
ME2ad_3	CYC1_4	GOT1p1_8	12	5	15	2	3	11	9	6	18	2	1	5	4	0	1	4	6	3	6	8	25	9	6	22	26	16	58	283	21.6016
X14140_3	l34449_5	QCR6p_9	0	3	15	0	4	12	14	7	28	1	1	2	0	1	5	2	2	14	5	9	26	12	4	27	16	24	49	283	21.5456
mtMDH_2	P102_3	GOT1Srg_5	3	7	16	0	1	4	9	8	14	4	11	7	4	2	2	13	12	23	16	9	25	2	3	5	13	16	56	285	21.5346
ME2ad_3	CYC1_4	GOT1p2_8	12	5	15	2	3	11	9	6	18	2	1	5	4	0	1	4	6	3	6	7	25	9	6	22	26	16	58	282	21.5339
mtMDH_2	QCR8p_3	GOT1Srg_5	4	6	16	0	1	5	8	9	13	3	8	6	3	2	1	15	15	25	14	10	24	3	4	4	14	14	58	285	21.4671
GOT2_8	QCR6p_9	l06422_10	2	7	8	4	9	5	17	6	24	1	0	10	1	2	7	7	11	18	5	5	12	6	7	13	22	23	52	284	21.4387
P060_2	ME2ad_3	ME1ad_12	10	5	7	1	3	2	12	6	15	3	3	11	0	1	5	14	10	30	14	6	20	5	0	8	14	26	47	278	21.3948
GDHad_1	GOT2_8	QCR6p_9	6	7	9	2	3	13	11	5	32	5	3	2	1	0	2	1	2	4	6	9	36	8	7	21	10	19	62	286	21.3927
mtMDH_2	GOT1p2_8	ME1ad_12	5	4	5	5	3	6	13	7	11	4	2	16	5	7	4	8	6	26	9	6	23	4	2	14	20	25	41	281	21.3873
X14140_3	P169_4	GOT2_8	10	5	20	0	0	2	18	12	18	1	3	5	0	0	0	8	6	5	3	6	22	2	0	1	42	23	73	285	21.3773
GDHad_1	GOT1Srg_5	GOT1p1_8	4	0	10	5	5	10	12	10	31	0	0	5	3	0	2	7	2	1	14	10	21	9	10	24	20	16	53	284	21.3661
l22708_3	CYC1_4	l06422_10	9	8	16	3	3	9	8	4	22	4	2	1	0	3	3	2	8	3	6	11	22	10	11	15	23	20	56	282	21.2683
ME2ad_3	P169_4	RISP_8	6	7	21	1	0	1	14	11	20	1	4	2	1	0	0	3	3	13	4	11	19	1	0	1	40	25	75	284	21.2487
QCR9p_2	CYC1_4	CYCad_6	5	0	14	3	1	4	11	5	22	6	8	11	7	2	7	11	3	25	7	4	22	11	5	19	28	14	27	282	21.2467
QCR8p_3	P169_4	l06422_10	8	5	17	1	1	0	15	11	33	5	2	0	0	1	0	2	7	6	7	11	20	0	2	0	26	30	74	284	21.2362
GDHad_1	X14140_3	QCR10p_4	11	5	12	1	0	6	10	4	36	4	1	1	0	2	1	2	5	4	20	4	24	4	4	10	20	15	71	277	21.2166
mtMDH_2	GOT1Srg_5	QCR6p_9	2	1	9	1	1	14	4	6	24	4	6	11	10	4	11	4	6	22	3	6	22	3	7	18	19	18	48	284	21.2099
GDHad_1	ME2ad_3	P169_4	12	1	18	3	1	3	10	0	40	4	0	1	0	0	4	2	1	9	18	1	26	4	0	12	23	1	91	285	21.1434
l22708_3	CYC1_4	GOT1p1_8	12	5	16	1	3	11	9	6	19	2	1	4	4	0	2	4	6	3	6	8	25	10	6	21	26	16	57	283	21.1234
GDHad_1	P102_3	QCR10p_4	11	5	19	1	1	2	10	3	33	4	0	1	0	1	2	2	7	3	15	7	35	3	2	11	26	14	62	280	21.1141
X14140_3	CYCad_6	GOT1p1_8	5	2	16	5	3	3	16	9	25	3	0	3	2	0	1	5	9	5	14	5	42	7	8	14	17	15	49	283	21.0747
l22708_3	CYC1_4	GOT1p2_8	12	5	16	1	3	11	9	6	19	2	1	4	4	0	2	4	6	3	6	7	25	10	6	21	26	16	57	282	21.0549
RISP_8	QCR6p_9	cytMDH_10	3	4	8	1	5	7	14	5	23	7	4	5	2	2	6	5	6	25	2	7	10	7	11	14	15	22	64	284	21.0406
P060_2	P102_3	cytMDH_10	4	10	13	3	0	2	5	8	17	6	3	13	1	5	2	12	12	24	7	8	36	3	1	5	15	19	50	284	21.0396
l34449_5	QCR7p_6	GOT1p1_8	6	2	16	1	4	3	9	5	17	5	1	13	6	1	3	4	12	20	13	5	33	6	6	12	25	16	42	286	21.0292

AD females

			Three Locus combinations (first/second/third locus genotype)																													Three Locus Totals	
first_locus	second_locus	third_locus	AA AA AA	AA AA DD	AA AA AD	AA DD AA	AA DD DD	AA DD AD	AA AD AA	AA AD DD	AA AD AD	DD AA AA	DD AA DD	DD AA AD	DD DD AA	DD DD DD	DD DD AD	DD AD AA	DD AD DD	DD AD AD	AD AA AA	AD AA DD	AD AA AD	AD DD AA	AD DD DD	AD DD AD	AD AD AA	AD AD DD	AD AD AD	Total #	chi-square value	Chromosome Combos	# p<0.05
X14140_3	P169_4	GOT1p1_8	16	7	30	0	1	5	16	11	12	1	4	6	0	0	0	7	5	5	7	7	28	6	1	7	31	25	73	311	31.0066	1,2,10	2
X14140_3	P169_4	GOT1p2_8	16	7	30	0	1	5	15	11	12	1	4	6	0	0	0	7	5	5	7	7	28	6	1	7	31	24	73	309	30.9227	1,3,4	1
ME2ad_3	QCR10p_4	l06422_10	6	16	24	3	1	5	6	14	19	4	1	0	0	2	2	0	7	7	4	14	32	1	6	18	14	32	69	307	30.7961	1,4,5	2
l22708_3	QCR10p_4	l06422_10	7	15	24	3	1	5	6	15	20	4	1	0	0	2	2	0	7	7	3	15	32	1	6	18	14	31	67	306	30.5268	1,4,12	1
X14140_3	P169_4	GOT2_8	14	6	33	0	1	5	16	11	11	2	3	6	0	0	0	4	5	8	7	7	28	7	1	6	33	27	69	310	30.1064	1,7,10	1
P102_3	QCR7p_6	RPOL_7	6	6	27	0	3	7	13	10	25	5	1	4	0	0	1	3	1	1	25	23	20	2	7	24	32	16	56	318	29.0434	1,8,10	2
QCR10p_4	l34449_5	GOT2_8	3	2	10	5	4	20	13	13	31	1	1	6	10	1	2	5	4	8	10	10	14	5	8	22	30	17	52	307	28.9755	1,9,10	1
X14140_3	QCR10p_4	QCR7p_6	11	4	34	3	1	4	13	8	17	5	1	3	1	0	3	4	1	10	14	11	17	12	1	13	50	15	50	306	28.8685	2,3,4	4
mtMDH_2	QCR10p_4	l34449_5	1	5	22	2	5	5	7	12	20	3	12	11	1	1	8	11	6	19	11	12	24	5	7	4	16	17	61	308	28.8669	2,3,5	2
P102_3	CYCad_6	RPOL_7	6	6	26	0	3	7	13	10	25	5	0	4	0	0	1	3	1	1	25	22	20	2	7	23	32	16	55	313	28.8242	2,3,8	1
X14140_3	QCR10p_4	CYCad_6	11	4	34	3	1	4	12	8	17	5	1	3	1	0	3	4	1	10	14	11	17	12	1	13	49	15	50	304	28.8205	2,4,5	6
X14140_3	QCR10p_4	GOT2_8	13	7	29	1	2	5	15	8	15	2	1	6	2	2	0	2	5	8	6	10	26	13	2	11	28	21	65	305	28.7436	2,4,6	10
X14140_3	P169_4	RISP_8	11	8	34	1	2	3	17	11	11	1	4	6	0	0	0	3	3	11	5	11	25	4	2	8	29	25	76	311	28.6657	2,4,8	1
QCR8p_3	QCR7p_6	RPOL_7	5	6	26	0	4	9	15	10	27	6	2	4	0	0	0	3	0	2	25	22	21	2	7	23	30	17	53	319	28.5083	2,4,10	1
X14140_3	P169_4	GOT1Srg_5	10	12	31	1	0	5	8	11	19	1	1	9	0	0	0	2	3	12	8	15	19	3	8	3	28	30	70	309	28.1223	2,7,10	1
QCR8p_3	QCR10p_4	l06422_10	6	15	24	3	2	6	5	13	25	4	1	0	0	2	2	0	4	4	4	15	32	1	5	17	15	36	69	310	28.0189	3,4,5	6
ME2ad_3	P169_4	RISP_8	11	7	31	1	2	2	17	11	12	1	2	2	1	0	0	4	1	12	5	15	32	3	2	9	28	26	74	311	27.8081	3,4,6	6
X14140_3	CYC1_4	l06422_10	5	17	21	1	3	6	11	12	22	4	3	3	0	0	5	0	7	6	5	17	29	6	7	22	7	29	65	313	27.4899	3,4,7	1
QCR8p_3	CYCad_6	RPOL_7	5	6	25	0	4	9	15	10	27	6	1	4	0	0	0	3	0	2	25	21	21	2	7	22	30	17	52	314	27.1824	3,4,8	29
X14140_3	P169_4	l06422_10	10	17	26	2	2	2	5	12	21	3	5	3	0	0	0	1	5	11	2	12	28	0	6	8	16	35	79	311	27.0449	3,4,9	3
X14140_3	P169_4	QCR7p_6	12	7	34	2	2	2	14	4	20	5	1	5	0	0	0	5	1	11	14	11	17	7	1	6	57	16	57	311	26.8923	3,4,10	10
P060_2	P102_3	GOT1Srg_5	2	4	21	0	1	4	9	18	21	4	10	10	3	0	1	11	13	22	14	7	22	1	0	5	17	27	62	309	26.8215	3,4,12	1
CYC1_4	RPOL_7	cytMDH_10	10	3	14	3	6	15	5	17	32	2	7	4	4	2	2	3	9	17	7	18	21	7	13	16	17	13	47	314	26.6215	3,6,7	8
X14140_3	P169_4	CYCad_6	12	7	34	2	2	2	13	5	20	5	1	5	0	0	0	5	1	11	14	11	17	7	1	6	56	16	57	310	26.4389	3,6,9	1
X14140_3	QCR10p_4	RISP_8	11	8	30	3	3	2	14	8	16	1	2	6	1	2	1	2	3	10	4	14	23	9	2	15	25	21	69	305	26.3128	3,6,10	3
P060_2	QCR10p_4	l34449_5	2	5	21	2	5	5	7	11	21	3	12	11	1	1	8	11	6	19	10	11	23	5	7	4	16	17	60	304	26.0811	4,5,8	5
ME2ad_3	CYC1_4	l06422_10	5	17	20	2	3	7	8	11	22	4	1	1	0	1	3	0	8	5	5	19	33	5	6	22	10	29	67	314	25.9721	4,5,12	1
X14140_3	CYC1_4	QCR7p_6	8	3	32	3	0	7	17	10	18	5	1	4	2	1	2	3	0	10	18	10	23	18	4	13	42	14	45	313	25.8934	4,6,7	2
X14140_3	QCR10p_4	l06422_10	7	16	26	2	1	5	7	15	16	4	3	2	0	1	3	0	6	9	3	12	27	2	7	17	13	32	70	306	25.6524	4,7,10	2
ME2ad_3	QCR7p_6	RPOL_7	4	9	16	0	5	6	17	9	29	7	1	3	0	1	0	4	2	5	25	20	31	2	5	25	27	16	44	313	25.6047	4,8,10	2
ME2ad_3	CYCad_6	RPOL_7	4	8	16	0	5	6	17	9	29	7	1	3	0	1	0	4	2	5	25	19	31	2	5	25	27	16	44	311	25.2221	4,9,10	1
l22708_3	CYC1_4	l06422_10	6	16	20	2	3	7	8	12	23	4	1	1	0	1	3	0	8	5	4	20	33	5	6	22	10	28	65	313	25.1962	5,6,7	2
mtMDH_2	P169_4	CYCad_6	7	6	15	4	0	1	11	10	27	9	8	11	3	0	2	24	1	15	15	6	30	2	3	5	40	10	48	313	25.0319	5,7,8	2
l22708_3	P169_4	RISP_8	11	7	30	1	2	2	17	12	14	1	2	2	1	0	0	4	1	12	5	15	33	3	2	9	27	25	72	310	25.0118	5,8,12	1
mtMDH_2	P102_3	GOT1Srg_5	2	5	21	0	1	4	9	18	21	4	10	10	3	0	1	11	13	22	14	7	23	1	1	5	17	27	63	313	24.7382	6,7,8	4
X14140_3	CYC1_4	CYCad_6	8	4	32	3	0	7	16	10	18	5	1	4	2	1	2	3	0	10	18	10	23	18	4	13	41	14	45	312	24.7115	6,7,10	2
QCR10p_4	GOT1Srg_5	GOT2_8	4	2	10	5	6	21	12	11	30	4	1	4	10	1	3	2	4	9	11	9	15	6	7	21	28	19	51	306	24.664	6,8,12	4
GDHad_1	QCR9p_2	l06422_10	1	7	15	2	10	7	9	15	21	0	4	0	2	1	9	5	3	6	6	16	28	5	10	35	9	29	58	313	24.5945	8,9,10	1
X14140_3	QCR10p_4	GOT1p2_8	15	9	25	1	2	5	14	8	16	1	2	6	2	2	0	5	5	5	7	9	26	11	3	12	26	18	69	304	24.5795		
P169_4	CYCad_6	RPOL_7	9	12	10	2	6	12	23	7	26	5	1	3	0	0	3	2	3	3	22	15	37	0	5	16	23	16	51	312	24.5789		
mtMDH_2	P169_4	QCR7p_6	7	6	15	4	0	1	12	10	27	9	8	11	3	0	2	24	1	15	15	6	30	2	3	5	41	10	48	315	24.5699		
X14140_3	P169_4	l34449_5	9	13	31	1	0	5	6	7	24	1	2	8	0	0	0	3	3	11	8	13	21	1	8	5	28	31	71	310	24.5665		
X14140_3	QCR10p_4	GOT1p1_8	15	9	25	1	2	5	14	8	16	1	2	6	2	2	0	5	5	5	7	9	26	11	3	12	26	19	69	305	24.5399		
ME2ad_3	QCR10p_4	RISP_8	11	7	28	4	3	2	14	10	15	1	1	3	2	1	1	3	1	10	4	17	28	7	3	15	24	21	70	306	24.4881		
P169_4	QCR7p_6	RPOL_7	9	12	10	2	6	12	23	7	26	5	1	3	0	0	3	2	3	3	22	17	37	0	5	16	23	16	51	314	24.4835		
P060_2	QCR10p_4	CYCad_6	9	6	13	3	0	9	10	10	18	8	7	11	8	0	2	20	2	15	12	4	28	5	2	9	36	12	45	304	24.4206		
mtMDH_2	P169_4	l34449_5	2	4	22	0	2	3	8	16	24	5	11	12	0	0	5	10	8	21	11	13	27	2	6	2	19	18	62	313	24.4029		
l22708_3	QCR7p_6	RPOL_7	4	8	17	0	5	7	17	10	29	7	1	3	0	1	0	4	2	5	25	21	30	2	5	23	27	15	44	312	24.3846		
X14140_3	QCR10p_4	QCR6p_9	6	10	33	3	3	2	8	3	27	1	2	6	0	0	4	3	3	9	13	8	21	3	5	18	20	19	75	305	24.3724		
mtMDH_2	QCR10p_4	CYCad_6	8	6	14	3	0	9	9	10	20	8	7	11	8	0	2	20	2	15	14	4	29	5	2	9	37	12	44	308	24.2996		
QCR9p_2	RPOL_7	l06422_10	0	12	8	0	2	10	7	13	25	3	8	17	4	6	8	2	7	26	6	7	24	4	16	18	13	24	43	313	24.2865		
P060_2	QCR10p_4	QCR7p_6	9	6	13	3	0	9	11	10	18	8	7	11	8	0	2	20	2	15	12	4	28	5	2	9	37	12	45	306	24.1662		
l22708_3	CYCad_6	RPOL_7	4	7	17	0	5	7	17	10	29	7	1	3	0	1	0	4	2	5	25	20	30	2	5	23	27	15	44	310	24.1651		
X14140_3	CYCad_6	cytMDH_10	8	4	14	6	3	5	7	18	32	1	1	8	0	2	0	5	4	7	17	25	34	4	6	18	10	25	46	310	24.1239		
ME2ad_3	P169_4	GOT2_8	13	6	30	0	0	5	16	12	12	1	1	3	1	0	0	5	3	9	9	10	34	6	2	6	32	29	66	311	24.102		
l34449_5	RPOL_7	GOT1p2_8	1	3	14	2	1	5	6	8	17	9	7	9	5	2	10	5	8	23	19	6	18	6	13	24	30	14	46	311	24.0366		
mtMDH_2	QCR10p_4	QCR7p_6	8	6	14	3	0	9	10	10	20	8	7	11	8	0	2	20	2	15	14	4	29	5	2	9	38	12	44	310	23.9749		
X14140_3	QCR10p_4	ME1ad_12	11	10	28	3	2	3	12	12	14	2	3	4	0	0	4	4	3	8	9	12	20	2	7	17	31	19	65	305	23.8936		
X14140_3	QCR10p_4	l34449_5	8	13	28	1	1	6	7	7	23	0	1	8	0	1	3	4	3	8	7	15	20	7	11	8	23	24	68	305	23.7995		
l34449_5	RPOL_7	GOT1p1_8	1	3	14	2	1	5	6	8	17	9	7	9	5	2	10	5	8	23	19	6	18	6	13	24	30	15	46	312	23.6759		
P060_2	P169_4	l34449_5	2	4	21	0	2	3	9	15	25	5	11	12	0	0	5	10	8	21	12	12	26	2	6	2	18	18	61	310	23.6149		
ME2ad_3	P169_4	GOT1Srg_5	8	10	31	1	0	4	9	12	19	0	2	3	1	0	0	4	3	10	11	16	26	2	8	4	25	29	73	311	23.5579		
GDHad_1	P169_4	l34449_5	4	5	23	0	3	5	7	13	28	4	3	1	1	0	1	1	3	17	11	20	37	1	5	4	29	26	62	314	23.5473		
CYCad_6	RPOL_7	cytMDH_10	8	14	14	10	4	14	9	11	28	1	0	1	2	5	4	6	6	19	10	14	24	2	11	14	10	22	49	312	23.5031		
RISP_8	QCR6p_9	l06422_10	1	6	11	0	7	3	10	6	28	0	2	14	1	2	8	7	9	24	3	6	14	3	15	16	14	41	63	314	23.4209		
QCR7p_6	GOT1p2_8	ME1ad_12	9	1	20	4	2	9	13	26	30	2	3	6	4	2	6	8	5	8	9	12	21	12	8	16	15	14	47	312	23.3986		
QCR8p_3	CYC1_4	GOT1p2_8	15	9	15	1	1	13	10	9	28	1	0	4	3	0	0	2	2	5	14	11	35	10	8	14	27	24	53	314	23.3779		
QCR10p_4	GOT1p1_8	l06422_10	2	9	12	0	9	12	12	13	32	0	2	12	0	2	5	4	5	8	8	10	27	5	8	21	7	34	49	308	23.3367		
l34449_5	GOT2_8	ME1ad_12	4	5	5	5	4	4	2	8	19	5	1	14	7	4	3	13	14	18	8	11	30	7	5	24	25	20	47	312	23.3015		
CYCad_6	GOT1p2_8	ME1ad_12	8	1	20	4	2	9	12	26	30	2	3	6	4	2	6	8	5	8	9	12	21	12	8	16	15	14	47	310	23.296		
QCR7p_6	GOT1p1_8	ME1ad_12	9	1	20	4	2	9	13	26	30	2	3	6	4	2	6	8	5	8	9	12	21	12	8	17	15	14	47	313	23.2822		
QCR10p_4	GOT1p2_8	l06422_10	2	9	12	0	9	12	12	13	32	0	2	12	0	2	5	4	5	8	8	10	27	5	8	20	7	34	49	307	23.274		
QCR10p_4	l34449_5	GOT1p2_8	3	1	11	6	6	17	14	14	29	0	2	6	8	1	4	6	4	7	6	9	19	5	9	20	34	14	50	305	23.2732		
CYCad_6	GOT1p1_8	ME1ad_12	8	1	20	4	2	9	12	26	30	2	3	6	4	2	6	8	5	8	9	12	21	12	8	17	15	14	47	311	23.1764		
QCR8p_3	CYC1_4	GOT1p1_8	15	9	15	1	1	13	10	9	28	1	0	4	3	0	0	2	2	5	14	12	35	10	8	14	27	24	53	315	23.1276		
mtMDH_2	P169_4	GOT1Srg_5	3	4	21	0	2	3	8	18	23	5	11	12	2	0	3	11	11	18	11	13	27	2	6	2	19	16	62	313	22.9639		
GDHad_1	P169_4	GOT1Srg_5	5	4	23	1	3	4	6	13	28	3	3	1	2	0	0	3	4	15	11	21	36	1	5	4	29	28	60	313	22.9091		
X14140_3	QCR10p_4	GOT1Srg_5	9	13	27	1	3	4	9	8	21	0	1	8	0	1	3	3	2	10	7	18	17	8	10	8	24	23	65	303	22.9023		
X14140_3	P169_4	RPOL_7	15	12	26	0	2	4	9	8	21	5	3	3	0	0	0	6	3	8	14	10	18	7	2	5	30	27	72	310	22.8657		
P060_2	P169_4	QCR7p_6	7	6	14	4	0	1	14	10	25	9	8	11	3	0	2	24	1	15	14	6	29	2	3	5	39	10	49	311	22.8508		
QCR10p_4	l34449_5	GOT1p1_8	3	1	11	6	6	17	14	14	29	0	2	6	8	1	4	6	4	7	6	9	19	5	9	20	34	15	50	306	22.8251		
ME2ad_3	P169_4	GOT1p1_8	15	7	27	0	0	5	14	12	14	1	1	3	1	0	0	8	3	6	8	11	34	5	2	7	31	27	69	311	22.8084		
ME2ad_3	P169_4	GOT1p2_8	15	7	27	0	0	5	14	12	14	1	1	3	1	0	0	8	3	6	8	12	34	5	2	7	31	26	69	311	22.8045		
QCR8p_3	QCR10p_4	GOT1p1_8	14	10	21	1	2	8	11	7	25	1	0	4	3	1	0	2	1	5	8	11	32	10	4	9	32	26	60	308	22.7706		
QCR10p_4	RPOL_7	cytMDH_10	12	5	13	3	7	14	7	16	24	3	6	5	2	1	2	3	5	11	4	17	20	8	13	16	15	17	59	308	22.635		
mtMDH_2	ME2ad_3	P169_4	10	3	15	1	0	5	17	2	28	16	0	8	1	1	2	11	4	29	23	2	17	3	0	10	25	8	71	312	22.6047		
P060_2	P169_4	CYCad_6	7	6	14	4	0	1	13	10	25	9	8	11	3	0	2	24	1	15	14	7	29	2	3	5	38	10	49	310	22.5815		
QCR7p_6	RPOL_7	cytMDH_10	8	14	14	10	5	15	9	11	28	1	0	1	2	5	4	6	6	19	10	14	24	2	11	14	10	22	49	314	22.575		
GDHad_1	mtMDH_2	l06422_10	2	8	17	3	10	5	7	14	23	0	2	0	1	2	6	6	4	10	9	14	30	4	10	34	7	31	58	317	22.547		
QCR8p_3	QCR10p_4	GOT1p2_8	14	10	21	1	2	8	11	7	25	1	0	4	3	1	0	2	1	5	8	11	32	10	4	9	32	25	60	307	22.5421		
P060_2	ME2ad_3	P169_4	9	3	15	1	0	5	17	2	29	16	0	8	1	1	2	11	4	29	22	2	17	2	0	10	26	8	70	310	22.5353		
GDHad_1	P169_4	ME1ad_12	12	6	13	2	0	6	10	13	24	0	2	5	0	2	0	6	2	14	16	19	33	4	1	5	25	28	65	313	22.5329		
ME2ad_3	QCR10p_4	GOT1p2_8	15	9	22	1	2	6	13	9	17	1	0	4	3	1	0	6	3	5	7	12	31	10	4	11	26	20	67	305	22.5232		
ME2ad_3	QCR10p_4	GOT1p1_8	15	9	22	1	2	6	13	9	17	1	0	4	3	1	0	6	3	5	7	12	31	10	4	11	26	21	67	306	22.4863		
GDHad_1	RPOL_7	cytMDH_10	6	6	11	4	9	8	2	11	30	2	5	5	0	0	3	8	3	5	11	17	23	10	12	22	15	25	61	314	22.3683		

GDHad_1	X14140_3	P169_4	16	3	12	5	0	4	11	5	32	6	0	3	0	0	3	1	2	15	31	3	23	6	0	10	30	7	83	311	22.2873
QCR8p_3	P169_4	I06422_10	5	15	24	1	2	2	8	13	30	4	1	0	0	1	0	0	5	6	6	18	34	1	5	8	14	34	78	315	22.2274
GDHad_1	GOT1p1_8	I06422_10	2	10	13	3	6	9	7	15	23	0	2	9	0	1	3	7	5	4	8	9	30	3	12	28	9	34	63	315	22.1735
QCR8p_3	CYC1_4	I06422_10	5	14	20	2	4	9	7	13	27	4	1	0	0	1	2	0	5	4	5	22	34	5	5	22	11	30	65	317	22.0949
GDHad_1	GOT1p2_8	I06422_10	2	10	13	3	6	9	7	15	23	0	2	9	0	1	4	7	5	4	8	9	30	3	12	27	9	34	63	315	22.0206
QCR8p_3	QCR10p_4	QCR6p_9	4	8	33	2	4	5	10	5	28	0	1	4	0	1	3	1	3	4	16	11	24	4	3	16	20	18	81	309	21.968
P060_2	I22708_3	P169_4	9	3	17	1	0	5	17	2	26	15	0	8	1	1	2	12	4	29	22	2	18	2	0	10	25	8	69	308	21.7479
I34449_5	QCR7p_6	RPOL_7	8	2	16	0	1	5	10	5	10	8	9	8	1	1	7	16	7	22	20	19	26	1	9	19	22	15	47	314	21.6648
ME2ad_3	P169_4	I34449_5	7	11	31	1	0	4	7	9	23	0	3	2	0	0	1	5	3	9	12	14	28	1	8	5	25	29	74	312	21.6508
mtMDH_2	I22708_3	P169_4	10	3	17	1	0	5	17	2	25	15	0	8	1	1	2	12	4	29	23	2	18	3	0	10	25	8	70	311	21.646
X14140_3	QCR7p_6	cytMDH_10	8	5	14	5	3	5	7	18	32	1	1	8	0	2	0	5	4	7	17	25	35	4	6	18	10	25	46	311	21.5938
mtMDH_2	CYC1_4	CYCad_6	9	5	13	3	2	5	10	9	25	7	6	8	8	0	3	21	3	19	15	4	38	12	3	14	30	12	31	315	21.5735
GOT1Srg_5	QCR7p_6	RPOL_7	8	2	17	0	1	6	11	5	11	11	9	9	1	1	7	15	6	23	17	19	24	1	9	18	22	16	44	313	21.5129
QCR9p_2	QCR10p_4	cytMDH_10	2	12	13	1	2	7	7	5	26	9	2	15	2	5	4	7	13	21	11	14	22	5	5	7	12	29	47	305	21.4608
QCR7p_6	RPOL_7	GOT1p2_8	12	3	21	6	5	19	12	7	30	1	0	1	4	4	3	6	8	17	16	13	19	3	7	17	23	16	40	313	21.4586
P060_2	P169_4	GOT1Srg_5	3	4	20	0	2	3	8	17	24	5	11	12	2	0	3	11	11	18	11	12	26	2	6	2	19	16	61	309	21.4481
I22708_3	QCR10p_4	GOT1p2_8	15	9	22	1	2	6	13	9	19	1	0	4	3	1	0	6	3	5	7	12	31	10	4	11	25	20	65	304	21.4311
QCR9p_2	P169_4	RISP_8	0	6	20	1	0	6	18	6	19	7	5	17	1	2	1	11	11	25	10	13	27	3	2	4	20	20	54	309	21.4191
I22708_3	QCR10p_4	GOT1p1_8	15	9	22	1	2	6	13	9	19	1	0	4	3	1	0	6	3	5	7	12	31	10	4	11	25	21	65	305	21.4164
ME2ad_3	CYC1_4	RISP_8	14	4	24	3	6	3	12	11	18	1	1	4	2	0	2	3	2	8	6	17	33	7	7	19	24	19	63	313	21.4078
CYCad_6	RPOL_7	GOT1p2_8	12	3	21	5	5	18	12	7	30	1	0	1	4	4	3	6	8	17	16	13	19	3	7	17	23	16	40	311	21.3864
P060_2	QCR8p_3	GOT2_8	6	8	16	1	2	2	14	12	19	11	8	6	2	0	2	10	11	25	8	4	33	4	0	3	27	19	59	312	21.3796
CYC1_4	I34449_5	GOT1p1_8	6	1	11	7	5	15	17	15	28	0	4	9	6	3	3	8	2	15	3	7	16	6	9	24	30	18	45	313	21.3586
QCR7p_6	RPOL_7	GOT1p1_8	12	3	21	6	5	19	12	7	30	1	0	1	4	4	3	6	8	17	16	13	19	3	7	17	23	17	40	314	21.3537
P102_3	CYCad_6	cytMDH_10	9	9	20	2	2	6	4	12	32	1	5	3	0	0	1	4	0	1	17	16	33	7	8	17	14	35	54	312	21.2944
CYCad_6	RPOL_7	GOT1p1_8	12	3	21	5	5	18	12	7	30	1	0	1	4	4	3	6	8	17	16	13	19	3	7	17	23	17	40	312	21.2775
P102_3	CYC1_4	GOT1p1_8	13	6	14	1	1	14	8	11	28	2	0	4	3	0	1	2	1	3	15	15	36	10	8	12	29	22	55	314	21.214
mtMDH_2	CYC1_4	QCR7p_6	9	5	13	3	2	5	11	9	25	7	6	8	8	0	3	21	3	19	15	4	38	12	3	14	31	12	31	317	21.1963
GDHad_1	QCR6p_9	I06422_10	3	8	7	1	8	4	8	16	34	0	0	7	1	2	3	6	6	6	1	7	25	2	14	20	17	34	76	316	21.1961
ME2ad_3	P169_4	QCR6p_9	9	9	31	1	3	1	9	5	26	0	0	5	0	1	0	1	4	12	14	10	29	2	2	10	22	21	85	312	21.1526
ME2ad_3	CYC1_4	GOT1p1_8	17	8	17	2	2	8	10	10	21	2	0	4	4	0	0	4	4	5	11	13	33	8	7	18	25	20	60	313	21.1364
QCR10p_4	I34449_5	ME1ad_12	1	4	9	6	11	12	16	10	31	1	3	4	2	1	10	2	5	10	9	10	15	16	6	13	22	18	60	307	21.135
ME2ad_3	QCR10p_4	GOT2_8	13	7	26	2	2	5	14	9	16	1	0	4	3	1	0	3	3	8	7	12	31	11	3	11	28	23	63	306	21.1129
P102_3	CYCad_6	QCR6p_9	8	6	25	2	0	8	7	10	30	0	5	4	1	0	0	0	0	5	12	13	42	4	4	24	22	17	64	313	21.1009
P102_3	CYC1_4	GOT1p2_8	13	6	14	1	1	14	8	11	28	2	0	4	3	0	1	2	1	3	15	14	36	10	8	12	29	22	55	313	21.1001
ME2ad_3	CYC1_4	GOT1p2_8	17	8	17	2	2	8	10	10	21	2	0	4	4	0	0	4	4	5	11	12	33	8	7	18	25	20	60	312	21.0667
I22708_3	QCR10p_4	RISP_8	11	8	27	4	3	2	14	10	17	1	1	3	2	1	1	3	1	10	4	16	29	7	3	15	23	21	68	305	21.0345
CYC1_4	QCR6p_9	I06422_10	0	9	10	1	8	10	13	20	34	1	1	6	1	6	2	5	3	24	3	5	23	2	10	15	13	33	58	316	21.0239

AD males

			Three Locus combinations (first/second/third locus genotype)																													Three Locus Totals	
first_locus	second_locus	third_locus	AA AA AA	AA AA DD	AA AA AD	AA DD AA	AA DD DD	AA DD AD	AA AD AA	AA AD DD	AA AD AD	DD AA AA	DD AA DD	DD AA AD	DD DD AA	DD DD DD	DD DD AD	DD AD AA	DD AD DD	DD AD AD	AD AA AA	AD AA DD	AD AA AD	AD DD AA	AD DD DD	AD DD AD	AD AD AA	AD AD DD	AD AD AD	Total #	chi-square value	Chromosome Combos	# p<0.05
GDHad_1	I22708_3	P169_4	3	5	14	2	1	1	6	6	40	2	8	4	0	0	3	5	0	5	13	9	16	1	0	5	16	17	58	240	33.4426	1,2,10	4
mtMDH_2	P102_3	P169_4	4	3	17	0	0	0	6	6	5	4	4	10	1	0	4	6	11	35	9	17	25	1	1	2	17	4	49	241	30.2713	1,3,4	2
QCR9p_2	RISP_8	I06422_10	5	2	6	8	8	6	6	0	12	7	2	7	8	6	7	16	2	10	13	3	11	11	2	13	11	13	41	236	30.2315	1,3,10	1
QCR9p_2	GOT1Srg_5	RPOL_7	6	0	8	4	0	5	9	7	14	8	0	9	9	2	7	13	10	10	11	8	10	7	11	8	20	6	39	241	30.0891	1,3,12	2
P060_2	P102_3	P169_4	4	3	17	0	0	0	6	6	5	4	4	10	1	0	4	6	11	35	9	17	26	1	1	2	17	4	48	241	29.8454	1,4,10	1
mtMDH_2	QCR8p_3	P169_4	5	3	15	1	0	0	4	6	7	3	4	6	0	1	3	8	10	40	9	17	24	1	0	2	17	5	50	241	29.6542	1,5,9	1
GDHad_1	QCR9p_2	cytMDH_10	1	4	12	6	0	14	4	6	31	1	2	3	8	1	2	0	5	5	7	6	17	15	5	16	26	7	36	240	29.6078	1,5,10	1
P060_2	QCR8p_3	P169_4	5	3	15	1	0	0	4	6	7	3	4	6	0	1	3	8	10	40	9	17	24	1	0	2	17	5	49	240	29.5735	1,7,10	1
GDHad_1	ME2ad_3	P169_4	3	5	15	2	1	2	6	6	38	2	8	4	0	0	2	5	0	6	12	9	16	1	0	6	17	17	58	241	28.6683	2,3,4	12
P169_4	GOT1Srg_5	cytMDH_10	4	0	6	7	2	6	1	8	14	8	0	6	1	1	2	10	6	12	8	8	19	8	4	23	21	7	49	241	28.5603	2,3,5	2
P060_2	QCR8p_3	QCR7p_6	7	4	13	0	0	1	1	2	14	2	2	9	0	0	4	5	16	37	8	17	25	0	2	1	20	17	35	242	27.4174	2,3,6	4
GDHad_1	mtMDH_2	cytMDH_10	1	3	8	5	0	18	5	7	32	1	0	2	7	2	2	1	6	6	8	5	12	15	7	19	25	6	38	241	27.3723	2,3,7	3
GDHad_1	P060_2	cytMDH_10	1	3	8	5	0	18	5	7	31	1	0	2	7	2	2	1	6	6	8	5	12	15	7	19	25	6	39	241	27.1666	2,3,8	11
P060_2	QCR8p_3	CYC1_4	5	3	15	1	0	0	4	5	8	5	5	3	0	3	1	8	17	33	6	13	31	1	0	2	16	17	39	241	26.6399	2,3,10	8
P060_2	QCR8p_3	RPOL_7	12	3	9	0	1	0	4	2	11	2	5	6	2	0	2	25	11	22	12	10	28	2	0	1	27	13	32	242	26.5638	2,3,12	1
I22708_3	CYC1_4	RISP_8	2	3	10	7	5	10	7	12	18	0	3	0	0	0	4	0	3	3	6	15	6	13	11	13	23	17	48	239	26.4161	2,4,8	2
mtMDH_2	QCR8p_3	CYC1_4	4	3	15	1	0	0	4	5	8	5	5	3	0	3	1	8	17	33	6	13	31	1	0	2	16	17	40	241	26.3453	2,4,9	1
QCR9p_2	QCR10p_4	GOT1p1_8	1	4	6	1	0	11	7	5	18	1	5	5	2	2	14	6	9	21	5	7	7	16	5	7	17	10	42	234	26.0836	2,4,10	2
QCR9p_2	QCR10p_4	GOT1p2_8	1	4	6	1	0	11	7	5	18	1	5	5	2	2	14	6	9	21	5	7	7	16	5	7	17	10	42	234	26.0836	2,5,6	6
QCR9p_2	GOT1Srg_5	I06422_10	4	1	9	5	0	4	10	9	11	12	2	3	8	5	5	12	3	16	5	1	22	10	4	12	20	13	32	238	26.0065	2,5,7	1
P060_2	QCR8p_3	CYCad_6	6	5	13	0	0	1	1	2	14	2	2	9	0	0	4	5	16	37	8	17	25	0	2	1	20	17	35	242	25.9308	2,5,10	1
P102_3	P169_4	ME1ad_12	3	5	9	6	6	12	22	10	18	1	0	1	0	1	0	4	1	1	8	8	13	7	7	7	9	23	57	239	25.9159	2,6,8	8
P102_3	QCR10p_4	GOT1p2_8	4	2	8	6	3	13	7	10	37	1	0	0	2	0	1	1	3	1	2	14	11	11	4	18	22	11	44	236	25.4725	2,7,10	2
GDHad_1	CYC1_4	cytMDH_10	3	2	5	3	2	17	5	6	36	2	0	4	3	3	1	4	5	5	9	3	17	20	3	11	19	12	41	241	25.4543	2,8,10	6
P060_2	QCR8p_3	I06422_10	10	2	11	0	1	0	6	3	8	6	0	7	1	2	1	23	9	25	10	7	32	3	0	0	27	14	30	238	25.4082	2,8,12	7
P102_3	QCR10p_4	GOT1p1_8	4	2	8	6	3	13	7	11	36	1	0	0	2	0	1	1	3	1	2	14	11	11	4	18	22	11	44	236	25.3892	3,4,8	12
ME2ad_3	CYC1_4	RISP_8	2	3	9	7	5	11	7	12	18	0	4	0	0	0	3	0	3	3	6	14	7	13	11	13	23	17	49	240	25.3543	3,4,12	2
P060_2	QCR8p_3	QCR10p_4	3	3	16	1	0	0	3	6	8	3	4	6	0	2	2	7	16	33	6	14	27	0	0	3	19	13	39	234	25.2485	3,5,12	3
mtMDH_2	QCR8p_3	QCR10p_4	3	3	16	1	0	0	3	6	8	3	4	6	0	2	2	7	16	33	6	14	27	0	0	3	19	13	40	235	25.1689	3,8,12	1
P102_3	QCR10p_4	GOT2_8	4	2	8	7	3	12	7	11	36	1	0	0	2	0	1	1	3	1	2	14	11	13	7	13	22	12	43	236	24.9236	4,5,10	3
mtMDH_2	QCR8p_3	RPOL_7	11	3	9	0	1	0	4	3	12	2	5	6	2	0	2	25	11	22	12	10	28	2	0	1	28	13	32	244	24.8313	4,5,12	1
QCR9p_2	QCR8p_3	cytMDH_10	4	7	16	0	0	2	5	6	14	7	0	6	1	2	1	22	4	25	9	11	27	0	0	2	21	7	43	242	24.635	4,9,10	1
mtMDH_2	QCR8p_3	CYCad_6	5	5	13	0	0	1	1	2	14	2	2	9	0	0	4	5	16	37	8	17	25	0	2	1	20	17	36	242	24.477	5,7,8	1
mtMDH_2	QCR8p_3	I06422_10	9	2	11	0	1	0	6	3	8	6	0	7	1	2	1	23	9	25	10	7	32	3	0	0	27	14	31	238	24.315	8,9,10	2
QCR8p_3	QCR10p_4	GOT2_8	3	2	7	6	3	12	5	7	37	0	0	1	0	0	2	1	3	1	4	14	11	16	7	12	24	15	42	235	24.1286		
QCR8p_3	P169_4	ME1ad_12	4	4	9	6	6	12	20	10	14	1	0	1	0	0	1	3	1	1	7	9	13	7	8	6	12	23	61	239	23.9076		
mtMDH_2	QCR10p_4	I06422_10	3	3	1	3	0	6	9	3	12	7	1	2	9	5	8	13	5	23	8	0	16	7	9	11	25	11	33	233	23.877		
P060_2	QCR10p_4	I06422_10	3	3	1	3	0	6	9	3	12	7	1	2	9	5	8	13	5	23	8	0	16	7	9	11	25	11	33	233	23.877		
P060_2	P102_3	I06422_10	10	2	12	0	0	0	6	4	7	7	1	10	1	2	2	22	8	21	9	9	33	4	0	0	27	12	30	239	23.6927		
mtMDH_2	QCR8p_3	QCR7p_6	6	4	13	0	0	1	2	3	14	2	2	9	0	0	4	5	16	37	8	17	25	0	2	1	20	17	36	244	23.6832		
QCR9p_2	I34449_5	CYCad_6	3	4	6	3	1	5	6	5	20	1	3	10	0	1	17	6	11	18	2	10	15	5	11	9	15	14	39	240	23.4085		
QCR9p_2	I34449_5	QCR7p_6	3	4	6	3	1	5	6	5	20	1	3	10	0	1	17	6	11	18	2	10	15	5	11	9	15	14	39	240	23.4085		
QCR9p_2	GOT1p1_8	I06422_10	3	1	5	6	2	1	10	7	18	5	0	4	6	5	5	20	5	15	14	8	15	6	1	15	15	9	36	237	23.3297		
QCR9p_2	GOT1p2_8	I06422_10	3	1	5	6	2	1	10	7	18	5	0	4	6	5	5	20	5	15	14	8	15	6	1	15	15	9	36	237	23.3297		
GDHad_1	QCR9p_2	I06422_10	4	2	11	10	1	9	11	12	18	2	1	3	6	1	4	2	2	6	13	7	10	15	8	11	22	4	42	237	23.2877		
P060_2	QCR8p_3	RISP_8	5	8	10	0	1	0	6	6	5	3	0	10	0	0	4	18	18	22	9	14	26	0	2	1	18	20	34	240	23.2855		
mtMDH_2	GOT1p1_8	ME1ad_12	0	3	3	1	2	2	13	5	11	4	1	7	4	6	4	8	15	26	12	6	21	6	5	17	12	18	28	240	23.2077		
mtMDH_2	GOT1p2_8	ME1ad_12	0	3	3	1	2	2	13	5	11	4	1	7	4	6	4	8	15	26	12	6	21	6	5	17	12	18	28	240	23.2077		
ME2ad_3	I34449_5	ME1ad_12	7	3	10	8	2	6	9	11	17	0	0	1	1	4	0	2	0	5	6	12	15	9	9	14	18	20	51	240	23.2024		
P060_2	QCR8p_3	GOT1p1_8	2	2	18	0	0	1	4	3	10	0	1	12	0	1	3	12	12	34	12	9	29	1	2	0	26	17	29	240	23.1355		
P060_2	QCR8p_3	GOT1p2_8	2	2	18	0	0	1	4	3	10	0	1	12	0	1	3	12	12	34	12	9	29	1	2	0	26	17	29	240	23.1355		
I22708_3	I34449_5	ME1ad_12	7	3	10	8	3	6	9	11	16	1	0	1	1	4	0	2	0	4	5	12	15	9	8	14	18	19	53	239	23.0509		
P060_2	GOT1p1_8	ME1ad_12	0	3	3	1	2	2	13	5	11	4	1	7	4	6	4	8	15	26	12	6	21	6	5	17	12	17	28	239	23.0153		
P060_2	GOT1p2_8	ME1ad_12	0	3	3	1	2	2	13	5	11	4	1	7	4	6	4	8	15	26	12	6	21	6	5	17	12	17	28	239	23.0153		
P060_2	QCR7p_6	GOT1p2_8	0	2	4	1	0	5	5	3	20	1	3	3	5	4	9	6	7	37	6	4	19	12	11	13	21	13	27	241	22.9417		
CYC1_4	I34449_5	ME1ad_12	5	3	2	1	6	6	5	6	11	5	3	9	2	3	4	9	11	17	3	9	15	15	6	10	15	14	45	240	22.9408		
mtMDH_2	GOT2_8	ME1ad_12	0	2	4	1	2	2	13	6	10	6	0	10	4	8	5	6	14	22	9	7	22	6	6	17	15	16	27	240	22.9362		

P060_2	GOT2_8	ME1ad_12	0	2	4	1	2	2	13	6	10	6	0	10	4	8	5	6	14	22	9	7	22	6	6	17	15	15	27	239	22.9285
P060_2	QCR8p_3	GOT2_8	3	2	18	0	0	1	4	3	10	2	1	10	0	1	3	14	15	29	10	9	31	1	2	0	27	18	27	241	22.87
P102_3	RISP_8	ME1ad_12	3	2	12	6	5	13	22	14	13	2	0	0	1	0	1	2	2	1	4	12	22	9	12	23	11	14	35	241	22.8304
mtMDH_2	QCR8p_3	GOT1p1_8	2	2	18	0	0	1	4	3	10	0	1	12	0	1	3	12	12	34	12	9	29	1	2	0	26	17	30	241	22.8299
mtMDH_2	QCR8p_3	GOT1p2_8	2	2	18	0	0	1	4	3	10	0	1	12	0	1	3	12	12	34	12	9	29	1	2	0	26	17	30	241	22.8299
QCR9p_2	P102_3	P169_4	4	5	18	0	0	1	11	6	9	5	4	8	1	0	3	7	9	30	8	15	26	1	1	2	10	6	50	240	22.764
P060_2	GOT1Srg_5	QCR7p_6	2	3	7	2	1	4	3	2	17	3	5	11	0	2	19	4	11	20	2	12	14	7	8	11	20	16	36	242	22.7399
QCR9p_2	QCR8p_3	GOT1p1_8	2	3	21	1	0	1	6	6	13	0	1	11	0	2	2	10	13	28	12	8	27	0	1	1	26	13	32	240	22.7037
QCR9p_2	QCR8p_3	GOT1p2_8	2	3	21	1	0	1	6	6	13	0	1	11	0	2	2	10	13	28	12	8	27	0	1	1	26	13	32	240	22.7037
mtMDH_2	P102_3	QCR10p_4	2	3	18	0	0	0	5	6	6	4	4	10	1	2	2	5	16	29	8	15	25	0	1	3	17	11	42	235	22.6666
QCR9p_2	GOT1p1_8	cytMDH_10	0	2	7	2	1	6	7	9	19	7	1	2	7	2	7	15	3	23	16	3	19	3	6	13	11	9	40	240	22.644
QCR9p_2	GOT1p2_8	cytMDH_10	0	2	7	2	1	6	7	9	19	7	1	2	7	2	7	15	3	23	16	3	19	3	6	13	11	9	40	240	22.644
QCR10p_4	GOT1Srg_5	cytMDH_10	4	1	5	7	1	6	1	4	13	10	1	4	2	2	4	11	7	17	5	6	20	7	4	21	19	10	44	236	22.6429
P060_2	QCR8p_3	GOT1Srg_5	6	6	11	0	0	1	6	1	10	5	1	7	1	1	2	13	19	26	15	11	24	1	2	0	12	13	47	241	22.6309
mtMDH_2	RPOL_7	l06422_10	5	0	10	1	3	1	9	3	8	10	5	13	6	1	9	14	5	11	18	4	19	10	2	11	12	15	33	238	22.585
P060_2	RPOL_7	l06422_10	6	0	10	1	3	1	9	3	8	10	5	13	6	1	9	14	5	11	18	4	18	10	2	11	12	15	33	238	22.5563
mtMDH_2	QCR8p_3	GOT2_8	3	2	18	0	0	1	4	3	10	2	1	10	0	1	3	14	15	29	10	9	31	1	2	0	27	18	28	242	22.5141
QCR9p_2	QCR8p_3	GOT2_8	3	3	21	1	0	1	8	7	10	3	1	8	0	2	2	12	16	23	9	8	30	0	1	1	25	13	33	241	22.5103
mtMDH_2	QCR8p_3	RISP_8	5	8	10	0	1	0	6	7	6	3	0	10	0	0	4	18	18	22	9	14	26	0	2	1	18	20	35	243	22.5023
P060_2	P102_3	QCR10p_4	2	3	18	0	0	0	5	6	6	4	4	10	1	2	2	5	16	29	8	15	26	0	1	3	17	11	41	235	22.3964
mtMDH_2	P102_3	l06422_10	9	2	12	0	0	0	6	4	7	7	1	10	1	2	2	22	8	21	9	9	32	4	0	0	27	12	31	238	22.3954
P060_2	CYCad_6	GOT1p1_8	0	2	4	1	0	5	5	3	20	1	3	3	5	4	9	6	7	37	6	4	18	12	11	13	21	14	27	241	22.3421
P060_2	GOT1Srg_5	CYCad_6	2	3	7	2	1	4	3	2	17	3	5	11	0	2	19	4	11	20	2	12	14	7	8	11	19	16	37	242	22.2187
mtMDH_2	GOT1Srg_5	CYCad_6	1	3	7	2	1	4	3	2	17	3	5	11	0	2	19	4	11	20	2	12	15	7	8	11	19	16	36	241	22.2155
mtMDH_2	GOT1Srg_5	QCR7p_6	1	3	7	2	1	4	3	2	17	3	5	11	0	2	19	4	11	20	2	12	15	7	8	11	19	16	36	241	22.2155
mtMDH_2	QCR8p_3	GOT1Srg_5	5	6	11	0	0	1	6	1	10	5	1	7	1	1	2	13	19	26	15	11	24	1	2	0	13	13	47	241	22.2146
QCR8p_3	CYC1_4	RISP_8	2	3	10	6	5	10	8	14	26	0	2	0	0	0	3	0	1	2	6	16	6	14	11	14	22	17	42	240	22.2111
QCR9p_2	P102_3	cytMDH_10	4	7	16	0	0	1	5	6	15	8	0	10	2	2	0	20	4	22	9	11	29	2	0	2	19	7	41	242	22.1581
QCR9p_2	RISP_8	ME1ad_12	0	5	8	7	5	11	11	3	5	2	3	12	6	7	8	7	8	14	7	6	13	3	5	18	17	19	30	240	22.1562
GDHad_1	QCR8p_3	ME1ad_12	12	4	11	0	0	2	8	9	32	2	6	4	2	0	0	3	5	5	16	10	20	2	1	1	15	26	44	240	22.0732
RISP_8	QCR6p_9	l06422_10	5	3	4	2	2	9	18	2	11	8	2	8	2	3	5	17	11	13	5	4	6	9	2	13	19	9	45	237	22.0122
GDHad_1	l34449_5	QCR6p_9	5	2	10	3	3	11	9	15	20	0	0	6	0	4	4	5	2	6	4	7	20	6	2	20	14	13	50	241	22.0079
P060_2	QCR8p_3	ME1ad_12	11	5	7	1	0	0	3	5	9	2	4	7	1	1	2	13	17	28	18	11	21	2	0	1	10	17	44	240	21.982
QCR9p_2	QCR8p_3	P169_4	6	5	16	1	0	1	8	6	11	4	4	4	0	1	3	9	8	34	7	15	25	1	0	1	11	7	52	240	21.9315
QCR9p_2	l22708_3	l06422_10	6	4	10	1	1	1	12	5	12	8	0	5	2	4	0	21	6	19	9	3	28	1	1	2	25	14	36	236	21.8725
mtMDH_2	CYCad_6	GOT1p1_8	0	2	4	1	0	5	5	3	20	1	3	3	5	4	9	6	7	37	6	4	18	12	11	13	21	13	28	241	21.8544
mtMDH_2	CYCad_6	GOT1p2_8	0	2	4	1	0	5	5	3	20	1	3	3	5	4	9	6	7	37	6	4	18	12	11	13	21	13	28	241	21.8544
mtMDH_2	QCR7p_6	GOT1p1_8	0	2	4	1	0	5	5	3	20	1	3	3	5	4	9	6	7	37	6	4	18	12	11	13	21	13	28	241	21.8544
mtMDH_2	QCR7p_6	GOT1p2_8	0	2	4	1	0	5	5	3	20	1	3	3	5	4	9	6	7	37	6	4	18	12	11	13	21	13	28	241	21.8544
P060_2	CYCad_6	GOT1p2_8	0	2	4	1	0	5	5	3	20	1	3	3	5	4	9	6	7	37	6	4	18	12	11	13	21	13	28	241	21.8544
QCR8p_3	QCR10p_4	GOT1p1_8	3	2	7	5	3	13	6	7	36	0	0	1	0	0	2	1	3	1	4	14	11	14	4	17	23	14	44	235	21.8432
QCR8p_3	QCR10p_4	GOT1p2_8	3	2	7	5	3	13	6	7	36	0	0	1	0	0	2	1	3	1	4	14	11	14	4	17	23	14	44	235	21.8432
P169_4	l34449_5	cytMDH_10	3	1	6	6	1	6	3	8	14	7	0	5	2	1	3	10	6	12	7	8	17	7	4	23	23	7	51	241	21.8131
GDHad_1	l22708_3	cytMDH_10	3	5	14	0	0	4	8	5	40	5	3	6	0	2	1	4	3	3	11	6	21	1	1	4	35	11	45	241	21.7873
GOT1Srg_5	RPOL_7	RISP_8	4	8	12	3	3	2	6	5	16	4	10	6	5	1	7	5	8	8	12	6	23	9	9	5	10	19	34	240	21.7775
P060_2	QCR7p_6	GOT1p1_8	0	2	4	1	0	5	5	3	20	1	3	3	5	4	9	6	7	37	6	5	18	12	11	13	21	13	27	241	21.7093
X14140_3	l34449_5	ME1ad_12	7	3	10	7	1	6	9	11	18	0	1	2	1	4	0	1	0	6	6	11	14	10	10	14	19	20	49	240	21.5812
QCR9p_2	QCR8p_3	RPOL_7	12	3	12	0	1	1	7	5	15	5	4	4	3	0	1	22	8	21	9	11	27	1	0	1	28	14	29	244	21.5731
X14140_3	P169_4	RISP_8	3	6	8	8	3	10	6	11	19	0	4	0	0	0	1	1	3	6	7	12	8	6	8	9	28	21	52	240	21.5607
RISP_8	QCR6p_9	cytMDH_10	1	3	8	6	1	7	17	1	13	6	3	9	2	4	4	9	3	29	2	3	11	6	5	13	19	13	42	240	21.5169
QCR9p_2	P169_4	QCR6p_9	0	1	14	4	3	4	5	8	14	3	3	7	3	3	7	10	7	24	7	4	8	5	4	13	9	13	55	238	21.4282
GDHad_1	GOT1Srg_5	cytMDH_10	5	4	11	1	0	16	5	6	31	1	2	2	4	1	3	4	5	5	14	2	18	11	6	12	23	10	40	242	21.2683
QCR9p_2	RISP_8	cytMDH_10	2	4	8	4	5	13	3	4	11	9	2	6	8	1	12	12	3	14	13	0	14	5	4	17	12	14	40	240	21.2426
QCR8p_3	CYC1_4	GOT2_8	3	3	9	4	4	13	7	5	37	0	1	1	0	0	3	1	2	0	5	13	10	16	8	15	24	15	42	241	21.2246
ME2ad_3	QCR10p_4	RISP_8	2	3	8	8	3	12	6	14	18	0	2	0	0	0	2	0	5	5	7	13	7	11	10	12	23	18	47	236	21.1937
GDHad_1	RPOL_7	cytMDH_10	5	1	24	2	2	11	4	7	23	4	4	2	0	0	3	5	4	5	20	8	18	8	3	15	20	7	36	241	21.1645
CYC1_4	QCR6p_9	cytMDH_10	4	0	6	3	1	2	7	4	18	0	3	8	4	2	5	22	3	16	5	6	14	7	7	17	16	10	50	240	21.137
GDHad_1	l22708_3	ME1ad_12	7	3	11	0	1	3	13	9	31	3	7	4	2	0	1	2	4	4	14	7	17	2	3	1	17	26	47	239	21.0991
mtMDH_2	QCR8p_3	cytMDH_10	4	6	13	0	0	1	6	3	8	5	1	7	0	2	2	22	6	30	10	11	29	1	0	2	20	8	45	242	21.0769
