# Supplementary material for: Chromosome-Wide Impacts on the Expression of Incompatibilities in Hybrids of Tigriopus californicus
Source: G3 (Bethesda). 2016 Apr 11;6(6):1739–49. doi: 10.1534/g3.116.028050 (PMC4889669; doi:10.1534/g3.116.028050)
Supplement: Supplemental Material [file supp_g3.116.028050_TableS6.pdf]

Supplemental Table 6. First and second day nauplii genotypes and statistical tests.

Locus		3QCR8p			3FBLRR			11sep_tub			c8_3336	
iPlex marker		3b			3d			11				
Age	first day	second day	adult	first day	second day	adult	first day	second day	adult	first day	second day	adult
AB/AB	32	34	70	32	42	69	36	39	62	29	21	42
SD/SD	56	18	3	58	34	3	43	39	34	29	36	53
Het.	72	52	153	64	60	155	86	61	131	102	82	130
total	160	104	226	154	136	227	165	139	227	160	139	225
1:2:1 chisq	8.8	4.92307692	68.0442478	13.1688312	2.82352941	68.7268722	0.89090909	2.07913669	12.3039648	12.1	7.73381295	6.52
AB rel viabilit	0.87671233	1.28301887	0.90909091	0.98461538	1.37704918	0.88461538	0.82758621	1.25806452	0.93939394	0.5631068	0.5060241	0.64122137
std dev rv	0.17230965	0.27885395	0.12189274	0.20098981	0.27619309	0.11843388	0.15052359	0.25327236	0.13530419	0.10248219	0.10555988	0.10020905
SD rel viab	1.53424658	0.67924528	0.03896104	1.78461538	1.1147541	0.03846154	0.98850575	1.25806452	0.51515152	0.5631068	0.86746988	0.80916031
std dev rv	0.2774543	0.16541635	0.01641921	0.33678878	0.23029552	0.01620225	0.17406906	0.25327236	0.08466065	0.10248219	0.16011787	0.12030088
chi-square 1d/2d or 2d/ad		11.44	32.2		6.65	55.2		2.36	10.55		2.75	0.84
P value		0.0033	0		0.036	0		0.31	0.0056		0.25	0.66
