# Supplementary material for: Chromosome-Wide Impacts on the Expression of Incompatibilities in Hybrids of Tigriopus californicus
Source: G3 (Bethesda). 2016 Apr 11;6(6):1739–49. doi: 10.1534/g3.116.028050 (PMC4889669; doi:10.1534/g3.116.028050)
Supplement: Supplemental Material [file supp_g3.116.028050_TableS7.pdf]

Supplemental Table 7. Observed genotypes at two markers in each of eight possible backcrosses. The signficance of deviations from expected 1:1 genotypic ratio is indicated [illegible] chi-square values with a Bonferroni correction for 8 tests per marker would be values exceeding 7.5.

	Chromosome 3 (ME2, 3d)						Chromosome 8 (GOT2, 8d)					
Backcross	AB/SD	SD/SD	AB/SD	SD/SD	exp#	Chi-Sq	AB/SD	SD/SD	AB/SD	SD/SD	exp#	Chi-Sq
DA x SDm	53	1	0.98148148	0.01851852	27	50.0740741	14	19	0.42424242	0.57575758	16.5	0.75757576
DA x SDf	36	36	0.5	0.5	36	0	40	37	0.51948052	0.48051948	38.5	0.11688312
AD x SDm	186	20	0.90291262	0.09708738	103	133.76699	10	14	0.41666667	0.58333333	12	0.66666667
AD x SDf	26	17	0.60465116	0.39534884	21.5	1.88372093	29	19	0.60416667	0.39583333	24	2.08333333
DA x ABm	22	21	0.51162791	0.48837209	21.5	0.02325581	25	17	0.5952381	0.4047619	21	1.52380952
DA x ABf	24	24	0.5	0.5	24	0	29	19	0.60416667	0.39583333	24	2.08333333
AD x ABm	65	54	0.54621849	0.45378151	59.5	1.01680672	28	18	0.60869565	0.39130435	23	2.17391304
AD x ABf	16	29	0.35555556	0.64444444	22.5	3.75555556	25	23	0.52083333	0.47916667	24	0.08333333
